# Improving mapping and SNP-calling performance in multiplexed targeted next-generation sequencing

**DOI:** 10.1186/1471-2164-13-417

**Published:** 2012-08-22

**Authors:** Abdou ElSharawy, Michael Forster, Nadine Schracke, Andreas Keller, Ingo Thomsen, Britt-Sabina Petersen, Björn Stade, Peer Stähler, Stefan Schreiber, Philip Rosenstiel, Andre Franke

**Affiliations:** 1 Institute of Clinical Molecular Biology, Christian-Albrechts-University, Kiel, Germany; 2 Febit biomed GmbH, Heidelberg, Germany; 3 Biomarker Discovery Center, Heidelberg, Germany; 4 Department of General Internal Medicine, Campus Kiel, University Hospital S.-H., Kiel, Germany

**Keywords:** Two-stage mapping, Read-backmapping, Software performance, SNP discovery, Multiplexed targeted next-generation sequencing

## Abstract

**Background:**

Compared to classical genotyping, targeted next-generation sequencing (*t*NGS) can be custom-designed to interrogate entire genomic regions of interest, in order to detect novel as well as known variants. To bring down the per-sample cost, one approach is to pool barcoded NGS libraries before sample enrichment. Still, we lack a complete understanding of how this multiplexed *t*NGS approach and the varying performance of the ever-evolving analytical tools can affect the quality of variant discovery. Therefore, we evaluated the impact of different software tools and analytical approaches on the discovery of single nucleotide polymorphisms (SNPs) in multiplexed *t*NGS data. To generate our own test model, we combined a sequence capture method with NGS in three experimental stages of increasing complexity (*E. coli* genes, multiplexed *E. coli*, and multiplexed HapMap *BRCA1/2* regions).

**Results:**

We successfully enriched barcoded NGS libraries instead of genomic DNA, achieving reproducible coverage profiles (Pearson correlation coefficients of up to 0.99) across multiplexed samples, with <10% strand bias. However, the SNP calling quality was substantially affected by the choice of tools and mapping strategy. With the aim of reducing computational requirements, we compared conventional whole-genome mapping and SNP-calling with a new faster approach: target-region mapping with subsequent ‘read-backmapping’ to the whole genome to reduce the false detection rate. Consequently, we developed a combined mapping pipeline, which includes standard tools (BWA, SAMtools, etc.), and tested it on public HiSeq2000 exome data from the 1000 Genomes Project. Our pipeline saved 12 hours of run time per Hiseq2000 exome sample and detected ~5% more SNPs than the conventional whole genome approach. This suggests that more potential novel SNPs may be discovered using both approaches than with just the conventional approach.

**Conclusions:**

We recommend applying our general ‘two-step’ mapping approach for more efficient SNP discovery in *t*NGS. Our study has also shown the benefit of computing inter-sample SNP-concordances and inspecting read alignments in order to attain more confident results.

## Background

Correlations between genotype and phenotype variations have traditionally been studied by determining the genotype of known markers. For example, genome-wide association studies (GWAS) have revealed associations of known common variants with several of the common diseases. But these associations typically explain less than 25% of the heritable risk estimated for each of those diseases [1,2]. This is a serious limitation for complex diseases, which are often genetically heterogeneous [3]. Existing GWAS data suggest that rare alleles also have a significant influence on common diseases [4]. Therefore, targeted resequencing of suspected exonic, intronic, and intergenic loci mapped by GWAS and linkage studies is the next logical consequence to identify the entire underlying genetic variation and its disease relevance.

Target enrichment methods rely either on PCR or sequence-specific nucleic acid hybridization, and each method has unique advantages and disadvantages [5-7]. The combination of sample enrichment methods and next-generation sequencing (NGS) pipelines is an effective analysis approach, but it also raises some important questions: How accurate is the sequence variation discovery within the targeted NGS (*t*NGS) data? What is the effect of the varying performance of the rapidly evolving alignment and analytical tools? Can a sample multiplexing approach reduce the analysis and study costs? To address these questions, as a test model, we combined a hybridization-based sample enrichment method (Febit biomed GmbH, Germany) with an NGS platform (SOLiD, Life Technologies, USA). With sample mix-up control and enrichment cost reduction in mind, we evaluated the enrichment of barcoded SOLiD libraries (individual and pooled), rather than preparing NGS libraries after enrichment [8-10]. Individual samples are indexed by inserting unique nucleotide signatures, the barcodes, and are then pooled together before enrichment so that the pooled DNA samples can be sequenced en-bloc. Sample identity is then re-established during the bioinformatic processing of the reads after sequencing. Sample barcoding has the advantage – in contrast to a simple pooling approach – that genotypes can be assigned at an individual level and that even rare variants can be identified with a high sensitivity [4,5,8,11-14]. We tested the applicability of the evaluated *t*NGS method on *E. coli*, as a simple model to optimize the process*,* and human genomic samples in three experimental stages of increasing scope and complexity, culminating in a SNP concordance evaluation for the *BRCA1* and *BRCA2* cancer genes.

To identify a suitable analytical approach in terms of computational time and accuracy of sequence variant detection in *t*NGS data, we carefully analyzed the data using different software and tools. We found that SNP detection depends strongly on the chosen analytical tools and settings, rather than on key enrichment measures such as high and uniform coverage, the percentage of reads mapped on target, or an adequate enrichment fold. Unexpectedly, all evaluated tools failed to identify a large proportion of true sequence variations (false negative SNPs). Whole-genome (WG) mapping/SNP-calling was time-consuming, but with the benefit of few false positive SNPs. Target region (TR) mapping/SNP-calling was faster and yielded more true SNPs than WG mapping, but led to a high proportion (up to 50%) of likely false positive SNPs. Target region mapping can force-map reads from other genomic loci into the target, leading to false positive SNPs and requiring a postprocessing cleanup. To benefit from both methods, we here report a novel ‘two-step’ mapping approach that starts with TR mapping/SNP-calling, followed by backmapping only the SNP-supporting reads to the WG. Because of the recent wide interest in whole exome sequencing, we also applied our approach to public human exome enrichment data generated by Illumina HiSeq instruments. Moreover, our report includes detailed practical instructions, such as validating SNPs by computing inter-sample SNP-concordances between multiplexed technical replicates, filtering for novel SNPs, or establishing and evaluating a *t*NGS method.

## Results

**Figure 1:**
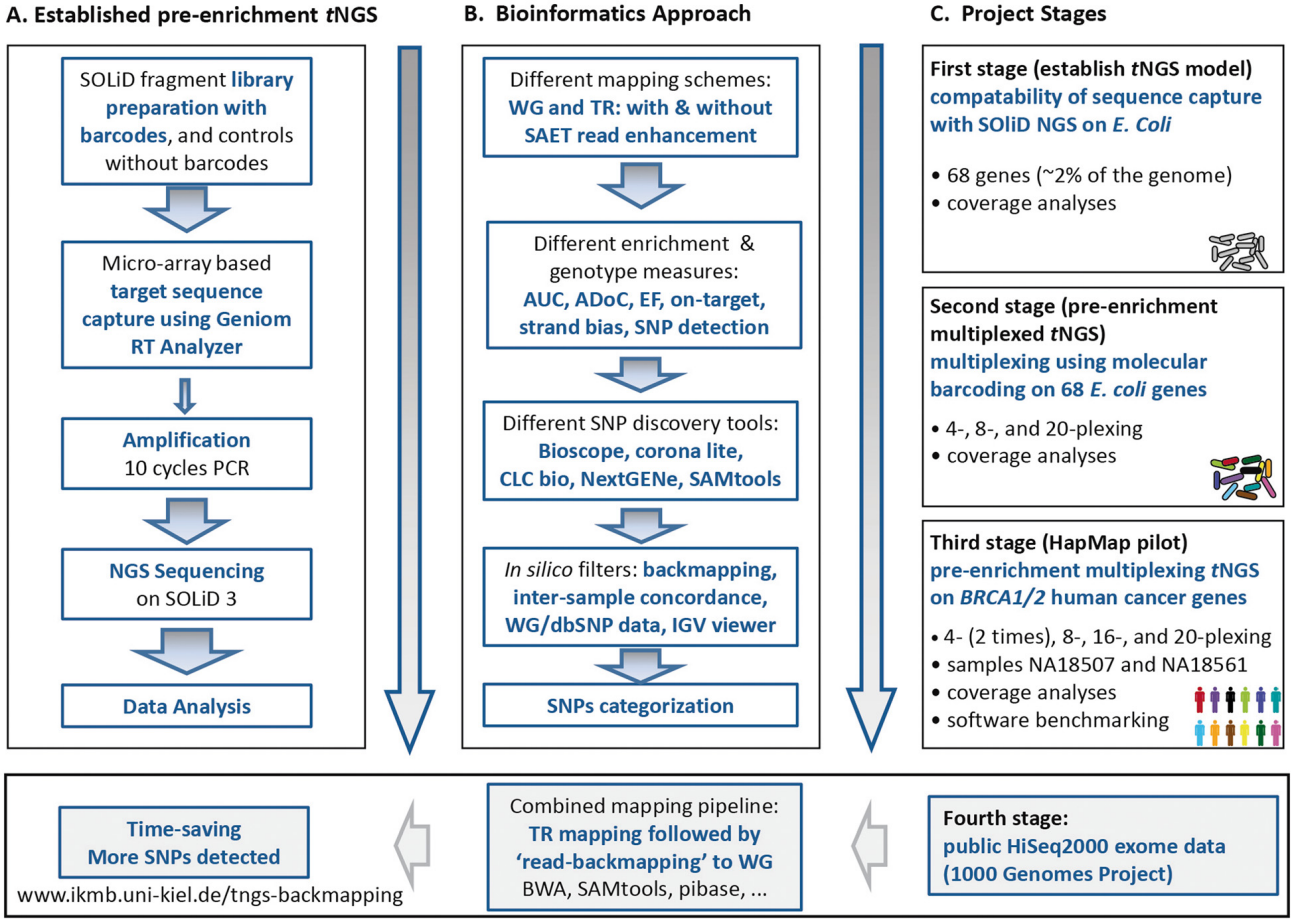
** Our *t*NGS test model, study design and established workflow.** Panel **A** summarizes the established workflow, while panel **B** lists the bioinformatics approach. In panel **C**, the three sequential stages are summarized. The first stage represents the proof-of-principle on *E. coli* enrichment, the second stage evaluated the multiplexing capacities, and the third stage demonstrated the application of the pipeline on clinically relevant human target genes. For the data analysis, we tested different mapping tools and approaches as shown in this Figure. (WG stands for whole genome, TR for target region, SAET for Spectral Analysis Enhancement Tool, AUC for area under the ROC curve, ADoC for average depth of coverage, and EF for enrichment factor). Finally, we applied our TR/backmapping pipeline to public exome data (lower panel).

We developed a novel, fast two-stage backmapping method in the course of three experimental and analytical stages (our test *t*NGS model) and a fourth, purely analytical stage using human exome data. The study design, the established wet-lab workflow, and the bioinformatics workflows for our test *t*NGS model are presented in Figure 1. In the first stage, we successfully enriched different SOLiD libraries instead of gDNA of *E. coli* for 68 genes and sequenced them on a SOLiD NGS platform. In the second stage, we enriched three pools of barcoded libraries of *E. coli* for the same 68 genes and sequenced these on the same NGS platform. The results of this stage showed reproducible uniform coverages and enrichment folds for most barcodes and multiplexes tested. Full details on the target enrichment experiments and results, especially the wet-lab procedure, can be found in the Additional file 1. In the following results section we concentrate on the respective data analysis and tool development.

### Pre-enrichment multiplexing of NGS libraries of *BRCA1/2* genes and data analysis

**Figure 2:**
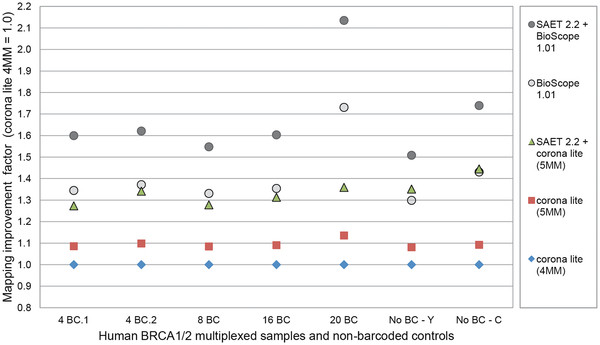
** Mapping improvements using different bioinformatics tools and settings for the same raw sequencing data (third stage of our multiplexed *t*NGS test model).** This figure illustrates the mapping performance of different tools for the human *BRCA1* and *BRCA2* sequence reads. From Corona Lite’s default mapping (4 mismatches in 50 colors) to SAET 2.2 read enhancement and subsequent Bioscope 1.0.1 mapping, the number of uniquely mappable reads increased by about 60%. SAET alone improved mappable reads by about 15%-20% and Bioscope improved mappable reads by about 35%-75%. Bioscope 1.0.1 improves coverage, because it can map a sub-segment of a read. As Corona Lite mapped less reads to the target for the 20-plex spot and the non-barcoded Chinese control spot, Bioscope’s segment-mapping approach over-proportionally increased mapped reads by a factor of 2.13 and 1.74.

In the third stage, we focused on enriching pools of barcoded libraries of HapMap individuals for non-repeat-masked regions of two clinically relevant human cancer genes (*BRCA1* and *BRCA2*). We enriched these genomic regions at different multiplexing levels (two 4-plex pools with two different 4-barcode combinations, 8-, 16- and 20-plex). We also included two non-barcoded controls. The sequencing results and enrichment measures are summarized in Additional file 2: Table S1 and Additional file 3: Figure S1. For the data analysis we used several tools, partly because the original tools turned out to be insufficient and incompatible with newer tools, and partly to benchmark the performance. We used the SOLiD Spectral Analysis Enhancement Tool (SAET 2.2; [15]) for correcting sequencing errors in the raw reads, and evaluated the coverage and SNP-calls with and without the use of SAET. We initially used SOLiD Corona Lite for mapping and diBayes for SNP-calling, but after achieving only insufficient results, we turned to SOLiD BioScope for mapping and SNP-calling. We validated the mapping and SNP-calling using CLC bio Genomics Workbench 3.7.1 software (CLC bio, Aarhus, Denmark) and the NextGENe V2 software (SoftGenetics, State College, PA, USA). We used SAMtools [16] for data format conversion, pibase [17] for automatic validations, and IGV [18] for viewing mapped reads. As a first result, we found that SAET increased the coverage by about 15-20% (Figure 2), but that the run time was very high (8 hours for the Yoruban control in the octant spot containing 35 million reads) and that the automatic SNP discovery rate was slightly decreased (3-19 percentage points, see Additional file 2: Table S2 and Table S3). Bioscope with SAET mapped up to 113% more reads than Corona Lite without SAET as shown in Figure 2. It also illustrates that the SAET error-correction combined with the Bioscope mapping led to the highest number of mapped reads and the highest coverage for the SOLiD data. Secondly, as expected, the TR mapping/SNP-calling approach required far less time than the conventional WG approach. Thirdly, we found that the resulting average coverages and enrichment factors were reproducible within each pool (more details in the Additional file 1 and Additional file 2: Table S1). The average depth of coverage (ADoC) was 407×, 330×, 187× and 30× for the 4-, 8-, 16-, and 20-plex experiments, respectively. At 8× coverage – a minimum SNP detection threshold commonly employed in NGS studies – 98.3%, 94.1%, 78.8%, 71.0% and 51.7% of the targeted bases were covered in the control libraries, 4-, 8-, 16- and 20-plex, respectively (Additional file 2 Table S4). Overall, the enrichment process was efficient, with regard to sensitivity and specificity, as indicated by the area under curve (AUC) values of 0.989 and 0.988 for the control libraries, and 0.976, 0.911, 0.897 and 0.856 for the 4-, 8-, 16- and 20-plex libraries, respectively (Additional file 3: Figure S2). Full details on the enrichment metrics can be found in the Additional file 1. 

### Genotype concordance and overlap analysis in the *BRCA1/2* experiments

**Figure 3:**
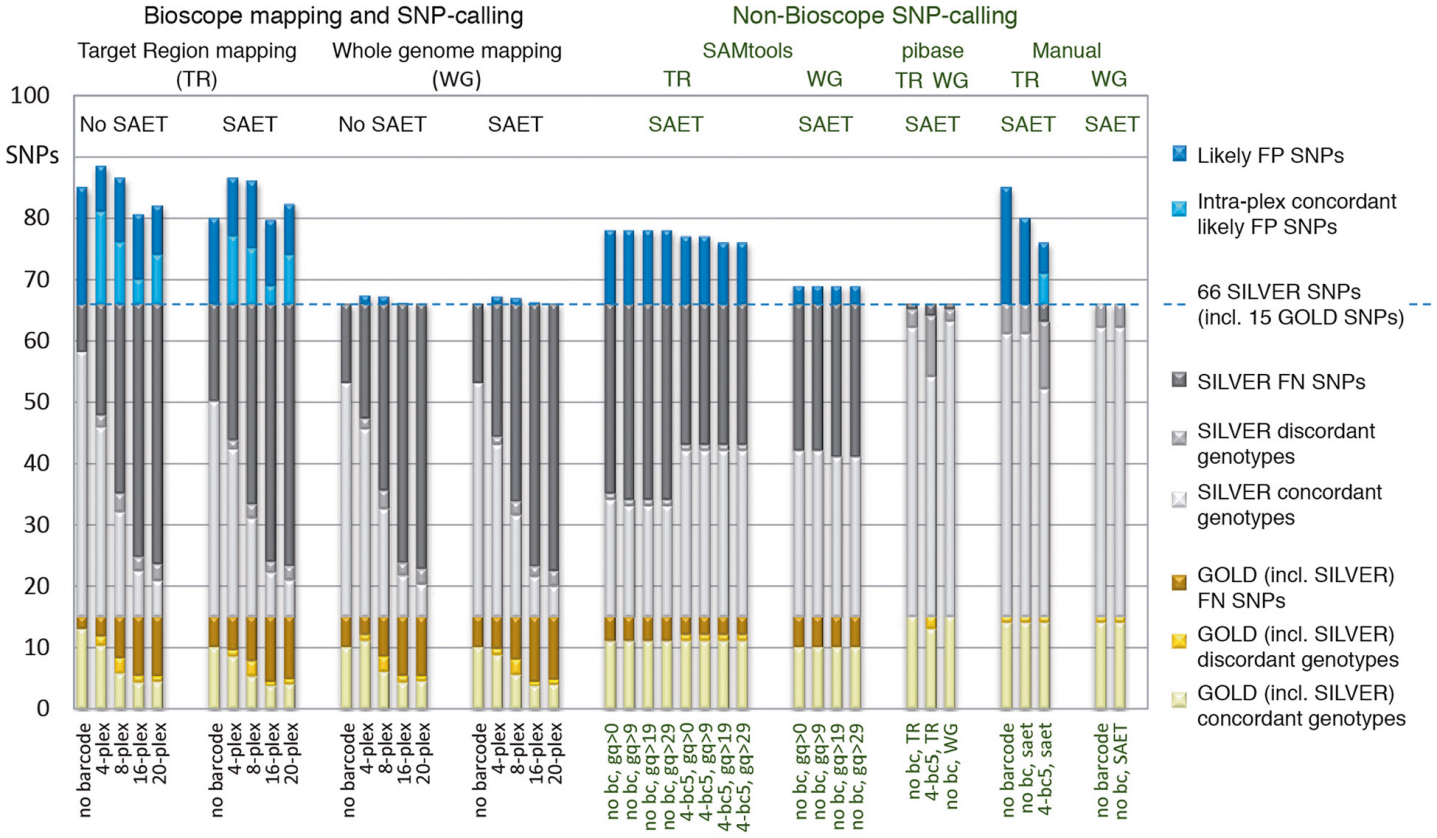
** SNP metrics for different multiplexes and analysis strategies (third stage of our multiplexed *t*NGS test model).** This figure summarizes the SNP detection and genotype concordance results of the human *BRCA1* and *BRCA2* experiments. The results are detailed for known SNPs within the TR for the 26 barcoded Yoruban samples (two 4-plexes, one 8-, 16- and 20-plex) and the non-barcoded control. The results shown in this figure were generated using Bioscope 1.0.1 (mapping, SNP-calling), SAET (pre-mapping read enhancement), SAMtools (SNP-calling), pibase (SNP-typing), and IGV (manual inspection). The identified SNPs were benchmarked against a ‘silver’ and a ‘gold’ consensus of published and validated genotypes (see Methods section). Known SNPs which we failed to detect with a specific tool are counted as false negatives (FN, dark grey for the ‘silver’ consensus and brown for the ‘gold’ consensus). Potential novel SNPs which could not be validated with certainty are counted as false positives (FP, blue for SNPs in only one library and light blue for SNPs in several libraries). The first four groups of columns show the automatic SNP-calling results, and the rightmost two groups show the manual inspection results. For TR mapping (first two groups), Bioscope detected more known SNPs than for whole-genome mapping (middle groups, no barcode, 4-plex) and also more FP SNPs. With SAET read enhancement Bioscope detected fewer known SNPs (second and fourth group, TR 4-plex and no barcode, WG 4-plex) than without SAET (first and third group). The SAMtools SNP-caller (fifth and sixth groups) performed worse than Bioscope. Our pibase re-analysis (seventh group) and manual inspection in IGV (rightmost two groups) revealed that the Bioscope and SAMtools SNP-callers filtered out known SNPs which were detected with pibase and also seen in the mapped reads. A detailed description is given in the Results section. In summary, to automatically detect SNPs with minimal manual filtering of false positives or false negatives, and to take advantage of short run times, we recommend a combination of multiplexing technical replicates, mapping to the TR, and backmapping the reads covering the SNPs to the WG (see Conclusion section).

The identification of sequence variants in targeted region(s) is a typical objective of resequencing. To test how well variants are discovered using our multiplexed *t*NGS model, we benchmarked different analysis strategies and focused on SNPs, the most abundant form of variation. Our SNP discovery results for selected multiplexes and different analysis strategies are summarized in Figure 3 and Additional file 2: Table S5. The initial SNP calling results using Corona Lite were not promising (Additional file 2: Table S6). We therefore reran the mapping and SNP-calling stages using Bioscope. We compared the SNP overlap and genotype concordance for several data processing strategies. Additional file 2: Table S7 shows a genotype concordance rate of 100% for the non-indexed control samples. The 4-, 8-, 16- and 20-plex experiments yielded an average genotype concordance rate of up to 98%, 79%, 80% and 55%. It was surprising to us that Bioscope consistently overlooked a few known ‘gold’ standard SNPs (for the definition refer to the Methods section) despite high coverages at the respective SNP positions in all Yoruban samples (Additional file 2: Table S2 and Table S3). The Bioscope consensus call output file gave more details than the Bioscope SNP file, reporting code ‘h15’ for rs206119 (which translated into: ‘genome position has low quality, i.e. needs more and longer reads to map this low-complexity region’) and codes ‘h4, h10, h9’ for rs206123 (‘too many invalid dicolors found, no conclusive second allele found, tri-allelic SNP’). We therefore complemented our analysis with CLC bio, NextGENe, pibase and IGV. We streamlined our manual genotype calling in IGV by classifying a genotype as homozygous if more than 80% of the reads indicated the same base at that position. Otherwise we classified it as heterozygous [8]. 

For the Yoruban sample we observed a good SNP overlap (15/15) and reached a concordance of 14/15 through visual inspection in IGV. Bioscope called only 13/15 SNPs with the chosen settings and also with many alternative settings (Bioscope_settings.xls at [19]). Our manual inspection of the mapped reads at the false negative SNP positions clearly showed a non-reference allele consensus, i.e. a SNP (Additional file 3: Figure S3 and Additional file 2: Table S8). The CLC bio analysis (sample “4plex-1 bc05”, a sample from the first 4-plex with barcode 5) called 15/15 SNPs with a concordance of 13/15 (or 15/15 if the minor allele threshold for heterozygosity was manually adjusted) (Additional file 2: Table S9). The NextGENe analysis called 12/15 SNPs (Additional file 2: Table S10). For the Chinese individual (Additional file 2: Table S1 and Table S11), the Bioscope genotype concordance rate was near 100% for the 4-plex samples and the control sample, but the Bioscope SNP overlap was significantly lower than for the Yoruban samples. This, we assume, was partially due to the considerable false positive rate in the HapMap 3 data. Finally, we analyzed the Yoruban samples to distinguish potential novel SNPs from false positives or sequencing errors. We eliminated about 15-20% of SNPs as false positives using SNP-backmapping (see Additional file 1 and Additional file 2: Table S2). Then we eliminated about 20-30% of SNPs in the 4-plexes as unconfirmed using inter-sample concordance checks between technical replicates (see Additional file 1 and Additional file 2: Table S12). For one sample and one bioinformatics strategy (4-plex1bc05, SAET read enhancement, Bioscope TR mapping, Bioscope SNP-calling), we manually inspected the remaining SNPs to assess more accurately the false positive SNP calling rate after our filtering process: Bioscope called 75 SNPs, of which we removed 13 SNPs (17%) by backmapping the SNP and its flanking 25 reference bases to the WG. We then eliminated 22 SNPs (29%) after inter-sample validation within the same multiplex, considering concordant genotypes shared by several samples valid and rejecting the rest as potential false positives. We eliminated 34 SNPs (45%) that were known in dbSNP130. We eliminated one further SNP (1.3%) that was known in our ‘silver’ consensus (see Methods for the definition and details). We inspected the remaining 11 SNPs (15%) in the IGV viewer (Additional file 2: Table S13a), leaving 5 SNPs (8%) as potential novel SNPs and estimating the upper bound for false positives called by Bioscope at 6 SNPs (9%), and the upper bound for false positives after manual inspection at 5 SNPs (8%). We repeated this procedure for the non-SAET reads that resulted in 15 potential novel SNPs (Additional file 2: Table S13b). Finally, we identified the most likely potential novel SNPs (Additional file 2: Table S13c), i.e. those that were present in the SAET-reads and the raw reads, and subjected these to Sanger sequencing. As controls, we also selected some unlikely novel SNPs and a known SNP for sequencing (Additional file 2: Table S13c). We could not validate the potential novel SNPs.

Figure 3 (and Additional file 2: Tables S2, Table S3 and Table S5) summarizes our different bioinformatics strategies and SNP-calling results for the same 26 indexed Yoruban samples and the non-barcoded Yoruban control. We explored whether TR mapping increases SNP-calling sensitivity compared to WG mapping, expecting a higher rate of false positive SNPs for TR mapping as a trade-off. The first four groups, in Figure 3, show automatic SNP-calling results using Bioscope without any manual inspection. The automatic detection of known SNPs (our internal ‘silver’ standard) worked best for the non-barcoded control and the 4-plex. There was very little difference in sensitivity between TR and WG mapping. However, SAET read-enhancement decreased the Bioscope detection rate for known SNPs slightly, for example from 48/66 SNPs to 44/66 SNPs in the 4-plex using TR mapping. This was surprising because SAET led to an increase in the mapping coverage. After TR mapping (first two groups in Figure 3), SNP-backmapping, and manual inter-sample genotype concordance computation, we obtained 3–15 potential novel SNPs (for instance, 15 candidate SNPs for the SAET-enhanced reads in the 4-plexes). For WG mapping (third and fourth group in Figure 3), we only obtained one potential novel SNP at most. Groups number five and six in Figure 3 show results from the mapping with Bioscope and the SNP-calling with SAMtools mpileup, for genotype quality thresholds of 0, 9, 19 and 29. The sensitivity of SAMtools‘SNP-calling is slightly inferior to that of Bioscope. SAMtools also called more false positives than Bioscope. Bioscope mapping and pibase genotyping (seventh group) clearly yielded the best results. A subjective manual inspection in IGV (eighth and ninth groups) revealed evidence or at least some traces of all known SNPs in the Yoruban control (original reads mapped to TR) and the 4 plex1bc05 (original reads and SAET-enhanced reads mapped to TR). But it also yielded more discordant SNPs than pibase.

### Fourth stage: read-backmapping approach for public exome data

**Figure 4:**
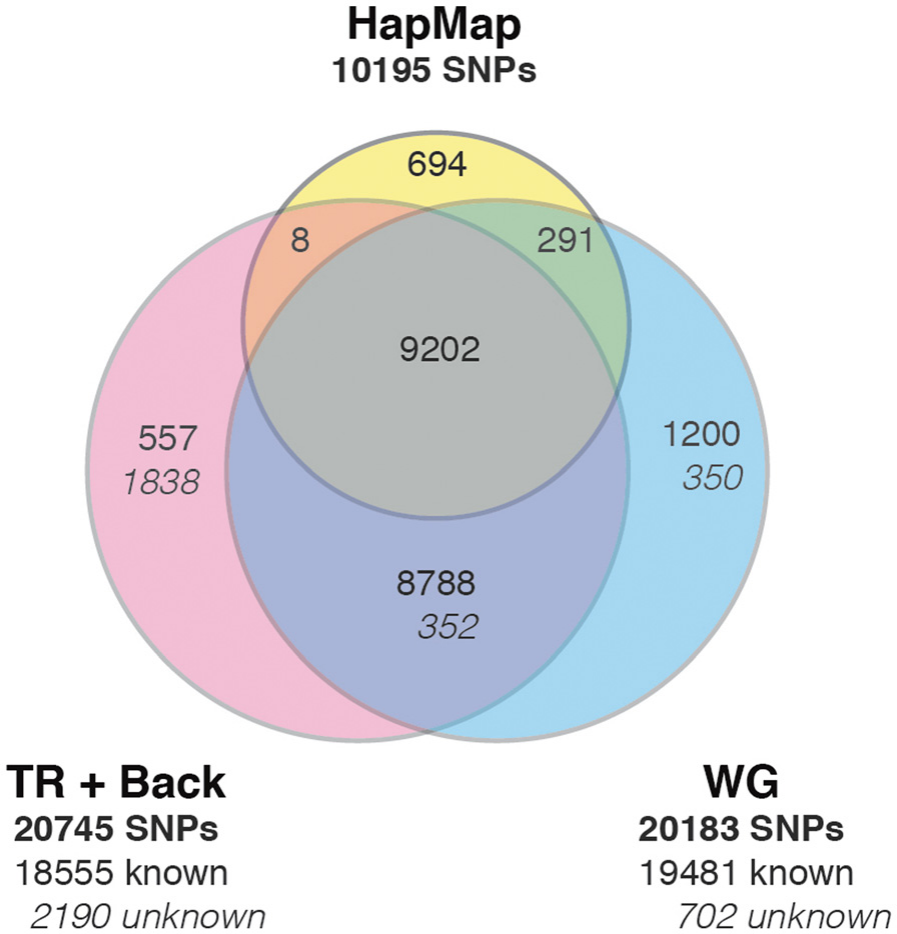
** Venn-Diagram of target-region-mapping SNPs, whole-genome-mapping SNPs, and HapMap chip data SNPs (fourth stage, exome data).** This Venn-diagram shows the total numbers of known SNPs and unknown SNPs (italics) in the exome TR from target-region mapping with SNP-cleanup by read-backmapping to the whole genome (TR + Back), whole-genome mapping (WG), and HapMap SNP-chip data from HapMap individual NA12878 (HapMap). The figure implies that the detection rate for unknown SNPs was increased three-fold by combining both mapping approaches (whole genome and target-region mapping).

In the fourth stage we applied our combined mapping approach (TR mapping and read-backmapping to WG) to two human exome Illumina HiSeq2000 data sets: a female CEU HapMap individual and her father (Additional file 2: Table S14). The entire process (mapping to the TR, read-filtering, initial SNP-calling, read-extraction, read-backmapping to the WG, and final SNP-calling) was two-fold faster, required only 10 hours (instead of ~22 hours) of computational time per exome on an eight-core linux compute node (Additional file 2: Table S15). At the initial SNP-calling stage, SAMtools called 71,488 variants for the daughter (Additional file 2: Table S16) of which 16,518 were homozygous, and 77,055 for the father, of which 17,052 were homozygous. After read-backmapping to the WG and final SNP-calling, 38,602 variants remained for the daughter and 38,763 for the father. This means that ~50% of the originally called variants were eliminated as likely false positives resulting from force-mapped reads (Additional file 3: Figure S4). It should be noted that some of the eliminated variants could be true variants in homologous regions of the genome. After filtering away the variants outside the exome, 20,745 remained for the daughter and 20,901 for the father. We estimated the false negative rate of SAMtools SNP-calling in the target region to be 9% (Additional file 2: Table S16), by computing the overlap with known SNPs in HapMap chip data (file: hapmap3_r1_b36_fwd.CEU.qc.poly.recode.map/ped at [20]). By comparison, we also performed mapping to the WG, SNP-calling, and filtering of non-exome variants, which required 22 hours of computational time. This resulted in only 20,183 SNPs for the daughter and 19,912 for the father, and showed that our approach has the potential to ‘rescue’ 3%-5% of valid SNPs which may not be detected using WG mapping. The SAMtools false negative rate in WG was only 6.5%. The SNP overlap between our approach and the conventional approach was only 92% (Figure 4), reflecting the problem of SAMtools not detecting all true SNPs, which we already mentioned in the *BRCA1/2* experiment (Figure 3). It should be noted that we used all SNPs without filtering away low-quality SNPs, to reduce the number of false negatives. The concordance between TR/backmapping and WG is 99% when including these unfiltered SNPs, showing that the backmapping method is applicable. Our results also suggest that SAMtools false negatives can only be reduced by using the TR/backmapping method as well as the WG method. 

## Discussion

We here present a novel and more specific ‘two-stage’ mapping/SNP-calling approach, which can speed up the analysis of a human exome sample by 12 hours on an 8-core compute node, and which can be applied to any targeted enrichment sample/region. We evolved this approach when we analyzed targeted enrichment runs and found that conventional mapping and SNP-calling takes up a wasteful amount of time and computing resources.

In our targeted enrichment experiments, we first evaluated a scalable multiplexed protocol for high coverage *t*NGS to investigate cost saving and quality control potential. We subjected barcoded (indexed) SOLiD libraries instead of gDNA to one selected microarray-based sequence capture method. We tested this pre-enrichment sample multiplex approach by sequencing 68 *E. coli* genes as well as two human cancer genes (*BRCA1* and *BRCA2*) in three independent stages and different multiplexing folds on the SOLiD system. We achieved good and reproducible coverage profiles for the TRs across most of the different multiplexed samples, enriching human exonic as well as intronic regions with less than 10% strand bias (Additional file 2: Table S17). Nevertheless, our enrichment design successfully captured only 54% of human *BRCA1/2* regions. This weakness of the tested enrichment design would also apply to other hybridization-based sequence capture bait designs that subject targets to repeat masking before probe design. For this reason, longer capture baits and iterative refinement of the bait design would be required for such genomic regions with low complexity. To evaluate the SNP discovery performance we then analyzed our sequencing data employing different mapping and analytical tools, which have rapidly evolved within the last two years (see Figure 2). We found that SNP detection in enriched regions - even at high coverages - depends strongly on the tools and their settings. These SNP-callers, including the widely used SAMtools [16], do not seem to be well-trained to handle enrichment data, and thus produced a significant fraction of false positive and negative SNP calls (Figure 3). To partially overcome this common mapping/SNP calling problem we proposed to combine the advantages of WG mapping (lower false positive SNP detection rate) with TR mapping (faster processing with higher SNP detection rate) (Figure 3) and developed our novel ‘two-step’ mapping approach. In this approach we first mapped raw reads to the TR to achieve faster mapping and SNP-calling, due to the smaller reference sequence, and to detect more SNPs, due to mapping more reads into low-complexity regions and obtaining higher coverages. We followed the first step with a SNP-cleanup mapping step to reduce false positives: In this step, only the SNP-supporting reads and their paired mates are mapped back to the WG (Additional file 3: Figure S4). Our novel approach resulted in more valid SNPs being detected and a more than two-fold speed-up of the time-consuming exome analysis.

Inadequate enrichment and/or coverage can prevent the detection of real nucleotide variants, leading to higher false negative rates, particularly for heterozygotes [21,22]. In general, 20-fold coverages are deemed necessary for reliable sequence variation calling in data from the 454 platform [23], Illumina Genome Analyzer [24], and SOLiD [24-26]. Other studies recommend a sequence coverage higher than 30× to minimize the risk of failing to detect true SNPs [27], and at least 33× to enable the correct genotyping of most of the heterozygous positions [21]. These high coverage thresholds are backed by a simulation of the SNP detection performance at the *NOD2* gene, which is associated with Crohn’s disease; it fell rapidly when the achieved coverage was below 40× [28]. But a high coverage is not the only prerequisite for accurate detection of sequence variation [6]. Rather, SNP detection seems to be significantly affected by the chosen alignment tools and SNP callers, as revealed from the results of our analyses (discussed further below). This is also in agreement with the results of a comparative analysis [29] of different alignment tools, which showed that there was a disturbingly low level of agreement between genome alignments produced by different tools. It concluded that it was not possible to make definitive qualitative statements concerning the alignment tools, as there are distinct trade-offs in their behaviours. Indeed, the 1000 Genomes Project Consortium [30] reported that their NGS genotyping accuracy at heterozygous sites was 95% and higher in some regions, dropping off to 70%-80% or lower in “harder to access regions of the genome”. To avoid false positives, the novel SNPs published by the consortium are the consensus of two or more independent groups, sequencing platforms, and pipelines.

Our experimental results (Figure 3) show that the enrichment and sequencing led to highly covered bases and the greatest number of SNP calls for the non-barcoded controls and the 4-plexes. They also illustrated that all SNP-calling tools performed weakly, with the exception of pibase and ‘manual’ inspection of aligned reads in IGV. Depending on the SNP-caller, WG mapping is generally characterized by a lower false positive and a slightly higher false negative SNP detection rate. The reverse holds true for TR mapping. Accordingly, combining both mapping approaches may help to rescue undetected true SNPs, and filter false positive SNP-calls. Regarding SNPs in non-repeat-masked regions, we feel confident that TR mapping with read-backmapping to the WG is an accurate and reliable method, because we discovered 4 genotyping errors in the HapMap data (21% of the non-reference genotypes) within our TRs. We supported this conclusion by Sanger sequencing (Additional file 3: Figure S5). When comparing our *BRCA1/2* SNPs with the HapMap3 SNP chip data, we detected up to 4.4× more known SNPs than have been published as HapMap chip data.

SNP-callers were originally optimised for low or moderate variations in coverage. In other words, extremely high coverages or high coverage gradients are currently challenging from the bioinformatics point of view. Even for non-enriched samples, some degree of automatic or manual postprocessing is required specifically to distinguish SNPs from misalignments [30]. A previous study [23] confirms our findings and emphasizes that manual inspection is an essential part of the analysis. As large-scale manual inspection is unfeasible and prone to subjective errors, we use the pibase software [17], manuscript under review) for interrogating BAM files [16], re-typing SNPs, and other analysis tasks. As shown in our *BRCA1/2* and exome analyses, the re-typing of SNPs in targeted enrichment experiments is cbuindispensible (for example, using pibase). We further recommend, if cost allows, to duplicate samples (which is easily performed in a multiplexed experiment) and validate computed SNPs by inter-sample concordance checks between these technical replicates.

The bioinformatic processing of eukaryotic and metagenomic sample data sets can occupy a compute cluster for days to weeks. For multiplexed targeted enrichment of human samples (1536 samples can be sequenced in a single SOLiD run), the genome-sized data traffic may overload the compute cluster when too many samples are processed in parallel. Compared to BioScope, other pipelines such as BWA alleviate this problem, but a complete exome run using BWA, SAMtools and Picard nevertheless takes about a full day. Our aim was to cut the computational time significantly by mapping to the small TR (100 kb of *BRCA1/2* regions, or 61 MB of exome regions, compared to 3 Gb of human genome) and cleaning up force-mapped reads. For exomes, we cut run time by more than half, enabling overnight runs on an 8-core node, or turnaround within a working day (4–5 hours) on a 16-core node. This run time cut is necessary to match the speed of new high-throughput platforms such as the Illumina HiSeq2500, which takes 27 hours for producing 120 Gb (see Additional file 2: Table S18 for details on potential energy saving calculations).

## Conclusions

We successfully demonstrated our novel time-saving ‘two-step’ mapping approach using Illumina HiSeq2000 human exome data from the 1000 Genomes Project. This approach consists of TR mapping with subsequent SNP-cleanup by read-backmapping to the WG. We developed this approach after designing targeted enrichment experiments and experiencing an odyssey of run-time and SNP-detection problems when we used a wide range of mapping and SNP-calling tools. We recommend our approach and the employed tools (BWA, SAMtools, Picard, VCFtools, pibase) for a reliable and efficient analysis of exome and targeted enrichment data. To attain confident results, we specifically recommend the SNP-validation between duplicated samples and a final *in silico* validation using recently developed software and/or manual inspection.

## Methods

The study design for our *tNGS* test model is shown in Figure 1. In our test model, we began the procedure by constructing the SOLiD sequencing fragment libraries (with and without barcodes) and pooling the desired libraries. Then, for each individual sample or pool we selected and enriched the TRs using the HybSelect sequence capture technology ([31-33]; Febit biomed GmbH, Heidelberg, Germany). This included three main steps: hybridization of the genomic DNA library to a Geniom biochip containing target-specific DNA capture probes, washing off non-captured DNA fragments, and elution of the captured fragments. Then, we resequenced the enriched products using the SOLiD 3.0 system platform (Life Technologies, Foster City, CA, USA). Details for each step (first, second, and third experimental stages) are fully described in the Additional file 1. Based on these experiments, we finally developed a novel ‘two-stage’ mapping/SNP-calling approach, which we demonstrated to work on human exome data from the 1000 Genomes Project.

### SOLiD sequence analysis (stages 1–3)

We performed read mapping and SNP calling with SOLiD Corona Lite and later switched to Bioscope (a pre-release version of v1.2), after the manufacturer advised that the Corona Lite SNP-caller (pre-release diBayes) was not designed for enriched samples (because of high coverages, steep coverage gradients and/or PCR duplicates). Our Bioscope mapping and SNP-calling settings (Bioscope plan and ini files) are fully documented on the project homepage [19]. For SNP-calling we followed the manufacturer’s recommendations for enriched samples and set ‘call.stringency = high_coverage’ and ‘coverage.iqr.het.high = 10000’. These settings tell the SNP-caller that the coverage is ‘high’, that SNPs must be supported by reads on both strands, and that very-high-coverage SNPs are not artifacts. The filtering of base qualities and reads (low quality reads, duplicate reads) is carried out internally by the Bioscope SNP-caller using its own methods, and is reported in a ‘consensus call output file’.

### SOLiD reference sequences

We mapped the 50-mer reads using two separate mapping strategies for each sample: WG mapping (i.e. the normal procedure) and TR mapping (i.e. the computationally faster procedure). For *E. coli* WG mapping we used the *E. coli* K-12 M1655 genome (GenBank U00096; Refseq NC_000913) as our mapping reference. For human WG mapping we used the NCBI36/hg18 assembly as our mapping reference. For TR mapping we constructed a single FASTA reference sequence consisting of greater TRs, each separated by a block of 50 Ns (i.e. taking into account the read length of 50). Greater TRs consisted of the actual TRs, extended by 49 bases and merged with adjacent TRs if an overlap occurred. The reason for defining greater TRs was to map reads which partially overlap actual TRs. For *E. coli* we targeted 68 genes with a total length of 90 kb. For the human samples we targeted 198 small regions with a total length of 90 kb in two genes (a reduction of the size of the intended TRs from 165 kb to 90 kb because of the repeat-masker to enable designing efficient capture probes).

### SOLiD coverage and technical reproducibility analyses

We wrote R-scripts [34] for the coverage analyses and plots, using the mapping statistics files and the base-wise coverage files generated by Corona Lite and Bioscope. Our coverage metrics included percentage on-target reads (specificity), average depth of coverage (ADoC), covered TR bases (completeness), percentage TR bases at various coverage depths (2×, 5×, 15×, etc.), on-target versus off-target coverages (AUC), and enrichment factor (EF, see Additional file 1, formula (1)). For visualizing the coverage uniformity between all barcoded samples of the same plex and comparing with non-barcoded samples, we generated a zoomed-in multi-sample coverage plot for each TR (see Additional file 3: Figure S6). To analyze the technical reproducibility of coverages between samples we created a scatter plot and computed Pearson’s coefficient of correlation for pairs of samples, and summarized these coefficients for all samples in the form of a correlation matrix (Additional file 3: Figure S7).

### Consensus SNP list for SNP-calling benchmark in the SOLiD *BRCA1/2* experiments

As validated references for our own SNP-calling, we created consensus SNP lists for the Yoruban HapMap individual NA18507. We created our ‘gold’ consensus from the HapMap3 data ([20]), WG Illumina NGS data [21] (file: pgYh1.txt.gz at [35]), WG SOLiD NGS data (file: Yoruban_snp_18x.gff at [36]), and by Sanger resequencing, resulting in 15 non-reference genotypes in the TR (Additional file 2: Table S19). This eliminated 4 of 19 HapMap SNPs (21%), which is broadly in line with the HapMap3 website (28 June 2012) estimate of a 12%-14% false positive rate. We created our ‘silver’ consensus of 66 non-reference genotypes from the WG Illumina data and the WG SOLiD data. For the Beijing Han Chinese HapMap individual NA1856 our benchmark SNP list was the HapMap3 SNP list with 28 non-reference genotypes (Additional file 2: Table S19), due to the unavailability of ‘highly’ covered WG data (see Results/Discussion sections in the Additional file 1).

### SNP cleanup by ‘read-backmapping’

To clean up SNPs obtained after our fast TR mapping approach, we initially tested a ‘SNP-backmapping’ approach (see Results and Additional file 1). This did not eliminate all potential false positive SNPs (Figure 3). We then developed our ‘read-backmapping’ approach to eliminate all force-mapped reads: First, only those reads (and, if present, their paired mates) that cover SNPs are mapped to the WG, which identifies reads that map better or equally well to a different locus. Then, each SNP is re-typed using only those reads that remain mapped uniquely over the SNP. The read-backmapping method corresponds exactly to the WG mapping, except that only the SNP-covering reads (and their paired mates, if appropriate) are mapped (and not all initial reads). To implement this method, we wrote Python scripts [19] and utilized existing freely available standard tools. We based our implementation on the current standard alignment format BAM [16] and the current standard genomic region format BED [37]. Our implementation is not applicable to SOLiD Corona Lite, SOLiD Bioscope v1.0.1 or pre-release v1.2, as these pipelines do not generate BAM files. As a note, BioScope and its successor LifeScope are not freely available. Therefore, we here show results for freely available data and pipelines from the 1000 Genomes Project [30].

### Stage four: illumina data from the 1000 genomes project

To evaluate the performance of our backmapping pipeline we used publicly available sequence data of a HapMap CEU family trio (ID 1463) who had been repeatedly sequenced within the 1000 Genomes Project [30]. As representative exome examples, we chose two runs submitted by the Broad Institute ([38]) where the genomic DNA of the father and his daughter had been prepared as paired-end exome libraries (Hybrid Selection) and sequenced on an Illumina HiSeq 2000. To test our approach on genomic data, we selected a third run submitted by the Broad Institute, where a paired-end WG library of the mother’s gDNA had been sequenced on an Illumina Genome Analyzer II. The data are publicly available from the sequence read archive [39]: female NA12878 (SRR098401), her father NA12891 (SRR098359), and her mother NA12892 (SRR032860). More details on the Illumina runs are given in Additional file 2: Table S14.

### Conventional mapping and SNP-calling (whole genome reference)

We aligned the reads to the human genome reference NCBI GRCh37 [40] using BWA [41], converted its SAM output to BAM format with SAMtools [16]. Then we removed duplicates with Picard [42]. Finally, we called variants using SAMtools with the option “mpileup –E” for higher sensitivity but lower specificity [43].

### Mapping and SNP-calling (exome reference)

The exome-based reference sequence for the TR mapping was created as follows: We downloaded a file of exonic regions, represented by the compatible Consensus Coding Sequence (CCDS) file ‘CCDS.20110907.txt’ at [44]. We then converted the CCDS file into BED-format [37] with a self-written Python script, resulting in 287,287 lines – chromosome, start, and stop for each exon. Next, we used another self-written Python script to extract the reference bases for each BED region from the WG reference GRCh37. The script included a padding of ± 50 bases to account for reads that only partly originated from an exon. The script also merged the overlapping regions, resulting in 183,410 sequences, and concatenated the sequences into a single sequence using 50 Ns as a buffer between sequence regions. The same script generated a text file with the coordinate transformation table between the regions of interest in the TR coordinates and the original WG coordinates. Finally, we mapped the reads of the three individuals against this new reference and called SNPs in the TR, using the conventional mapping and SNP-calling pipeline described above for the WG reference.

### Backmapping of SNP-supporting reads

We uniquely extracted reads covering heterozygous SNPs, from the BAM-file. As the reads had been sequenced from paired-end libraries, we also extracted the according mate from the BAM-file and created new FASTQ files containing these reads and their (possibly unmapped) mates. We implemented the read-extraction process in a self-written shell script which used VCF-Tools [45], SAMtools, and Picard. Finally, we aligned the extracted reads to the WG reference GRCh37, converted formats, and removed duplicate reads using the steps and programs previously described.

### Final SNP typing

Using a self-written Python script and the coordinate transformation file (see above), we transformed the coordinates in the SNP-list from the exome reference coordinate system to the genomic coordinate system. We divided the SNP-list into heterozygous SNPs and homozygous SNPs. Because the heterozygous SNPs can be artifacts resulting from the alignment of non-exonic reads to the exome reference, we extracted the corrected genotype at the SNP-coordinates from the backmapped WG BAM file using the pibase software.

## Competing interests

None of the co-authors provided competing of interests.

## Authors’ contributions

AE, AF designed the study; NS, AK, PFS performed the sample enrichment; AE designed the next-generation sequencing experiments; MF, AK, IT, B-SP, BS, NS performed data analysis; IT, B-SP, MF created the tNGS Backmapping pipeline; AE, MF interpreted the data and wrote the manuscript; NS, PR, IT, SS, AF proof-read and edited the manuscript; AE, MF, IT, B-SP, PR, AF revised the manuscript; AE, PFS, PR, AF coordinated the project. All authors read and approved the final manuscript.

## Supplementary Material

Additional file 1 ** Supporting Methods and Results.** It includes additional details of the Methods, Results and Discussion. - It lists all the Supplementary Tables and Figures (shown in Additional file 2 and Additional file 3, respectively).- It also includes Tables A1-A3 and Figures A1-A5. Click here for file

Additional file 2 Supporting Tables. Tables S1-S26. Click here for file

Additional file 3 Supporting Figures. Figures S1-S9. Click here for file
