# Supplementary material for: Improving mapping and SNP-calling performance in multiplexed targeted next-generation sequencing
Source: BMC Genomics. 2012 Aug 22;13:417. doi: 10.1186/1471-2164-13-417 (PMC3563481; doi:10.1186/1471-2164-13-417)
Supplement: Additional file 1 — Supporting Methods and Results. It includes additional details of the Methods, Results and Discussion. - It lists all the Supplementary Tables and Figures (shown in Additional file 2 and Additional file 3, respectively).- It also includes Tables A1-A3 and Figures A1-A5. [file 1471-2164-13-417-S1.pdf]

Supplemental Note

(Including Figures A1-A4, Tables A1-A3 and lists of Supplementary Tables and Figures)

Improving mapping and SNP-calling performance in multiplexed targeted next-generation sequencing

Abdou ElSharawy[1,5], Michael Forster[1, 5], Nadine Schracke[2], Andreas Keller [3], Ingo Thomsen[1], Britt-Sabina Petersen[1], Björn Stade[1], Peer Stähler[2], Stefan Schreiber[1,4], Philip Rosenstiel[1] and Andre Franke[1]

1 Institute of Clinical Molecular Biology, Christian-Albrechts-University, Kiel, Germany

2 Febit biomed GmbH, Heidelberg, Germany.

3 Biomarker Discovery Center, Heidelberg, Germany

4 Department of General Internal Medicine, Campus Kiel, University Hospital S.-H., Germany

5 These authors contributed equally to this work

Email addresses

AE: a.sharawy@mucosa.de

MF: m.forster@ikmb.uni-kiel.de

NS: nadine@schracke.de

AK: keller.andreas@siemens.com

BS: b.stade@ikmb.uni-kiel.de

B-SP: b.petersen@ikmb.uni-kiel.de

IT: i.thomsen@ikmb.uni-kiel.de

PFS: peer.staehler@t-online.de

SS: s.schreiber@mucosa.de

PR: p.rosenstiel@mucosa.de

AF: a.franke@mucosa.de

METHODS

First stage – establishing a tNGS test model: test of the compatibility of microarray sequence capture technology with SOLiD NGS

In the first experiment, to generate our test targeted next-generation sequencing (*t*NGS) model, we tested the compatibility of HybSelect sequence capture with the SOLiD 50mer sequencing pipeline using two different ***Escherichia coli*** (*E. coli*) genomic SOLiD fragment libraries in 4× technical replicates. To start with a simple genomic model, we selected 68 genes (~2% of *E. coli* genome) from three different metabolic pathways as targets for resequencing. Instead of choosing the normal procedure (i.e. using gDNA for sequence capture) we started our procedure with the construction of the SOLiD sequencing library. For details, see the SOLiD sequencing library section below. To assess the efficiency, uniformity of coverage, and reproducibility, we performed four sequence captures for each library type ('express' vs. 'standard' fragment libraries: 4× technical replicates each; a total of eight samples). After eluting the captured DNA off the DNA microarray, we made the 8 samples double-stranded using few cycles of PCR and then quantified and used them directly for the emulsion PCR (emPCR) as described in the manufacturer's protocol (Applied Biosystems SOLiD 3.0 System; Templated Bead Preparation Guide, Part Number 4407421 Rev. B 02/2009). After bead enrichment, we resequenced the eight samples, which were physically separated on an octet sequencing slide.

Second stage – test of pre-enrichment sample multiplexing in the established tNGS workflow

In the second round of experiments, we constructed five *E. coli* genomic SOLiD 50mer 'standard' fragment libraries and indexed these with barcodes (BCs) 1-4, 5-8, 9-12, 13-16, and 17-20, respectively (SOLiD barcode series B-20, supplied by Ambion, Austin, TX, USA). We generated three pools of libraries for sequence capture and downstream resequencing. We pooled BCs 1-4 (pool 1; 4-plex), BCs 1-8 (pool 2; 8-plex), and BCs 1-20 (pool 3; 20-plex). For each pool, we added equimolar amounts of the respective libraries into one single tube for processing using the protocol established in the first stage. After target enrichment and resequencing, we analyzed the data as pooled libraries as well as individual samples.

Third stage – test of pre-enrichment multiplexed tNGS on human cancer genes (BRCA1/2)

In the third stage, we generated 12 human genomic SOLiD 50mer fragment standard libraries from two HapMap samples, namely NA18507 Yoruban from Ibadan, Nigeria and NA18561 Chinese from Beijing. We selected these two HapMap samples for our experiment, because their genotype data and whole genome NGS data were already publicly available for comparison (see Results; YRI; sample

NA18507; [1]; CHB; sample NA18561; ftp://ftp.1000genomes.ebi.ac.uk/vol1/ftp/pilot_data/data/NA18561/alignment/). We prepared six libraries using HapMap NA18507 gDNA: one 'standard' fragment library without barcoding (control 1) and five libraries indexed with BCs 1-2, 5-6, 9-10, 13-14, and 17-18, respectively. We prepared the other six libraries using HapMap NA18561 gDNA: one 'standard' fragment library without barcoding (control 2) and five libraries indexed with BCs 3-4, 7-8, 11-12, 15-16, and 19-20, respectively. We included the two non-barcoded gDNA libraries as controls for the multiplexing performance, because the human genome is far more complex than that of *E. coli*. We collected the barcoded samples into 5 pools as follows, with each pool containing NA18507 and NA18561: BCs 5-6 and 7-8 (pool 1; 4 BCs); BCs 7-8 and 9-10 (pool 2; another 4 BC combination); BCs 1-2, 3-4, 5-6 and 7-8 (pool 3; 8 BCs); BCs 1-2, 3-4, 5-6, 7-8, 9-10, 11-12, 13-14 and 19-20 (pool 4; 16 BCs); BCs 1-2, 3-4, 5-6, 7-8, 9-10, 11-12, 13-14, 15-16, 17-18 and 19-20 (pool 5; 20 BCs). For each pool, we added equimolar amounts of the respective libraries into one single tube for processing. During sequence capture and resequencing we processed the two non-barcoded controls and the five indexed pools in parallel, physically separated into seven different microfluidic 'arrays' or slide 'spots'.

SOLiD sequencing library construction

For each fragment sequence library preparation we used five to ten micrograms of either *E. coli* K12 MG1655 gDNA (LGC Standards, Wesel, Germany) or HapMap gDNA samples (NA18507 and NA18561; Coriell Repositories, USA). In the first stage of experiments (**Figure 1c**) we prepared two different fragment sequencing libraries, namely 'express' and 'standard' fragment libraries, according to the standard SOLiD 3.0 system protocols (Applied Biosystems, CA, USA). Since 'standard' fragment libraries showed better enrichment performance in the first stage, we constructed all libraries in the second and third stage using the 'standard' fragment protocol. For the pooled barcoded samples (second and third stage of experiments) an important stage of the protocol was to pipette equimolar amounts of each library based on their final concentrations after library preparation.

Targeted genomic sequences

We preferred to start the protocol optimization with a simple genomic model. We first targeted a total of 90 kb of genomic sequences for enrichment, consisting of 68 genes of three metabolic pathways (glycolysis, fatty acid synthesis, and β-oxidation) distributed across the *E. coli* genome (~2% of the *E. coli* K-12 MG1655 genome; see ***E coli.* Supplementary Table ET4** on the project homepage). These genes were: *aceE-2, acn, acn, pps, fadE, sucA, fadB, fadJ, edd, glcB, sdhA, maoC, accC, aceB, gpmM, pykA, fadI, fadD, zwf, pgi, aidB, fabF, aceA, pykF, fumC, eno, icd, gltA, fadK, mqo, sucB, prpC, fadA, fabB, fbaB, fbp, fabD, ydiO, pgk, aceF, sucC, fbaA, accD, glpX, tpiA, sucD, ybhA, sdhB,*

accA, eda, fabA, pfkA, fabI, ytjC, mdh, fumA, gapA, pgI-2, fabH, pfkB, gpmA, fumB, accB, fabZ, sdhC, fabG, sdhD, and *lpd*. For the human genome experiments, we aimed to target a total of 165 kb of genomic sequences for enrichment. These constituted the two human cancer genes *BRCA1* at chromosome 17 and *BRCA2* at chromosome 13 with 81 and 84 kb size, respectively. We selected these two genes because of their well-known role in the development of breast cancers and clinical implications across race and ethnicity [2-4]. In addition, the *BRCA1/2* Geniom Biochip had been successfully tested in another independent experiment using Illumina technology [5].

Biochip design and synthesis

We performed *in-situ* oligonucleotide synthesis using a digital micromirror device (Texas Instruments, Dallas, TX, USA) for light-directed activation on an activated microfluidic array consisting of a glass/silicon-glass sandwich within the Geniom instrument (febit biomed GmbH, Heidelberg, Germany) [5]. Each chip consisted of eight arrays, thus allowing 8-fold multiplexing, with 15,624 individual DNA oligonucleotide features. This resulted in a total content of ~125,000 features. For enriching the 68 *E. coli* genes, we used one array as the enrichment matrix for each experiment. Fifty-mer probes were distributed over the coding sequences with a six base pair tiling density. These probes aimed to fish both the sense and anti-sense strand in an alternating manner.

A substantial fraction of the human genome comprises repetitive and transposable elements and >5% of which is duplicated [6] that would interfere with HybSelect probe design and specificity on one hand and complicate the assembly/alignment of the retrieved sequences on the other. The targets were therefore subjected to repeat masking before probe design to avoid capture of homologous repetitive elements (low complexity regions). This strategy did not reduce the region of interest (ROI) of 90 kb of *E. coli* as all prokaryotic genes consist of non-repetitive elements and hence are of high complexity. In contrast, the 165 Kb of human cancer genes were reduced to a core of 89,568 bp (54%), corresponding to a capacity of > 0.7 Mb ROI or > 1.3 Mb HR per biochip.

HybSelect sequence capture of target regions

We carried out the HybSelect sequence capture according to the standard protocol [5]. Briefly the protocol includes the following steps:

DNA sample preparation for HybSelect. Adaptor-ligated gDNA library (1500 ng, 750 ng or 2.14 ng; sample 4 with 1500 ng blocking DNA) and 400 µg tRNA / sample (except samples 5-7) were dried in a Speed-Vac and dissolved in febit Hybmix-5, heated to 95°C for 5 min and placed on ice. The sample mixture was injected automatically onto the biochip in the Geniom RT Analyzer. Hybridization was performed for 16 hours at 42°C with movement (agitation) of the sample. After

hybridization, each array was washed twice with 6× SSPE at room temperature and 0.5× SSPE at 45°C. Each array was subsequently washed with 2 ml each of SSPE-based febit stringent wash buffers 1 and 2 at room temperature. Hybridization and all washing steps were carried out fully automated by the Geniom RT Analyzer. For elution of the enriched samples, the biochip was inserted into a HybSelect Elution Holder, and the arrays were filled with 15 µl elution reagent each and incubated at 70°C for 30 min. The solutions were then injected into separate plastic PCR tubes, placed in a Speed-Vac and dried.

Enzymatic treatment of eluted DNA. Upon drying, the DNA was dissolved in 23 µl H_2O, 25 µl Phusion HF DNA Pol 2× MasterMix (Finnzymes, Espoo, Finland) and 1 µl 10 µM primer. Then each of the SOLiD PCR primers used for PCR within the library-preparation protocol was added. The samples were incubated in a PCR cycler using the following protocol.

1×	10×	1×
30 sec 98°C	10 sec 98°C	5 min 72°C
	30 sec 62°C	hold 4°C
	30 sec 72°C	

The DNA was purified using the MinElute PCR Purification Kit (Qiagen, Hilden, Germany). Elution was carried out using 20 µl of kit-supplied EB-buffer.

HybSelect of human samples. For the eukaryotic samples, the HybSelect procedure was carried out twice. After elution from the biochip, the enriched libraries were dried according to the protocol, and 12 cycles instead of 10 were employed in the enzymatic treatment step. The second cycle was then performed exactly as described in the protocol followed by 10 cycles of PCR.

Quantification of the eluted products was carried out using the fluorospectrometer NanoDrop ND-3300 (Peqlab, Erlangen, Germany) in combination with the Quant-iT™ PicoGreen dsDNA Reagent and Kits (Invitrogen, Carlsbad, CA, USA) according to the manufacturers' instructions.

NGS using SOLiD™ 3.0 system platform

The principle steps of the SOLiD sequencing protocol are well covered [7-10]. In brief, the short fragment DNA libraries first underwent titration emulsion PCR (emPCR) followed by sequencing to determine the optimal template concentration. After this, the libraries were clonally amplified on beads using large-scale emPCR. The emPCRs were then broken and washed to purify the emulsified beads, enriched for template beads, and 3'-end modified to allow the beads to attach to the sequencing slide. Each enriched product (single or pooled libraries) was run on a single octant of an octet slide.

The SOLiD sequencer can process two slides in parallel, independently of each other. Slides are available with 1, 2, 4 or 8 wells (called spots). The method uses two-base-encoded probes, i.e. 2 bases are interrogated at a time using color-coded “universal 1,2-primers" which bind to 5 bases, of which the first 2 bases are specific and the last 3 are generic. Each base is interrogated by 2 separate universal probe hybridizations (i.e. 2 color calls) which has the advantage of improved base calling accuracy (invalid color call sequences point to sequencing errors). This ‘color space’ concept is a unique feature of the SOLiD system. A primer is hybridized to templates amplified by emulsion PCR (emPCR). The SOLiD cycle of 1,2-probe hybridization and ligation, imaging, and probe cleavage is repeated ten times to yield ten color calls spaced in five-base intervals. The extended primer is then stripped from the solid-phase-bound templates. A second ligation round is performed with an ‘n – 1’ primer, which resets the interrogation bases and the corresponding ten color calls one position to the left. Ten ligation cycles ensue, followed by three more rounds of ligation cycles. Color calls from the five ligation rounds are then ordered into a linear sequence (that is, the color space sequence or “read”). Color space reads are translated into (base space) FASTA code during alignment to a reference genome, which is performed off the machine, on a high performance computer cluster.

NGS data analysis on high performance computing clusters (HPCs)

When processing the large amounts of NGS data, run time is the most significant limiting factor leading to trade-offs between accuracy and speed. To successfully run lengthy alignment computations on inherently unreliable clusters (e.g. due to other users' errors which crash compute nodes, storage shortage, hardware failures, network file system time outs and failures), NGS cluster software breaks down the process into incremental stages which run between minutes and 1-2 days. The alignment process is broken down into (a) mapping, (b) SNP detection, and (c) indel detection.

Some software tools parallelize the mapping process by splitting the reference sequence into chromosomes, mapping all reads against one chromosome each per job, and identifying duplicate hits over all chromosomes (e.g. Corona Lite). Other software tools parallelize the mapping process by splitting the reads files and mapping against the entire reference (e.g. Bioscope). The second approach allows more balanced CPU usage at the cost of greater job concurrency, i.e. greater disk usage concurrency (competing disk input/output) and greater peak network loads (competing network traffic).

We experienced that extreme disk input/output (I/O) peaks on a single storage server node caused the server node to require re-booting (after a hard reset), and furthermore propagated network file system (nfs) problem to other nodes, which halted the entire cluster. Since this experience repeated itself, we now wait for some minutes between each job submission to the cluster job management system, and avoid too many I/O intensive jobs within the same cluster queue. We also found it necessary to ensure

that the software's disk requirements are not greater than the available resources on a given compute node – a frequent source of job failures on a multi-user cluster – for example by running jobs on entire nodes, as opposed to sharing nodes with other jobs.

We used a CentOS 5.3 linux cluster with 32 × 8-core AMD Shanghai nodes (32 MB RAM per node, 1.6 TB local scratch disk per node), which is managed by the HPC center of the Christian-Albrechts University of Kiel. We adapted Corona Lite to run under the PBS Pro job management system which was used by our HPC center instead of the Torque scheduler for which Corona Lite was originally written.

Mapping the SOLiD sequencing data

The SOLiD software maps reads in color space: For Corona Lite the FASTA reference sequence must be converted to color space in a separate step, whereas Bioscope accepts a normal FASTA reference sequence and converts this into color space itself.

Mapping in Corona Lite is BLAST-like (SOLiD 3 Bioinformatics Training manual, May 2009) and uses only the color space FASTA (csfasta) files: A seed sequence is mapped and then extended to the read length, allowing up to 5 mismatches (MM) in 50-color reads. The seed sequence is discontinuous, resulting in about 10× faster mapping than classic BLAST.

Mapping methods in CLC bio are unpublished and proprietary. The software takes into account both base space and color space, and makes use of both the csfasta and the quality value files. We mapped the *E. coli* samples and a single human sample using the fast ungapped mapping option with the default settings.

Mapping in Bioscope is performed using a seed-and-extend alignment method (BioScope™ Software for Scientists Guide, Data Analysis Methods and Interpretation, April 2010). By default the software uses 2 alternative seed “anchors” (segments of a read), extending the mapped seed in both directions, and finally selecting the best alignment based on a score. The user may specify more than the 2 anchors to further improve mapping. Bioscope’s method improves the probability of mapping at least a segment of a previously unmappable read (e.g. a segment up to and including an indel somewhere in the read, or a segment ending at a stretch of sequencing errors). Bioscope requires both the csfasta and the quality value files.

We observed that mapping results change from version to version. For example from Bioscope 1.0.1 (used for this manuscript) to Bioscope 1.2., our first test (using unpublished data and performing a linux "diff" on the first 5000 reads) showed that some reads were now uniquely mapped in v1.2, e.g.:

Bioscope 1.2: read with ID 1_20_1725_F3 matched uniquely on chromosome 5, reverse strand, position -42349950

<>1_**20_1725_F3**,5_-42349950.3:(40.3.0):q54

>>**1_20_1725_F3**,23_62939894.2:(40.4.0):q0,23_127235763.2:(26.2.0):q0, 23_115651593.2:(40.5.0):q0, ...

Bioscope 1.0.1: read with ID 1_20_1725_F3 matched in many locations (list truncated)

Or that reads matched in a different location in v1.2 than in v1.0.1, e.g.

Bioscope 1.2: read with ID 1_1102_875_F3 matched uniquely on chromosome 13, position 58984145

<>1_1102_875_F3,13_58984145.2:(24.2.15):q7

>>1_1102_875_F3,9_31114714.2:(24.2.0):q7

Bioscope 1.0.1: read with ID 1_1102_875_F3 matched uniquely on chromosome 9, position 31114714

Enhancing off-machine SOLiD sequencing data

For the human samples, we compared off-machine reads and SAET 2.2 (http://solidsoftwaretools.com/gf/project/saet/) software enhanced reads. SAET aims to improve mapping and SNP calling accuracy. The software uses the original csfasta and quality value files, and produces new files which can be used in exactly the same way as the original files. We used the default options, specifying a reference length of 118822 bases, i.e. the FASTA bases in the target region reference.

Generation of coverage files

Corona Lite generates coverage files during a mapping run, while Bioscope 1.0.1 does not (Bioscope 1.2, for instance, offers the option to generate these files), but these can be generated from the Bioscope mapping gff3 files using the script `gff3_to_coverage.pl` supplied by the

Bioscope developers. Users of the CLC bio Workbench software can generate coverage files from within the graphical user interface after right-clicking the coverage plot window.

Enrichment Factor

We used the following enrichment factor formula, which was recommended by the READNA consortium (READNA-symposium, 5-7 July 2009, Berlin, A. Brookes), where "count" denotes the number of reads:

$$(1) \qquad EF = \frac{\dfrac{count_{(on-target)}}{size_{(target)}}}{\dfrac{count_{(total)}}{size_{(genome)}}}$$

For Bioscope's progressive mapping of SAET-enhanced reads we observed that the EF depends on our choice of read cut-off length (**Table S22**). Readers who are interested in EFs should note that the SAET/Bioscope EFs are not comparable to EFs from older mapping tools. As an example we calculated two variants of the EF, firstly for all reads (read lengths 24-50) and, secondly, for only those reads of length 49 with 0 mismatches (including length 50 with 0 MM and 1 MM) which we use to confirm otherwise ambiguous SNP calls. The latter EF was nearly 2× higher, indicating that complete reads were mapped well to the target region and short segments were mapped well to the off-target regions of the genome.

AUC specificity/sensitivity metric

To measure the enrichment efficiency with regard to sensitivity and specificity we computed receiver operator characteristic curves (ROC) and calculated the area under the curve (AUC). The ROC curve shows the sensitivity as a function of specificity, with the area under the curve as a quality metric. The minimum and maximum values of AUC are 0 and 1. An AUC value of 1 indicates the best enrichment efficiency, meaning that all bases in the target region are covered while no single base outside the target region is covered. For a completely unspecific enrichment the AUC would be 0.5. For computing AUC, the area under a receiver operator curve (ROC), we calculate the specificity (2) and sensitivity (3) for 20 coverage thresholds of $t = 1, 10, 20,...200$, where true positives are the number of target region bases covered more than t times, and true negatives the number of off-target bases covered less than t times. False positives are the number of off-target bases covered more than t times, and false negatives are the on-target bases covered less than t times. We computed AUC by trapeze integration of the specificity over (1-sensitivity) curve.

$$(2) \qquad specificity = \frac{true\ negatives}{true\ negatives + false\ positives}$$

$$(3) \qquad sensitivity = \frac{true\ positives}{true\ positives + false\ negatives}$$

SNP-cleanup by 'SNP backmapping'

SNP backmapping addresses the issue of potential false positive SNPs arising from target region mapping. If reads from a different locus are mapped onto the target region reference sequence, it is likely that mapping-related artifacts will be called as SNPs. We first tried a 'SNP- backmapping' approach to report whether a test sequence (i.e. the SNP flanked upstream and downstream by half a read length of reference sequence) maps uniquely to the genome. Using BLAST [11], we mapped the test sequence to the whole genome allowing a maximum of 3 mismatches. The SNP was flagged if at least one hit not corresponding to the original position was detected. Flagged SNPs were interpreted as likely artifacts and removed from our SNP list.

Search for novel SNPs in the BRCA1/2 samples

To identify potential novel SNPs and minimize the number of false positives we used the following approach: We filtered the Bioscope list of SNPs to exclude SNPs outside the target region. We then filtered each SNP using our SNP backmapping method. We filtered the remaining SNPs (using dbSNP130 and our 'silver' standard SNP list) into a list of known SNPs and a list of potential novel SNPs. We validated potential novel SNPs *in silico* before proceeding to more time-consuming and expensive validation methods (e.g. manual reads inspection, laboratory resequencing): In a first inter-sample comparison we estimated an upper bound for false positive SNP calls by identifying potential novel SNPs in barcoded samples which were unconfirmed by the (higher covered) non-barcoded control sample (the results are given in **Table S20**). In a second inter-sample comparison we analyzed intra-plex concordance between technical replicates (of the same individual but with different barcodes) and kept only those potential novel SNPs which were shared by these technical replicates. In a third step, we inspected the potential novel SNP positions in IGV using both the target-region mapping file and whole genome mapping file, to identify potential artifacts arising from target-region mapping. We selected the most likely potential novel SNPs, some artifacts, and a known SNP, for validation by Sanger sequencing.

RESULTS / DISCUSSION

First stage – targeted resequencing of 68 E. coli genes: test of compatibility and optimization

The aim of the first stage of experiments was to generate and optimize our test *t*NGS model. The analysis of the sequencing runs is summarized in **Table A1**. We compared in this stage the performance of two different fragment SOLiD libraries, namely 'express' and 'standard' libraries. The difference between the two protocols is a gel size-selection step in the 'standard' library protocol. On average, for each of the 4× sample replicates, 16 million reads (800 Mb) were generated. There were no significant differences in the generated number of reads between each of the octants of the slide. For the four 'standard' library preparation samples, CLC bio Workbench 3.7.1 mapped 43.46% of these reads uniquely to the genome, and of these 31.76% to the target regions. For the four 'express' library preparation samples, 39.28% of reads were mappable to the genome, and of these 19.29% mapped to the target regions (**Table A1** and ***E. coli* supplement** on the project homepage).

Table A1 shows the average depth of coverage (ADoC) ranging from 540× to 1470× and the enrichment factor (**Figure A2 and formula (1)**) ranging from 8.6 to 20.34. The theoretically maximal EF for this experimental setup is 51.93. Moreover, to assess the reproducibility of the applied sample enrichment method we calculated the pair-wise Pearson correlation coefficient between samples (**Figure S7a and S7b**). The overall reproducibility within the eight experiments was high as demonstrated by Pearson correlation values of between 0.974 and 0.997 (**Figure S7b**). In the scatter plot (**Figure S7b**), each dot represents a position in the region of interest, where the x-coordinate is equal to the coverage of sample 635, and the y-coordinate is equal to the coverage of sample 636. The scatter plot shows a fairly narrow band of dots which is symmetric to the bisector, meaning that the coverage profiles in samples 635 and 636 are very similar. In other words, the enrichment and sequencing process is very reproducible, which is also expressed in the Pearson correlation coefficient of 0.996. The heatmap plot (**Figure S7a**) summarizes the Pearson correlation coefficients for all pairwise comparisons of coverage profiles between pairs of sample replicates. We obtained the best results with the 'standard' fragment library protocol, which exhibited both higher percentages of on-target reads (31.76% versus 19.29%) as well as better enrichment factors (16.49 versus 10.02) (**Figure S7a and S7c**). Consequently, we used the 'standard' protocol in our following experiments. The high ADoC from this stage (**Table A1**) ensured statistical reliability of the generated data and offered the basis to proceed to the next level and test the performance of the molecular barcoding scheme.

The better performance for the 'standard' over the 'express' SOLiD fragment library protocol (**Figure S7**) may imply that applying a small-sized (~ 150-200 bp) homogenous fragment population

to the array enhances sequence capture and coverage, and in turn improves the effective sequencing throughput. In contrast, using a different targeted resequencing approach, a recent study [12] showed that the efficiency of DNA resequencing using long-range PCR amplicons can be greatly improved by utilizing a larger library size. Increasing the library size, from 200 bp to 600 bp, reduced the coverage variability across the amplicon by 28%, thereby improving overall coverage uniformity. This experience highlights the need for more benchmarking experiments to provide optimized protocols for different available targeted enrichment methods depending on the scientific question being asked and methodological approach applied.

E. coli K12 (68 genes)	Reads	Bases	Bases mapped to genome	Bases mapped to target	on genome	on target	ADoC
standard-1	18,111,114	905,374,400	414,140,500	131,372,600	46%	32%	1470
standard-2	14,451,387	722,311,250	287,086,900	112,429,300	40%	39%	1258
standard-3	13,955,973	697,549,900	322,682,950	102,629,750	46%	32%	1149
standard-4	17,409,622	870,160,900	358,264,050	62,038,150	41%	17%	694
express-1	14,562,797	727,777,150	306,544,600	50,761,500	42%	17%	568
express-2	18,640,190	930,988,600	382,012,400	91,236,200	41%	24%	1021
express-3	13,466,991	672,748,300	252,491,250	48,281,600	38%	19%	540
express-4	16,441,568	820,859,100	305,845,550	59,511,200	37%	19%	666
illumina S3	11,790,026	424,440,936	288,334,000	77,620,000	68%	27%	862
illumina S8	11,072,622	398,614,392	293,460,732	67,520,592	74%	23%	750

Table A1: Mapping data of reads obtained from targeted enrichment of 68 *E. coli* genes. This table includes information about the mappable bases, on-target percentage, and average depth of coverage (ADoC) obtained from the first stage of experiments (**Figure 1A**). We compared febit HybSelect enrichment results between 4 technical replicates with the SOLiD 3 "standard" library protocol (50bps fragment), 4 technical replicates with the SOLiD 3 "express" library protocol (50bp fragment), and two formerly published Illumina GAII (36bp fragment) results (Schracke et al 2009). The best performance was obtained for the SOLiD 3 "standard" library protocol. We mapped the SOLiD data from this *E. coli* experiment using the CLC bio Workbench 3.7.1 software with default settings. The mappable bases refer to uniquely mapped reads multiplied with the fragment length.

Second stage – employing molecular barcodes for multiplexing targeted resequencing of the same 68 E. coli genes

One way to save resources and reduce costs is to multiplex barcoded NGS libraries, instead of using gDNA or individual libraries, and enrich them for a subset of the genome. In the second round of experiments we tested the enrichment of the same 68 *E. coli* genes at three different multiplexing levels (4-, 8-, and 20-plex) using molecular barcodes (**Figure 1**). A comprehensive analysis of the resequencing results of the three multiplexing experiments is shown in **Table A2**. In concordance with our findings from the first stage of the experiments (non-multiplexed *t*NGS), all library preparations for the following experiments were prepared according to the 'standard' fragment library protocol. The resulting average coverages were reproducible within each pool except for the sample with barcode four (**Figure A1**). In this stage, samples indexed with BC4 showed lower number of reads and consequently less coverage depth at intended bases. We suspected a systematic problem because this finding was confirmed by the results of the third stage (human *BRCA1/2* experiments). This issue is discussed in the 'General comments on the results of the second and third stages' section in this document.

The CLC bio mapping data in **Table A2** show that up to 42.1%, 37.4% and 41.3% of reads mapped to the target for the 4-plex, 8-plex, and 20-plex pools, respectively. Very low standard deviations of 1.0%, 1.4% and 1.9% were obtained for 4-plex, 8-plex, and 20-plex, respectively. The enrichment factor was reproducible for all barcodes and multiplexes (Supplementary Figure SF2). These two findings indicated that the enrichment step worked well, but that the quantifying, pipetting, or sequencing step worked poorly for barcode four. **Table A3** shows coverage metrics obtained with Corona Lite: The ADoC was consistent for each multiplexing fold. With the exception of BC 4, the ADoC per sample ranged from 575 to 671 (2138 per pool) for the 4-plex, 147 to 168 (1234 per pool) for the 8-plex, and 94 to 120 (2084 per pool) for the 20-plex pool. **Table A3** also demonstrates that 99.7% to 100% of the target region was covered with at least one read (1× coverage) in all of the multiplex experiments. At 20× coverage up to 99.7%, 98.2%, and 97.0% of the target region was covered in the 4-plex, 8-plex, and 20-plex pool, respectively.

E. Coli K12 (68 genes) pooled and bar-coded	Reads	uniquely mapped to genome	uniquely mapped to target	on genome	on target	ADoC
**4-plex**	**20,863,482**	**9,075,701**	**3,820,363**	**43.7%**	**42.1%**	**535**
BC1	6,074,647	2,617,680	1,106,147	43.1%	42.3%	619
BC2	5,539,829	2,462,846	1,027,764	44.5%	41.7%	575
BC3	6,655,640	2,834,618	1,198,559	42.6%	42.3%	671
BC4	*2,593,366*	*1,160,557*	*487,893*	*44.8%*	*42.0%*	*273*
**8-plex**	**16,954,098**	**5,895,302**	**2,204,262**	**34.9%**	**37.4%**	**154**
BC1	2,261,647	803,189	300,162	35.5%	37.4%	168
BC2	2,237,104	781,896	281,338	35.0%	36.0%	157
BC3	2,410,051	834,285	315,382	34.6%	37.8%	176
BC4	*973,344*	*351,767*	*134,000*	*36.1%*	*38.1%*	*75*
BC5	1,966,149	697,576	262,510	35.5%	37.6%	147
BC6	2,157,530	795,230	298,649	36.9%	37.6%	167
BC7	2,453,202	804,093	300,562	32.8%	37.4%	168
BC8	2,495,071	827,266	311,659	33.2%	37.7%	174
**20-plex**	**27,936,260**	**9,026,290**	**3,723,426**	**32.4%**	**41.3%**	**104**
BC1	1,521,507	491,378	197,480	32.3%	40.2%	111
BC2	1,331,110	442,744	189,902	33.3%	42.9%	106
BC3	1,550,163	496,354	211,300	32.0%	42.6%	118
BC4	*620,628*	*206,926*	*86,921*	*33.3%*	*42.0%*	*49*
BC5	1,138,154	371,252	156,889	32.6%	42.3%	88
BC6	1,146,956	394,596	171,604	34.4%	43.5%	96
BC7	1,291,443	401,606	170,402	31.1%	42.4%	95
BC8	1,373,499	420,512	179,159	30.6%	42.6%	100
BC9	1,525,047	489,006	193,971	32.1%	39.7%	109
BC10	1,351,928	434,516	174,849	32.1%	40.2%	98
BC11	1,595,282	508,307	212,588	31.9%	41.8%	119
BC12	1,735,077	559,424	228,165	32.2%	40.8%	128
BC13	1,392,977	460,016	187,698	33.0%	40.8%	105
BC14	1,304,122	417,336	167,669	32.0%	40.2%	94
BC15	1,570,915	459,460	185,810	29.2%	40.4%	104
BC16	1,808,034	551,830	219,807	30.5%	39.8%	123
BC17	1,339,572	462,183	191,027	34.5%	41.3%	107
BC18	1,325,781	447,226	184,661	33.7%	41.3%	103
BC19	1,639,678	492,454	198,447	30.0%	40.3%	111
BC20	1,374,387	519,164	215,077	37.8%	41.4%	120

Table A2: Mapping data of reads obtained from targeted enrichment of 68 *E. coli* genes using three multiplexing levels. This table includes information about the mappable reads, on-target percentage, and ADoC obtained from the second stage of experiments of 4-plex, 8-plex, and 20-plex pools as well as for the separate barcoded samples in each multiplex experiment (see also **Figure S8**). In our second stage, we pooled enriched barcoded libraries into three octant spots. We used the SOLiD Multiplexing Series B(1-20) barcode libraries, of which barcodes 1-16 are now used in the SOLiD BC Kit Module 1-16. We obtained uniform separation of barcoded reads in each spot, except for barcode 4 (highlighted in italics). The uniform mapping and ADoC results show that febit HybSelect is compatible with SOLiD barcoded libraries. The mapping results shown in this table were obtained using the CLC bio Workbench 3.7.1 software with default settings, which are lower than those obtained with Corona Lite (**Table A3**).

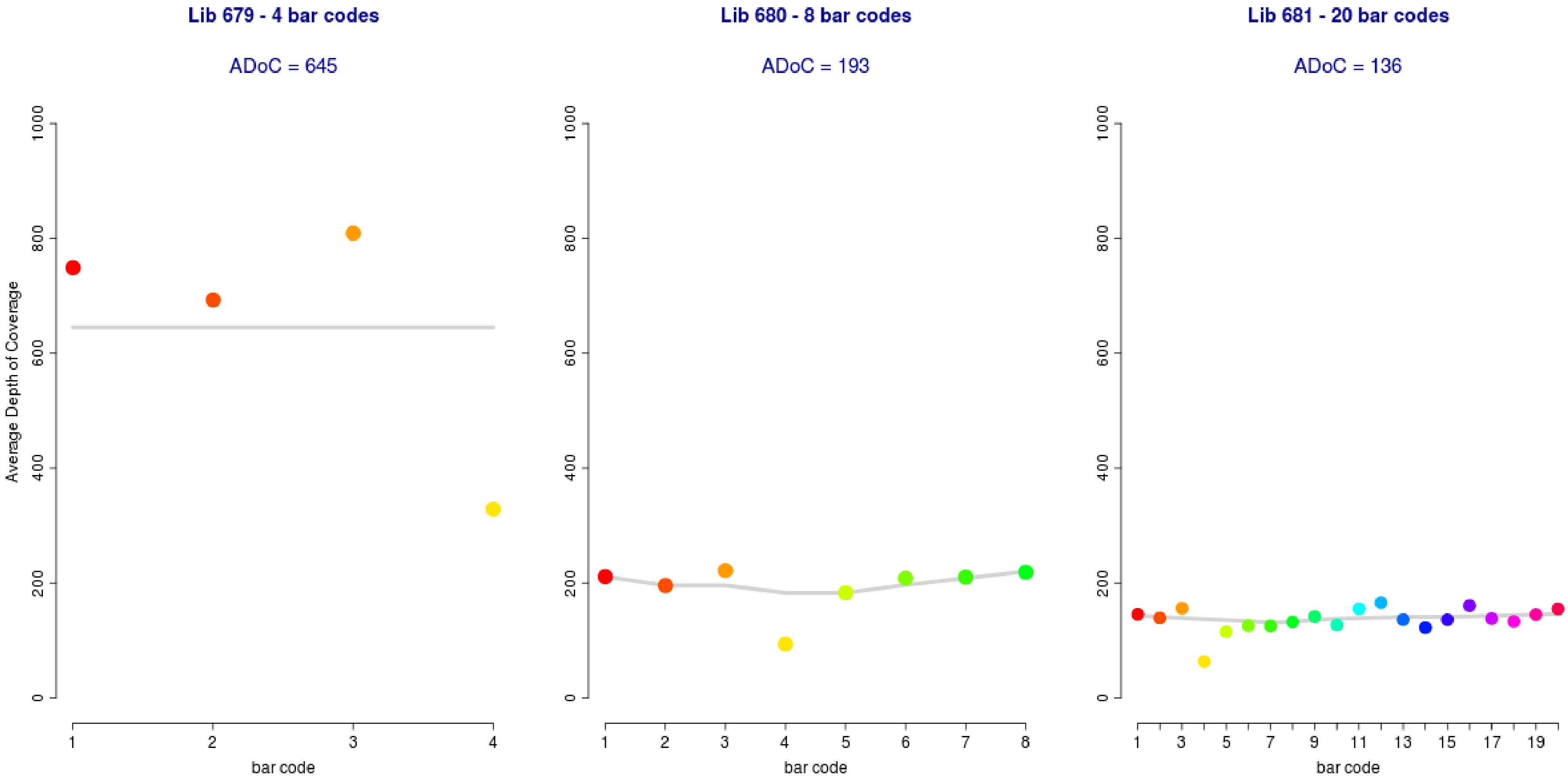

Figure A1: *E. coli* average depth of coverage (ADoC) for 4-plex, 8-plex, and 20-plex. The coverages across samples of each plex were uniform, except for barcode 4 (orange), for which fewer reads were assigned than expected.

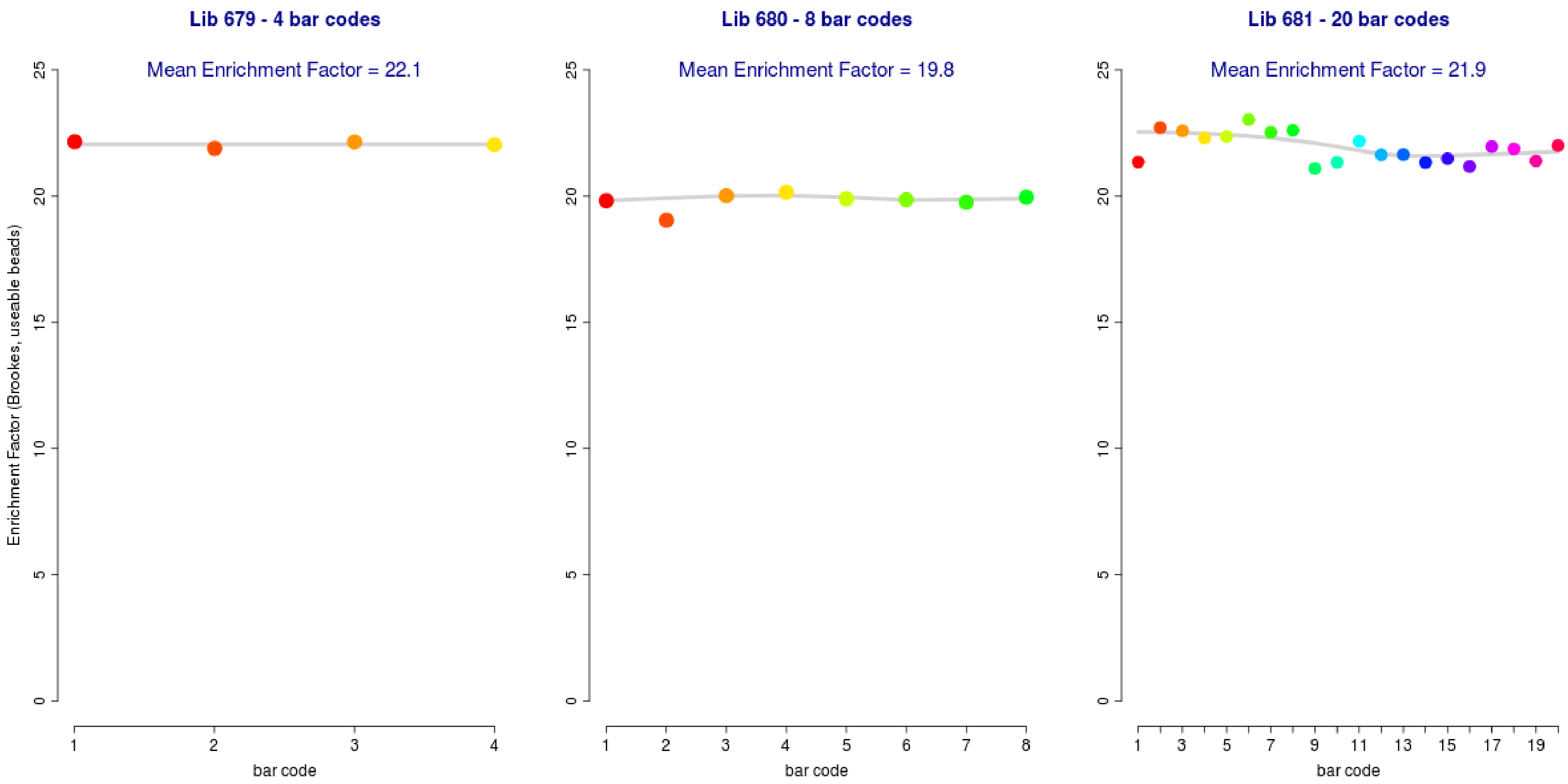

Figure A2: *E. coli* enrichment factors (EF) for 4-plex, 8-plex, and 20-plex. The graphs show almost ideal uniformity of enrichment in each sample. The enrichment factor is by definition independent of the number of reads (formula (1) in this document), therefore barcode 4 (orange) is not affected here. Because EF depends on the genome size divided by the target region size, the theoretically maximal EF is 51.93 (see `Ecoli_supplement.xls`, sheet ET1, cell N28), meaning that our experimental results of 20× – 22× are well within the expected range.

We observed a uniform coverage distribution (**Table A3** and **Figure S8**). The Pearson correlation coefficients were slightly lower in the second stage than in the first stage (**Figure S8a**), and the scatter plots (**Figure S8b**) were less banded. These two metrics each indicated that the coverage profiles were slightly less reproducible between barcoded replicates than between non-barcoded replicates. This is further illustrated by the coverage spectrum plot (**Figure S8c**). The reproducibility of the coverage profiles (and hence the reproducibility of the tNGS approach), as indicated by Pearson coefficients, was excellent for the 4-plex and 8-plex, but less good for the 20-plex (**Figure S8a**). Nonetheless, up to 99.7%, 98.2% and 97.0% of the target region was covered at 20× or higher in the 4-plex, 8-plex and 20-plex pools, respectively (**Table A3**). Interestingly, the coverage distribution was more stable and uniform at higher multiplexing folds (**Figure S8a**). The results summarized in **Figure S8c** show that average maximum and mean coverage depth per sample were as high as 1250 and 645 for the 4-plex pool, 434 and 193 for the 8-plex pool, and 299 and 136 for the 20-plex pool, respectively. The difference between maximum and mean depth of coverage is approximately 50%, and comparable between all three pools, regardless of the magnitude of multiplexing. The enrichment factor was almost identical at different multiplexing levels, showing high uniformity for all 4 BCs tested in the 4-plex experiment (**Figure A2**). Up to 19-22-fold enrichment of the target region was achieved for all pools tested (**Supplementary *E. coli* Table ET1** on the project homepage). The correlation matrix of Pearson values (**Figure S9a**) and the representative scatter plot (**Figure S9b**) show that the individual experiments in this stage were highly correlated.

E. Coli K12 **(68 genes) bar-coded**	**ADoC**	**sdv**	**1× Cov.**	**5× Cov.**	**10× Cov.**	**20× Cov.**	**100× Cov.**
**4-plex**	**645**	**216**	**100.0%**	**99.9%**	**99.7%**	**99.5%**	**96.1%**
BC1	749		100.0%	99.9%	99.8%	99.6%	97.5%
BC2	693		100.0%	99.9%	99.8%	99.6%	97.3%
BC3	809		100.0%	99.9%	99.8%	99.7%	97.8%
BC4	*329*		*100.0%*	*99.8%*	*99.5%*	*99.1%*	*91.7%*
**8-plex**	**193**	**42**	**99.9%**	**99.5%**	**98.9%**	**97.4%**	**72.6%**
BC1	211		99.9%	99.6%	99.3%	98.0%	78.0%
BC2	196		99.9%	99.6%	99.1%	97.7%	75.1%
BC3	222		99.9%	99.7%	99.3%	98.2%	80.1%
BC4	*94*		*99.8%*	*98.9%*	*97.3%*	*93.3%*	*38.5%*
BC5	183		99.9%	99.6%	99.0%	97.7%	73.0%
BC6	209		99.9%	99.6%	99.2%	98.0%	78.0%
BC7	210		99.9%	99.6%	99.2%	98.0%	78.3%
BC8	219		99.9%	99.7%	99.2%	98.1%	79.5%
**20-plex**	**136**	**22**	**99.8%**	**99.3%**	**98.3%**	**95.9%**	**61.3%**
BC1	146		99.9%	99.4%	98.7%	96.6%	66.0%
BC2	139		99.9%	99.4%	98.6%	96.4%	63.9%
BC3	156		99.9%	99.4%	98.8%	96.9%	69.2%
BC4	*64*		*99.7%*	*98.2%*	*95.8%*	*88.5%*	*14.2%*
BC5	115		99.8%	99.3%	98.3%	95.5%	53.4%
BC6	126		99.8%	99.3%	98.4%	96.1%	59.4%
BC7	126		99.9%	99.3%	98.4%	95.7%	58.6%
BC8	133		99.8%	99.4%	98.5%	96.3%	61.5%
BC9	142		99.8%	99.2%	98.4%	96.2%	65.0%
BC10	127		99.8%	99.2%	98.2%	95.8%	58.8%
BC11	155		99.9%	99.3%	98.5%	96.7%	69.0%
BC12	166		99.9%	99.4%	98.8%	97.0%	71.8%
BC13	136		99.8%	99.3%	98.4%	96.3%	63.1%
BC14	122		99.7%	99.1%	98.2%	95.4%	57.2%
BC15	136		99.9%	99.2%	98.3%	96.2%	62.5%
BC16	161		99.9%	99.5%	98.7%	97.0%	70.6%
BC17	138		99.9%	99.3%	98.5%	96.2%	63.7%
BC18	122		99.9%	99.2%	98.3%	96.2%	62.2%
BC19	145		99.9%	99.3%	98.4%	96.5%	65.5%
BC20	155		99.9%	99.4%	98.7%	96.9%	69.6%

Table A3: Coverage analysis of 68 *E. coli* genes using three multiplexing levels. This table includes information about the mean ADoC, standard deviation of mean ADoC within each plex, and percentage of target region covered by at least 1, 5, 10, 20, or 100 reads. The mapping results shown in this table were obtained using the SOLiD Corona Lite 4.0 software with default settings. ADoC is 20%-30% higher than in **Table A2** because Corona Lite does not truncate reads (see Methods). The results are uniform for each barcode within a plex, except for barcode 4 (highlighted in italics), as noted in **Table A2**.

Third stage: Enrichment of human BRCA1/2 genes (barcoded tNGS pools)

Coverages and reproducibility

The coverages were reproducible for all barcodes except BC4 (**Table S1**). The problem with BC4 is described in the *E.coli* experiments above, and appears to be a systematic problem. We found that the coverage completeness in the controls and the 4 plex pools was highly reproducible. Up to 99.6%, 98.2%, 92.3%, 92.2, and 87.8% of the target region was covered with at least one read (≥1× coverage) for the control libraries, 4-plex, 8-plex, 16-plex and 20-plex pools, respectively (**Table S1**). The reproducibility between experiments can be seen in **Figure A3 and A4** (Yoruban 4-plex) and **Figure A5** (Yoruban and Chinese sample). At 8× coverage – a minimum SNP detection threshold employed by others in previous NGS studies – 98.3%, 94.1%, 78.8%, 71.0% and 51.7% of the targeted bases were covered in the control libraries, 4-plex, 8-plex, 16-plex and 20-plex, respectively (**Table S4**). At 20× coverage 97.3%, 89.2%, 69.0%, 59.2% and 32.2% of the targeted bases were covered in the control libraries, 4-plex, 8-plex, 16-plex and 20-plex, respectively. The target region coverage distributions were reproducible across the barcoded samples in each resequenced pool (**Table S1** and **Figures S1a and S1b**). The data was consistent at each multiplexing level (**Table S1** and **Figure S1a**). The comparison of the different multiplexing experiments to the non-barcoded control libraries indicated realistic average maximum and mean coverages (**Figure S1a**): >8600× and 2100× for the control libraries, >3400× and 650× for the 4-plex pools, >2250× and 340× for the 8-plex pool, >1430× and 190× for the 16-plex pool, and >370× and 30× for the 20-plex pool, respectively. Furthermore, we observed comparable enrichment factors for the control libraries (12256×) and all the multiplexing experiments (8519× and 15598× for the 4-plex, 13839× for the 8-plex, 13103× for the 16-plex, to 6897× for the 20-plex) as well as within each resequenced pool (**Figure S1c**). The number of reads aligned uniquely to *BRCA1/2* target regions (**Table S26**) using different mapping tools are summarized in **Table 25** and **Figure 2**.

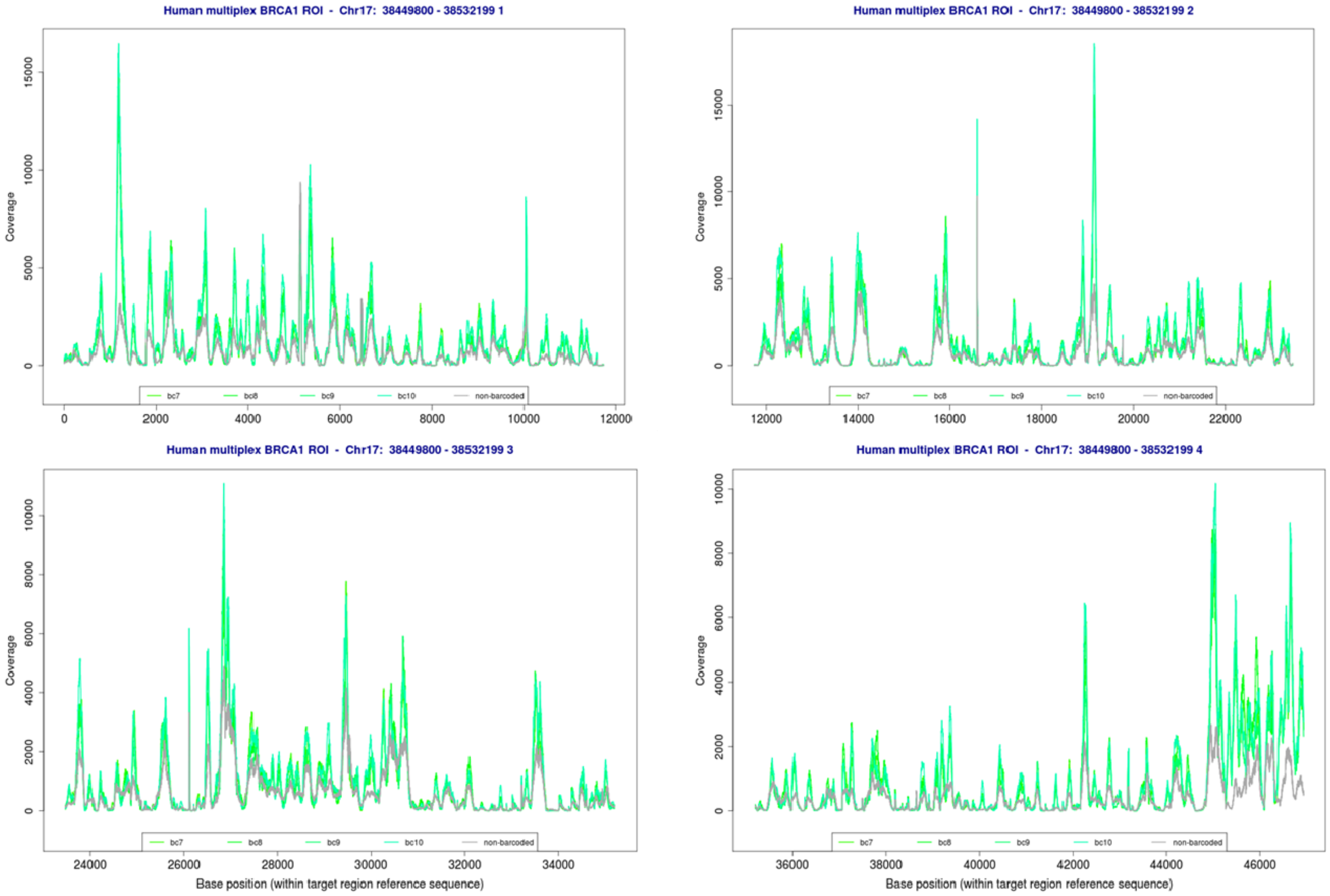

Figure A3: Coverage overview for the *BRCA1* gene region of interest for the human multiplex samples. Coverage distribution overview for the entire *BRCA1* gene target region in the example of the 4-plex2 reads processed with SAET and mapped with Bioscope 1.0.1. For the purpose of this coverage plot we removed the low-complexity regions between the individual target regions and plotted concatenation of the target regions. The coverage peaks reach 10,000-20,000× and some regions are less well covered than others.

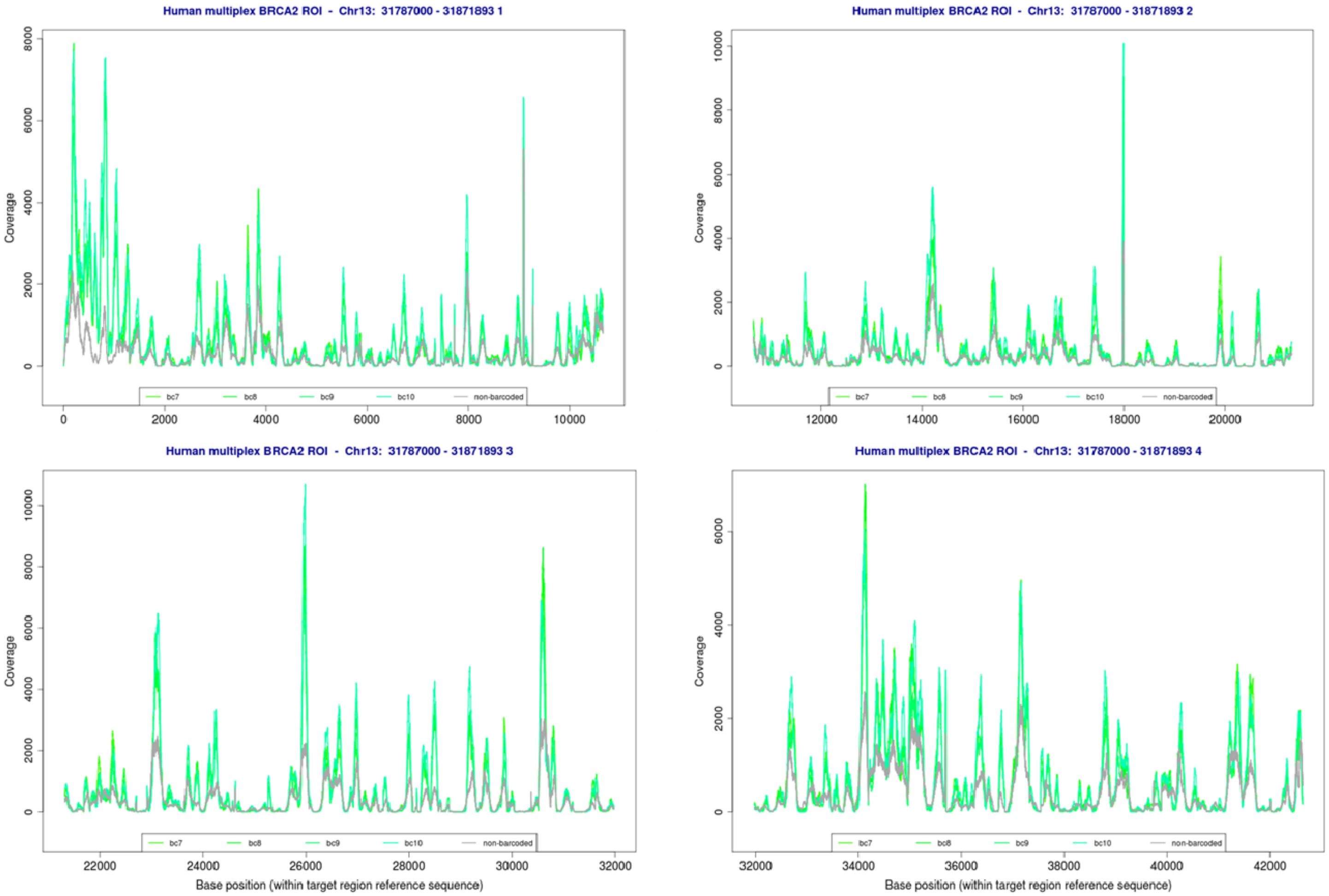

Figure A4: Coverage overview for the *BRCA2* gene region of interest for the human multiplex samples. Coverage distribution overview for the entire *BRCA2* gene target region in the example of the 4-plex2 reads processed with SAET and mapped with Bioscope 1.0.1. For the purpose of this coverage plot we removed the low-complexity regions between the individual target regions and plotted concatenation of the target regions. The coverage peaks reach 5,000-11,000× and some regions are less well covered than others.

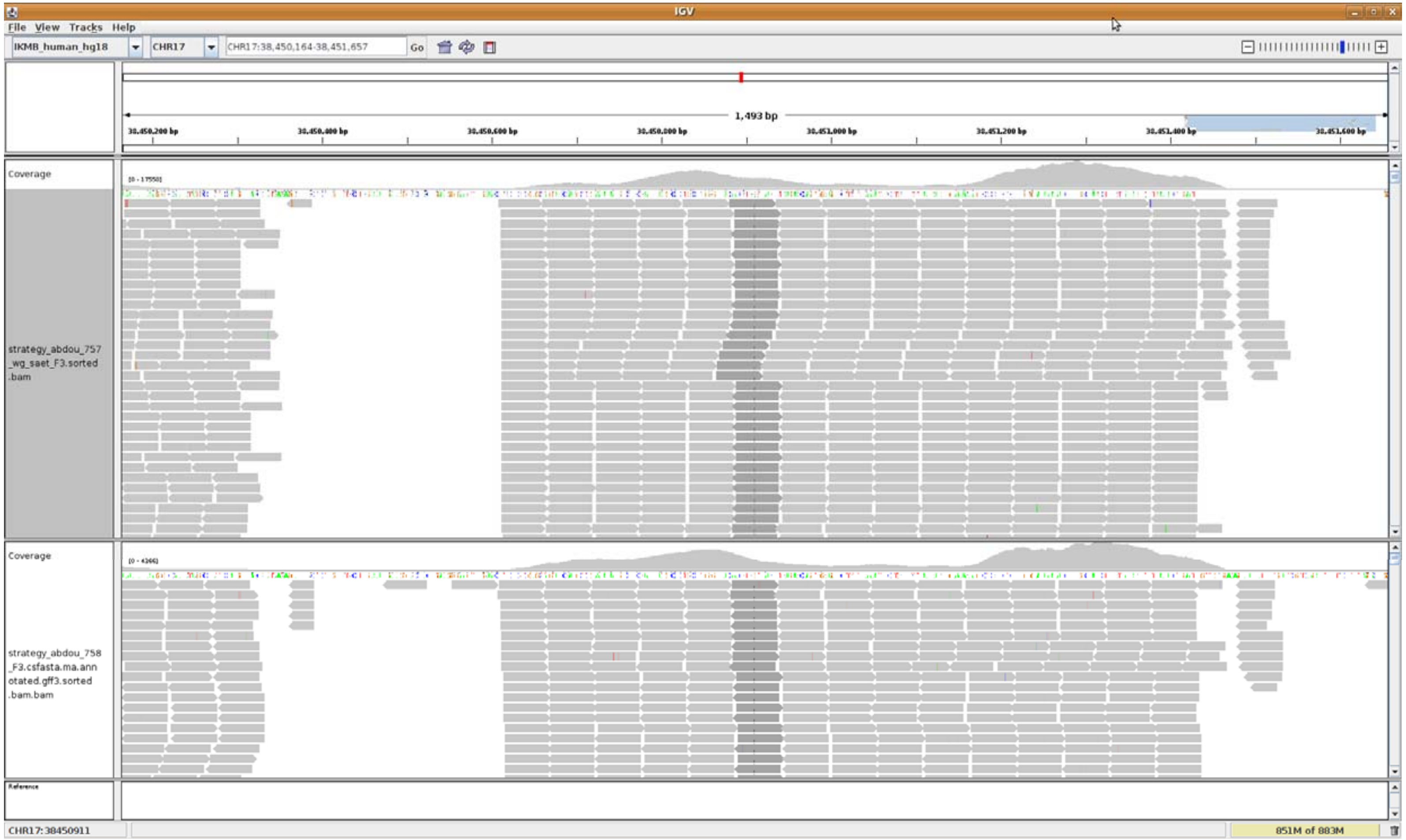

Figure A5: IGV viewer showing read mapping to target regions 1+2 of *BRCA1/2* for Yoruban (top) and Chinese (bottom) non-barcoded control sample. The reads include technical replicates of identical (good) quality and also show sufficiently good tiling (overlapping) and forward/reverse strand balance. The tiling of mapped reads and strand balance are influenced by capture probe design. The target region starts and ends are clearly visible from the "pileup" of reads.

General comments on the results of the second and third stages

The overall results from second and third stages indicate that the sequenced pools did not substantially affect capture efficiency but rather increased throughput. It seems plausible that integration of multiplexed NGS libraries to sequence capture before entering into the NGS workflow could reduce, to a certain extent, a fraction of the greater variability in the number of sequenced reads per indexed sample, which has been previously exhibited using a non-selective NGS approach [8]. The achieved uniform coverage distribution is crucial for the detection of heterozygous variants in particular. It is important to capture both alleles of a SNP in sufficient abundance so that such variants can be detected.

The data generated in the present study reflect adequate performance of all multiplexed human samples and controls except for (i) the lower coverage metrics compared to multiplexing using *E. coli* DNA/target (second stage; **Table A3**), (ii) the 20-plex experiment (20-61× ADoC) and (iii) the bias revealed from samples indexed with BC 4 (2-22× ADoC). Given the low complexity of the human genome, due to the presence of more highly repetitive sequences than in *E. coli* genome and shared homologies within closely related gene families and pseudogenes [10], the number of uniquely aligned reads can be substantially reduced when aligning to the human genome compared to smaller *E. coli* genomes. This is consistent with the observation that 97% of the *E. coli* genome can be uniquely aligned with 18-bp reads but less than 90% of the human genome can be uniquely aligned with 30- to 50-bp reads [13-15]. Further decreasing performance can also arise from increasing sample multiplexing to a certain degree; 20-plex in our case. The drop in performance at higher multiplexing degrees is expected and can be accepted as long as it does not impact on the accuracy of the downstream SNP calling. Even so, we can modify the experimental design according to project needs and required output. For instance, to improve coverage for larger target regions, sequencing could be performed using a lower degree of multiplexing (4-, 8-, or 10-plex scheme) on a quarter of a sequencing slide (quads; 4-well slide) instead of on an eighth of a slide (octant; 8-well slide). Following such a strategy we allow more sequencing room and subsequent higher coverage magnitude; by decreasing the inherent effect of slide's physical separation that decreases the overall number of sequences obtained [16]. For each study and a given region of interest, we can vary the experimental design according to specific needs and objectives, keeping in mind the desired enrichment fold and specificity and available budget. With respect to BC 4, we observed that samples indexed with BC 4 in both the second (*E. coli*; **Table A3**) and the third (human; **Figure S1b)** stages were extremely under-represented at all tested multiplexing levels. This bias of samples with BC 4 has also been revealed in other multiplex experiment, where a different targeted enrichment method was used (data not shown). Therefore, it is unlikely that the odd performance and strong bias of samples indexed with BC 4 were due to lower representation of that barcode in the original libraries

before pooling/enrichment. Accordingly, we prefer to exclude BC 4, and use other ABI SOLiD barcodes instead, for instance BC 5-20, in our following multiplexing experiments to achieve more even bead distribution and coverage.

While the absolute coverages achieved in the *BRCA1/2* target regions were high throughout all samples, we also observed an unexpectedly low on-target percentage in the non-barcoded Chinese control spot (see **Tables S4, S22-S24** for more details). We found that this percentage is even lower for the Bioscope tool than for mapping using Corona Lite, because Bioscope 1.0.1 may map a short segment of a read which corona lite will discard as unmappable. Fast read mapping methods can map a small fraction of reads to non-optimal positions, and we observed exactly this discrepancy when we started testing Bioscope 1.2 (see 'Mapping the SOLiD sequencing data' in the supplement). We suspect that these short segments of short reads were more susceptible to mismapping than complete reads. It should also be kept in mind that NGS data may include reads from regions previously refractory to sequencing, and that these reads cannot be mapped to a reference genome which does not include these regions. A substantial fraction of reads from the genomes sequenced to date could not be mapped to the reference genome, which is not surprising as ~ 1-2 Mb of euchromatic sequence is absent from the reference genome. A fraction of the genome remains uncharted in regions that were refractory to Sanger sequencing; 341 gaps remained in the gold standard reference genome, 250 of which were in euchromatic regions (reviewed in [17]). Other studies [18-20] argue that off-target reads and regions with lower coverage may be explained by the vicinity to extremely repeat- or GC-rich contents, or by sequence misalignment, rather than by sequencing errors.

Gold Standard consensus genotype list

As our "gold" standard for genotype concordance and SNP overlap analysis we created a consensus SNP list for the Yoruban HapMap individual NA18507 from the HapMap data (http://www.sanger.ac.uk/resources/downloads/human/hapmap3.html), the whole genome Illumina NGS data [1] (ftp://hgdownload.cse.ucsc.edu/goldenPath/hg18/database/, `pgYh1.txt.gz`), and the whole genome SOLiD NGS data (http://solidsoftwaretools.com/gf/project/yoruban/, `Yoruban_snp_18x.gff`). This resulted in 15 non-reference genotype SNPs (**Table S19**). HapMap SNPs rs28897731, rs28897686, rs4986848, and rs28897673 were removed from the consensus, because they were not confirmed by the two different NGS platforms. Manual inspection of our 17 highly covered Yoruban NA18507 libraries validated the published NGS results (**Table S8**).

Silver Standard consensus genotype list

To allow a more extensive validation of our genotypes for the Yoruban HapMap individual NA18507, we defined our "silver" standard - the consensus list of the aforementioned whole genome NGS SNP lists (Illumina: [1]; ftp://hgdownload.cse.ucsc.edu/goldenPath/hg18/database/`pgYh1.txt.gz`;

SOLiD: http://solidsoftwaretools.com/gf/project/yoruban/,`Yoruban_snp_18x.gff`). This resulted in 66 non-reference genotypes.

Bronze Standard consensus genotype list

For the Beijing Han Chinese HapMap individual NA18561, our standard was the pure HapMap3 genotype list with 28 non-reference genotypes. Highly covered whole genome NGS SNPs relating to the hg18 assembly were not readily available. The 1000 Genomes Project Consortium data for the Chinese HapMap individual NA18561 relating to assembly version hg18 (ftp://ftp.1000genomes.ebi.ac.uk/vol1/ftp/pilot_data/data/NA18561/alignment/) are based on low coverages, and the data relating to hg19 (ftp://ftp.1000genomes.ebi.ac.uk/vol1/ftp/data/NA18561/alignment/) requires coordinate transformations with the risk of introducing errors. The results are summarized in **Table S21**.

SNPs detected by Bioscope which were not in the Silver Standard

For the Yoruban sample (e.g. the non-barcoded control), Bioscope called two known SNPs (namely rs11571666 and rs8176279) which were not known in the "silver" consensus. Both SNPs are near short tandem repeat regions. We did not exclude rs11571666 to be a T/C genotype (reference base is T, located next to a TG-repeat region), because the C allele was confirmed when only well-mapped high-quality reads (minimal length 49, minimal mapping quality 20, minimal base quality 20) were considered. However, we excluded the rs8176279 A/C heterozygote (reference base is A, located next to a CA-repeat region) as a mapping artefact, because the C allele was not confirmed when only well-mapped high-quality reads (see above) were considered.

TABLE AND FIGURE LISTS

Tables and Figures Shown in this Supplemental Note

Tables:

A1: Mapping data of reads obtained from targeted enrichment of 68 *E. coli* genes.

A2: Mapping data of reads obtained from targeted enrichment of 68 *E. coli* genes using three multiplexing levels.

A3: Coverage analysis of 68 *E. coli* genes using three multiplexing levels.

Figures:

A1: *E. coli* average depth of coverage (ADoC) for 4-plex, 8-plex, and 20-plex.

A2: *E. coli* enrichment factors (EF) for 4-plex, 8-plex, and 20-plex.

A3: Coverage overview for the *BRCA1* gene region of interest (ROI) for the human multiplex samples.

A4: Coverage overview for the *BRCA2* gene region of interest (ROI) for the human multiplex samples.

A5: IGV viewer showing read mapping to target regions 1+2 of *BRCA1/2* for Yoruban (top) and Chinese (bottom) non-barcoded control sample.

List of Supplementary Tables (Supplementary_tables.pdf)

E.coli (First and Second Stages) Supplementary Tables:

(http://www.ikmb.uni-kiel.de/tngs-backmapping/Ecoli_supplement.pdf)

ET1: Mapping, enrichment and ADoC for *68 E.coli* genes using febit Hybselect, SOLiD 3 (50bp fragments), and CLC bio Genomics Workbench software 3.7.1

ET2: Mapping, enrichment and ADoC for *68 E.coli* genes using febit Hybselect, SOLiD 3 (50bp fragments), and SOLiD software Corona Lite whole genome mapping

ET3: Mapping summary for Corona Lite v4.0r2.0 whole genome mapping and target region mapping (fragment length 50, 4 mismatches), all bar coded *E.coli* samples

ET4: List of 68 *E. coli K12* genes targeted for enrichment

BRCA1/2 (Third Stage) and Backmapping/Exome Experiments (Fourth Stage) Supplementary Tables (Supplementary_tables.pdf):

S1: Multi-sample enrichment (AUC) and coverage results for the target regions in BRCA1 and BRCA2

S2: diBayes SNP-calling for Yoruban samples versus our Gold Standard consensus

S3: diBayes SNP-calling for Yoruban samples versus our Silver Standard consensus

S4: Detailed summary for Bioscope 1.0.1 whole genome and target mapping, all HapMap samples. WG using off-machine reads, TR using SAET 2.2 enhanced reads.

S5: Table for SNP summary Figure 7

S6: Initial Corona Lite / stand-alone diBayes SNP-caller results comparison against HapMap3

S7: diBayes SNP-calling for Yoruban Samples versus our Gold Standard and Chinese samples versus HapMap (our Bronze Standard)

S8: Accurate sequencing is confirmed for the Yoruban samples using the Integrated Genomics Viewer (IGV)

S9: CLC bio Workbench 3.7.1 SNP-List after target region mapping (Yoruban HapMap ID NA18507, sample 4-plex1bc05)

S10: NextGENe V2 Annotated SNP-List after whole genome mapping (Yoruban HapMap ID NA18507, sample 4-plex1bc05)

S11: diBayes SNP-calling for Chinese samples versus our Bronze Standard

S12: In silico novel SNP validation by computing inter-sample intra-plex SNP concordances

S13: Potential novel SNPs manually inspected and selected for Sanger re-sequencing

S14: Exome Sequencing Run Info for the selected public exome sequencing data and public genome sequencing data

S15: Exome comparison of mapping and SNP-calling times for Proposed Combined Target region Mapping + Whole Genome Read-Backmapping (TR) vs. Conventional Whole-Genome-Mapping (WG)

S16: Exome comparison of SNP-results for Proposed Combined Target region Mapping + Whole Genome Read-Backmapping (TR) vs. Conventional Whole-Genome-Mapping (WG)

S17: Coverage differences between plus and minus strand in the *BRCA1* and *BRCA2* regions

S18: Potential Energy/CO2-Footprint Reduction resulting from Proposed Combined Targetregion Mapping + Whole Genome Read-Backmapping (TR) for exome data

S19: Our Gold/Silver Standards (Yoruban consensus) and Bronze Standard (Chinese, raw HapMap3)

S20: Upper bound estimate for false positive SNPs via inter-sample SNP validation between barcoded samples versus non-barcoded controls

S21: Summary of Mapping and SNP-calling with Bioscope 1.0.1/diBayes on all samples

S22: Detailed mapping summary for off-machine reads using Bioscope 1.0.1

S23a: Detailed summary for Corona Lite v4.0r2.0 whole genome and target mapping (fragment length 50, 5 mismatches), all HapMap samples

S23b: Detailed summary for Corona Lite v4.0r2.0 target region mapping (fragment length 50, 4 mismatches), all HapMap samples.

S24: Detailed summary for SAET 2.2 / Corona Lite v4.0r2.0 whole genome and target mapping (fragment length 50, 5 mismatches), all HapMap samples.

S25: On-target reads (uniquely mapped) for different mapping strategies

S26: *BRCA1/2* target regions (1-based hg18 / NCBI36 coordinates)

List of Supplementary Figures (Supplementary_figures.pdf)

S1: Coverage distribution across all the barcoded human samples for different multiplexing levels at the *BRCA1/2* genes (third stage of our tNGS test model).

S2: *BRCA1*/2 target region ROC curves and AUC values for whole genome coverages obtained using SAET 2.2 read enhancement and Bioscope 1.0.1 read mapping.

S3: Manual inspection in IGV uncovers SNPs which Bioscope did not call.

S4: Cleaning up SNP-artifacts from target-region mapping by employing read-backmapping to the whole genome (fourth stage, exome data).

S5: Sanger traces for 4 resequenced SNPs.

S6: Multi-sample coverage plots give valuable insights into mapping uniformity and regions of high and low mapping.

S7: Sample correlation, reproducibility, and consensus coverage for the tested eight technical replicates (first stage of our tNGS test model).

S8: Coverage analysis of barcoded samples (second stage of our tNGS test model).

S9: Correlation and reproducibility of multiplexed targeted NGS approach (second stage of our tNGS test model).

Bioscope Settings: *plan* and *ini* files

http://www.ikmb.uni-kiel.de/tngs-backmapping/bioscope_settings.xls

REFERENCES

1. Bentley DR, Balasubramanian S, Swerdlow HP, Smith GP, Milton J, Brown CG, Hall KP, Evers DJ, Barnes CL, Bignell HR, et al: **Accurate whole human genome sequencing using reversible terminator chemistry.** *Nature* 2008, **456:**53-59.
2. Bordeleau L, Panchal S, Goodwin P: **Prognosis of BRCA-associated breast cancer: a summary of evidence.** *Breast Cancer Res Treat* 2010, **119:**13-24.
3. Kurian AW: **BRCA1 and BRCA2 mutations across race and ethnicity: distribution and clinical implications.** *Curr Opin Obstet Gynecol* 2010, **22:**72-78.
4. ten Bosch JR, Grody WW: **Keeping up with the next generation: massively parallel sequencing in clinical diagnostics.** *J Mol Diagn* 2008, **10:**484-492.
5. Summerer D, Wu H, Haase B, Cheng Y, Schracke N, Stahler CF, Chee MS, Stahler PF, Beier M: **Microarray-based multicycle-enrichment of genomic subsets for targeted next-generation sequencing.** *Genome Res* 2009, **19:**1616-1621.
6. Lander ES, Linton LM, Birren B, Nusbaum C, Zody MC, Baldwin J, Devon K, Dewar K, Doyle M, FitzHugh W, et al: **Initial sequencing and analysis of the human genome.** *Nature* 2001, **409:**860-921.
7. Mardis ER: **The impact of next-generation sequencing technology on genetics.** *Trends Genet* 2008, **24:**133-141.
8. Metzker ML: **Sequencing technologies - the next generation.** *Nat Rev Genet* 2010, **11:**31-46.
9. Valouev A, Ichikawa J, Tonthat T, Stuart J, Ranade S, Peckham H, Zeng K, Malek JA, Costa G, McKernan K, et al: **A high-resolution, nucleosome position map of C. elegans reveals a lack of universal sequence-dictated positioning.** *Genome Res* 2008, **18:**1051-1063.
10. Voelkerding KV, Dames SA, Durtschi JD: **Next-generation sequencing: from basic research to diagnostics.** *Clin Chem* 2009, **55:**641-658.
11. Altschul SF, Gish W, Miller W, Myers EW, Lipman DJ: **Basic local alignment search tool.** *J Mol Biol* 1990, **215:**403-410.
12. Harismendy O, Frazer K: **Method for improving sequence coverage uniformity of targeted genomic intervals amplified by LR-PCR using Illumina GA sequencing-by-synthesis technology.** *Biotechniques* 2009, **46:**229-231.
13. Durbin RM, Abecasis GR, Altshuler DL, Auton A, Brooks LD, Gibbs RA, Hurles ME, McVean GA: **A map of human genome variation from population-scale sequencing.** *Nature* 2010, **467:**1061-1073.
14. Warren RL, Sutton GG, Jones SJ, Holt RA: **Assembling millions of short DNA sequences using SSAKE.** *Bioinformatics* 2007, **23:**500-501.
15. Whiteford N, Haslam N, Weber G, Prugel-Bennett A, Essex JW, Roach PL, Bradley M, Neylon C: **An analysis of the feasibility of short read sequencing.** *Nucleic Acids Res* 2005, **33:**e171.
16. Meyer M, Stenzel U, Myles S, Prufer K, Hofreiter M: **Targeted high-throughput sequencing of tagged nucleic acid samples.** *Nucleic Acids Res* 2007, **35:**e97.
17. Mir KU: **Sequencing genomes: from individuals to populations.** *Brief Funct Genomic Proteomic* 2009, **8:**367-378.
18. Gnirke A, Melnikov A, Maguire J, Rogov P, LeProust EM, Brockman W, Fennell T, Giannoukos G, Fisher S, Russ C, et al: **Solution hybrid selection with ultra-long oligonucleotides for massively parallel targeted sequencing.** *Nat Biotechnol* 2009, **27:**182-189.
19. Hoischen A, Gilissen C, Arts P, Wieskamp N, van der Vliet W, Vermeer S, Steehouwer M, de Vries P, Meijer R, Seiqueros J, et al: **Massively parallel sequencing of ataxia genes after array-based enrichment.** *Hum Mutat* 2010, **31:**494-499.
20. Porreca GJ, Zhang K, Li JB, Xie B, Austin D, Vassallo SL, LeProust EM, Peck BJ, Emig CJ, Dahl F, et al: **Multiplex amplification of large sets of human exons.** *Nat Methods* 2007, **4:**931-936.
