# Supplementary material for: Improving mapping and SNP-calling performance in multiplexed targeted next-generation sequencing
Source: BMC Genomics. 2012 Aug 22;13:417. doi: 10.1186/1471-2164-13-417 (PMC3563481; doi:10.1186/1471-2164-13-417)
Supplement: Additional file 2 — Supporting Tables. Tables S1-S26. [file 1471-2164-13-417-S2.pdf]

Supplementary Tables S1-S26

Improving mapping and SNP-calling performance in multiplexed targeted next-generation sequencing

Abdou ElSharawy[1,5], Michael Forster[1, 5], Nadine Schracke[2], Andreas Keller [3], Ingo Thomsen[1], Britt-Sabina Petersen[1], Björn Stade[1], Peer Stähler[2], Stefan Schreiber[1,4], Philip Rosenstiel[1] and Andre Franke[1]

1 Institute of Clinical Molecular Biology, Christian-Albrechts-University, Kiel, Germany

2 Febit biomed GmbH, Heidelberg, Germany

3 Biomarker Discovery Center, Heidelberg, Germany

4 Department of General Internal Medicine, Campus Kiel, University Hospital S.-H., Germany

5 These authors contributed equally to this work

Email addresses

AE: a.sharawy@mucosa.de

MF: m.forster@ikmb.uni-kiel.de

NS: nadine@schracke.de

AK: keller.andreas@siemens.com

BS: b.stade@ikmb.uni-kiel.de

B-SP: b.petersen@ikmb.uni-kiel.de

IT: i.thomsen@ikmb.uni-kiel.de

PFS: Peer.staehler@t-online.de

SS: s.schreiber@mucosa.de

PR: p.rosenstiel@mucosa.de

AF: a.franke@mucosa.de

Table S1: Multi-sample enrichment (AUC) and coverage results for the target regions in BRCA1 and BRCA2.

Yoruban HapMap NA18507									Chinese HapMap NA18561								
Bar codes in spot	AUC	target region bases covered					ADoC	EF	Bar codes in spot	AUC	target region bases covered					ADoC	EF
		1X	10X	20X	30X	50X					1X	10X	20X	30X	50X		
**No BC**	**0.99**	**99.6%**	**98%**	**98%**	**97%**	**95%**	**3119.23**	**21399**	**No BC**	**0.99**	**99.6%**	**98%**	**96%**	**95%**	**92%**	**1084.07**	**5298**
**4-plex 1**	**0.98**	**98.0%**	**93%**	**88%**	**85%**	**79%**	**457**	**12296**	**4-plex 1**	**0.98**	**97.5%**	**90%**	**85%**	**81%**	**74%**	**357**	**11312**
BC5	0.98	98.2%	93%	89%	86%	80%	515	12470	BC7	0.98	97.5%	90%	85%	81%	74%	345	11240
BC6	0.98	97.8%	92%	88%	84%	78%	399	12121	BC8	0.98	97.4%	90%	85%	81%	75%	369	11383
**4-plex 2**	**0.98**	**96.9%**	**92%**	**89%**	**86%**	**82%**	**865**	**25173**	**4-plex 2**	**0.97**	**97.5%**	**93%**	**90%**	**87%**	**83%**	**848**	**25646**
BC9	0.98	97.0%	91%	88%	85%	81%	765	24566	BC7	0.97	97.5%	93%	89%	87%	83%	830	26399
BC10	0.98	96.8%	93%	89%	87%	83%	965	25781	BC8	0.97	97.5%	93%	90%	87%	83%	865	24892
**8-plex**	**0.94**	**91.0%**	**83%**	**78%**	**74%**	**67%**	**431**	**20718**	**8-plex**	**0.88**	**80.6%**	**67%**	**60%**	**55%**	**48%**	**230**	**18032**
BC1	0.94	92.3%	86%	81%	78%	72%	561	21424	BC3	0.93	89.1%	79%	73%	68%	61%	333	18311
BC2	0.94	90.6%	83%	77%	73%	66%	403	21536	*BC4*	*0.77*	*57.1%*	*32%*	*25%*	*21%*	*15%*	*25*	*15957*
BC5	0.94	90.8%	83%	78%	73%	66%	421	20315	BC7	0.92	88.4%	78%	71%	66%	59%	273	19024
BC6	0.93	90.2%	81%	75%	70%	63%	339	19595	BC8	0.92	87.8%	77%	71%	66%	59%	290	18836
**16-plex**	**0.91**	**85.3%**	**71%**	**63%**	**58%**	**50%**	**219**	**17998**	**16-plex**	**0.89**	**81.2%**	**64%**	**56%**	**50%**	**43%**	**156**	**17744**
BC1	0.94	92.1%	81%	74%	70%	63%	401	19086	BC3	0.91	85.9%	69%	60%	55%	48%	184	16425
BC2	0.91	85.9%	73%	65%	60%	52%	225	18335	*BC4*	*0.73*	*49.7%*	*22%*	*16%*	*12%*	*8%*	*14*	*14199*
BC5	0.92	87.6%	72%	65%	60%	52%	249	18661	BC7	0.89	83.1%	65%	57%	51%	43%	154	16750
BC6	0.90	84.9%	69%	61%	56%	47%	171	17358	BC8	0.89	82.9%	64%	56%	50%	42%	155	17126
BC9	0.88	79.7%	63%	54%	48%	41%	142	16132	BC11	0.90	84.6%	69%	60%	55%	47%	156	18938
BC10	0.90	83.3%	68%	60%	55%	48%	198	17214	BC12	0.90	84.9%	71%	63%	57%	49%	179	20076
BC13	0.90	85.2%	71%	63%	57%	49%	200	18729	BC19	0.91	86.4%	72%	63%	57%	49%	168	18961
BC14	0.90	83.8%	69%	61%	55%	48%	171	18465	BC20	0.95	92.2%	78%	70%	64%	56%	234	19480

This table shows multi-sample enrichment and coverage results for the target regions in BRCA1 and BRCA2. Enrichment was very successful despite the extremely small size of the target regions compared to the genome size (0.003% of the genome), as shown by very high AUC (area under the ROC curve, denoting mapping specificity over 1-sensitivity), percentage of target region bases covered by 1, 10, 20, 30, 50 reads, ADoC (target region base coverages divided by target bases), enrichment factor (EF, using "49.0" reads) and IGV visualization (see Figure S3). The barcode 4 problem (highlighted in italics) which we discovered in the E. coli run was more pronounced in this BRCA1/BRCA2 run. EF for the long high-quality reads ("49.0") ranged between 10,000×-26,000× with the exception of the Chinese non-barcoded control which was "only" enriched 5300× (outlier). The values shown in this table were obtained with SAET 2.2 (read enhancement) and Bioscope 1.0.1 (mapping); for other bioinformatics tools and the 20-plex experiments, see Figure 2 and Tables S4, S22-S24.

Table S2: Bioscope diBayes SNP-calling for Yoruban samples versus our Gold Standard consensus*

HapMap Yoruban from Ibadan NA18507 (Y)	Bioscope: Identical mapping and SNP-calling settings (see Table T1 in http://www.ikmb.uni-kiel.de/tngs-backmapping/bioscope_settings.xls)																																			
	With SNP Backmapping												Without SNP Backmapping																							
	TR mapping / no SAET						TR mapping / SAET						TR mapping / no SAET						TR mapping / SAET						WG mapping / no SAET						WG mapping / SAET					
	SNP concord. rate**	SNP overlap** (Gold)	Non-Gold SNPs total	Non-Gold SNPs annotated	Non-Gold SNPs pot. novel	ADoC at SNPs	SNP concord. rate	SNP overlap (Gold)	Non-Gold SNPs total	Non-Gold SNPs annotated	Non-Gold SNPs pot. novel	ADoC at SNPs	SNP concord. rate	SNP overlap (Gold)	Non-Gold SNPs total	Non-Gold SNPs annotated	Non-Gold SNPs pot. novel	ADoC at SNPs	SNP concord. rate	SNP overlap (Gold)	Non-Gold SNPs total	Non-Gold SNPs annotated	Non-Gold SNPs pot. novel	ADoC at SNPs	SNP concord. rate	SNP overlap (Gold)	Non-Gold SNPs total	Non-Gold SNPs annotated	Non-Gold SNPs pot. novel	ADoC at SNPs	SNP concord. rate	SNP overlap (Gold)	Non-Gold SNPs total	Non-Gold SNPs annotated	Non-Gold SNPs pot. novel	ADoC at SNPs
**No BC - Y**	**100%**	**13 / 15**	**62**	**43**	**19**	**1774**	**100%**	**10 / 15**	**48**	**34**	**14**	**2319**	**100%**	**13 / 15**	**77**	**45**	**32**	**2214**	**100%**	**10 / 15**	**57**	**35**	**22**	**2590**	**100%**	**10 / 15**	**40**	**38**	**2**	**2028**	**100%**	**10 / 15**	**40**	**38**	**2**	**2028**
**4-plex 1**	**96%**	**80%**	**64**	**37**	**27**	**393**	**95%**	**70%**	**56**	**33**	**24**	**513**	**96%**	**80%**	**80**	**40**	**40**	**571**	**95%**	**70%**	**67**	**36**	**32**	**784**	**96%**	**80%**	**33**	**32**	**1.5**	**327**	**95%**	**70%**	**32**	**31**	**1**	**347**
BC5 - Y	92%	12 / 15	63	35	28	439	90%	10 / 15	54	32	22	613	92%	12 / 15	80	39	41	629	90%	10 / 15	65	35	30	923	92%	12 / 15	36	34	2	385	90%	10 / 15	33	32	1	413
BC6 - Y	100%	12 / 15	64	38	26	347	100%	11 / 15	58	33	25	412	100%	12 / 15	79	41	38	512	100%	11 / 15	69	36	33	646	100%	12 / 15	30	29	1	270	100%	11 / 15	30	29	1	282
**4-plex 2**	**86%**	**77%**	**54**	**36**	**18**	**756**	**81%**	**57%**	**45**	**28**	**18**	**863**	**86%**	**77%**	**66**	**38**	**28**	**967**	**81%**	**57%**	**53**	**30**	**24**	**1363**	**87%**	**80%**	**36**	**35**	**1.5**	**607**	**81%**	**60%**	**30**	**29**	**1.5**	**581**
BC9 - Y	80%	10 / 15	51	34	17	646	63%	8 / 15	42	26	16	729	80%	10 / 15	63	37	26	888	63%	8 / 15	50	28	22	1194	82%	11 / 15	34	33	1	548	63%	8 / 15	28	27	1	520
BC10 - Y	92%	13 / 15	56	37	19	867	100%	9 / 15	48	29	19	998	92%	13 / 15	68	38	30	1046	100%	9 / 15	56	31	25	1531	92%	13 / 15	38	36	2	666	100%	10 / 15	32	30	2	642
**8-plex**	**70%**	**55%**	**43**	**23**	**21**	**404**	**68%**	**52%**	**40**	**20**	**20**	**462**	**70%**	**55%**	**55**	**25**	**30**	**483**	**68%**	**52%**	**49**	**22**	**27**	**705**	**71%**	**57%**	**23**	**22**	**1.3**	**364**	**69%**	**53%**	**20**	**19**	**1**	**386**
BC1 - Y	50%	8 / 15	42	24	18	526	50%	8 / 15	37	18	19	588	50%	8 / 15	56	27	29	631	50%	8 / 15	47	20	27	896	56%	9 / 15	23	22	1	497	56%	9 / 15	19	18	1	497
BC2 - Y	86%	7 / 15	39	22	17	406	86%	7 / 15	35	20	15	518	86%	7 / 15	56	26	30	471	86%	7 / 15	45	22	23	742	86%	7 / 15	21	20	1	373	86%	7 / 15	18	18	0	481
BC5 - Y	63%	8 / 15	48	24	24	385	57%	7 / 15	48	21	27	397	63%	8 / 15	56	25	31	423	57%	7 / 15	56	23	33	632	63%	8 / 15	27	25	2	316	57%	7 / 15	23	21	2	273
BC6 - Y	80%	10 / 15	43	20	23	297	78%	9 / 15	40	21	19	345	80%	10 / 15	53	22	31	408	78%	9 / 15	49	23	26	552	80%	10 / 15	20	19	1	269	78%	9 / 15	21	20	1	295
**16-plex**	**80%**	**36%**	**27**	**12**	**15**	**233**	**86%**	**29%**	**25**	**11**	**14**	**268**	**80%**	**36%**	**38**	**14**	**24**	**308**	**86%**	**29%**	**32**	**12**	**20**	**392**	**80%**	**36%**	**10**	**9.8**	**0.3**	**219**	**86%**	**29%**	**9**	**9**	**0.4**	**256**
BC1 - Y	75%	8 / 15	32	16	16	340	60%	5 / 15	25	12	13	414	75%	8 / 15	44	17	27	481	60%	5 / 15	33	13	20	567	75%	8 / 15	13	13	0	265	60%	5 / 15	12	12	0	259
BC2 - Y	100%	4 / 15	34	15	19	243	100%	4 / 15	30	12	18	285	100%	4 / 15	40	15	25	267	100%	4 / 15	36	13	23	361	100%	4 / 15	13	13	0	242	100%	4 / 15	10	9	1	295
BC5 - Y	57%	7 / 15	29	14	15	290	75%	4 / 15	21	11	10	361	57%	7 / 15	38	17	21	395	75%	4 / 15	29	12	17	507	57%	7 / 15	12	12	0	258	75%	4 / 15	9	9	0	336
BC6 - Y	83%	6 / 15	31	11	20	168	100%	6 / 15	30	11	19	185	83%	6 / 15	44	13	31	212	100%	6 / 15	35	12	23	251	83%	6 / 15	9	9	0	113	100%	6 / 15	8	8	0	128
BC9 - Y	67%	3 / 15	24	13	11	156	100%	3 / 15	25	13	12	190	67%	3 / 15	37	15	22	185	100%	3 / 15	36	14	22	259	67%	3 / 15	9	8	1	248	100%	3 / 15	11	10	1	271
BC10 - Y	80%	5 / 15	25	10	15	306	75%	4 / 15	22	11	11	225	80%	5 / 15	34	11	23	351	75%	4 / 15	27	12	15	363	80%	5 / 15	9	8	1	265	75%	4 / 15	10	9	1	246
BC13 - Y	80%	5 / 15	23	10	13	227	75%	4 / 15	24	10	14	314	80%	5 / 15	36	12	24	364	75%	4 / 15	34	12	22	537	80%	5 / 15	9	9	0	214	75%	4 / 15	9	9	0	303
BC14 - Y	100%	5 / 15	18	10	8	134	100%	5 / 15	20	8	12	169	100%	5 / 15	28	12	16	206	100%	5 / 15	26	10	16	289	100%	5 / 15	6	6	0	143	100%	5 / 15	6	6	0	209
**20-plex**	**82%**	**35%**	**27**	**11**	**16**	**62**	**85%**	**32%**	**27**	**10**	**16**	**75**	**82%**	**35%**	**39**	**13**	**26**	**86**	**85%**	**32%**	**38**	**12**	**25**	**119**	**87%**	**35%**	**9**	**8.5**	**0.2**	**32**	**85%**	**31%**	**8**	**8**	**0**	**36**
BC1 - Y	50%	8 / 15	37	15	22	107	43%	7 / 15	36	14	22	138	50%	8 / 15	52	19	33	151	43%	7 / 15	50	17	33	233	50%	8 / 15	13	12	1	58	43%	7 / 15	13	12	1	78
BC2 - Y	100%	7 / 15	31	14	17	73	100%	6 / 15	35	13	22	90	100%	7 / 15	47	18	29	85	100%	6 / 15	50	15	35	104	100%	7 / 15	13	13	0	44	100%	6 / 15	11	11	0	49
BC5 - Y	80%	5 / 15	26	13	13	76	80%	5 / 15	27	11	16	91	80%	5 / 15	38	17	21	114	80%	5 / 15	33	13	20	151	80%	5 / 15	10	10	0	35	80%	5 / 15	9	9	0	33
BC6 - Y	100%	5 / 15	29	12	17	53	100%	5 / 15	25	10	15	45	100%	5 / 15	40	13	27	68	100%	5 / 15	37	13	24	93	100%	5 / 15	9	9	0	20	100%	5 / 15	9	9	0	23
BC9 - Y	100%	3 / 15	25	11	14	50	100%	3 / 15	23	11	12	59	100%	3 / 15	36	13	23	65	100%	3 / 15	33	13	20	81	100%	3 / 15	8	8	0	36	100%	3 / 15	7	7	0	43
BC10 - Y	50%	2 / 15	25	9	16	63	100%	3 / 15	24	8	16	80	50%	2 / 15	35	11	24	87	100%	3 / 15	35	10	25	128	100%	2 / 15	6	6	0	33	100%	2 / 15	6	6	0	33
BC13 - Y	100%	7 / 15	30	13	17	48	100%	6 / 15	25	12	13	46	100%	7 / 15	42	14	28	69	100%	6 / 15	39	14	25	106	100%	7 / 15	12	11	1	26	100%	6 / 15	9	9	0	22
BC14 - Y	67%	6 / 15	18	8	10	40	60%	5 / 15	24	10	14	56	67%	6 / 15	36	9	27	59	60%	5 / 15	37	11	26	78	67%	6 / 15	7	7	0	21	60%	5 / 15	7	7	0	21
BC17 - Y	75%	4 / 15	25	8	17	52	67%	3 / 15	24	9	15	73	75%	4 / 15	32	9	23	82	67%	3 / 15	34	11	23	118	75%	4 / 15	6	6	0	28	67%	3 / 15	8	8	0	31
BC18 - Y	100%	6 / 15	23	6	17	57	100%	5 / 15	23	6	17	66	100%	6 / 15	35	8	27	77	100%	5 / 15	30	7	23	99	100%	6 / 15	3	3	0	21	100%	5 / 15	4	4	0	24

* Gold Standard consensus: consensus of 19 non-reference-base HapMap genotypes [http://www.sanger.ac.uk/humgen/hapmap3/] and 66 non-reference-base whole genome NGS SNPs [ftp://hgdownload.cse.ucsc.edu/goldenPath/hg18/database/pgYh1.txt.gz], [http://solidsoftwaretools.com/gf/project/yoruban/, Yoruban_snp_18x.gff], see Table S19

** Manual inspection using IGV gives 15/15 (100%) SNPs overlap and 14/15 concordant genotypes; see Table S8.

Annotated SNPs are SNPs in dbSNP130

The object of this table is to identify the best approach(es) from: enhanced (SAET 2.2) reads or raw off-machine reads, whole genome (WG) or target region (TR) mapping, SNP-backmapping or not. We observe the expected even distribution of concordance/overlap within samples of the same plex, and the expected deterioration of results for higher plexing. TR mapping of raw reads leads to the highest number of known SNPs, despite lower coverages, and also to the highest number of potential novel SNPs. WG mapping leads to near-zero potential novel SNPs. Backmapping reduces the total number of SNPs by about 15-20% and the number of potential novel SNPs by about a third, eliminating obvious false positives or errors. The average depth of coverage at the SNPs is higher than 100-fold throughout all samples except in the 20-plex pool, where it is higher than 20-fold. To validate the potential novel SNPs we examined inter-sample concordances (Table S20) and manually inspected reads at these positions in IGV (Table S13).

Table S3: Bioscope diBayes SNP-calling for Yoruban samples versus our Silver Standard consensus*

Bioscope: Identical mapping and SNP-calling settings (see Table T1 in http://www.ikmb.uni-kiel.de/tngs-backmapping/bioscope_settings.xls)

With SNP Backmapping

HapMap Yoruban from Ibadan NA18507 (Y)	TR mapping / no SAET						TR mapping / SAET					
	SNP concord. rate	SNP overlap (Silver)	Non-Silver SNPs: total	Non-Silver SNPs: annotated	Non-Silver SNPs: pot. novel	ADoC at SNPs	SNP concord. rate	SNP overlap (Silver)	Non-Silver SNPs: total	Non-Silver SNPs: annotated	Non-Silver SNPs: pot. novel	ADoC at SNPs
**No BC - Y**	100%	56 / 66	19	2	17	1774	100%	45 / 66	13	1	12	2319
**4-plex 1**	92%	67%	31	5	26	393	93%	63%	25	3	22	513
BC5 - Y	91%	44 / 66	31	4	27	439	90%	40 / 66	24	3	21	613
BC6 - Y	93%	45 / 66	31	6	25	347	95%	43 / 66	26	3	23	412
**4-plex 2**	92%	67%	21	4	17	756	94%	53%	19	2	17	863
BC9 - Y	90%	40 / 66	21	5	16	646	91%	33 / 66	17	2	15	729
BC10 - Y	94%	49 / 66	20	3	17	867	97%	37 / 66	20	2	18	998
**8-plex**	81%	43%	23	3	20	404	82%	39%	22	2	20	462
BC1 - Y	83%	29 / 66	21	4	17	526	79%	24 / 66	21	2	19	588
BC2 - Y	77%	26 / 66	20	3	17	406	83%	24 / 66	18	3	15	518
BC5 - Y	77%	30 / 66	26	2	24	385	74%	27 / 66	28	1	27	397
BC6 - Y	86%	28 / 66	25	3	22	297	90%	29 / 66	20	2	18	345
**16-plex**	77%	23%	17	2.8	15	233	82%	20%	16	2	14	268
BC1 - Y	85%	20 / 66	20	4	16	340	80%	15 / 66	15	2	13	414
BC2 - Y	82%	17 / 66	21	2	19	243	86%	14 / 66	20	2	18	285
BC5 - Y	68%	19 / 66	17	2	15	290	86%	14 / 66	11	1	10	361
BC6 - Y	80%	15 / 66	22	2	20	168	86%	14 / 66	22	3	19	185
BC9 - Y	75%	12 / 66	15	4	11	156	85%	13 / 66	15	3	12	190
BC10 - Y	69%	13 / 66	17	2	15	306	69%	13 / 66	13	2	11	225
BC13 - Y	85%	13 / 66	15	2	13	227	75%	12 / 66	16	2	14	314
BC14 - Y	73%	11 / 66	12	4	8	134	91%	11 / 66	14	2	12	169
**20-plex**	74%	21%	18	2.5	16	62	77%	20%	18	2	16	75
BC1 - Y	75%	20 / 66	25	4	21	107	74%	19 / 66	24	3	21	138
BC2 - Y	84%	19 / 66	19	2	17	73	82%	17 / 66	24	2	22	90
BC5 - Y	60%	15 / 66	16	3	13	76	64%	14 / 66	18	2	16	91
BC6 - Y	71%	14 / 66	20	3	17	53	69%	13 / 66	17	2	15	45
BC9 - Y	82%	11 / 66	17	3	14	50	82%	11 / 66	15	3	12	59
BC10 - Y	67%	9 / 66	18	2	16	63	89%	9 / 66	18	2	16	80
BC13 - Y	72%	18 / 66	19	2	17	48	73%	15 / 66	16	3	13	46
BC14 - Y	69%	13 / 66	11	1	10	40	75%	12 / 66	17	3	14	56
BC17 - Y	67%	9 / 66	20	3	17	52	70%	10 / 66	17	2	15	73
BC18 - Y	90%	10 / 66	19	2	17	57	90%	10 / 66	18	1	17	66

Without SNP Backmapping

HapMap Yoruban from Ibadan NA18507 (Y)	TR mapping / no SAET						TR mapping / SAET					
	SNP concord. rate	SNP overlap (Silver)	Non-Silver SNPs: total	Non-Silver SNPs: annotated	Non-Silver SNPs: pot. novel	ADoC at SNPs	SNP concord. rate	SNP overlap (Silver)	Non-Silver SNPs: total	Non-Silver SNPs: annotated	Non-Silver SNPs: pot. novel	ADoC at SNPs
**No BC - Y**	98%	56 / 66	34	4	30	2214	100%	45 / 66	22	2	20	2590
**4-plex 1**	92%	67%	47	8.5	39	571	93%	63%	36	6	30	784
BC5 - Y	91%	44 / 66	48	8	40	629	90%	40 / 66	35	6	29	923
BC6 - Y	93%	45 / 66	46	9	37	512	95%	43 / 66	37	6	31	646
**4-plex 2**	92%	67%	33	6	27	967	94%	53%	27	4	23	1363
BC9 - Y	90%	40 / 66	33	8	25	888	91%	33 / 66	25	4	21	1194
BC10 - Y	94%	49 / 66	32	4	28	1046	97%	37 / 66	28	4	24	1531
**8-plex**	81%	43%	35	5.5	30	483	82%	39%	31	4	27	705
BC1 - Y	83%	29 / 66	35	7	28	631	79%	24 / 66	31	4	27	896
BC2 - Y	77%	26 / 66	37	7	30	471	83%	24 / 66	28	5	23	742
BC5 - Y	77%	30 / 66	34	3	31	423	74%	27 / 66	36	3	33	632
BC6 - Y	86%	28 / 66	35	5	30	408	90%	29 / 66	29	4	25	552
**16-plex**	77%	23%	28	4.4	24	308	82%	20%	23	3	20	392
BC1 - Y	85%	20 / 66	32	5	27	481	80%	15 / 66	23	3	20	567
BC2 - Y	82%	17 / 66	27	2	25	267	86%	14 / 66	26	3	23	361
BC5 - Y	68%	19 / 66	26	5	21	395	86%	14 / 66	19	2	17	507
BC6 - Y	80%	15 / 66	35	4	31	212	86%	14 / 66	27	4	23	251
BC9 - Y	75%	12 / 66	28	6	22	185	85%	13 / 66	26	4	22	259
BC10 - Y	69%	13 / 66	26	3	23	351	69%	13 / 66	18	3	15	363
BC13 - Y	85%	13 / 66	28	4	24	364	75%	12 / 66	26	4	22	537
BC14 - Y	73%	11 / 66	22	6	16	206	91%	11 / 66	20	4	16	289
**20-plex**	74%	21%	31	4.6	26	86	77%	20%	30	4	25	119
BC1 - Y	75%	20 / 66	40	8	32	151	74%	19 / 66	38	6	32	233
BC2 - Y	85%	20 / 66	34	5	29	85	82%	17 / 66	39	4	35	104
BC5 - Y	60%	15 / 66	28	7	21	114	64%	14 / 66	24	4	20	151
BC6 - Y	71%	14 / 66	31	4	27	68	69%	13 / 66	29	5	24	93
BC9 - Y	82%	11 / 66	28	5	23	65	82%	11 / 66	25	5	20	81
BC10 - Y	67%	9 / 66	28	4	24	87	89%	9 / 66	29	4	25	128
BC13 - Y	72%	18 / 66	31	3	28	69	73%	15 / 66	30	5	25	106
BC14 - Y	69%	13 / 66	29	2	27	59	75%	12 / 66	30	4	26	78
BC17 - Y	67%	9 / 66	27	4	23	82	70%	10 / 66	27	4	23	118
BC18 - Y	90%	10 / 66	31	4	27	77	90%	10 / 66	25	2	23	99

HapMap Yoruban from Ibadan NA18507 (Y)	WG mapping / no SAET						WG mapping / SAET					
	SNP concord. rate	SNP overlap (Silver)	Non-Silver SNPs: total	Non-Silver SNPs: annotated	Non-Silver SNPs: pot. novel	ADoC at SNPs	SNP concord. rate	SNP overlap (Silver)	Non-Silver SNPs: total	Non-Silver SNPs: annotated	Non-Silver SNPs: pot. novel	ADoC at SNPs
**No BC - Y**	100%	48 / 66	2	2	0	2028	100%	48 / 66	2	2	0	2028
**4-plex 1**	96%	64%	3	2	0.5	327	94%	61%	2	1.5	0	347
BC5 - Y	91%	45 / 66	3	2	1	385	90%	42 / 66	1	1	0	413
BC6 - Y	100%	40 / 66	2	2	0	270	97%	39 / 66	2	2	0	282
**4-plex 2**	92%	70%	2	2	0	607	94%	57%	2	1.5	0	581
BC9 - Y	90%	42 / 66	3	3	0	548	91%	34 / 66	2	2	0	520
BC10 - Y	94%	50 / 66	1	1	0	666	98%	41 / 66	1	1	0	642
**8-plex**	81%	44%	2	1.5	0.8	364	82%	41%	2	0.8	0.8	386
BC1 - Y	83%	30 / 66	2	2	0	497	81%	26 / 66	2	1	1	497
BC2 - Y	77%	26 / 66	2	1	1	373	83%	24 / 66	1	1	0	481
BC5 - Y	77%	31 / 66	4	2	2	316	75%	28 / 66	2	0	2	273
BC6 - Y	86%	29 / 66	1	1	0	269	90%	29 / 66	1	1	0	295
**16-plex**	76%	21%	1	1	0.3	219	82%	19%	1	1	0.4	256
BC1 - Y	84%	19 / 66	2	2	0	265	81%	16 / 66	1	1	0	259
BC2 - Y	81%	16 / 66	1	1	0	242	92%	12 / 66	2	1	1	295
BC5 - Y	72%	18 / 66	1	1	0	258	85%	13 / 66	0	0	0	336
BC6 - Y	79%	14 / 66	1	1	0	113	85%	13 / 66	1	1	0	128
BC9 - Y	73%	11 / 66	1	0	1	248	83%	12 / 66	2	1	1	271
BC10 - Y	67%	12 / 66	2	1	1	265	67%	12 / 66	2	1	1	246
BC13 - Y	85%	13 / 66	1	1	0	214	75%	12 / 66	1	1	0	303
BC14 - Y	70%	10 / 66	1	1	0	143	90%	10 / 66	1	1	0	209
**20-plex**	75%	20%	1	0.9	0.1	32	74%	18%	1	1	0	36
BC1 - Y	74%	19 / 66	2	2	0	58	72%	18 / 66	2	2	0	78
BC2 - Y	84%	19 / 66	1	1	0	44	81%	16 / 66	1	1	0	49
BC5 - Y	64%	14 / 66	1	1	0	35	62%	13 / 66	1	1	0	33
BC6 - Y	71%	14 / 66	0	0	0	20	62%	13 / 66	1	1	0	23
BC9 - Y	80%	10 / 66	1	1	0	36	80%	10 / 66	0	0	0	43
BC10 - Y	86%	7 / 66	1	1	0	33	86%	7 / 66	1	1	0	33
BC13 - Y	71%	17 / 66	2	1	1	26	71%	14 / 66	1	1	0	22
BC14 - Y	67%	12 / 66	1	1	0	21	73%	11 / 66	1	1	0	21
BC17 - Y	67%	9 / 66	1	1	0	28	60%	10 / 66	1	1	0	31
BC18 - Y	89%	9 / 66	0	0	0	21	89%	9 / 66	0	0	0	24

* Silver Standard consensus: consensus of 66 overlapping non-reference-base whole genome NGS SNPs [ftp://hgdownload.cse.ucsc.edu/goldenPath/hg18/database/pgYh1.txt.gz], [http://solidsoftwaretools.com/gf/project/yoruban/, Yoruban_snp_18x.gff], where a 67th SNP (rs11571671 at chr13:31816222) was discarded because it only occurred in SOLiD (genotype GG).

Annotated SNPs are SNPs in dbSNP130

The object of this table is to validate Table S2 and help identify the best approach(es) from: enhanced (SAET 2.2) reads or raw off-machine reads, whole genome (WG) or target region (TR) mapping, SNP-backmapping or not. This table confirms the Gold Standard results (Table S2): Concordance/overlap within samples of the same plex are evenly distributed, and the results deteriorate as expected for higher plexing. TR mapping of raw reads leads to the highest number of known SNPs, despite lower coverages, and also to the highest number of potential novel SNPs. WG mapping leads to near-zero potential novel SNPs. Backmapping reduces the total number of SNPs by about 15-20% and the number of potential novel SNPs by about a third. The average depth of coverage at the SNPs is higher than 100-fold throughout all samples except in the 20-plex pool, where it is higher than 20-fold. The sequencing error is probably much smaller than the Bioscope diBayes SNP-calling error (see Table S2, footnote ** manual inspection using IGV).

Table S4: Detailed summary for Bioscope 1.0.1 whole genome and target region mapping, all HapMap samples. Whole genome mapping was performed with off-machine reads, target region mapping with SAET 2.2 enhanced reads.

HapMap YRI NA18507 (Y) & CHB NA18561 (C)	Number of barcodes	Genome Size	HybSelected Region	Reads Obtained (raw reads)*	Reads uniquely mapped to genome (incl. 24.2 reads)	49.0 reads uniquely mapped to genome	Mb of coverage	On-Target Reads (unique)	49.0 reads uniquely mapped to target	Mb of target coverage	Percent On-Target Reads	Percent on target 49.0 reads	Percent on target bases	Enrichment Factor	Enrichment Factor (49.0 reads)	Average Depth of Coverage (per barcode)	Average Depth of Coverage (per spot)	1X Consensus Coverage	5X Cons. Cov.	8X Cons. Cov.	10X Cons. Cov.	15X Cons. Cov.	20X Cons. Cov.	30X Cons. Cov.	40X Cons. Cov.	50X Cons. Cov.
4-plex 1	4	3,080,436,051	89,568	28,392,276	16,340,678	5,671,837	744	3,591,810	1,954,519	160	22%	34%	21%	7559.7	11851.6	406.9	1627.6	97.7%	94.6%	92.6%	91.4%	88.8%	86.6%	83.0%	79.8%	76.7%
4-plex 2	4	3,080,436,051	89,568	34,631,217	19,008,476	5,211,856	851	7,077,969	3,851,966	319	37%	74%	38%	12806.2	25418.5	856.4	3425.4	97.2%	94.7%	93.3%	92.4%	90.6%	89.2%	86.6%	84.3%	82.3%
8-plex	8	3,080,436,051	89,568	29,352,928	16,357,189	5,215,004	741	5,543,898	3,030,494	250	34%	58%	34%	11656.4	19985.6	330.5	2644.2	85.8%	79.0%	76.4%	74.8%	71.6%	69.0%	64.5%	60.7%	57.6%
16-plex	16	3,080,436,051	89,568	37,197,621	19,798,860	6,080,436	893	6,296,338	3,215,926	283	32%	53%	32%	10937.2	18189.9	187.5	3000.0	83.3%	73.3%	69.3%	67.2%	62.7%	59.2%	53.9%	49.8%	46.3%
20-plex	20	3,080,436,051	89,568	33,875,493	20,753,516	6,699,709	946	1,717,165	572,708	70	8%	9%	7%	2845.6	2939.9	29.9	598.2	76.5%	57.5%	49.3%	45.2%	37.6%	32.2%	24.8%	19.8%	16.2%
No BC - Y	-	3,080,436,051	89,568	33,216,839	19,465,742	6,050,455	880	6,232,487	3,764,551	285	32%	62%	32%	11011.6	21398.6	0.0	3119.2	99.6%	99.0%	98.7%	98.5%	98.0%	97.6%	96.8%	96.0%	95.4%
No BC - C	-	3,080,436,051	89,568	37,890,578	22,414,101	7,949,682	1,021	2,554,210	1,224,557	110	11%	15%	11%	3919.2	5297.7	0.0	1084.1	99.6%	98.7%	98.0%	97.7%	96.8%	96.0%	94.6%	93.0%	91.6%
**no BC - averaged**	**-**	**3,080,436,051**	**89,568**	**35,553,708**	**20,939,921**	**7,000,068**	**950**	**4,393,348**	**2,494,554**	**197**	**21%**	**36%**	**21%**	**7215.7**	**12256.0**	**0.0**	**2043.6**	**99.7%**	**98.8%**	**98.3%**	**98.1%**	**97.4%**	**96.8%**	**95.7%**	**94.5%**	**93.5%**

	Barcode	Genome Size	HybSelected Region	Reads Obtained (raw reads)*	Reads uniquely mapped to genome (incl. 24.2 reads)	49.0 reads uniquely mapped to genome	Mb of coverage	On-Target Reads (unique)	49.0 reads uniquely mapped to target	Mb of target coverage	Percent On-Target Reads	Percent on target 49.0 reads	Percent on target bases	Enrichment Factor	Enrichment Factor (49.0 reads)	Average Depth of Coverage (per barcode)	Average Depth of Coverage (per spot)	1X Consensus Coverage	5X Cons. Cov.	8X Cons. Cov.	10X Cons. Cov.	15X Cons. Cov.	20X Cons. Cov.	30X Cons. Cov.	40X Cons. Cov.	50X Cons. Cov.
**4-plex 1**	BC5 - Y	3,080,436,051	89,568	9,096,031	5,045,339	1,676,393	228	1,138,964	607,850	50	23%	36%	22%	7763.9	12470.4	515.4		98.2%	95.9%	94.3%	93.5%	91.2%	89.3%	85.9%	83.0%	80.5%
	BC6 - Y	3,080,436,051	89,568	6,611,794	3,912,812	1,379,671	179	872,393	486,241	39	22%	35%	22%	7668.0	12120.9	398.7		97.8%	95.1%	93.3%	92.2%	89.7%	87.5%	83.9%	80.7%	77.6%
	BC7 - C	3,080,436,051	89,568	6,076,429	3,586,261	1,283,820	164	760,764	419,578	34	21%	33%	21%	7295.7	11240.0	344.8		97.5%	93.5%	91.4%	90.0%	87.1%	84.8%	80.9%	77.7%	74.3%
	BC8 - C	3,080,436,051	89,568	6,608,022	3,796,266	1,331,953	173	819,689	440,850	36	22%	33%	21%	7425.9	11383.1	368.6		97.4%	93.6%	91.2%	89.9%	87.1%	84.9%	81.2%	77.7%	74.6%
	**ALL**	**3,080,436,051**	**89,568**	**28,392,276**	**16,340,678**	**5,671,837**	**744**	**3,591,810**	**1,954,519**	**160**	**22%**	**34%**	**21%**	**7559.7**	**11851.6**	**406.9**	**1627.6**	**97.7%**	**94.6%**	**92.6%**	**91.4%**	**88.8%**	**86.6%**	**83.0%**	**79.8%**	**76.7%**
**4-plex 2**	BC7 - C	3,080,436,051	89,568	7,961,278	4,437,986	1,227,092	199	1,705,759	941,887	77	38%	77%	39%	13218.8	26398.6	829.6		97.5%	95.4%	93.9%	92.9%	91.1%	89.5%	86.8%	84.5%	82.6%
	BC8 - C	3,080,436,051	89,568	8,900,065	4,938,100	1,349,598	221	1,791,048	976,815	81	36%	72%	36%	12474.0	24892.4	865.4		97.5%	95.1%	93.6%	92.8%	91.1%	89.9%	87.0%	84.8%	82.7%
	BC9 - Y	3,080,436,051	89,568	8,028,156	4,350,560	1,191,386	195	1,586,804	850,981	71	36%	71%	37%	12544.0	24565.6	765.3		97.0%	93.9%	92.2%	91.4%	89.3%	87.9%	85.3%	83.0%	80.8%
	BC10 - Y	3,080,436,051	89,568	9,741,718	5,281,830	1,443,780	236	1,994,358	1,082,283	90	38%	75%	38%	12986.1	25781.0	965.1		96.8%	94.5%	93.5%	92.7%	91.0%	89.5%	87.1%	84.9%	83.1%
	**ALL**	**3,080,436,051**	**89,568**	**34,631,217**	**19,008,476**	**5,211,856**	**851**	**7,077,969**	**3,851,966**	**319**	**37%**	**74%**	**38%**	**12806.2**	**25418.5**	**856.4**	**3425.4**	**97.2%**	**94.7%**	**93.3%**	**92.4%**	**90.6%**	**89.2%**	**86.6%**	**84.3%**	**82.3%**
**8-plex**	BC1 - Y	3,080,436,051	89,568	6,004,455	3,327,875	1,035,687	150	1,170,691	645,168	53	35%	62%	35%	12098.6	21424.2	560.9		92.3%	88.6%	86.9%	85.8%	83.4%	81.4%	77.8%	74.4%	71.6%
	BC2 - Y	3,080,436,051	89,568	4,008,564	2,327,933	754,952	106	834,081	472,752	38	36%	63%	36%	12322.5	21536.4	402.7		90.6%	86.4%	84.2%	82.7%	79.8%	77.1%	73.0%	69.3%	66.2%
	BC3 - C	3,080,436,051	89,568	4,114,328	2,237,043	715,287	101	696,228	380,833	31	31%	53%	31%	10703.8	18311.1	332.6		89.1%	83.2%	80.4%	78.9%	75.4%	72.8%	68.4%	64.3%	61.1%
	BC4 - C	3,080,436,051	89,568	290,433	162,453	55,667	7	52,846	25,828	2	33%	46%	32%	11187.8	15957.0	24.7		57.1%	37.9%	34.3%	32.3%	28.0%	25.4%	21.1%	17.6%	14.7%
	BC5 - Y	3,080,436,051	89,568	4,880,335	2,616,438	795,955	118	888,915	470,166	40	34%	59%	34%	11684.5	20315.2	420.6		90.8%	86.6%	84.3%	82.9%	80.2%	77.7%	73.3%	69.5%	66.1%
	BC6 - Y	3,080,436,051	89,568	3,626,078	2,109,171	695,238	96	710,705	396,116	32	34%	57%	33%	11588.8	19595.1	339.4		90.2%	85.5%	82.5%	80.7%	77.8%	75.1%	70.5%	66.6%	63.4%
	BC7 - C	3,080,436,051	89,568	3,052,954	1,727,544	568,319	79	576,042	314,370	26	33%	55%	33%	11467.9	19024.3	273.1		88.4%	82.5%	79.4%	77.9%	74.4%	71.2%	66.4%	62.0%	58.6%
	BC8 - C	3,080,436,051	89,568	3,375,781	1,848,732	593,899	84	614,390	325,261	28	33%	55%	33%	11429.6	18835.6	290.3		87.8%	81.4%	78.8%	77.4%	74.1%	70.9%	65.9%	62.2%	59.0%
	**ALL**	**3,080,436,051**	**89,568**	**29,352,928**	**16,357,189**	**5,215,004**	**741**	**5,543,898**	**3,030,494**	**250**	**34%**	**58%**	**34%**	**11656.4**	**19985.6**	**330.5**	**2644.2**	**85.8%**	**79.0%**	**76.4%**	**74.8%**	**71.6%**	**69.0%**	**64.5%**	**60.7%**	**57.6%**
**16-plex**	BC1 - Y	3,080,436,051	89,568	4,899,027	2,591,032	776,023	117	840,229	430,658	38	32%	55%	32%	11152.8	19086.1	401.0		92.1%	85.5%	82.5%	80.9%	77.1%	74.2%	69.7%	66.1%	63.2%
	BC2 - Y	3,080,436,051	89,568	2,676,886	1,469,076	452,853	66	469,776	241,429	21	32%	53%	32%	10997.8	18335.4	224.5		85.9%	78.2%	75.1%	72.8%	68.6%	65.0%	59.8%	55.8%	52.2%
	BC3 - C	3,080,436,051	89,568	2,668,379	1,333,918	398,232	60	389,808	190,187	17	29%	48%	29%	10050.3	16424.9	183.5		85.9%	75.5%	71.5%	68.9%	64.2%	60.4%	55.2%	51.1%	47.6%
	BC4 - C	3,080,436,051	89,568	192,251	101,453	33,820	5	30,890	13,963	1	30%	41%	30%	10471.6	14199.2	14.4		49.7%	28.5%	23.7%	21.8%	18.2%	15.8%	12.4%	9.8%	8.0%
	BC5 - Y	3,080,436,051	89,568	3,195,035	1,639,682	483,018	74	526,516	262,087	24	32%	54%	32%	11043.6	18661.3	249.1		87.6%	78.5%	74.4%	72.4%	67.7%	64.5%	59.6%	55.9%	52.4%
	BC6 - Y	3,080,436,051	89,568	2,137,151	1,169,254	368,531	53	359,581	186,004	16	31%	50%	31%	10576.6	17358.3	170.8		84.9%	75.7%	71.7%	69.4%	64.8%	61.2%	55.6%	51.1%	47.2%
	BC7 - C	3,080,436,051	89,568	2,046,478	1,062,486	331,351	48	327,672	161,373	15	31%	49%	31%	10606.6	16749.5	154.1		83.1%	71.8%	67.4%	65.3%	60.8%	56.8%	51.3%	47.1%	43.0%
	BC8 - C	3,080,436,051	89,568	2,009,518	1,054,989	329,533	48	329,037	164,096	15	31%	50%	31%	10726.5	17126.1	155.2		82.9%	71.2%	66.5%	64.3%	59.6%	56.1%	50.2%	46.0%	42.1%
	BC9 - Y	3,080,436,051	89,568	1,880,236	998,390	315,735	45	302,505	148,103	14	30%	47%	30%	10420.6	16132.5	141.9		79.7%	68.5%	64.8%	62.6%	57.5%	53.7%	48.5%	44.5%	41.0%
	BC10 - Y	3,080,436,051	89,568	2,779,455	1,360,684	407,227	61	422,948	203,820	19	31%	50%	31%	10690.3	17213.5	197.6		83.3%	73.5%	69.9%	67.9%	63.5%	60.1%	55.1%	51.1%	47.5%
	BC11 - C	3,080,436,051	89,568	1,711,792	956,872	307,332	43	322,884	169,234	15	34%	55%	34%	11605.2	18938.2	155.6		84.6%	75.9%	71.4%	69.4%	64.1%	60.5%	54.6%	50.2%	46.5%
	BC12 - C	3,080,436,051	89,568	1,943,943	1,103,327	332,318	50	368,324	193,990	17	33%	58%	33%	11481.1	20076.4	179.3		84.9%	76.8%	73.0%	71.0%	66.2%	62.7%	56.8%	52.6%	48.7%
	BC13 - Y	3,080,436,051	89,568	2,389,711	1,265,241	393,244	57	420,863	214,148	19	33%	54%	33%	11440.0	18728.9	200.3		85.2%	76.9%	72.8%	70.7%	66.4%	63.3%	57.5%	52.8%	49.5%
	BC14 - Y	3,080,436,051	89,568	1,979,651	1,101,275	347,642	50	355,431	186,651	16	32%	54%	32%	11099.9	18465.3	170.6		83.8%	74.7%	70.9%	68.6%	64.4%	60.6%	55.5%	50.9%	47.5%
	BC19 - C	3,080,436,051	89,568	2,009,942	1,086,007	329,468	49	346,545	181,639	16	32%	55%	32%	10974.5	18960.7	167.7		86.4%	77.6%	73.3%	71.5%	66.8%	63.1%	57.1%	52.4%	48.9%
	BC20 - C	3,080,436,051	89,568	2,678,166	1,505,174	474,109	68	483,329	268,544	22	32%	57%	32%	11043.7	19480.3	234.3		92.2%	84.2%	80.0%	77.8%	73.7%	69.7%	63.9%	59.5%	56.0%
	**ALL**	**3,080,436,051**	**89,568**	**37,197,621**	**19,798,860**	**6,080,436**	**893**	**6,296,338**	**3,215,926**	**283**	**32%**	**53%**	**32%**	**10937.2**	**18189.9**	**187.5**	**3000.0**	**83.3%**	**73.3%**	**69.3%**	**67.2%**	**62.7%**	**59.2%**	**53.9%**	**49.8%**	**46.3%**
**20-plex**	BC1 - Y	3,080,436,051	89,568	4,126,955	2,453,376	763,979	111	202,306	64,407	8	8%	8%	7%	2836.0	2899.4	70.0		87.8%	75.4%	68.7%	65.1%	57.2%	51.5%	43.1%	37.4%	32.6%
	BC2 - Y	3,080,436,051	89,568	1,991,692	1,250,868	405,698	57	108,983	39,141	5	9%	10%	8%	2996.4	3318.1	39.5		81.1%	65.4%	57.7%	53.5%	45.4%	39.6%	31.5%	25.7%	21.4%
	BC3 - C	3,080,436,051	89,568	1,561,461	933,948	300,853	42	74,202	23,432	3	8%	8%	7%	2732.5	2678.6	25.7		76.0%	56.3%	48.5%	44.3%	36.5%	30.7%	22.9%	17.8%	14.3%
	BC4 - C	3,080,436,051	89,568	131,453	76,352	25,250	3	6,728	1,641	0	9%	6%	8%	3030.6	2235.1	2.1		32.6%	11.4%	7.3%	6.0%	3.9%	2.7%	1.2%	0.7%	0.4%
	BC5 - Y	3,080,436,051	89,568	2,139,831	1,260,586	389,943	57	105,030	32,620	4	8%	8%	7%	2865.5	2877.0	35.4		80.4%	63.0%	54.6%	49.8%	42.0%	36.2%	28.7%	23.5%	19.6%
	BC6 - Y	3,080,436,051	89,568	1,508,733	948,873	314,672	43	75,966	25,361	3	8%	8%	7%	2753.4	2771.8	26.2		78.2%	58.0%	48.9%	44.1%	35.5%	30.1%	22.9%	18.2%	14.7%
	BC7 - C	3,080,436,051	89,568	1,292,026	779,109	253,107	35	67,284	21,777	3	9%	9%	8%	2970.1	2959.1	22.9		74.8%	52.8%	44.7%	40.9%	33.6%	28.1%	20.5%	15.7%	12.2%
	BC8 - C	3,080,436,051	89,568	1,268,419	768,829	253,869	35	65,679	21,343	3	9%	8%	8%	2938.0	2891.4	22.4		72.2%	52.5%	44.2%	40.5%	32.6%	26.8%	20.0%	16.1%	12.4%
	BC9 - Y	3,080,436,051	89,568	1,447,438	874,628	288,115	40	69,109	19,972	3	8%	7%	7%	2717.5	2384.0	22.4		73.8%	52.8%	44.0%	39.4%	31.6%	26.5%	19.6%	15.2%	12.2%
	BC10 - Y	3,080,436,051	89,568	1,879,568	1,105,076	360,499	50	88,136	26,175	4	8%	7%	7%	2743.0	2497.1	28.5		75.8%	56.6%	48.2%	44.2%	36.8%	31.5%	24.3%	19.3%	16.0%
	BC11 - C	3,080,436,051	89,568	1,130,415	713,192	239,842	33	63,760	24,134	3	9%	10%	8%	3074.7	3460.7	23.4		76.2%	56.0%	46.9%	42.6%	35.3%	29.6%	22.2%	16.8%	12.9%
	BC12 - C	3,080,436,051	89,568	1,423,950	900,471	280,065	41	77,141	28,697	3	9%	10%	8%	2946.3	3524.0	28.9		80.3%	61.2%	52.5%	48.1%	40.3%	34.3%	26.1%	20.3%	16.1%
	BC13 - Y	3,080,436,051	89,568	1,619,611	1,014,058	335,415	46	85,387	28,525	4	8%	9%	8%	2895.9	2924.8	29.8		78.7%	58.9%	50.3%	46.0%	38.3%	32.9%	25.1%	20.1%	17.0%
	BC14 - Y	3,080,436,051	89,568	1,374,871	873,743	290,001	40	76,077	26,945	3	9%	9%	8%	2994.5	3195.5	27.0		76.6%	58.3%	49.5%	45.4%	38.0%	32.2%	23.8%	18.4%	14.6%
	BC15 - C	3,080,436,051	89,568	2,021,309	1,215,553	382,056	55	97,836	31,254	4	8%	8%	7%	2768.1	2813.4	33.9		80.1%	61.5%	53.7%	49.5%	41.6%	36.4%	28.0%	22.3%	18.5%
	BC16 - C	3,080,436,051	89,568	2,211,808	1,331,904	429,764	61	113,270	37,770	5	9%	9%	8%	2924.8	3022.6	39.5		82.5%	64.8%	56.5%	52.2%	44.6%	39.0%	30.7%	24.9%	21.0%
	BC17 - Y	3,080,436,051	89,568	1,911,811	1,152,099	362,882	52	94,006	30,619	4	8%	8%	7%	2806.2	2901.9	32.0		79.2%	60.5%	51.6%	47.6%	39.8%	34.3%	26.7%	21.5%	17.8%
	BC18 - Y	3,080,436,051	89,568	1,594,490	997,706	333,102	46	81,370	27,402	3	8%	8%	7%	2804.9	2829.2	28.1		79.2%	59.1%	50.6%	45.6%	37.0%	31.5%	24.4%	19.6%	15.8%
	BC19 - C	3,080,436,051	89,568	1,413,120	889,009	281,439	40	69,821	25,264	3	8%	9%	7%	2701.1	3087.3	25.9		79.9%	59.4%	51.0%	46.8%	38.8%	32.8%	24.3%	18.6%	14.4%
	BC20 - C	3,080,436,051	89,568	1,826,532	1,214,136	409,158	56	95,074	36,229	4	8%	9%	7%	2693.1	3045.3	34.5		84.9%	66.0%	57.5%	52.8%	43.8%	37.6%	29.5%	24.1%	20.0%
	**ALL**	**3,080,436,051**	**89,568**	**33,875,493**	**20,753,516**	**6,699,709**	**946**	**1,717,165**	**572,708**	**70**	**8%**	**9%**	**7%**	**2845.6**	**2939.9**	**29.9**	**598.2**	**76.5%**	**57.5%**	**49.3%**	**45.2%**	**37.6%**	**32.2%**	**24.8%**	**19.8%**	**16.2%**
**No BC - Y**	**-**	**3,080,436,051**	**89,568**	**33,216,839**	**19,465,742**	**6,050,455**	**880**	**6,232,487**	**3,764,551**	**285**	**32%**	**62%**	**32%**	**11011.6**	**21398.6**		**3119.2**	**99.6%**	**99.0%**	**98.7%**	**98.5%**	**98.0%**	**97.6%**	**96.8%**	**96.0%**	**95.4%**
**No BC - C**	**-**	**3,080,436,051**	**89,568**	**37,890,578**	**22,414,101**	**7,949,682**	**1,021**	**2,554,210**	**1,224,557**	**110**	**11%**	**15%**	**11%**	**3919.2**	**5297.7**		**1084.1**	**99.6%**	**98.7%**	**98.0%**	**97.7%**	**96.8%**	**96.0%**	**94.6%**	**93.0%**	**91.6%**
**No BC - Y & C**	**Average**	**3,080,436,051**	**89,568**	**35,553,708**	**20,939,921**	**7,000,068**	**950**	**4,393,348**	**2,494,554**	**197**	**21%**	**36%**	**21%**	**7215.7**	**12256.0**		**2043.6**	**99.7%**	**98.8%**	**98.3%**	**98.1%**	**97.4%**	**96.8%**	**95.7%**	**94.5%**	**93.5%**

* herein identical to reads obtained off the SOLiD 3 machine, i.e. without QualityValue-filtering (this filtering is carried out during tertiary analyses, e.g. mapping/pairing/SNP-calling)

24.2 reads are read segments of length 24 which could be mapped with 2 mismatches; this is the shortest allowed read segment in the default mapping settings (see Table T1 in http://www.ikmb.uni-kiel.de/tngs-backmapping/bioscope_settings.xls)

49.0 reads are read segments of length 49 which could be mapped with 0 mismatches; this is the longest possible read segment and the best possible match

This table shows in detail the effect of SAET 2.2 (SOLiD Software Spectral Analysis Enhancement Tool) on read mapping with Bioscope. Comparison with Table S22 (no SAET 2.2), shows that SAET 2.2 improved coverages by 15%. Notably, SAET 2.2 doubled(!) the mapping rate of long segments (49.0 reads) to the target region.

Table S5 Table for SNP summary Figure 3

		For Summary Figure								Just for calculating numbers											
		Gold (incl. silver) concordant genotypes	Gold (incl. silver) discordant genotypes	Gold (incl. silver) FN SNPs	Silver concordant genotypes	Silver discordant genotypes	Silver FN SNPs	Intra-plex concordant likely FP SNPs	Likely FP SNPs	Total SNPs	Pot. novel SNPs	In silico val. novel SNPs (inter-sample)	Upper bound diBayes FP SNPs (From s3)	In silico val. novel SNPs (viewer, S12)	Upper bound FP SNPs after manual inspection	GOLD concordant SNPs	GOLD overlap	GOLD discordant SNPs	SILVER concordant SNPs	SILVER overlap	SILVER discordant SNPs
Bioscope, TR, BM	no barcode	13	0	2	43	0	8	**0**	**19**	75	19	**NA**	**NA**		NA						
	4-plex	10.25	1.50	3.25	31	2	18	15	7.5	70.25	22.5	15	13		NA	41	47	6	164	178	14
	8-plex	5.75	2.50	6.75	17	3	31	10	10.5	51.25	20.5	10	14		NA	23	33	10	91	113	22
	16-plex	4.25	1.13	9.63	7	2	41	4	10.6	32.375	14.625	4	16		NA	34	43	9	93	120	27
	20-plex	4.40	0.90	9.70	6	3	43	8	8.0	32.2	16	8	14		NA	44	53	9	102	138	36
Bioscope, TR, SAET, BM	no barcode	10	0	5	35	0	16	**0**	**14**	58	14	**NA**	**NA**		NA						
	4-plex	8.50	1.00	5.50	27	2	22	11	9.5	60	20.5	11	14		NA	34	38	4	143	153	10
	8-plex	5.25	2.50	7.25	16	2	33	9	11.0	47.75	20	9	18		NA	21	31	10	85	104	19
	16-plex	3.75	0.63	10.63	7	2	42	3	10.6	29	13.625	3	16		NA	30	35	5	87	106	19
	20-plex	4.00	0.80	10.20	6	2	43	8	8.2	31.4	16	8	6		NA	40	48	8	99	130	31
Bioscope, WG	no barcode	10	0	5	38	0	13	**0**	**0**	50	0	0	0		NA						
	4-plex	11.00	1.00	3.00	31	2	19	0	1.5	46.5	1.5	0	1		NA	44	48	4	166	177	11
	8-plex	6.00	2.50	6.50	18	3	31	0	1.3	31.25	1.25	0	2		NA	24	34	10	94	116	22
	16-plex	4.25	1.13	9.63	7	2	42	0	0.3	15.375	0.25	0	1		NA	34	43	9	87	113	26
	20-plex	4.50	0.80	9.70	5	3	43	0	0.1	14	0.1	0	1		NA	45	53	8	97	130	33
Bioscope, SAET, WG	no barcode	10	0	5	38	0	13	**0**	**0**	50	0	0	0		NA						
	4-plex	8.75	1.00	5.25	28	1	22	0	1.3	40.5	1.25	0	0		NA	35	39	4	147	156	9
	8-plex	5.50	2.50	7.00	17	2	32	0	1.0	28.25	1	0	2		NA	22	32	10	88	107	19
	16-plex	3.75	0.63	10.63	7	2	43	0	0.4	13.75	0.375	0	2		NA	30	35	5	82	100	18
	20-plex	3.90	0.80	10.30	5	2	44	0	0.1	13	0	0	1		NA	39	47	8	88	121	33
Bioscope + IGV, TR+BM	no barcode	14	1	0	46	5	**0**	**0**	**19**	75	19	**NA**	**NA**	1	19						
	no bc, SAET	14	1	0	46	5	**0**	**0**	**14**	58	14	**NA**	**NA**		NA						
	4-bc5, SAET	14	1	0	37	11	3	5	5	64	22	11	11	5	5	4-plex1bc5, SAET					
Bioscope + IGV, WG	no barcode	14	1	0	47	4	**0**	**0**	**0**	50	0	**2**	**0**		NA						
	no bc, SAET	14	1	0	47	4	**0**	**0**	**0**	50	0	**2**	**0**		NA						
CLC Bio	CLC: 4-plex1bc5	93.3%			93.3%			NA	NA	NA	NA	NA	NA		NA						
NextGENe	NG: 4-plex1bc5	12	0	3.00	1			NA	NA	NA	NA	NA	NA		NA						
	HapMap Yoruban from Ibadan NA18507 (Y)	(from S3)			(from S3)					(S3)	(S3)	(S12)	(S3)								

Table S6: Initial corona lite / stand-alone diBayes SNP-caller results comparison against HapMap3*

HapMap Yoruban from Ibadan NA18507 (Y) and Han Chinese from Beijing NA18561 (C)	corona lite mapping, early access diBayes SNP-calling								
	Mapping with 4 MM			Mapping with 5 mismatches (MM)					
	TR / no SAET			TR / SAET***			WG / no SAET		
	SNP concord. rate	SNP overlap (HapMap)	Non-HapMap SNPs**	SNP concord. rate	SNP overlap (HapMap)	Non-HapMap SNPs	SNP concord. rate	SNP overlap (HapMap)	Non-HapMap SNPs
No BC - Y				100%	11 / 15	218	100%	8 / 15	9
No BC - C				100%	16 / 28	376	100%	1 / 28	7
**no BC - mean**				**100%**	**63%**	**297**	**100%**	**21%**	**8**
**4-plex 1**	**71%**	**93%**	**217**	**71%**	**93%**	**217**	**100%**	**53%**	**8**
BC5 - Y	71%	14 / 15	217	71%	14 / 15	217	100%	8 / 15	8

* HapMap3: 15 non-reference-base NA18507 SNPs and 28 non-reference-base NA18561 SNPs [http://www.sanger.ac.uk/humgen/hapmap3/]

** dbSNP130 lists 1011 known SNP positions for our 118822 base BRCA1/2 target region. For the Yoruban and Chinese samples, many of these SNP positions are expected to be reference bases, i.e. without a SNP-call.

*** SAET 2.2 (SOLiD software tools; Spectral Analysis Enhancement Tool)

In this table we summarise seven of our initial SNP-calling results based on corona lite v4.0r2.0 mapping, using the stand-alone early-access diBayes 1.1.1 tool, both of which we adapted to work on our PBS Pro cluster (corona lite is meant for Torque clusters, diBayes 1.1.1 is meant for on-machine use). The results were unpromising, esp. for reads which were mapped to the whole genome. The best results (shown here) were obtained for the diBayes setting "call.stringency=default". We abandoned this early access diBayes tool, because the new Bioscope 1.0.1 diBayes allows additional settings especially for enriched samples (see Table T1 in http://www.ikmb.uni-kiel.de/tngs-backmapping/bioscope_settings.xls).

Table S7: diBayes SNP-calling for Yoruban Samples versus our Gold Standard* and Chinese samples versus HapMap Bronze**

HapMap Yoruban from Ibadan NA18507 (Y) and Han Chinese from Beijing NA18561 (C)	Bioscope: Identical mapping and SNP-calling settings (see Table T1 in http://www.ikmb.uni-kiel.de/tngs-backmapping/bioscope_settings.xls)																																				Bioscope (settings: Table T22)			CLC bio 3.7.1 defaults		nextGENe V2 defaults	
	With SNP Backmapping												Without SNP Backmapping																														
	TR mapping / no SAET						TR mapping / SAET						TR mapping / no SAET						TR mapping / SAET						WG mapping / no SAET						WG mapping / SAET						TR mapping / SAET			TR mapping		WG mapping	
	SNP concord. rate	SNP overlap (HapMap Bronze / Gold)	Non-HapMap SNPs total	annotated	novel	ADoC at SNPs	SNP concord. rate	SNP overlap (HapMap Bronze / Gold)	Non-HapMap SNPs total	annotated	novel	ADoC at SNPs	SNP concord. rate	SNP overlap (HapMap Bronze / Gold)	Non-HapMap SNPs total	annotated	novel	ADoC at SNPs	SNP concord. rate	SNP overlap (HapMap Bronze / Gold)	Non-HapMap SNPs total	annotated	novel	ADoC at SNPs	SNP concord. rate	SNP overlap (HapMap Bronze / Gold)	Non-HapMap SNPs total	annotated	novel	ADoC at SNPs	SNP concord. rate	SNP overlap (HapMap Bronze / Gold)	Non-HapMap SNPs total	annotated	novel	ADoC at SNPs	SNP concord. rate	SNP overlap (HapMap Bronze / Gold)	Non-HapMap SNPs (total)	SNP concord. rate	SNP overlap (Gold)	SNP concord. rate	SNP overlap (Gold)
No BC - Y	100%	13 / 15	62	43	19	1774	100%	10 / 15	48	34	14	2319	100%	13 / 15	77	45	32	2214	100%	10 / 15	57	35	22	2590	100%	10 / 15	40	38	2	2028	100%	10 / 15	40	38	2	2028	100%	11 / 15	238				
No BC - C	100%	14 / 28	57	24	33	667	100%	12 / 28	42	24	18	887	100%	14 / 28	73	26	47	970	100%	12 / 28	54	26	28	1316	100%	14 / 28	28	27	1	473	100%	14 / 28	28	27	1	473	100%	15 / 28	278				
**no BC - mean**	**100%**	**63%**	**60**	**34**	**26**	**1220**	**100%**	**51%**	**45**	**29**	**16**	**1603**	**100%**	**63%**	**75**	**36**	**40**	**1592**	**100%**	**51%**	**56**	**31**	**25**	**1953**	**100%**	**56%**	**34**	**33**	**2**	**1250**	**100%**	**56%**	**34**	**33**	**2**	**1250**	**100%**	**60%**	**258**				
**4-plex 1**	**98%**	**55%**	**60**	**31**	**28**	**355**	**98%**	**49%**	**51**	**28**	**24**	**455**	**98%**	**55%**	**76**	**35**	**42**	**516**	**98%**	**49%**	**63**	**31**	**32**	**716**	**98%**	**55%**	**29**	**28**	**1**	**263**	**98%**	**49%**	**27**	**26**	**1**	**271**	**98%**	**56%**	**267**	**87%**	**100%**	**100%**	**80%**
BC5 - Y	92%	12 / 15	63	35	28	439	90%	10 / 15	54	32	22	613	92%	12 / 15	80	39	41	629	90%	10 / 15	65	35	30	923	92%	12 / 15	36	34	2	385	90%	10 / 15	33	32	1	413	92%	13 / 15	264	86.7%	15 / 15	100%	12 / 15
BC6 - Y	100%	12 / 15	64	38	26	347	100%	11 / 15	58	33	25	412	100%	12 / 15	79	41	38	512	100%	11 / 15	69	36	33	646	100%	12 / 15	30	29	1	270	100%	11 / 15	30	29	1	282	100%	13 / 15	267				
BC7 - C	100%	12 / 28	61	25	36	283	100%	12 / 28	44	21	23	404	100%	12 / 28	76	27	49	405	100%	12 / 28	56	24	32	641	100%	12 / 28	22	21	1	172	100%	12 / 28	20	19	1	176	100%	12 / 28	267				
BC8 - C	100%	11 / 28	50	27	23	349	100%	9 / 28	49	25	24	390	100%	11 / 28	70	31	39	519	100%	9 / 28	61	27	34	656	100%	11 / 28	28	27	1	224	100%	9 / 28	26	25	1	213	100%	10 / 28	269				
**4-plex 2**	**91%**	**51%**	**49**	**30**	**19**	**655**	**88%**	**40%**	**41**	**23**	**17**	**825**	**91%**	**51%**	**61**	**32**	**29**	**921**	**88%**	**40%**	**49**	**25**	**24**	**1362**	**91%**	**52%**	**30**	**29**	**1**	**520**	**88%**	**41%**	**25**	**24**	**1**	**533**	**89%**	**55%**	**209**				
BC7 - C	100%	11 / 28	45	24	21	529	100%	8 / 28	32	17	15	735	100%	11 / 28	56	27	29	896	100%	8 / 28	42	19	23	1327	100%	11 / 28	23	22	1	415	100%	8 / 28	16	15	1	462	100%	11 / 28	191				
BC8 - C	90%	10 / 28	44	24	20	579	89%	9 / 28	40	21	19	839	90%	10 / 28	58	27	31	854	89%	9 / 28	48	23	25	1397	90%	10 / 28	26	25	1	450	89%	9 / 28	23	22	1	508	90%	10 / 28	208				
BC9 - Y	80%	10 / 15	51	34	17	646	63%	8 / 15	42	26	16	729	80%	10 / 15	63	37	26	888	63%	8 / 15	50	28	22	1194	82%	11 / 15	34	33	1	548	63%	8 / 15	28	27	1	520	75%	12 / 15	207				
BC10 - Y	92%	13 / 15	56	37	19	867	100%	9 / 15	48	29	19	998	92%	13 / 15	68	38	30	1046	100%	9 / 15	56	31	25	1531	92%	13 / 15	38	36	2	666	100%	10 / 15	32	30	2	642	93%	14 / 15	228				
**8-plex ex bc4**	**77%**	**31%**	**38**	**18**	**20**	**374**	**79%**	**30%**	**34**	**16**	**18**	**427**	**77%**	**31%**	**50**	**20**	**29**	**465**	**79%**	**30%**	**43**	**18**	**25**	**650**	**78%**	**32%**	**18**	**17**	**1**	**301**	**79%**	**31%**	**16**	**16**	**1**	**330**	**80%**	**42%**	**230**				
**8-plex**	**67%**	**27%**	**34**	**17**	**18**	**337**	**69%**	**26%**	**31**	**15**	**16**	**384**	**67%**	**27%**	**45**	**19**	**27**	**418**	**69%**	**26%**	**39**	**16**	**23**	**580**	**68%**	**27%**	**16**	**15**	**1**	**273**	**70%**	**26%**	**15**	**14**	**1**	**299**	**70%**	**36%**	**210**				
BC1 - Y	50%	8 / 15	42	24	18	526	50%	8 / 15	37	18	19	588	50%	8 / 15	56	27	29	631	50%	8 / 15	47	20	27	896	56%	9 / 15	23	22	1	497	56%	9 / 15	19	18	1	497	64%	11 / 15	234				
BC2 - Y	86%	7 / 15	39	22	17	406	86%	7 / 15	35	20	15	518	86%	7 / 15	56	26	30	471	86%	7 / 15	45	22	23	742	86%	7 / 15	21	20	1	373	86%	7 / 15	18	18	0	481	89%	9 / 15	244				
BC3 - C	100%	3 / 28	25	12	13	343	100%	4 / 28	20	12	8	368	100%	3 / 28	40	14	26	447	100%	4 / 28	28	14	14	581	100%	3 / 28	11	11	0	267	100%	4 / 28	11	11	0	234	86%	7 / 28	213				
BC4 - C	0%	1 / 28	11	7	4	76	0%	1 / 28	7	3	4	76	0%	1 / 28	16	8	8	86	0%	1 / 28	10	3	7	96	0%	1 / 28	4	4	0	72	0%	1 / 28	1	1	0	80	0%	1 / 28	71				
BC5 - Y	63%	8 / 15	48	24	24	385	57%	7 / 15	48	21	27	397	63%	8 / 15	56	25	31	423	57%	7 / 15	56	23	33	632	63%	8 / 15	27	25	2	316	57%	7 / 15	23	21	2	273	63%	8 / 15	231				
BC6 - Y	80%	10 / 15	43	20	23	297	78%	9 / 15	40	21	19	345	80%	10 / 15	53	22	31	408	78%	9 / 15	49	23	26	552	80%	10 / 15	20	19	1	269	78%	9 / 15	21	20	1	295	92%	12 / 15	253				
BC7 - C	100%	4 / 28	32	12	20	356	100%	3 / 28	31	12	19	393	100%	4 / 28	40	14	26	457	100%	3 / 28	39	14	25	557	100%	4 / 28	10	10	0	173	100%	3 / 28	11	11	0	238	89%	9 / 28	229				
BC8 - C	60%	5 / 28	35	13	22	306	80%	5 / 28	30	10	20	383	60%	5 / 28	46	15	31	418	80%	5 / 28	38	12	26	587	60%	5 / 28	11	10	1	213	80%	5 / 28	12	11	1	291	80%	5 / 28	205				
**16-plex ex bc4**	**80%**	**20%**	**28**	**12**	**16**	**224**	**76%**	**18%**	**25**	**10**	**14**	**266**	**80%**	**20%**	**38**	**13**	**24**	**298**	**76%**	**18%**	**31**	**11**	**20**	**383**	**80%**	**20%**	**9**	**9**	**0**	**196**	**76%**	**18%**	**8**	**8**	**0**	**219**	**81%**	**24%**	**194**				
**16-plex**	**75%**	**19%**	**23**	**11**	**13**	**197**	**71%**	**16%**	**23**	**10**	**13**	**231**	**75%**	**19%**	**33**	**12**	**21**	**262**	**71%**	**16%**	**30**	**11**	**19**	**344**	**75%**	**19%**	**8**	**8**	**0**	**181**	**71%**	**16%**	**8**	**8**	**1**	**213**	**76%**	**22%**	**174**				
BC1 - Y	75%	8 / 15	32	16	16	340	60%	5 / 15	25	12	13	414	75%	8 / 15	44	17	27	481	60%	5 / 15	33	13	20	567	75%	8 / 15	13	13	0	265	60%	5 / 15	12	12	0	259	56%	9 / 15	231				
BC2 - Y	100%	4 / 15	34	15	19	243	100%	4 / 15	30	12	18	285	100%	4 / 15	40	15	25	267	100%	4 / 15	36	13	23	361	100%	4 / 15	13	13	0	242	100%	4 / 15	10	9	1	295	100%	5 / 15	207				
BC3 - C	100%	3 / 28	27	11	16	215	100%	2 / 28	26	12	14	255	100%	3 / 28	34	12	22	289	100%	2 / 28	34	13	21	408	100%	3 / 28	8	8	0	229	100%	2 / 28	9	9	0	267	100%	5 / 28	187				
BC4 - C	0%	0 / 28	7	4	3	29	0%	0 / 28	8	5	3	48	0%	0 / 28	13	5	8	54	0%	0 / 28	15	6	9	84	0%	0 / 28	3	3	0	20	0%	0 / 28	4	4	0	41	0%	0 / 28	54				
BC5 - Y	57%	7 / 15	29	14	15	290	75%	4 / 15	21	11	10	361	57%	7 / 15	38	17	21	395	75%	4 / 15	29	12	17	507	57%	7 / 15	12	12	0	258	75%	4 / 15	9	9	0	336	71%	7 / 15	197				
BC6 - Y	83%	6 / 15	31	11	20	168	100%	6 / 15	30	11	19	185	83%	6 / 15	44	13	31	212	100%	6 / 15	35	12	23	251	83%	6 / 15	9	9	0	113	100%	6 / 15	8	8	0	128	100%	6 / 15	193				
BC7 - C	100%	3 / 28	22	9	13	156	100%	3 / 28	20	6	14	164	100%	3 / 28	29	9	20	204	100%	3 / 28	27	8	19	268	100%	3 / 28	7	6	1	130	100%	3 / 28	4	4	0	114	100%	5 / 28	167				
BC8 - C	100%	1 / 28	23	8	15	158	0%	1 / 28	20	7	13	202	100%	1 / 28	34	9	25	239	0%	1 / 28	26	8	18	322	100%	1 / 28	4	4	0	132	0%	1 / 28	4	4	0	135	50%	2 / 28	178				
BC9 - Y	67%	3 / 15	24	13	11	156	100%	3 / 15	25	13	12	190	67%	3 / 15	37	15	22	185	100%	3 / 15	36	14	22	259	67%	3 / 15	9	8	1	248	100%	3 / 15	11	10	1	271	100%	3 / 15	168				
BC10 - Y	80%	5 / 15	25	10	15	306	75%	4 / 15	22	11	11	225	80%	5 / 15	34	11	23	351	75%	4 / 15	27	12	15	363	80%	5 / 15	9	8	1	265	75%	4 / 15	10	9	1	246	86%	7 / 15	177				
BC11 - C	100%	2 / 28	16	5	11	152	75%	4 / 28	21	6	15	171	100%	2 / 28	25	6	19	197	75%	4 / 28	30	7	23	239	100%	2 / 28	2	2	0	156	75%	4 / 28	3	3	0	152	83%	6 / 28	163				
BC12 - C	67%	3 / 28	15	9	6	200	67%	3 / 28	23	11	12	266	67%	3 / 28	21	10	11	300	67%	3 / 28	29	12	17	355	67%	3 / 28	8	7	1	134	67%	3 / 28	11	8	3	293	67%	3 / 28	145				
BC13 - Y	80%	5 / 15	23	10	13	227	75%	4 / 15	24	10	14	314	80%	5 / 15	36	12	24	364	75%	4 / 15	34	12	22	537	80%	5 / 15	9	9	0	214	75%	4 / 15	9	9	0	303	75%	4 / 15	186				
BC14 - Y	100%	5 / 15	18	10	8	134	100%	5 / 15	20	8	12	169	100%	5 / 15	28	12	16	206	100%	5 / 15	26	10	16	289	100%	5 / 15	6	6	0	143	100%	5 / 15	6	6	0	209	100%	5 / 15	189				
BC19 - C	25%	4 / 28	19	10	9	178	33%	3 / 28	22	10	12	247	25%	4 / 28	32	13	19	191	33%	3 / 28	26	11	15	314	25%	4 / 28	7	7	0	189	33%	3 / 28	7	7	0	226	50%	4 / 28	154				
BC20 - C	60%	5 / 28	28	14	14	207	80%	5 / 28	23	11	12	207	60%	5 / 28	43	15	28	256	80%	5 / 28	34	12	22	376	60%	5 / 28	14	13	1	154	80%	5 / 28	12	10	2	139	83%	6 / 28	185				
**20-plex ex bc4**	**54%**	**14%**	**24**	**8.7**	**15**	**63**	**55%**	**13%**	**24**	**9**	**16**	**76**	**54%**	**14%**	**35**	**11**	**24**	**91**	**55%**	**13%**	**35**	**11**	**25**	**123**	**56%**	**14%**	**7**	**7**	**0**	**32**	**55%**	**13%**	**6**	**6**	**0**	**34**	**81%**	**16%**	**184**				
**20-plex**	**51%**	**13%**	**23**	**8.4**	**15**	**61**	**52%**	**12%**	**23**	**8**	**15**	**73**	**51%**	**13%**	**34**	**11**	**24**	**88**	**52%**	**12%**	**34**	**10**	**24**	**118**	**54%**	**13%**	**6**	**6**	**0**	**30**	**52%**	**12%**	**6**	**6**	**0**	**32**	**77%**	**15%**	**176**				
BC1 - Y	50%	8 / 15	37	15	22	107	43%	7 / 15	36	14	22	138	50%	8 / 15	52	19	33	151	43%	7 / 15	50	17	33	233	50%	8 / 15	13	12	1	58	43%	7 / 15	13	12	1	78	33%	9 / 15	255				
BC2 - Y	100%	7 / 15	31	14	17	73	100%	6 / 15	35	13	22	90	100%	7 / 15	47	18	29	85	100%	6 / 15	50	15	35	104	100%	7 / 15	13	13	0	44	100%	6 / 15	11	11	0	49	100%	7 / 15	209				
BC3 - C	0%	0 / 28	21	9	12	59	0%	0 / 28	22	8	14	88	0%	0 / 28	34	13	21	93	0%	0 / 28	33	11	22	125	0%	0 / 28	7	7	0	29	0%	0 / 28	6	6	0	19	0%	0 / 28	164				
BC4 - C	0%	0 / 28	7	2	5	23	0%	0 / 28	6	2	4	19	0%	0 / 28	12	4	8	29	0%	0 / 28	10	4	6	31	0%	0 / 28	0	0	0	0	0%	0 / 28	0	0	0	0	0%	0 / 28	31				
BC5 - Y	80%	5 / 15	26	13	13	76	80%	5 / 15	27	11	16	91	80%	5 / 15	38	17	21	114	80%	5 / 15	33	13	20	151	80%	5 / 15	10	10	0	35	80%	5 / 15	9	9	0	33	83%	6 / 15	205				
BC6 - Y	100%	5 / 15	29	12	17	53	100%	5 / 15	25	10	15	45	100%	5 / 15	40	13	27	68	100%	5 / 15	37	13	24	93	100%	5 / 15	9	9	0	20	100%	5 / 15	9	9	0	23	83%	6 / 15	198				
BC7 - C	0%	0 / 28	17	5	12	76	0%	0 / 28	21	5	16	82	0%	0 / 28	25	7	18	86	0%	0 / 28	33	8	25	110	0%	0 / 28	3	3	0	31	0%	0 / 28	3	3	0	29	50%	2 / 28	153				
BC8 - C	0%	0 / 28	19	3	16	49	0%	0 / 28	19	4	15	70	0%	0 / 28	27	6	21	86	0%	0 / 28	27	5	22	110	0%	0 / 28	1	1	0	17	0%	0 / 28	2	2	0	28	100%	1 / 28	161				
BC9 - Y	100%	3 / 15	25	11	14	50	100%	3 / 15	23	11	12	59	100%	3 / 15	36	13	23	65	100%	3 / 15	33	13	20	81	100%	3 / 15	8	8	0	36	100%	3 / 15	7	7	0	43	100%	3 / 15	196				
BC10 - Y	50%	2 / 15	25	9	16	63	100%	3 / 15	24	8	16	80	50%	2 / 15	35	11	24	87	100%	3 / 15	35	10	25	128	100%	2 / 15	6	6	0	33	100%	2 / 15	6	6	0	33	100%	3 / 15	193				
BC11 - C	0%	0 / 28	22	7	15	67	0%	1 / 28	20	6	14	67	0%	0 / 28	34	9	25	85	0%	1 / 28	31	8	23	112	0%	1 / 28	3	3	0	57	0%	1 / 28	3	3	0	57	100%	2 / 28	153				
BC12 - C	100%	1 / 28	23	8	15	66	100%	1 / 28	25	8	17	93	100%	1 / 28	31	9	22	80	100%	1 / 28	38	11	27	132	100%	1 / 28	7	7	0	44	100%	1 / 28	6	6	0	49	100%	2 / 28	157				
BC13 - Y	100%	7 / 15	30	13	17	48	100%	6 / 15	25	12	13	46	100%	7 / 15	42	14	28	69	100%	6 / 15	39	14	25	106	100%	7 / 15	12	11	1	26	100%	6 / 15	9	9	0	22	100%	6 / 15	207				
BC14 - Y	67%	6 / 15	18	8	10	40	60%	5 / 15	24	10	14	56	67%	6 / 15	36	9	27	59	60%	5 / 15	37	11	26	78	67%	6 / 15	7	7	0	21	60%	5 / 15	7	7	0	21	67%	3 / 15	189				
BC15 - C	0%	1 / 28	22	8	14	66	0%	1 / 28	21	7	14	64	0%	1 / 28	35	11	24	114	0%	1 / 28	33	9	24	145	0%	1 / 28	5	5	0	23	0%	1 / 28	5	5	0	23	50%	2 / 28	164				
BC16 - C	100%	2 / 28	26	4	22	70	100%	1 / 28	26	6	20	93	100%	2 / 28	38	7	31	112	100%	1 / 28	37	8	29	152	100%	1 / 28	5	4	1	34	100%	1 / 28	5	4	1	34	100%	3 / 28	196				
BC17 - Y	75%	4 / 15	25	8	17	52	67%	3 / 15	24	9	15	73	75%	4 / 15	32	9	23	82	67%	3 / 15	34	11	23	118	75%	4 / 15	6	6	0	28	67%	3 / 15	8	8	0	31	67%	3 / 15	185				
BC18 - Y	100%	6 / 15	23	6	17	57	100%	5 / 15	23	6	17	66	100%	6 / 15	35	8	27	77	100%	5 / 15	30	7	23	99	100%	6 / 15	3	3	0	21	100%	5 / 15	4	4	0	24	100%	4 / 15	185				
BC19 - C	0%	0 / 28	16	6	10	61	0%	0 / 28	18	6	12	71	0%	0 / 28	21	8	13	110	0%	0 / 28	26	8	18	113	0%	0 / 28	4	4	0	23	0%	0 / 28	4	4	0	23	100%	1 / 28	137				
BC20 - C	0%	0 / 28	24	7	17	68	0%	1 / 28	23	9	14	79	0%	0 / 28	34	9	25	107	0%	1 / 28	34	11	23	139	0%	0 / 28	5	5	0	22	0%	0 / 28	5	5	0	22	100%	1 / 28	190				

* Gold Standard consensus: consensus of 19 non-reference-base HapMap SNPs [http://www.sanger.ac.uk/humgen/hapmap3/] and 66 non-reference-base whole genome NGS SNPs [ftp://hgdownload.cse.ucsc.edu/goldenPath/hg18/database/pgYh1.txt.gz], [http://solidsoftwaretools.com/gf/project/yoruban/, Yoruban_snp_18x.gff], see Table S19

** Bronze Standard: 28 non-reference-base HapMap SNPs [http://www.sanger.ac.uk/humgen/hapmap3/], not validated by further means.

Annotated SNPs are SNPs in dbSNP130

This table comprises Tables S2 and S11. We separated barcode 4 samples from the other samples, because the SOLiD on-machine software SETS assigned only ca. 1% of the expected reads to barcode 4. After this separation, we observe the expected even distribution of concordance/overlap within samples of the same plex, and the expected deterioration of results for higher plexing. Target region (TR) mapping of raw reads leads to the highest number of known SNPs, despite lower coverages, and also to the highest number of potential novel SNPs. Whole genome (WG) mapping leads to near-zero potential novel SNPs. Backmapping reduces the total number of SNPs by 15-20% and the number of potential novel SNPs by about a third, eliminating obvious false positives or errors. To validate the potential novel SNPs we examined inter-sample concordances (Table S20) and manually inspected reads at these positions in IGV (Table S12). The sequencing error is probably much smaller than the Bioscope diBayes SNP-calling error (see Table S2, footnote ** manual inspection using IGV).

Table S8: Accurate sequencing is confirmed for the Yoruban samples using the Integrated Genomics Viewer (IGV*)

					IGV results					
Sample	SNP	Pos.	Ref	HapMap	∑ reads	∑ A	∑ C	∑ G	∑ T	∑ N
4-plex1 bc05	rs28897686	36800	C	G	656	0	656	0	0	0
	rs4986848	37582	A	T	240	240	0	0	0	0
	rs28897673	47280	T	A	115	0	0	0	115	0
	rs28897731	83819	T	A	291	0	0	1	275	0
	rs206123	69217	G	C	20	0	16	1	0	4
4-plex1 bc06	rs28897686	36800	C	G	431	0	431	0	0	0
	rs4986848	37582	A	T	182	182	0	0	0	0
	rs28897673	47280	T	A	44	0	0	0	44	0
	rs28897731	83819	T	A	171	0	0	0	171	0
	rs206123	69217	G	C	43	0	35	3	0	5
4-plex2 bc09	rs28897686	36800	C	G	1079	0	1076	0	1	2
	rs4986848	37582	A	T	350	350	0	0	0	0
	rs28897673	47280	T	A	49	0	0	0	49	0
	rs28897731	83819	T	A	440	1	0	0	437	2
	rs206123	69217	G	C	29	0	28	1	0	0
4-plex2 bc10	rs28897686	36800	C	G	1255	0	1255	0	0	0
	rs4986848	37582	A	T	298	297	0	0	0	1
	rs28897673	47280	T	A	114	0	1	1	112	0
	rs28897731	83819	T	A	754	0	0	0	754	0
	rs206123	69217	G	C	36	0	32	0	0	4
8-plex bc01	rs28897686	36800	C	G	489	0	489	0	0	0
	rs4986848	37582	A	T	222	222	0	0	0	0
	rs28897673	47280	T	A	46	0	0	0	45	1
	rs28897731	83819	T	A	135	0	0	0	134	1
	rs206123	69217	G	C	16	0	13	1	0	2
8-plex bc02	rs28897686	36800	C	G	283	0	283	0	0	0
	rs4986848	37582	A	T	43	43	0	0	0	0
	rs28897673	47280	T	A	19	0	0	0	19	0
	rs28897731	83819	T	A	325	0	0	0	325	0
	rs206123	69217	G	C	8	0	8	0	0	0
8-plex bc05	rs28897686	36800	C	G	519	0	519	0	0	0
	rs4986848	37582	A	T	91	91	0	0	0	0
	rs28897673	47280	T	A	41	0	0	0	41	0
	rs28897731	83819	T	A	217	0	0	0	217	0
	rs206123	69217	G	C	17	0	12	2	0	3
8-plex bc06	rs28897686	36800	C	G	327	1	326	0	0	0
	rs4986848	37582	A	T	81	81	0	0	0	0
	rs28897673	47280	T	A	25	0	0	0	25	0
	rs28897731	83819	T	A	54	0	0	0	54	0
	rs206123	69217	G	C	54	0	34	7	0	13
16-plex bc01	rs28897686	36800	C	G	470	0	469	0	0	1
	rs4986848	37582	A	T	21	21	0	0	0	0
	rs28897673	47280	T	A	5	0	0	0	5	0
	rs28897731	83819	T	A	252	0	0	0	251	1
	rs206123	69217	G	C	28	0	27	0	0	1
16-plex bc02	rs28897686	36800	C	G	192	1	191	0	0	0
	rs4986848	37582	A	T	60	60	0	0	0	0
	rs28897673	47280	T	A	15	0	0	0	15	0
	rs28897731	83819	T	A	3	0	0	0	3	0
	rs206123	69217	G	C	28	0	27	1	0	0
16-plex bc05	rs28897686	36800	C	G	319	0	319	0	0	0
	rs4986848	37582	A	T	45	45	0	0	0	0
	rs28897673	47280	T	A	5	0	0	0	5	0
	rs28897731	83819	T	A	124	0	0	0	124	0
	rs206123	69217	G	C	2	0	1	1	0	0
16-plex bc06	rs28897686	36800	C	G	284	0	284	0	0	0
	rs4986848	37582	A	T	68	68	0	0	0	0
	rs28897673	47280	T	A	6	0	0	0	6	0
	rs28897731	83819	T	A	65	0	0	0	65	0
	rs206123	69217	G	C	2	0	2	0	0	0
16-plex bc09	rs28897686	36800	C	G	138	0	138	0	0	0
	rs4986848	37582	A	T	2	2	0	0	0	0
	rs28897673	47280	T	A	4	0	0	0	4	0
	rs28897731	83819	T	A	3	0	0	0	3	0
	rs206123	69217	G	C	1	0	1	0	0	0
16-plex bc10	rs28897686	36800	C	G	217	0	217	0	0	0
	rs4986848	37582	A	T	20	20	0	0	0	0
	rs28897673	47280	T	A	9	0	0	0	9	0
	rs28897731	83819	T	A	33	0	0	0	33	0
	rs206123	69217	G	C	15	0	15	0	0	0
16-plex bc13	rs28897686	36800	C	G	339	0	339	0	0	0
	rs4986848	37582	A	T	77	77	0	0	0	0
	rs28897673	47280	T	A	23	0	1	0	22	0
	rs28897731	83819	T	A	203	0	0	0	203	0
	rs206123	69217	G	C	1	1	0	0	0	0
16-plex bc14	rs28897686	36800	C	G	436	0	436	0	0	0
	rs4986848	37582	A	T	48	48	0	0	0	0
	rs28897673	47280	T	A	31	0	0	0	31	0
	rs28897731	83819	T	A	266	0	0	0	266	0
	rs206123	69217	G	C	4	0	4	0	0	0
control (target region mapping of SAET reads)	rs28897686	36800	C	G	5382	1	5377	0	1	3
	rs4986848	37582	A	T	3862	3860	0	0	0	2
	rs28897673	47280	T	A	2147	1	1	1	2141	3
	rs28897731	83819	T	A	2401	2	1	0	2397	1
	rs206123	69217	G	C	799	0	788	7	0	4
control (target region mapping of off-machine reads)	rs28897686	36800	C	G	4819	1	4816	0	1	1
	rs4986848	37582	A	T	3517	3516	1	0	0	0
	rs28897673	47280	T	A	1817	3	2	8	1799	5
	rs28897731	83819	T	A	1952	1	0	2	1948	1
	rs206123	69217	G	C	653	1	535	82	1	34
control (whole genome mapping of SAET reads)	rs28897686	chr17:38497326	C	G	5369	0	5364	0	1	3
	rs4986848	chr17:38498108	A	T	3825	3823	0	0	0	2
	rs28897673	chr17:38509792	T	A	2138	1	1	1	2132	3
	rs28897731	chr13:31811420	T	A	2165	0	0	1	2162	2
	rs206123	chr13:31793377	G	C	799	0	788	7	0	4

* IGV [http://www.broadinstitute.org/igv/v1.4]

** HapMap [http://www.sanger.ac.uk/humgen/hapmap3/]

*** whole genome NGS [ftp://hgdownload.cse.ucsc.edu/goldenPath/hg18/database/pgYh1.txt.gz], [http://solidsoftwaretools.com/gf/project/yoruban/, Yoruban_snp_18x.gff]

SAET-enhanced reads mapped to target region and to whole genome, using Bioscope 1.0.1 (control sample: also raw off-machine reads). Settings see Table T1 in http://www.ikmb.uni-kiel.de/tngs-backmapping/bioscope_settings.xls

We manually inspected 19 mapping runs (17 separate samples) for the HapMap Yoruban individual NA18507 using IGV. We manually inspected 5 SNPs within the BRCA1/2 target region which are given in the HapMap3** data. We do not confirm the first 4 HapMap SNPs (rs28897686, rs4986848, rs28897673, rs28897731) manually, nor automatically with Bioscope diBayes, nor in the whole genome NGS*** data. We resequenced the first 3 SNPs with the Sanger method, confirming errors in HapMap. We did not resequence the 4th erroneous HapMap SNP with the Sanger method, because we discovered this error later, and it falls into the same pattern as the first 3 errors. It should be noted that the HapMap website estimates a false positive SNP rate of 12%-14% in their data (June 2012). The last SNP (rs206123) is not called by any diBayes analysis, but our manual inspections confirm the presence of this variant for SAET-enhanced reads as well as for raw off-machine reads, and for target region mapping as well as for whole genome mapping.

Table S9: CLC bio Workbench 3.7.1 SNP-List after target region mapping (Yoruban HapMap ID NA18507, sample 4-plex1bc05)

Chromosome (chr1: target region mapping)	CLCBio or HapMap	Variation type	Reference position	Reference position	Length	Reference	Variants	Allele variations	Frequencies	Counts	Coverage	Variant #1	Frequency of #1	Count of #1	Variant #2	Frequency of #2	Count of #2	Overlapping annotations	Amino acid change
chr1	CLCBio	SNP	717	717	1	C	1	A	81.8	9	11	A	81.8181818	9					
chr1	CLCBio	SNP	730	730	1	A	2	A/G	92.9/6.0	78/5	84	A	92.8571429	78	G	5.95238095	5		
chr1	CLCBio	SNP	734	734	1	T	2	T/C	91.9/8.1	91/8	99	T	91.9191919	91	C	8.08080808	8		
chr1	CLCBio	SNP	749	749	1	C	2	C/T	96.9/3.1	155/5	160	C	96.875	155	T	3.125	5		
chr1	CLCBio	SNP	762	762	1	C	2	C/T	92.5/5.6	148/9	160	C	92.5	148	T	5.625	9		
chr1	CLCBio	SNP	763	763	1	G	2	G/A	91.0/9.0	162/16	178	G	91.011236	162	A	8.98876404	16		
chr1	CLCBio	SNP	773	773	1	A	2	A/G	96.0/4.0	192/8	200	A	96	192	G	4	8		
chr1	CLCBio	SNP	774	774	1	T	2	T/C	96.6/3.4	197/7	204	T	96.5686275	197	C	3.43137255	7		
chr1	CLCBio	SNP	936	936	1	C	2	C/A	95.5/4.5	404/19	423	C	95.5082742	404	A	4.49172577	19		
chr1	CLCBio	SNP	1328	1328	1	T	2	T/C	99.0/1.0	1032/10	1042	T	99.0403071	1032	C	0.9596929	10		
chr1	CLCBio	SNP	1369	1369	1	G	2	G/A	99.7/0.3	3294/11	3305	G	99.667171	3294	A	0.33282905	11		
chr1	CLCBio	SNP	1373	1373	1	G	2	G/A	98.8/1.2	3179/37	3216	G	98.8495025	3179	A	1.15049751	37		
chr1	CLCBio	SNP	1404	1404	1	C	2	C/A	99.3/0.7	1267/9	1276	C	99.2946708	1267	A	0.70532915	9		
chr1	CLCBio	SNP	1421	1421	1	G	2	G/T	99.4/0.6	2344/13	2357	G	99.4484514	2344	T	0.55154858	13		
chr1	CLCBio	SNP	1426	1426	1	A	2	A/G	99.4/0.6	2263/13	2276	A	99.4288225	2263	G	0.5711775	13		
chr1	CLCBio	SNP	1455	1455	1	C	2	C/T	99.4/0.6	1884/11	1895	C	99.4195251	1884	T	0.58047493	11		
chr1	CLCBio	SNP	1874	1874	1	G	2	G/T	98.9/1.1	606/7	613	G	98.858075	606	T	1.14192496	7		
chr1	CLCBio	SNP	2469	2469	1	G	2	G/T	98.3/1.7	296/5	301	G	98.3388704	296	T	1.66112957	5		
chr1	CLCBio	SNP	2738	2738	1	C	2	C/T	91.6/8.4	87/8	95	C	91.5789474	87	T	8.42105263	8		
chr1	CLCBio	SNP	2739	2739	1	G	2	G/A	88.8/11.2	95/12	107	G	88.7850467	95	A	11.2149533	12		
chr1	CLCBio	SNP	2765	2765	1	A	2	A/G	82.2/16.7	143/29	174	A	82.183908	143	G	16.6666667	29		
chr1	CLCBio	SNP	2777	2777	1	A	2	A/G	96.8/3.2	214/7	221	A	96.8325792	214	G	3.16742081	7		
chr1	CLCBio	SNP	2884	2884	1	A	2	A/G	96.6/3.4	143/5	148	A	96.6216216	143	G	3.37837838	5		
chr1	CLCBio	SNP	3056	3056	1	G	2	G/A	99.5/0.5	1233/6	1239	G	99.5157385	1233	A	0.4842615	6		
chr1	CLCBio	SNP	3102	3102	1	G	2	G/T	97.3/2.7	1286/36	1322	G	97.2768533	1286	T	2.72314675	36		
chr1	CLCBio	SNP	3122	3122	1	C	2	C/A	99.0/1.0	932/9	941	C	99.0435707	932	A	0.95642933	9		
chr1	CLCBio	SNP	3796	3796	1	C	2	C/T	97.2/2.7	1100/31	1132	C	97.1731449	1100	T	2.7385159	31		
chr1	CLCBio	SNP	4306	4306	1	A	2	A/G	89.6/10.4	60/7	67	A	89.5522388	60	G	10.4477612	7		
chr1	CLCBio	SNP	4327	4327	1	T	2	T/C	50.8/47.5	61/57	120	T	50.8333333	61	C	47.5	57		
chr1	CLCBio	SNP	4343	4343	1	T	2	T/C	92.0/8.0	104/9	113	T	92.0353982	104	C	7.96460177	9		
chr1	CLCBio	SNP	4349	4349	1	G	2	G/A	93.3/6.7	70/5	75	G	93.3333333	70	A	6.66666667	5		
chr1	CLCBio	SNP	4526	4526	1	G	2	G/A	65.2/34.8	197/105	302	G	65.2317881	197	A	34.7682119	105		
chr1	CLCBio	SNP	5645	5645	1	T	2	T/C	75.0/25.0	12/4	16	T	75	12	C	25	4		
chr1	CLCBio	SNP	5815	5815	1	C	2	C/A	99.2/0.8	1056/8	1064	C	99.2481203	1056	A	0.7518797	8		
chr1	CLCBio	SNP	5829	5829	1	C	2	C/A	98.0/2.0	1033/21	1054	C	98.0075901	1033	A	1.99240987	21		
chr1	CLCBio	SNP	6346	6346	1	G	2	G/A	96.9/3.1	156/5	161	G	96.8944099	156	A	3.10559006	5		
chr1	CLCBio	Complex SNP	6351	6351	1	G	4	G/C/A	78.2/18.3/2.0/	269/63/7/5	344	G	78.1976744	269	C	18.3139535	63		
chr1	CLCBio	Complex SNP	6353	6353	1	A	3	A/C/T	79.3/16.6/3.8	482/101/23	608	A	79.2763158	482	C	16.6118421	101		
chr1	CLCBio	SNP	6354	6354	1	A	2	A/T	99.1/0.5	1534/8	1548	A	99.0956072	1534	T	0.51679587	8		
chr1	CLCBio	Complex SNP	6355	6355	1	C	3	C/T/G	98.9/0.8/0.2	2026/16/5	2048	C	98.9257813	2026	T	0.78125	16		
chr1	CLCBio	SNP	6398	6398	1	T	2	T/A	96.1/3.4	423/15	440	T	96.1363636	423	A	3.40909091	15		
chr1	CLCBio	SNP	6400	6400	1	T	2	T/A	98.2/1.8	275/5	280	T	98.2142857	275	A	1.78571429	5		
chr1	CLCBio	SNP	6578	6578	1	T	2	T/C	57.1/42.9	24/18	42	T	57.1428571	24	C	42.8571429	18		
chr1	CLCBio	SNP	6598	6598	1	T	2	T/C	93.3/6.7	140/10	150	T	93.3333333	140	C	6.66666667	10		
chr1	CLCBio	SNP	6613	6613	1	A	2	A/G	92.9/7.1	353/27	380	A	92.8947368	353	G	7.10526316	27		
chr1	CLCBio	SNP	6620	6620	1	A	2	A/G	99.0/1.0	472/5	477	A	98.951782	472	G	1.04821803	5		
chr1	CLCBio	SNP	6624	6624	1	G	2	G/A	99.1/0.9	543/5	548	G	99.0875912	543	A	0.91240876	5		
chr1	CLCBio	SNP	6811	6811	1	G	2	G/T	99.0/1.0	622/6	628	G	99.044586	622	T	0.95541401	6		
chr1	CLCBio	SNP	6892	6892	1	A	2	A/G	95.9/4.1	164/7	171	A	95.9064327	164	G	4.09356725	7		
chr1	CLCBio	Complex SNP	6901	6901	1	C	3	C/T/A	91.4/6.3/2.2	245/17/6	268	C	91.4179104	245	T	6.34328358	17		
chr1	CLCBio	SNP	6902	6902	1	G	2	G/A	92.1/7.5	233/19	253	G	92.0948617	233	A	7.50988142	19		
chr1	CLCBio	SNP	6904	6904	1	C	2	C/T	98.1/1.9	259/5	264	C	98.1060606	259	T	1.89393939	5		
chr1	CLCBio	SNP	6906	6906	1	G	2	G/A	97.6/2.1	281/6	288	G	97.5694444	281	A	2.08333333	6		
chr1	CLCBio	SNP	6922	6922	1	G	2	G/A	96.6/3.4	168/6	174	G	96.5517241	168	A	3.44827586	6		
chr1	CLCBio	Complex SNP	6929	6929	1	T	3	T/C/A	86.4/10.4/3.2	191/23/7	221	T	86.4253394	191	C	10.4072398	23		
chr1	CLCBio	SNP	6935	6935	1	A	2	A/T	94.8/3.6	182/7	192	A	94.7916667	182	T	3.64583333	7		
chr1	CLCBio	SNP	6938	6938	1	C	2	C/T	80.8/17.4	135/29	167	C	80.8383234	135	T	17.3652695	29		

chr1	CLCBio	SNP	6939	6939	1	G	2	G/A	85.5/12.1	148/21	173	G	85.5491329	148	A	12.1387283	21		
chr1	CLCBio	SNP	6945	6945	1	C	2	C/T	92.5/7.5	98/8	106	C	92.4528302	98	T	7.54716981	8		
chr1	CLCBio	SNP	7452	7452	1	G	2	G/C	97.9/2.1	714/15	729	G	97.9423868	714	C	2.05761317	15		
chr1	CLCBio	SNP	7513	7513	1	C	2	C/A	99.5/0.5	1313/7	1320	C	99.469697	1313	A	0.53030303	7		
chr1	CLCBio	SNP	7613	7613	1	C	2	C/T	98.1/1.7	353/6	360	C	98.0555556	353	T	1.66666667	6		
chr1	CLCBio	SNP	7648	7648	1	C	2	C/T	96.3/3.7	360/14	374	C	96.2566845	360	T	3.74331551	14		
chr1	CLCBio	SNP	7881	7881	1	G	2	G/C	60.0/40.0	6/4	10	G	60	6	C	40	4		
chr1	CLCBio	SNP	7961	7961	1	G	2	G/T	98.8/1.2	648/8	656	G	98.7804878	648	T	1.2195122	8		
chr1	CLCBio	SNP	7977	7977	1	C	2	C/A	97.3/2.7	538/15	553	C	97.2875226	538	A	2.7124774	15		
chr1	CLCBio	SNP	8287	8287	1	A	2	A/G	71.4/28.6	5/2	7	A	71.4285714	5	G	28.5714286	2		
chr1	CLCBio	SNP	8295	8295	1	C	2	C/T	57.1/42.9	4/3	7	C	57.1428571	4	T	42.8571429	3		
chr1	CLCBio	SNP	8916	8916	1	G	2	G/T	97.1/2.9	235/7	242	G	97.107438	235	T	2.89256198	7		
chr1	CLCBio	SNP	8956	8956	1	A	2	A/G	80.0/20.0	16/4	20	A	80	16	G	20	4		
chr1	CLCBio	SNP	9068	9068	1	A	1	G	100.0	10	10	G	100	10					
chr1	CLCBio	SNP	9265	9265	1	C	1	A	100.0	4	4	A	100	4					
chr1	CLCBio	SNP	10323	10323	1	G	2	G/T	97.6/2.4	321/8	329	G	97.5683891	321	T	2.43161094	8		
chr1	CLCBio	SNP	10755	10755	1	C	2	C/T	75.0/25.0	21/7	28	C	75	21	T	25	7		
chr1	CLCBio	SNP	10837	10837	1	G	2	A/G	66.7/22.2	6/2	9	A	66.6666667	6	G	22.2222222	2		
chr1	CLCBio	SNP	11901	11901	1	T	2	C/T	50.0/50.0	2/2	4	C	50	2	T	50	2		
chr1	CLCBio	SNP	12972	12972	1	G	2	G/T	98.0/2.0	447/9	456	G	98.0263158	447	T	1.97368421	9		
chr1	CLCBio	SNP	13033	13033	1	G	2	G/T	97.3/2.7	649/18	667	G	97.3013493	649	T	2.69865067	18		
chr1	CLCBio	SNP	13034	13034	1	T	2	T/C	98.7/1.3	623/8	631	T	98.7321712	623	C	1.26782884	8		
chr1	CLCBio	SNP	14171	14171	1	C	2	C/T	88.7/9.7	55/6	62	C	88.7096774	55	T	9.67741935	6		
chr1	CLCBio	SNP	14176	14176	1	T	2	T/C	63.6/34.1	28/15	44	T	63.6363636	28	C	34.0909091	15		
chr1	CLCBio	SNP	14177	14177	1	A	2	A/G	74.4/25.6	32/11	43	A	74.4186047	32	G	25.5813953	11		
chr1	CLCBio	SNP	14877	14877	1	T	2	T/C	72.7/27.3	8/3	11	T	72.7272727	8	C	27.2727273	3		
chr1	CLCBio	SNP	14896	14896	1	G	2	G/C	71.4/28.6	10/4	14	G	71.4285714	10	C	28.5714286	4		
chr1	CLCBio	SNP	15004	15004	1	G	2	G/T	98.5/1.5	473/7	480	G	98.5416667	473	T	1.45833333	7		
chr1	CLCBio	SNP	15032	15032	1	C	2	C/A	97.6/2.4	249/6	255	C	97.6470588	249	A	2.35294118	6		
chr1	CLCBio	SNP	15190	15190	1	G	2	G/A	91.0/7.5	61/5	67	G	91.0447761	61	A	7.46268657	5		
chr1	CLCBio	SNP	15194	15194	1	C	2	C/T	93.8/6.2	76/5	81	C	93.8271605	76	T	6.17283951	5		
chr1	CLCBio	SNP	15195	15195	1	G	2	G/A	88.5/10.3	77/9	87	G	88.5057471	77	A	10.3448276	9		
chr1	CLCBio	SNP	15212	15212	1	A	2	A/G	52.2/44.6	48/41	92	A	52.173913	48	G	44.5652174	41		
chr1	CLCBio	SNP	15221	15221	1	G	2	G/A	85.3/14.7	29/5	34	G	85.2941176	29	A	14.7058824	5		
chr1	CLCBio	SNP	15222	15222	1	T	2	T/C	85.4/12.5	41/6	48	T	85.4166667	41	C	12.5	6		
chr1	CLCBio	SNP	15528	15528	1	C	2	C/G	73.3/16.7	22/5	30	C	73.3333333	22	G	16.6666667	5		
chr1	CLCBio	SNP	15768	15768	1	T	2	T/C	67.0/31.9	63/30	94	T	67.0212766	63	C	31.9148936	30		
chr1	CLCBio	SNP	16080	16080	1	C	2	C/T	93.1/6.9	67/5	72	C	93.0555556	67	T	6.94444444	5		
chr1	CLCBio	SNP	16091	16091	1	T	2	C/T	50.0/46.9	32/30	64	C	50	32	T	46.875	30		
chr1	CLCBio	SNP	16125	16125	1	A	2	A/G	75.0/25.0	3/1	4	A	75	3	G	25	1		
chr1	CLCBio	SNP	16436	16436	1	A	2	A/G	75.0/25.0	6/2	8	A	75	6	G	25	2		
chr1	CLCBio	SNP	16452	16452	1	C	2	C/T	75.0/25.0	3/1	4	C	75	3	T	25	1		
chr1	CLCBio	SNP	16785	16785	1	G	2	G/T	98.7/1.3	453/6	459	G	98.6928105	453	T	1.30718954	6		
chr1	CLCBio	SNP	17058	17058	1	C	2	C/A	99.2/0.8	1167/10	1177	C	99.1503823	1167	A	0.84961767	10		
chr1	CLCBio	SNP	17099	17099	1	C	2	C/A	99.2/0.8	1461/12	1473	C	99.185336	1461	A	0.81466395	12		
chr1	CLCBio	SNP	17147	17147	1	A	2	A/C	98.6/1.4	562/8	570	A	98.5964912	562	C	1.40350877	8		
chr1	CLCBio	Complex SNP	18122	18122	1	T	3	T/G/A	69.8/17.5/12.7	44/11/8	63	T	69.8412698	44	G	17.4603175	11		
chr1	CLCBio	SNP	18867	18867	1	C	2	C/T	96.8/3.2	609/20	629	C	96.8203498	609	T	3.17965024	20		
chr1	CLCBio	SNP	18870	18870	1	G	2	G/A	99.3/0.7	896/6	902	G	99.3348115	896	A	0.66518847	6		
chr1	CLCBio	Complex SNP	18914	18914	1	G	3	G/T/C	99.5/0.3/0.2	2042/6/5	2053	G	99.4641987	2042	T	0.29225524	6		
chr1	CLCBio	SNP	18916	18916	1	T	2	T/C	99.7/0.3	2133/6	2139	T	99.7194951	2133	C	0.28050491	6		
chr1	CLCBio	SNP	19056	19056	1	A	2	A/T	98.7/1.1	452/5	458	A	98.6899563	452	T	1.09170306	5		
chr1	CLCBio	SNP	19528	19528	1	A	2	A/G	51.6/45.2	16/14	31	A	51.6129032	16	G	45.1612903	14		
chr1	CLCBio	SNP	19543	19543	1	A	2	A/G	91.2/7.4	62/5	68	A	91.1764706	62	G	7.35294118	5		
chr1	CLCBio	SNP	19571	19571	1	G	2	G/A	94.6/5.4	106/6	112	G	94.6428571	106	A	5.35714286	6		
chr1	CLCBio	SNP	19574	19574	1	A	2	G/A	61.8/38.2	42/26	68	G	61.7647059	42	A	38.2352941	26		
chr1	CLCBio	SNP	19579	19579	1	T	2	T/C	81.6/18.4	80/18	98	T	81.6326531	80	C	18.3673469	18		
chr1	CLCBio	SNP	19586	19586	1	C	2	C/T	92.0/8.0	80/7	87	C	91.954023	80	T	8.04597701	7		
chr1	CLCBio	SNP	19785	19785	1	G	2	G/A	84.4/15.6	27/5	32	G	84.375	27	A	15.625	5		
chr1	CLCBio	Complex SNP	19794	19794	1	G	2	T/A	81.4/11.6	35/5	43	T	81.3953488	35	A	11.627907	5		
chr1	CLCBio	SNP	19827	19827	1	T	2	T/C	87.3/9.5	55/6	63	T	87.3015873	55	C	9.52380952	6		
chr1	CLCBio	SNP	19832	19832	1	G	2	G/A	89.4/10.6	42/5	47	G	89.3617021	42	A	10.6382979	5		
chr1	CLCBio	SNP	19839	19839	1	C	2	C/T	83.3/16.7	30/6	36	C	83.3333333	30	T	16.6666667	6		
chr1	CLCBio	SNP	19902	19902	1	C	2	C/T	80.0/20.0	4/1	5	C	80	4	T	20	1		
chr1	CLCBio	SNP	19903	19903	1	G	2	G/A	75.0/25.0	3/1	4	G	75	3	A	25	1		

chr1	CLCBio	SNP	19916	19916	1	T	2	T/C	75.9/17.2	22/5	29	T	75.862069	22	C	17.2413793	5		
chr1	CLCBio	SNP	19918	19918	1	C	2	C/T	81.2/18.8	26/6	32	C	81.25	26	T	18.75	6		
chr1	CLCBio	SNP	19949	19949	1	T	2	T/C	64.3/28.6	18/8	28	T	64.2857143	18	C	28.5714286	8		
chr1	CLCBio	SNP	20279	20279	1	T	2	T/C	66.7/33.3	4/2	6	T	66.6666667	4	C	33.3333333	2		
chr1	CLCBio	SNP	21517	21517	1	G	2	G/T	99.0/1.0	1225/12	1237	G	99.0299111	1225	T	0.97008892	12		
chr1	CLCBio	SNP	21562	21562	1	G	2	G/A	99.1/0.9	749/7	756	G	99.0740741	749	A	0.92592593	7		
chr1	CLCBio	SNP	21594	21594	1	C	2	C/T	97.9/2.1	640/14	654	C	97.8593272	640	T	2.14067278	14		
chr1	CLCBio	SNP	21611	21611	1	A	2	A/G	63.4/36.6	635/367	1002	A	63.3732535	635	G	36.6267465	367		
chr1	CLCBio	SNP	21781	21781	1	A	2	A/T	98.7/1.3	887/12	899	A	98.6651835	887	T	1.33481646	12		
chr1	CLCBio	SNP	21785	21785	1	A	2	A/C	99.4/0.6	894/5	899	A	99.4438265	894	C	0.55617353	5		
chr1	CLCBio	SNP	21913	21913	1	T	2	T/G	96.3/2.7	211/6	219	T	96.347032	211	G	2.73972603	6		
chr1	CLCBio	SNP	21954	21954	1	A	1	G	100.0	14	14	G	100	14					
chr1	CLCBio	SNP	21957	21957	1	T	2	T/C	66.7/33.3	6/3	9	T	66.6666667	6	C	33.3333333	3		
chr1	CLCBio	SNP	21958	21958	1	T	2	T/C	77.8/22.2	7/2	9	T	77.7777778	7	C	22.2222222	2		
chr1	CLCBio	SNP	22592	22592	1	A	2	G/A	54.2/45.8	13/11	24	G	54.1666667	13	A	45.8333333	11		
chr1	CLCBio	SNP	22659	22659	1	A	2	A/G	88.9/9.5	112/12	126	A	88.8888889	112	G	9.52380952	12		
chr1	CLCBio	SNP	22666	22666	1	A	2	A/T	98.1/1.1	881/10	898	A	98.1069042	881	T	1.11358575	10		
chr1	CLCBio	SNP	22667	22667	1	G	2	G/A	99.0/1.0	816/8	824	G	99.0291262	816	A	0.97087379	8		
chr1	CLCBio	SNP	22668	22668	1	C	2	C/T	95.6/3.7	847/33	886	C	95.5981941	847	T	3.72460497	33		
chr1	CLCBio	Complex SNP	22669	22669	1	A	4	A/G/C	70.2/26.0/2.2/	510/189/16/1	726	A	70.2479339	510	G	26.0330579	189		
chr1	CLCBio	SNP	22670	22670	1	A	2	A/G	98.5/1.1	737/8	748	A	98.5294118	737	G	1.06951872	8		
chr1	CLCBio	SNP	22673	22673	1	C	2	C/T	99.2/0.5	1061/5	1070	C	99.1588785	1061	T	0.46728972	5		
chr1	CLCBio	SNP	22674	22674	1	T	2	T/C	99.4/0.6	933/6	939	T	99.3610224	933	C	0.63897764	6		
chr1	CLCBio	SNP	22675	22675	1	C	2	C/T	98.0/1.6	846/14	863	C	98.0301275	846	T	1.62224797	14		
chr1	CLCBio	Complex SNP	22676	22676	1	C	4	C/T/G	97.1/1.3/1.0/0	1139/15/12/7	1173	C	97.1014493	1139	T	1.27877238	15		
chr1	CLCBio	SNP	22677	22677	1	T	2	T/C	99.3/0.5	1029/5	1036	T	99.3243243	1029	C	0.48262548	5		
chr1	CLCBio	SNP	22678	22678	1	G	2	G/A	98.5/1.1	1131/13	1148	G	98.5191638	1131	A	1.13240418	13		
chr1	CLCBio	SNP	22679	22679	1	C	2	C/T	98.1/1.6	735/12	749	C	98.1308411	735	T	1.60213618	12		
chr1	CLCBio	SNP	22680	22680	1	C	2	C/T	99.2/0.6	1054/6	1063	C	99.1533396	1054	T	0.56444026	6		
chr1	CLCBio	SNP	22682	22682	1	C	2	C/T	99.1/0.8	965/8	974	C	99.0759754	965	T	0.82135524	8		
chr1	CLCBio	SNP	22684	22684	1	G	2	G/A	98.9/0.8	1145/9	1158	G	98.8773748	1145	A	0.77720207	9		
chr1	CLCBio	SNP	22685	22685	1	C	2	C/T	98.8/0.7	960/7	972	C	98.7654321	960	T	0.72016461	7		
chr1	CLCBio	SNP	22686	22686	1	C	2	C/T	99.2/0.5	1188/6	1197	C	99.2481203	1188	T	0.50125313	6		
chr1	CLCBio	SNP	22687	22687	1	T	2	T/C	99.4/0.4	1206/5	1213	T	99.4229184	1206	C	0.41220115	5		
chr1	CLCBio	SNP	22688	22688	1	C	2	C/T	98.5/1.4	1021/14	1037	C	98.4570878	1021	T	1.35004822	14		
chr1	CLCBio	SNP	22689	22689	1	C	2	C/T	98.9/1.0	923/9	933	C	98.9281886	923	T	0.96463023	9		
chr1	CLCBio	Complex SNP	22690	22690	1	T	3	T/C/A	73.5/24.5/1.7	790/263/18	1075	T	73.4883721	790	C	24.4651163	263		
chr1	CLCBio	Complex SNP	22691	22691	1	G	3	G/A/C	97.0/2.2/0.9	1119/25/10	1154	G	96.9670711	1119	A	2.16637782	25		
chr1	CLCBio	SNP	22693	22693	1	G	2	G/A	99.1/0.7	1197/8	1208	G	99.089404	1197	A	0.66225166	8		
chr1	CLCBio	Complex SNP	22696	22696	1	G	3	G/A/T	98.7/0.6/0.4	1158/7/5	1173	G	98.7212276	1158	A	0.59676044	7		
chr1	CLCBio	SNP	22697	22697	1	C	2	C/T	99.0/0.5	918/5	927	C	99.0291262	918	T	0.53937433	5		
chr1	CLCBio	SNP	22699	22699	1	G	2	G/A	98.8/1.1	1123/13	1137	G	98.7686895	1123	A	1.14335972	13		
chr1	CLCBio	SNP	22700	22700	1	G	2	G/A	98.0/1.7	1201/21	1226	G	97.9608483	1201	A	1.71288744	21		
chr1	CLCBio	SNP	22701	22701	1	G	2	G/A	99.0/0.7	1143/8	1154	G	99.0467938	1143	A	0.6932409	8		
chr1	CLCBio	SNP	22703	22703	1	T	2	T/C	88.5/11.3	1008/129	1139	T	88.4986831	1008	C	11.3257243	129		
chr1	CLCBio	SNP	22705	22705	1	A	2	A/G	99.5/0.5	1060/5	1065	A	99.5305164	1060	G	0.46948357	5		
chr1	CLCBio	SNP	22706	22706	1	C	2	C/T	98.8/1.2	976/12	988	C	98.7854251	976	T	1.2145749	12		
chr1	CLCBio	SNP	22708	22708	1	G	2	G/A	98.9/0.8	831/7	840	G	98.9285714	831	A	0.83333333	7		
chr1	CLCBio	SNP	22710	22710	1	C	2	C/T	99.0/1.0	493/5	498	C	98.9959839	493	T	1.00401606	5		
chr1	CLCBio	SNP	22711	22711	1	A	2	A/G	95.2/4.3	376/17	395	A	95.1898734	376	G	4.30379747	17		
chr1	CLCBio	SNP	22712	22712	1	C	2	C/T	93.8/5.0	303/16	323	C	93.8080495	303	T	4.95356037	16		
chr1	CLCBio	Complex SNP	22713	22713	1	C	4	C/A/G	85.1/7.9/5.0/2	257/24/15/6	302	C	85.0993377	257	A	7.94701987	24		
chr1	CLCBio	SNP	22714	22714	1	T	2	T/C	71.9/26.4	128/47	178	T	71.9101124	128	C	26.4044944	47		
chr1	CLCBio	SNP	22715	22715	1	G	2	G/A	97.0/3.0	192/6	198	G	96.969697	192	A	3.03030303	6		
chr1	CLCBio	SNP	22722	22722	1	C	2	C/T	86.9/11.9	73/10	84	C	86.9047619	73	T	11.9047619	10		
chr1	CLCBio	SNP	24386	24386	1	G	2	G/A	61.5/38.5	8/5	13	G	61.5384615	8	A	38.4615385	5		
chr1	CLCBio	SNP	24406	24406	1	G	2	G/T	50.0/45.8	12/11	24	G	50	12	T	45.8333333	11		
chr1	CLCBio	SNP	25437	25437	1	T	2	T/C	59.4/40.6	107/73	180	T	59.4444444	107	C	40.5555556	73		
chr1	CLCBio	SNP	25446	25446	1	T	2	T/C	50.5/49.5	141/138	279	T	50.5376344	141	C	49.4623656	138		
chr1	CLCBio	SNP	25447	25447	1	G	2	G/A	97.7/2.3	377/9	386	G	97.6683938	377	A	2.33160622	9		
chr1	CLCBio	SNP	25453	25453	1	A	2	A/G	96.8/3.2	274/9	283	A	96.819788	274	G	3.18021201	9		
chr1	CLCBio	SNP	25457	25457	1	C	2	C/T	98.6/1.4	436/6	442	C	98.6425339	436	T	1.35746606	6		
chr1	CLCBio	SNP	25468	25468	1	G	2	G/C	98.9/1.1	448/5	453	G	98.8962472	448	C	1.10375276	5		
chr1	CLCBio	SNP	25477	25477	1	A	2	A/T	98.2/1.8	600/11	611	A	98.1996727	600	T	1.80032733	11		
chr1	CLCBio	SNP	25478	25478	1	G	2	G/A	99.2/0.8	595/5	600	G	99.1666667	595	A	0.83333333	5		

chr1	CLCBio	SNP	25480	25480	1	G	2	G/A	99.2/0.8	637/5	642	G	99.2211838	637	A	0.7788162	5		
chr1	CLCBio	SNP	25485	25485	1	C	2	C/G	99.1/0.9	570/5	575	C	99.1304348	570	G	0.86956522	5		
chr1	CLCBio	Complex SNP	25486	25486	1	T	3	C/T/G	56.4/40.2/3.1	275/196/15	488	C	56.352459	275	T	40.1639344	196		
chr1	CLCBio	SNP	25487	25487	1	G	2	G/A	96.9/3.1	436/14	450	G	96.8888889	436	A	3.11111111	14		
chr1	CLCBio	SNP	25489	25489	1	T	2	T/C	96.9/2.6	403/11	416	T	96.875	403	C	2.64423077	11		
chr1	CLCBio	SNP	25490	25490	1	A	2	A/G	94.7/5.3	378/21	399	A	94.7368421	378	G	5.26315789	21		
chr1	CLCBio	SNP	25491	25491	1	T	2	T/C	94.9/4.6	542/26	571	T	94.9211909	542	C	4.55341506	26		
chr1	CLCBio	SNP	25493	25493	1	T	2	T/C	98.6/1.4	492/7	499	T	98.5971944	492	C	1.40280561	7		
chr1	CLCBio	SNP	25499	25499	1	C	2	C/G	98.6/1.2	567/7	575	C	98.6086957	567	G	1.2173913	7		
chr1	CLCBio	SNP	25500	25500	1	T	2	T/C	95.5/4.5	644/30	674	T	95.5489614	644	C	4.45103858	30		
chr1	CLCBio	SNP	25506	25506	1	T	2	T/C	98.8/0.9	681/6	689	T	98.838897	681	C	0.87082729	6		
chr1	CLCBio	SNP	25510	25510	1	A	2	A/G	99.0/1.0	666/7	673	A	98.9598811	666	G	1.04011887	7		
chr1	CLCBio	SNP	25515	25515	1	A	2	A/G	98.7/1.3	534/7	541	A	98.7060998	534	G	1.29390018	7		
chr1	CLCBio	SNP	25516	25516	1	C	2	C/G	98.9/1.0	617/6	624	C	98.8782051	617	G	0.96153846	6		
chr1	CLCBio	Complex SNP	25517	25517	1	A	3	A/C/G	95.3/2.3/2.3	409/10/10	429	A	95.3379953	409	C	2.33100233	10		
chr1	CLCBio	SNP	25525	25525	1	G	2	G/A	98.6/1.4	632/9	641	G	98.5959438	632	A	1.40405616	9		
chr1	CLCBio	SNP	25526	25526	1	T	2	T/C	98.7/1.3	625/8	633	T	98.7361769	625	C	1.26382306	8		
chr1	CLCBio	SNP	25528	25528	1	C	2	C/G	98.2/1.7	690/12	703	C	98.1507824	690	G	1.70697013	12		
chr1	CLCBio	SNP	25538	25538	1	T	2	T/C	84.9/15.1	583/104	687	T	84.8617176	583	C	15.1382824	104		
chr1	CLCBio	SNP	25547	25547	1	G	2	G/A	98.9/1.1	659/7	666	G	98.9489489	659	A	1.05105105	7		
chr1	CLCBio	SNP	25553	25553	1	G	2	G/A	80.7/19.3	421/101	522	G	80.651341	421	A	19.348659	101		
chr1	CLCBio	SNP	25566	25566	1	A	2	A/T	67.8/32.2	306/145	451	A	67.8492239	306	T	32.1507761	145		
chr1	CLCBio	SNP	25580	25580	1	A	2	A/G	98.1/1.9	565/11	576	A	98.0902778	565	G	1.90972222	11		
chr1	CLCBio	Complex SNP	25586	25586	1	C	3	C/T/G	67.6/30.1/2.2	332/148/11	491	C	67.6171079	332	T	30.1425662	148		
chr1	CLCBio	Complex SNP	25594	25594	1	G	3	G/C/T	99.1/0.5/0.4	1194/6/5	1205	G	99.0871369	1194	C	0.49792531	6		
chr1	CLCBio	SNP	25595	25595	1	T	2	T/C	98.8/1.2	1240/15	1255	T	98.8047809	1240	C	1.19521912	15		
chr1	CLCBio	SNP	25596	25596	1	T	2	T/C	99.1/0.9	540/5	545	T	99.0825688	540	C	0.91743119	5		
chr1	CLCBio	SNP	25598	25598	1	A	2	A/T	99.0/1.0	898/9	907	A	99.0077178	898	T	0.99228225	9		
chr1	CLCBio	SNP	25607	25607	1	T	2	T/C	99.3/0.4	1222/5	1230	T	99.3495935	1222	C	0.40650407	5		
chr1	CLCBio	SNP	25609	25609	1	C	2	C/T	99.5/0.5	1060/5	1065	C	99.5305164	1060	T	0.46948357	5		
chr1	CLCBio	SNP	25610	25610	1	C	2	C/T	98.2/1.8	1376/25	1401	C	98.2155603	1376	T	1.78443969	25		
chr1	CLCBio	SNP	25614	25614	1	C	2	C/T	99.3/0.7	1009/7	1016	C	99.3110236	1009	T	0.68897638	7		
chr1	CLCBio	SNP	25616	25616	1	A	2	A/G	98.4/1.6	1294/21	1315	A	98.4030418	1294	G	1.59695817	21		
chr1	CLCBio	SNP	25618	25618	1	T	2	T/G	98.4/1.6	836/14	850	T	98.3529412	836	G	1.64705882	14		
chr1	CLCBio	SNP	25620	25620	1	T	2	T/C	99.1/0.9	995/9	1004	T	99.1035857	995	C	0.89641434	9		
chr1	CLCBio	SNP	25621	25621	1	T	2	T/C	97.3/2.7	1285/35	1320	T	97.3484848	1285	C	2.65151515	35		
chr1	CLCBio	SNP	25623	25623	1	C	2	C/T	98.1/1.9	869/17	886	C	98.0812641	869	T	1.91873589	17		
chr1	CLCBio	SNP	25626	25626	1	T	2	T/C	99.4/0.6	1121/7	1128	T	99.3794326	1121	C	0.62056738	7		
chr1	CLCBio	SNP	25630	25630	1	A	2	A/T	98.9/1.1	809/9	818	A	98.8997555	809	T	1.1002445	9		
chr1	CLCBio	SNP	25631	25631	1	A	2	A/G	98.8/1.2	916/11	927	A	98.8133765	916	G	1.18662352	11		
chr1	CLCBio	SNP	25633	25633	1	A	2	A/C	98.9/1.1	438/5	443	A	98.8713318	438	C	1.12866817	5		
chr1	CLCBio	SNP	25634	25634	1	C	2	C/G	98.7/1.3	694/9	703	C	98.7197724	694	G	1.2802276	9		
chr1	CLCBio	SNP	25640	25640	1	A	2	G/A	51.0/49.0	221/212	433	G	51.039261	221	A	48.960739	212		
chr1	CLCBio	SNP	25650	25650	1	C	2	C/T	94.5/5.5	137/8	145	C	94.4827586	137	T	5.51724138	8		
chr1	CLCBio	SNP	25696	25696	1	A	2	A/G	50.0/50.0	2/2	4	A	50	2	G	50	2		
chr1	CLCBio	SNP	25718	25718	1	T	2	T/C	80.0/20.0	4/1	5	T	80	4	C	20	1		
chr1	CLCBio	SNP	25739	25739	1	A	2	G/A	66.7/33.3	6/3	9	G	66.6666667	6	A	33.3333333	3		
chr1	CLCBio	SNP	25757	25757	1	C	2	T/C	70.3/26.6	45/17	64	T	70.3125	45	C	26.5625	17		
chr1	CLCBio	SNP	25770	25770	1	C	2	C/T	96.0/4.0	479/20	499	C	95.991984	479	T	4.00801603	20		
chr1	CLCBio	SNP	25771	25771	1	G	2	G/A	94.3/5.6	525/31	557	G	94.2549372	525	A	5.56552962	31		
chr1	CLCBio	SNP	25772	25772	1	C	2	C/T	81.8/18.2	445/99	544	C	81.8014706	445	T	18.1985294	99		
chr1	CLCBio	SNP	25773	25773	1	G	2	G/A	85.1/13.8	473/77	556	G	85.0719424	473	A	13.8489209	77		
chr1	CLCBio	SNP	25775	25775	1	G	2	G/A	98.6/1.4	717/10	727	G	98.6244842	717	A	1.37551582	10		
chr1	CLCBio	SNP	25776	25776	1	A	2	A/G	99.3/0.7	731/5	736	A	99.3206522	731	G	0.67934783	5		
chr1	CLCBio	SNP	25778	25778	1	G	2	G/A	99.3/0.7	807/6	813	G	99.2619926	807	A	0.73800738	6		
chr1	CLCBio	SNP	25780	25780	1	A	2	A/G	99.3/0.7	685/5	690	A	99.2753623	685	G	0.72463768	5		
chr1	CLCBio	SNP	25789	25789	1	A	2	A/G	99.5/0.5	1091/5	1096	A	99.5437956	1091	G	0.45620438	5		
chr1	CLCBio	Complex SNP	25794	25794	1	A	3	A/C/G	97.3/1.5/1.2	1079/17/13	1109	A	97.2948602	1079	C	1.53291253	17		
chr1	CLCBio	SNP	25798	25798	1	A	2	A/G	99.4/0.6	1284/8	1292	A	99.380805	1284	G	0.61919505	8		
chr1	CLCBio	SNP	25799	25799	1	A	2	A/G	96.7/3.3	1152/39	1191	A	96.7254408	1152	G	3.27455919	39		
chr1	CLCBio	SNP	25808	25808	1	A	2	A/C	98.5/1.5	1235/19	1254	A	98.4848485	1235	C	1.51515152	19		
chr1	CLCBio	SNP	25812	25812	1	A	2	A/C	98.9/0.9	1129/10	1142	A	98.8616462	1129	C	0.87565674	10		
chr1	CLCBio	SNP	25814	25814	1	A	2	A/T	99.6/0.4	1500/6	1506	A	99.6015936	1500	T	0.39840637	6		
chr1	CLCBio	Complex SNP	25815	25815	1	G	3	G/A/C	92.9/6.6/0.3	1375/98/5	1480	G	92.9054054	1375	A	6.62162162	98		
chr1	CLCBio	Complex SNP	25816	25816	1	G	3	G/C/T	88.1/10.6/1.3	1123/135/16	1274	G	88.1475667	1123	C	10.5965463	135		

chr1	CLCBio	SNP	25817	25817	1	T	2	T/G	96.5/3.2	1094/36	1134	T	96.4726631	1094	G	3.17460317	36		
chr1	CLCBio	SNP	25818	25818	1	A	2	A/G	97.7/2.0	1243/25	1272	A	97.7201258	1243	G	1.96540881	25		
chr1	CLCBio	SNP	25820	25820	1	G	2	C/G	69.1/30.7	998/444	1445	C	69.0657439	998	G	30.7266436	444		
chr1	CLCBio	Complex SNP	25826	25826	1	T	3	T/C/G	97.6/1.6/0.8	2046/34/16	2096	T	97.6145038	2046	C	1.6221374	34		
chr1	CLCBio	SNP	25828	25828	1	A	2	A/T	99.5/0.5	2133/10	2143	A	99.5333644	2133	T	0.46663556	10		
chr1	CLCBio	SNP	25830	25830	1	C	2	C/A	99.3/0.6	2572/16	2591	C	99.2666924	2572	A	0.61752219	16		
chr1	CLCBio	SNP	25831	25831	1	T	2	T/A	94.5/5.3	2364/133	2501	T	94.5221911	2364	A	5.31787285	133		
chr1	CLCBio	Complex SNP	25832	25832	1	G	3	G/A/C	76.0/20.8/3.2	1999/547/83	2629	G	76.0365158	1999	A	20.8063903	547		
chr1	CLCBio	SNP	25836	25836	1	C	2	C/T	94.1/5.9	2789/175	2964	C	94.0958165	2789	T	5.90418354	175		
chr1	CLCBio	Complex SNP	25837	25837	1	A	4	A/G/C	71.0/27.4/1.2/	2125/820/35/1	2995	A	70.951586	2125	G	27.3789649	820		
chr1	CLCBio	SNP	25840	25840	1	A	2	A/C	99.1/0.9	3078/27	3106	A	99.098519	3078	C	0.86928525	27		
chr1	CLCBio	Complex SNP	25842	25842	1	C	3	C/T/G	98.6/1.2/0.2	3262/40/5	3307	C	98.6392501	3262	T	1.20955549	40		
chr1	CLCBio	SNP	25843	25843	1	C	2	C/T	99.7/0.3	3471/9	3480	C	99.7413793	3471	T	0.25862069	9		
chr1	CLCBio	SNP	25844	25844	1	T	2	T/C	99.3/0.7	3661/24	3687	T	99.2948196	3661	C	0.65093572	24		
chr1	CLCBio	SNP	25845	25845	1	G	2	G/A	98.9/1.0	3358/35	3394	G	98.9393047	3358	A	1.03123159	35		
chr1	CLCBio	SNP	25847	25847	1	T	2	T/A	95.9/4.0	3661/152	3816	T	95.9381551	3661	A	3.98322851	152		
chr1	CLCBio	SNP	25851	25851	1	C	2	C/T	96.0/4.0	2917/123	3040	C	95.9539474	2917	T	4.04605263	123		
chr1	CLCBio	Complex SNP	25856	25856	1	A	3	A/G/C	99.1/0.7/0.2	3708/26/9	3743	A	99.0649212	3708	G	0.69462998	26		
chr1	CLCBio	SNP	25857	25857	1	G	2	G/A	99.7/0.3	4105/12	4117	G	99.7085256	4105	A	0.29147437	12		
chr1	CLCBio	Complex SNP	25858	25858	1	A	3	A/C/T	97.0/2.4/0.6	3134/79/19	3232	A	96.9678218	3134	C	2.44430693	79		
chr1	CLCBio	SNP	25859	25859	1	A	2	A/G	98.8/1.2	4382/53	4435	A	98.8049605	4382	G	1.19503946	53		
chr1	CLCBio	SNP	25860	25860	1	G	2	G/A	99.0/1.0	3933/39	3972	G	99.0181269	3933	A	0.98187311	39		
chr1	CLCBio	SNP	25863	25863	1	C	2	C/T	99.7/0.3	4207/11	4218	C	99.7392129	4207	T	0.2607871	11		
chr1	CLCBio	SNP	25864	25864	1	A	2	A/G	99.8/0.2	4027/9	4036	A	99.7770069	4027	G	0.22299306	9		
chr1	CLCBio	SNP	25865	25865	1	C	2	C/G	98.7/1.2	3481/44	3526	C	98.7237663	3481	G	1.24787294	44		
chr1	CLCBio	Complex SNP	25867	25867	1	T	3	T/G/C	95.8/3.3/1.0	4115/140/41	4296	T	95.7867784	4115	G	3.25884544	140		
chr1	CLCBio	SNP	25868	25868	1	T	2	T/G	99.6/0.4	3960/17	3977	T	99.5725421	3960	G	0.42745788	17		
chr1	CLCBio	SNP	25869	25869	1	G	2	G/A	99.3/0.7	3919/26	3946	G	99.3157628	3919	A	0.65889508	26		
chr1	CLCBio	SNP	25875	25875	1	A	2	A/G	95.5/4.5	2269/107	2377	A	95.4564577	2269	G	4.50147244	107		
chr1	CLCBio	SNP	25877	25877	1	C	2	C/A	99.8/0.2	2831/5	2836	C	99.8236953	2831	A	0.17630465	5		
chr1	CLCBio	SNP	25878	25878	1	A	2	A/C	99.8/0.2	2798/7	2805	A	99.7504456	2798	C	0.24955437	7		
chr1	CLCBio	SNP	25879	25879	1	A	2	A/G	97.4/2.6	3055/83	3138	A	97.3550032	3055	G	2.64499681	83		
chr1	CLCBio	SNP	25880	25880	1	T	2	T/C	98.5/1.5	2247/34	2281	T	98.5094257	2247	C	1.49057431	34		
chr1	CLCBio	Complex SNP	25882	25882	1	G	3	G/A/C	96.0/2.2/1.7	2641/60/48	2750	G	96.0363636	2641	A	2.18181818	60		
chr1	CLCBio	SNP	25886	25886	1	G	2	G/A	99.5/0.5	2058/10	2068	G	99.516441	2058	A	0.48355899	10		
chr1	CLCBio	Complex SNP	25887	25887	1	G	3	G/T/C	98.5/1.0/0.4	2026/21/9	2056	G	98.540856	2026	T	1.02140078	21		
chr1	CLCBio	SNP	25890	25890	1	C	2	C/T	98.0/2.0	1338/27	1365	C	98.021978	1338	T	1.97802198	27		
chr1	CLCBio	SNP	25891	25891	1	C	2	C/T	99.4/0.6	1559/9	1568	C	99.4260204	1559	T	0.57397959	9		
chr1	CLCBio	SNP	25895	25895	1	G	2	G/A	98.6/1.4	1028/15	1043	G	98.5618408	1028	A	1.43815916	15		
chr1	CLCBio	SNP	25897	25897	1	A	2	T/A	84.4/15.6	867/160	1027	T	84.4206426	867	A	15.5793574	160		
chr1	CLCBio	SNP	25902	25902	1	G	2	G/A	99.5/0.5	974/5	979	G	99.4892748	974	A	0.51072523	5		
chr1	CLCBio	Complex SNP	25905	25905	1	C	3	C/T/A	94.6/2.9/2.5	715/22/19	756	C	94.5767196	715	T	2.91005291	22		
chr1	CLCBio	SNP	25906	25906	1	C	2	C/T	97.5/2.5	811/21	832	C	97.4759615	811	T	2.52403846	21		
chr1	CLCBio	Complex SNP	25908	25908	1	A	3	A/G/C	94.2/3.1/2.7	980/32/28	1040	A	94.2307692	980	G	3.07692308	32		
chr1	CLCBio	SNP	25909	25909	1	T	2	T/C	97.6/2.4	775/19	794	T	97.6070529	775	C	2.3929471	19		
chr1	CLCBio	SNP	25915	25915	1	A	2	A/G	90.1/9.9	608/67	675	A	90.0740741	608	G	9.92592593	67		
chr1	CLCBio	SNP	25935	25935	1	G	2	G/A	90.5/9.5	86/9	95	G	90.5263158	86	A	9.47368421	9		
chr1	CLCBio	SNP	25940	25940	1	A	2	A/T	91.4/8.6	96/9	105	A	91.4285714	96	T	8.57142857	9		
chr1	CLCBio	SNP	26616	26616	1	G	1	C	100.0	15	15	C	100	15					
chr1	CLCBio	SNP	26646	26646	1	G	2	G/A	66.7/33.3	4/2	6	G	66.6666667	4	A	33.3333333	2		
chr1	CLCBio	SNP	26757	26757	1	C	2	C/T	75.0/25.0	6/2	8	C	75	6	T	25	2		
chr1	CLCBio	SNP	26769	26769	1	G	2	A/G	78.6/21.4	11/3	14	A	78.5714286	11	G	21.4285714	3		
chr1	CLCBio	SNP	28053	28053	1	G	2	G/T	98.3/1.7	875/15	890	G	98.3146067	875	T	1.68539326	15		
chr1	CLCBio	SNP	28432	28432	1	T	2	T/C	95.5/4.5	128/6	134	T	95.5223881	128	C	4.47761194	6		
chr1	CLCBio	SNP	28556	28556	1	A	2	A/G	97.2/2.8	1646/48	1694	A	97.1664699	1646	G	2.83353011	48		
chr1	CLCBio	Complex SNP	29399	29399	1	A	3	A/C/T	55.9/23.5/20.	19/8/7	34	A	55.8823529	19	C	23.5294118	8		
chr1	CLCBio	SNP	29909	29909	1	G	2	A/G	79.1/20.9	125/33	158	A	79.1139241	125	G	20.8860759	33		
chr1	HapMap rs8067269	SNP	29909	29909		G		A	hom										
chr1	CLCBio	SNP	30157	30157	1	A	2	A/G	83.0/17.0	39/8	47	A	82.9787234	39	G	17.0212766	8		
chr1	CLCBio	SNP	30158	30158	1	C	2	C/T	89.8/10.2	53/6	59	C	89.8305085	53	T	10.1694915	6		
chr1	CLCBio	SNP	30164	30164	1	T	2	T/C	92.7/6.1	76/5	82	T	92.6829268	76	C	6.09756098	5		
chr1	CLCBio	SNP	30577	30577	1	C	2	C/A	99.6/0.4	1181/5	1186	C	99.5784148	1181	A	0.42158516	5		
chr1	CLCBio	SNP	30618	30618	1	C	2	C/A	95.8/4.2	295/13	308	C	95.7792208	295	A	4.22077922	13		
chr1	CLCBio	SNP	31466	31466	1	C	2	C/A	99.0/1.0	566/6	572	C	98.951049	566	A	1.04895105	6		
chr1	CLCBio	SNP	31467	31467	1	A	2	A/G	98.1/1.9	369/7	376	A	98.1382979	369	G	1.86170213	7		

chr1	CLCBio	SNP	31506	31506	1	C	2	C/G	96.5/3.5	946/34	980	C	96.5306122	946	G	3.46938776	34		
chr1	CLCBio	SNP	31525	31525	1	A	2	A/T	99.1/0.9	1164/10	1174	A	99.1482112	1164	T	0.85178876	10		
chr1	CLCBio	SNP	32080	32080	1	C	2	C/A	94.7/5.3	107/6	113	C	94.6902655	107	A	5.30973451	6		
chr1	CLCBio	SNP	32848	32848	1	C	2	C/A	98.5/1.3	447/6	454	C	98.4581498	447	A	1.3215859	6		
chr1	CLCBio	SNP	33369	33369	1	A	2	A/G	66.7/33.3	6/3	9	A	66.6666667	6	G	33.3333333	3		
chr1	CLCBio	SNP	34198	34198	1	T	2	T/C	60.0/40.0	6/4	10	T	60	6	C	40	4		
chr1	CLCBio	SNP	34220	34220	1	C	2	C/T	77.8/22.2	7/2	9	C	77.7777778	7	T	22.2222222	2		
chr1	CLCBio	SNP	34712	34712	1	A	2	A/G	73.7/24.0	126/41	171	A	73.6842105	126	G	23.9766082	41		
chr1	CLCBio	SNP	34728	34728	1	C	2	C/T	80.5/18.6	182/42	226	C	80.5309735	182	T	18.5840708	42		
chr1	CLCBio	SNP	34729	34729	1	G	2	G/A	84.5/15.1	196/35	232	G	84.4827586	196	A	15.0862069	35		
chr1	CLCBio	SNP	34735	34735	1	T	2	T/C	90.4/7.4	170/14	188	T	90.4255319	170	C	7.44680851	14		
chr1	CLCBio	SNP	34736	34736	1	G	2	G/A	96.6/3.4	199/7	206	G	96.6019417	199	A	3.39805825	7		
chr1	CLCBio	SNP	34738	34738	1	G	2	G/A	97.0/2.6	226/6	233	G	96.9957082	226	A	2.5751073	6		
chr1	CLCBio	SNP	34745	34745	1	T	2	T/C	75.1/22.7	175/53	233	T	75.1072961	175	C	22.7467811	53		
chr1	CLCBio	SNP	34746	34746	1	G	2	G/A	97.6/2.4	204/5	209	G	97.6076555	204	A	2.3923445	5		
chr1	CLCBio	SNP	34757	34757	1	T	2	C/T	52.9/47.1	9/8	17	C	52.9411765	9	T	47.0588235	8		
chr1	CLCBio	SNP	34759	34759	1	T	1	C	83.3	15	18	C	83.3333333	15					
chr1	CLCBio	SNP	35617	35617	1	T	2	C/T	57.6/42.4	19/14	33	C	57.5757576	19	T	42.4242424	14		
chr1	CLCBio	SNP	35625	35625	1	G	2	C/G	66.0/30.2	35/16	53	C	66.0377358	35	G	30.1886792	16		
chr1	CLCBio	SNP	35876	35876	1	A	2	A/G	98.9/1.1	730/8	738	A	98.9159892	730	G	1.08401084	8		
chr1	CLCBio	SNP	35891	35891	1	G	2	G/T	98.8/1.2	887/11	898	G	98.7750557	887	T	1.22494432	11		
chr1	CLCBio	SNP	35986	35986	1	G	2	G/T	99.5/0.5	1038/5	1043	G	99.5206136	1038	T	0.47938639	5		
chr1	CLCBio	SNP	36190	36190	1	T	2	T/G	98.7/1.3	745/10	755	T	98.6754967	745	G	1.32450331	10		
chr1	HapMap rs28897686	SNP	36800	36800		C		G	hom										
chr1	CLCBio	SNP	36803	36803	1	T	2	T/G	98.0/2.0	240/5	245	T	97.9591837	240	G	2.04081633	5		
chr1	CLCBio	SNP	37000	37000	1	T	2	T/C	94.9/5.1	392/21	413	T	94.9152542	392	C	5.08474576	21		
chr1	CLCBio	SNP	37308	37308	1	C	2	C/A	98.9/1.1	534/6	540	C	98.8888889	534	A	1.11111111	6		
chr1	HapMap rs4986848	SNP	37582	37582		A		T	hom										
chr1	CLCBio	SNP	37936	37936	1	G	2	A/G	83.6/16.4	214/42	256	A	83.59375	214	G	16.40625	42		
chr1	HapMap rs799917	SNP	37936	37936		G		A	hom										
chr1	CLCBio	SNP	38466	38466	1	G	2	G/A	99.0/1.0	761/8	769	G	98.9596879	761	A	1.04031209	8		
chr1	CLCBio	SNP	38493	38493	1	A	2	A/T	99.6/0.4	1336/6	1342	A	99.5529061	1336	T	0.44709389	6		
chr1	CLCBio	SNP	38559	38559	1	G	2	G/T	97.1/2.9	2013/60	2073	G	97.105644	2013	T	2.89435601	60		
chr1	CLCBio	SNP	38574	38574	1	C	2	C/A	99.0/1.0	1279/13	1292	C	98.993808	1279	A	1.00619195	13		
chr1	CLCBio	SNP	39269	39269	1	C	2	C/A	96.2/3.8	152/6	158	C	96.2025316	152	A	3.79746835	6		
chr1	CLCBio	SNP	39568	39568	1	G	2	G/T	98.6/1.4	421/6	427	G	98.5948478	421	T	1.40515222	6		
chr1	CLCBio	SNP	39780	39780	1	C	2	C/A	99.0/1.0	1089/11	1100	C	99	1089	A	1	11		
chr1	CLCBio	SNP	40803	40803	1	G	2	G/T	97.2/2.8	275/8	283	G	97.1731449	275	T	2.82685512	8		
chr1	CLCBio	SNP	41027	41027	1	T	2	T/C	87.5/12.5	35/5	40	T	87.5	35	C	12.5	5		
chr1	CLCBio	SNP	43595	43595	1	T	1	C	81.8	9	11	C	81.8181818	9					
chr1	CLCBio	SNP	43615	43615	1	C	2	C/T	75.0/25.0	3/1	4	C	75	3	T	25	1		
chr1	CLCBio	SNP	43677	43677	1	C	2	T/C	57.1/42.9	12/9	21	T	57.1428571	12	C	42.8571429	9		
chr1	CLCBio	SNP	43685	43685	1	G	2	G/A	94.3/5.7	99/6	105	G	94.2857143	99	A	5.71428571	6		
chr1	CLCBio	SNP	43692	43692	1	C	2	C/T	83.1/15.6	133/25	160	C	83.125	133	T	15.625	25		
chr1	CLCBio	SNP	43693	43693	1	G	2	G/A	81.5/17.9	132/29	162	G	81.4814815	132	A	17.9012346	29		
chr1	CLCBio	Complex SNP	43697	43697	1	A	3	A/G/T	84.5/11.2/3.4	174/23/7	206	A	84.4660194	174	G	11.1650485	23		
chr1	CLCBio	SNP	43702	43702	1	G	2	G/A	92.5/6.7	235/17	254	G	92.519685	235	A	6.69291339	17		
chr1	CLCBio	SNP	43715	43715	1	G	2	G/A	97.2/2.1	275/6	283	G	97.1731449	275	A	2.12014134	6		
chr1	CLCBio	SNP	43718	43718	1	G	2	G/A	97.6/2.0	246/5	252	G	97.6190476	246	A	1.98412698	5		
chr1	CLCBio	SNP	43719	43719	1	G	2	G/A	97.9/1.8	275/5	281	G	97.8647687	275	A	1.77935943	5		
chr1	CLCBio	SNP	43729	43729	1	C	2	C/T	93.4/4.9	170/9	182	C	93.4065934	170	T	4.94505495	9		
chr1	CLCBio	SNP	43730	43730	1	G	2	G/A	82.2/14.1	134/23	163	G	82.208589	134	A	14.1104294	23		
chr1	CLCBio	SNP	43742	43742	1	G	2	G/A	91.3/8.7	105/10	115	G	91.3043478	105	A	8.69565217	10		
chr1	CLCBio	SNP	44033	44033	1	T	2	C/T	50.0/50.0	6/6	12	C	50	6	T	50	6		
chr1	CLCBio	SNP	44063	44063	1	T	2	T/C	91.4/7.6	96/8	105	T	91.4285714	96	C	7.61904762	8		
chr1	CLCBio	SNP	44074	44074	1	A	2	A/G	91.7/8.3	122/11	133	A	91.7293233	122	G	8.27067669	11		
chr1	CLCBio	SNP	44075	44075	1	T	2	T/C	91.3/8.7	115/11	126	T	91.2698413	115	C	8.73015873	11		
chr1	CLCBio	SNP	44079	44079	1	T	2	T/C	94.2/5.8	113/7	120	T	94.1666667	113	C	5.83333333	7		
chr1	CLCBio	SNP	44338	44338	1	C	2	C/A	98.6/1.4	1222/17	1239	C	98.6279257	1222	A	1.37207425	17		
chr1	CLCBio	SNP	44383	44383	1	C	2	C/A	99.4/0.6	811/5	816	C	99.3872549	811	A	0.6127451	5		
chr1	CLCBio	SNP	45164	45164	1	A	2	A/G	75.0/25.0	3/1	4	A	75	3	G	25	1		
chr1	CLCBio	SNP	45168	45168	1	T	2	T/C	75.0/25.0	3/1	4	T	75	3	C	25	1		
chr1	CLCBio	SNP	45286	45286	1	G	2	G/A	90.0/8.6	63/6	70	G	90	63	A	8.57142857	6		
chr1	CLCBio	SNP	45312	45312	1	C	2	G/C	50.5/45.2	47/42	93	G	50.5376344	47	C	45.1612903	42		
chr1	CLCBio	SNP	45627	45627	1	A	2	T/A	62.5/37.5	5/3	8	T	62.5	5	A	37.5	3		

chr1	CLCBio	SNP	45671	45671	1	A	1	T	85.0	17	20	T	85	17					
chr1	CLCBio	SNP	46350	46350	1	C	2	C/T	87.8/12.2	43/6	49	C	87.755102	43	T	12.244898	6		
chr1	CLCBio	SNP	46971	46971	1	G	2	G/A	75.0/25.0	3/1	4	G	75	3	A	25	1		
chr1	HapMap rs28897673	SNP	47280	47280		T		A	hom										
chr1	CLCBio	SNP	48172	48172	1	T	1	C	97.0	130	134	C	97.0149254	130					
chr1	HapMap rs799912	SNP	48172	48172		T		C	hom										
chr1	CLCBio	SNP	48903	48903	1	C	2	C/T	82.9/17.1	29/6	35	C	82.8571429	29	T	17.1428571	6		
chr1	CLCBio	SNP	48908	48908	1	T	2	T/C	82.9/17.1	29/6	35	T	82.8571429	29	C	17.1428571	6		
chr1	CLCBio	SNP	48920	48920	1	C	2	C/T	83.9/16.1	26/5	31	C	83.8709677	26	T	16.1290323	5		
chr1	CLCBio	SNP	48925	48925	1	C	1	G	90.3	28	31	G	90.3225806	28					
chr1	CLCBio	SNP	49536	49536	1	T	2	T/G	80.2/19.8	174/43	217	T	80.1843318	174	G	19.8156682	43		
chr1	CLCBio	SNP	50170	50170	1	G	2	G/A	96.9/2.9	495/15	511	G	96.8688845	495	A	2.93542074	15		
chr1	CLCBio	SNP	50692	50692	1	G	2	G/A	78.9/21.1	56/15	71	G	78.8732394	56	A	21.1267606	15		
chr1	CLCBio	SNP	50891	50891	1	A	2	A/C	75.0/25.0	3/1	4	A	75	3	C	25	1		
chr1	CLCBio	SNP	50898	50898	1	C	2	C/A	80.0/20.0	4/1	5	C	80	4	A	20	1		
chr1	CLCBio	SNP	51185	51185	1	A	2	T/A	91.1/8.9	368/36	404	T	91.0891089	368	A	8.91089109	36		
chr1	HapMap rs8065872	SNP	51185	51185		A		T	hom										
chr1	CLCBio	SNP	51564	51564	1	C	2	C/T	75.0/25.0	3/1	4	C	75	3	T	25	1		
chr1	CLCBio	SNP	51573	51573	1	T	2	T/A	80.0/20.0	4/1	5	T	80	4	A	20	1		
chr1	CLCBio	SNP	51575	51575	1	T	2	T/C	75.0/25.0	3/1	4	T	75	3	C	25	1		
chr1	CLCBio	SNP	51583	51583	1	G	2	G/C	60.0/40.0	3/2	5	G	60	3	C	40	2		
chr1	CLCBio	SNP	51676	51676	1	C	2	C/T	80.0/20.0	4/1	5	C	80	4	T	20	1		
chr1	CLCBio	SNP	51710	51710	1	C	2	C/T	92.6/6.6	113/8	122	C	92.6229508	113	T	6.55737705	8		
chr1	CLCBio	SNP	51711	51711	1	G	2	G/A	89.6/8.1	121/11	135	G	89.6296296	121	A	8.14814815	11		
chr1	CLCBio	SNP	51720	51720	1	C	2	C/T	90.8/9.2	129/13	142	C	90.8450704	129	T	9.15492958	13		
chr1	CLCBio	SNP	51726	51726	1	C	2	C/T	88.0/11.1	103/13	117	C	88.034188	103	T	11.1111111	13		
chr1	CLCBio	SNP	51727	51727	1	G	2	G/A	92.9/5.9	79/5	85	G	92.9411765	79	A	5.88235294	5		
chr1	CLCBio	SNP	52281	52281	1	A	2	A/G	62.5/37.5	5/3	8	A	62.5	5	G	37.5	3		
chr1	CLCBio	SNP	52290	52290	1	C	2	C/A	77.8/22.2	7/2	9	C	77.7777778	7	A	22.2222222	2		
chr1	CLCBio	SNP	52983	52983	1	G	2	G/A	87.3/11.9	110/15	126	G	87.3015873	110	A	11.9047619	15		
chr1	CLCBio	SNP	52989	52989	1	C	2	C/T	89.7/8.5	191/18	213	C	89.6713615	191	T	8.45070423	18		
chr1	CLCBio	Complex SNP	52990	52990	1	G	3	G/A/T	69.0/26.4/4.6	149/57/10	216	G	68.9814815	149	A	26.3888889	57		
chr1	CLCBio	Complex SNP	52994	52994	1	A	3	A/G/T	92.4/4.5/2.3	244/12/6	264	A	92.4242424	244	G	4.54545455	12		
chr1	CLCBio	SNP	53013	53013	1	C	2	C/T	97.3/1.7	284/5	292	C	97.260274	284	T	1.71232877	5		
chr1	CLCBio	SNP	53016	53016	1	G	2	G/A	97.3/1.8	326/6	335	G	97.3134328	326	A	1.79104478	6		
chr1	CLCBio	SNP	53022	53022	1	C	2	C/T	97.8/1.6	307/5	314	C	97.7707006	307	T	1.59235669	5		
chr1	CLCBio	SNP	53024	53024	1	G	2	G/A	97.4/2.2	263/6	270	G	97.4074074	263	A	2.22222222	6		
chr1	CLCBio	SNP	53025	53025	1	G	2	G/A	96.4/3.2	212/7	220	G	96.3636364	212	A	3.18181818	7		
chr1	CLCBio	SNP	53026	53026	1	C	2	C/T	88.9/10.7	208/25	234	C	88.8888889	208	T	10.6837607	25		
chr1	CLCBio	SNP	53027	53027	1	G	2	G/A	91.0/8.1	201/18	221	G	90.9502262	201	A	8.14479638	18		
chr1	CLCBio	SNP	54682	54682	1	G	2	G/A	76.7/23.3	23/7	30	G	76.6666667	23	A	23.3333333	7		
chr1	CLCBio	SNP	55494	55494	1	C	2	C/T	88.6/10.0	62/7	70	C	88.5714286	62	T	10	7		
chr1	CLCBio	Complex SNP	55509	55509	1	A	3	C/A/T	48.1/40.3/11.	37/31/9	77	C	48.0519481	37	A	40.2597403	31		
chr1	CLCBio	SNP	55522	55522	1	C	2	C/T	90.8/7.9	69/6	76	C	90.7894737	69	T	7.89473684	6		
chr1	CLCBio	SNP	55523	55523	1	G	2	G/A	93.0/7.0	66/5	71	G	92.9577465	66	A	7.04225352	5		
chr1	CLCBio	SNP	55539	55539	1	A	2	A/G	72.7/27.3	8/3	11	A	72.7272727	8	G	27.2727273	3		
chr1	CLCBio	SNP	55602	55602	1	A	2	A/G	78.6/21.4	22/6	28	A	78.5714286	22	G	21.4285714	6		
chr1	CLCBio	SNP	55640	55640	1	G	2	G/A	69.6/30.4	16/7	23	G	69.5652174	16	A	30.4347826	7		
chr1	CLCBio	SNP	55646	55646	1	T	2	T/C	66.7/25.0	8/3	12	T	66.6666667	8	C	25	3		
chr1	CLCBio	SNP	55647	55647	1	T	2	T/C	77.8/22.2	7/2	9	T	77.7777778	7	C	22.2222222	2		
chr1	CLCBio	SNP	57180	57180	1	T	2	T/C	59.1/40.9	26/18	44	T	59.0909091	26	C	40.9090909	18		
chr1	CLCBio	SNP	57213	57213	1	C	2	C/T	64.7/35.3	11/6	17	C	64.7058824	11	T	35.2941176	6		
chr1	CLCBio	SNP	57345	57345	1	C	2	C/T	81.4/18.6	83/19	102	C	81.372549	83	T	18.627451	19		
chr1	CLCBio	SNP	57362	57362	1	A	2	A/C	69.1/30.9	170/76	246	A	69.1056911	170	C	30.8943089	76		
chr1	CLCBio	SNP	57411	57411	1	T	2	T/G	91.2/8.8	166/16	182	T	91.2087912	166	G	8.79120879	16		
chr1	CLCBio	SNP	57414	57414	1	G	2	G/A	86.9/13.1	106/16	122	G	86.8852459	106	A	13.1147541	16		
chr1	CLCBio	SNP	57701	57701	1	A	2	A/G	75.0/25.0	3/1	4	A	75	3	G	25	1		
chr1	CLCBio	Complex SNP	57703	57703	1	C	3	C/A/T	50.0/25.0/25.	2/1/1	4	C	50	2	A	25	1		
chr1	CLCBio	SNP	58220	58220	1	T	2	T/C	75.0/25.0	3/1	4	T	75	3	C	25	1		
chr1	CLCBio	SNP	58678	58678	1	A	2	A/T	98.9/1.1	709/8	717	A	98.8842399	709	T	1.11576011	8		
chr1	CLCBio	SNP	58714	58714	1	A	2	A/G	95.7/4.3	473/21	494	A	95.7489879	473	G	4.25101215	21		
chr1	CLCBio	SNP	58726	58726	1	G	2	G/A	99.0/1.0	1443/14	1458	G	98.9711934	1443	A	0.96021948	14		
chr1	CLCBio	SNP	58978	58978	1	C	2	C/T	86.4/13.6	146/23	169	C	86.3905325	146	T	13.6094675	23		
chr1	CLCBio	SNP	59034	59034	1	C	2	C/T	93.9/6.1	92/6	98	C	93.877551	92	T	6.12244898	6		
chr1	CLCBio	SNP	59040	59040	1	T	2	T/G	94.4/5.6	102/6	108	T	94.4444444	102	G	5.55555556	6		

chr1	CLCBio	SNP	59110	59110	1	A	2	A/G	84.3/15.7	59/11	70	A	84.2857143	59	G	15.7142857	11		
chr1	CLCBio	SNP	59462	59462	1	C	2	C/A	86.1/13.9	216/35	251	C	86.0557769	216	A	13.9442231	35		
chr1	CLCBio	SNP	59648	59648	1	C	2	C/T	94.2/5.2	290/16	308	C	94.1558442	290	T	5.19480519	16		
chr1	CLCBio	Complex SNP	59649	59649	1	G	3	G/A/T	96.3/1.8/1.1	420/8/5	436	G	96.3302752	420	A	1.83486239	8		
chr1	CLCBio	SNP	59652	59652	1	C	2	C/T	98.8/1.1	564/6	571	C	98.7740806	564	T	1.05078809	6		
chr1	CLCBio	SNP	59653	59653	1	G	2	G/A	91.9/8.0	485/42	528	G	91.8560606	485	A	7.95454545	42		
chr1	CLCBio	SNP	59654	59654	1	C	2	C/T	96.9/2.5	657/17	678	C	96.9026549	657	T	2.50737463	17		
chr1	CLCBio	SNP	59655	59655	1	G	2	G/A	97.2/2.2	627/14	645	G	97.2093023	627	A	2.17054264	14		
chr1	CLCBio	SNP	59660	59660	1	C	2	C/T	99.0/0.8	624/5	630	C	99.047619	624	T	0.79365079	5		
chr1	CLCBio	SNP	59662	59662	1	C	2	C/T	99.3/0.7	677/5	682	C	99.2668622	677	T	0.73313783	5		
chr1	CLCBio	Complex SNP	59664	59664	1	C	3	C/T/A	87.9/10.2/1.7	580/67/11	660	C	87.8787879	580	T	10.1515152	67		
chr1	CLCBio	Complex SNP	59665	59665	1	G	3	G/A/T	94.7/4.1/1.0	647/28/7	683	G	94.7291362	647	A	4.09956076	28		
chr1	CLCBio	SNP	59666	59666	1	C	2	C/T	97.8/1.7	589/10	602	C	97.8405316	589	T	1.66112957	10		
chr1	CLCBio	SNP	59667	59667	1	C	2	C/T	97.9/2.0	737/15	753	C	97.875166	737	T	1.99203187	15		
chr1	CLCBio	SNP	59669	59669	1	G	2	G/A	98.8/1.1	906/10	917	G	98.8004362	906	A	1.09051254	10		
chr1	CLCBio	SNP	59671	59671	1	A	2	A/C	98.9/0.9	640/6	647	A	98.9180835	640	C	0.92735703	6		
chr1	CLCBio	Complex SNP	59674	59674	1	C	4	C/A/G	98.3/0.6/0.6/0	890/5/5/5	905	C	98.3425414	890	A	0.55248619	5		
chr1	CLCBio	SNP	59675	59675	1	C	2	C/T	98.6/1.1	898/10	911	C	98.5729967	898	T	1.09769484	10		
chr1	CLCBio	SNP	59676	59676	1	C	2	C/T	97.6/2.1	798/17	818	C	97.5550122	798	T	2.07823961	17		
chr1	CLCBio	SNP	59679	59679	1	C	2	C/A	98.8/0.6	916/6	927	C	98.8133765	916	A	0.64724919	6		
chr1	CLCBio	SNP	59683	59683	1	T	2	T/C	98.5/0.9	538/5	546	T	98.5347985	538	C	0.91575092	5		
chr1	CLCBio	SNP	59686	59686	1	G	2	G/A	99.2/0.8	716/6	722	G	99.1689751	716	A	0.83102493	6		
chr1	CLCBio	SNP	59687	59687	1	G	2	G/A	97.6/1.7	573/10	587	G	97.6149915	573	A	1.70357751	10		
chr1	CLCBio	SNP	59689	59689	1	G	2	G/A	99.1/0.7	738/5	745	G	99.0604027	738	A	0.67114094	5		
chr1	CLCBio	SNP	59691	59691	1	C	2	C/T	98.8/1.2	413/5	418	C	98.8038278	413	T	1.19617225	5		
chr1	CLCBio	SNP	59693	59693	1	G	2	G/A	97.6/1.8	479/9	491	G	97.5560081	479	A	1.83299389	9		
chr1	CLCBio	Complex SNP	59697	59697	1	T	3	T/C/A	76.0/21.4/2.6	291/82/10	383	T	75.9791123	291	C	21.4099217	82		
chr1	CLCBio	SNP	59701	59701	1	C	2	C/T	86.9/11.3	246/32	283	C	86.9257951	246	T	11.3074205	32		
chr1	CLCBio	SNP	59702	59702	1	G	2	G/A	92.3/7.7	252/21	273	G	92.3076923	252	A	7.69230769	21		
chr1	CLCBio	SNP	59708	59708	1	C	2	C/T	90.6/9.4	183/19	202	C	90.5940594	183	T	9.40594059	19		
chr1	CLCBio	SNP	59709	59709	1	G	2	G/A	97.4/2.6	184/5	189	G	97.3544974	184	A	2.64550265	5		
chr1	CLCBio	SNP	59773	59773	1	G	2	G/A	80.0/20.0	4/1	5	G	80	4	A	20	1		
chr1	CLCBio	SNP	59795	59795	1	G	2	C/G	56.2/37.5	18/12	32	C	56.25	18	G	37.5	12		
chr1	CLCBio	SNP	59798	59798	1	G	2	G/A	83.3/16.7	35/7	42	G	83.3333333	35	A	16.6666667	7		
chr1	CLCBio	SNP	59828	59828	1	C	2	C/G	44.4/38.9	8/7	18	C	44.4444444	8	G	38.8888889	7		
chr1	CLCBio	SNP	59886	59886	1	A	2	A/C	60.0/40.0	3/2	5	A	60	3	C	40	2		
chr1	CLCBio	SNP	59907	59907	1	A	2	G/A	70.0/30.0	14/6	20	G	70	14	A	30	6		
chr1	CLCBio	SNP	60381	60381	1	A	2	A/G	57.3/42.7	98/73	171	A	57.3099415	98	G	42.6900585	73		
chr1	CLCBio	SNP	60396	60396	1	G	2	G/A	89.6/10.4	121/14	135	G	89.6296296	121	A	10.3703704	14		
chr1	CLCBio	SNP	60477	60477	1	G	2	G/T	95.8/4.2	226/10	236	G	95.7627119	226	T	4.23728814	10		
chr1	CLCBio	SNP	61250	61250	1	G	2	G/A	95.0/5.0	209/11	220	G	95	209	A	5	11		
chr1	CLCBio	SNP	61348	61348	1	A	2	A/G	85.7/14.3	677/113	790	A	85.6962025	677	G	14.3037975	113		
chr1	CLCBio	SNP	61390	61390	1	A	2	A/T	77.0/23.0	274/82	356	A	76.9662921	274	T	23.0337079	82		
chr1	CLCBio	SNP	61403	61403	1	T	2	T/C	77.4/22.6	164/48	212	T	77.3584906	164	C	22.6415094	48		
chr1	CLCBio	SNP	61477	61477	1	A	2	A/T	96.3/3.7	184/7	191	A	96.3350785	184	T	3.66492147	7		
chr1	CLCBio	SNP	61478	61478	1	G	2	G/C	94.8/5.2	127/7	134	G	94.7761194	127	C	5.2238806	7		
chr1	CLCBio	SNP	61549	61549	1	C	2	C/T	98.5/1.5	465/7	472	C	98.5169492	465	T	1.48305085	7		
chr1	CLCBio	SNP	61972	61972	1	T	2	T/C	92.5/7.5	683/55	738	T	92.5474255	683	C	7.45257453	55		
chr1	CLCBio	SNP	61979	61979	1	T	2	T/C	69.4/30.6	343/151	494	T	69.4331984	343	C	30.5668016	151		
chr1	CLCBio	SNP	62023	62023	1	A	2	A/G	95.8/4.2	1379/61	1440	A	95.7638889	1379	G	4.23611111	61		
chr1	CLCBio	SNP	62046	62046	1	C	2	C/T	97.3/2.7	1834/51	1885	C	97.2944297	1834	T	2.70557029	51		
chr1	CLCBio	SNP	62054	62054	1	A	2	A/G	98.8/1.2	1582/19	1601	A	98.8132417	1582	G	1.18675828	19		
chr1	CLCBio	SNP	62082	62082	1	T	2	T/A	95.2/4.8	1865/95	1960	T	95.1530612	1865	A	4.84693878	95		
chr1	CLCBio	SNP	62089	62089	1	T	2	T/C	89.6/10.4	2091/243	2334	T	89.5886889	2091	C	10.4113111	243		
chr1	CLCBio	SNP	62163	62163	1	G	2	G/A	81.2/18.8	621/144	765	G	81.1764706	621	A	18.8235294	144		
chr1	CLCBio	SNP	62166	62166	1	G	2	G/A	78.3/21.7	490/136	626	G	78.2747604	490	A	21.7252396	136		
chr1	CLCBio	SNP	62207	62207	1	C	2	C/A	99.1/0.9	1213/11	1224	C	99.1013072	1213	A	0.89869281	11		
chr1	CLCBio	SNP	62242	62242	1	C	2	C/T	96.0/4.0	1502/63	1565	C	95.9744409	1502	T	4.02555911	63		
chr1	CLCBio	SNP	62252	62252	1	A	2	A/G	98.4/1.6	1231/20	1251	A	98.401279	1231	G	1.59872102	20		
chr1	CLCBio	SNP	62543	62543	1	C	2	C/A	99.5/0.5	1514/7	1521	C	99.5397765	1514	A	0.46022354	7		
chr1	CLCBio	SNP	62635	62635	1	C	2	C/A	99.2/0.8	620/5	625	C	99.2	620	A	0.8	5		
chr1	CLCBio	SNP	62663	62663	1	G	2	G/C	58.7/41.3	750/528	1278	G	58.685446	750	C	41.314554	528		
chr1	CLCBio	SNP	62681	62681	1	G	2	G/A	99.4/0.5	1802/9	1812	G	99.4481236	1802	A	0.49668874	9		
chr1	CLCBio	SNP	62683	62683	1	C	2	C/A	99.5/0.5	1997/11	2008	C	99.4521912	1997	A	0.54780876	11		
chr1	CLCBio	SNP	62785	62785	1	A	2	A/C	99.0/1.0	853/9	862	A	98.9559165	853	C	1.04408353	9		

chr1	CLCBio	SNP	62789	62789	1	C	2	C/A	99.2/0.8	597/5	602	C	99.1694352	597	A	0.83056478	5		
chr1	CLCBio	SNP	62799	62799	1	G	2	G/C	96.7/3.2	786/26	813	G	96.6789668	786	C	3.19803198	26		
chr1	CLCBio	SNP	62829	62829	1	A	2	A/G	96.3/3.7	619/24	643	A	96.2674961	619	G	3.73250389	24		
chr1	CLCBio	SNP	62954	62954	1	C	2	C/A	99.1/0.9	544/5	549	C	99.0892532	544	A	0.91074681	5		
chr1	CLCBio	SNP	63111	63111	1	C	2	C/T	99.2/0.8	602/5	607	C	99.1762768	602	T	0.82372323	5		
chr1	CLCBio	SNP	63122	63122	1	G	2	G/T	98.1/1.9	632/12	644	G	98.136646	632	T	1.86335404	12		
chr1	CLCBio	SNP	63351	63351	1	G	2	G/C	97.3/2.7	550/15	565	G	97.3451327	550	C	2.65486726	15		
chr1	CLCBio	SNP	63360	63360	1	A	2	A/G	84.0/16.0	440/84	524	A	83.9694656	440	G	16.0305344	84		
chr1	CLCBio	SNP	63398	63398	1	G	2	G/A	63.5/36.5	434/250	684	G	63.4502924	434	A	36.5497076	250		
chr1	CLCBio	SNP	63406	63406	1	A	2	A/G	99.0/1.0	605/6	611	A	99.0180033	605	G	0.98199673	6		
chr1	CLCBio	SNP	63592	63592	1	T	2	T/C	63.1/36.9	70/41	111	T	63.0630631	70	C	36.9369369	41		
chr1	CLCBio	SNP	63624	63624	1	A	2	A/G	92.3/7.7	143/12	155	A	92.2580645	143	G	7.74193548	12		
chr1	CLCBio	SNP	63644	63644	1	C	2	C/A	95.4/4.6	208/10	218	C	95.412844	208	A	4.58715596	10		
chr1	CLCBio	SNP	63698	63698	1	C	2	C/A	97.9/2.1	743/16	759	C	97.8919631	743	A	2.10803689	16		
chr1	CLCBio	SNP	63853	63853	1	G	2	G/A	70.2/29.8	1231/522	1753	G	70.2224758	1231	A	29.7775242	522		
chr1	CLCBio	SNP	63880	63880	1	A	2	A/G	99.0/1.0	1755/17	1772	A	99.0406321	1755	G	0.95936795	17		
chr1	CLCBio	SNP	63988	63988	1	A	2	A/G	95.7/4.3	928/42	970	A	95.6701031	928	G	4.32989691	42		
chr1	CLCBio	SNP	64003	64003	1	A	2	A/G	91.0/9.0	810/80	890	A	91.011236	810	G	8.98876404	80		
chr1	CLCBio	SNP	64036	64036	1	G	2	G/T	96.6/3.4	595/21	616	G	96.5909091	595	T	3.40909091	21		
chr1	CLCBio	SNP	64095	64095	1	C	2	C/A	98.1/1.9	665/13	678	C	98.0825959	665	A	1.91740413	13		
chr1	CLCBio	SNP	64363	64363	1	T	2	T/C	98.2/1.8	372/7	379	T	98.1530343	372	C	1.8469657	7		
chr1	CLCBio	SNP	64413	64413	1	C	2	C/A	99.2/0.8	859/7	866	C	99.1916859	859	A	0.80831409	7		
chr1	CLCBio	SNP	64497	64497	1	C	2	C/A	99.3/0.6	2196/14	2211	C	99.3215739	2196	A	0.63319765	14		
chr1	CLCBio	SNP	64661	64661	1	G	2	G/A	94.4/5.6	656/39	695	G	94.3884892	656	A	5.61151079	39		
chr1	CLCBio	SNP	64702	64702	1	G	2	G/A	97.3/2.7	181/5	186	G	97.311828	181	A	2.68817204	5		
chr1	CLCBio	SNP	64883	64883	1	G	2	G/A	87.5/12.5	42/6	48	G	87.5	42	A	12.5	6		
chr1	CLCBio	SNP	64961	64961	1	G	2	G/A	98.5/1.5	322/5	327	G	98.470948	322	A	1.52905199	5		
chr1	CLCBio	SNP	65133	65133	1	G	2	G/T	99.2/0.8	1150/9	1159	G	99.2234685	1150	T	0.77653149	9		
chr1	CLCBio	SNP	65266	65266	1	G	2	G/A	78.1/21.9	82/23	105	G	78.0952381	82	A	21.9047619	23		
chr1	HapMap rs206119	SNP	65266	65266		G		A	hom										
chr1	CLCBio	SNP	65324	65324	1	C	2	C/T	52.7/47.0	287/256	545	C	52.6605505	287	T	46.9724771	256		
chr1	HapMap rs9562605	SNP	65324	65324		C		T	het										
chr1	CLCBio	SNP	65460	65460	1	C	2	C/A	96.4/3.6	186/7	193	C	96.373057	186	A	3.62694301	7		
chr1	CLCBio	SNP	65468	65468	1	C	2	C/A	97.4/2.6	185/5	190	C	97.3684211	185	A	2.63157895	5		
chr1	CLCBio	SNP	65504	65504	1	G	2	G/T	96.1/3.9	418/17	435	G	96.091954	418	T	3.90804598	17		
chr1	CLCBio	SNP	65525	65525	1	G	2	G/T	83.7/16.3	339/66	405	G	83.7037037	339	T	16.2962963	66		
chr1	CLCBio	SNP	66887	66887	1	C	2	C/G	60.0/40.0	3/2	5	C	60	3	G	40	2		
chr1	CLCBio	SNP	66914	66914	1	A	2	A/G	63.6/36.4	7/4	11	A	63.6363636	7	G	36.3636364	4		
chr1	CLCBio	SNP	66925	66925	1	T	2	T/C	71.4/28.6	5/2	7	T	71.4285714	5	C	28.5714286	2		
chr1	CLCBio	SNP	67002	67002	1	T	2	C/T	62.5/37.5	5/3	8	C	62.5	5	T	37.5	3		
chr1	CLCBio	SNP	67023	67023	1	T	2	T/A	80.0/20.0	4/1	5	T	80	4	A	20	1		
chr1	CLCBio	SNP	67595	67595	1	G	2	G/A	98.8/1.2	477/6	483	G	98.757764	477	A	1.24223602	6		
chr1	CLCBio	SNP	67886	67886	1	A	2	A/G	78.8/21.2	156/42	198	A	78.7878788	156	G	21.2121212	42		
chr1	CLCBio	SNP	67973	67973	1	A	1	G	100.0	85	85	G	100	85					
chr1	HapMap rs9534174	SNP	67973	67973		A		G	hom										
chr1	CLCBio	SNP	68003	68003	1	C	2	C/T	98.1/1.9	264/5	269	C	98.1412639	264	T	1.85873606	5		
chr1	CLCBio	SNP	68005	68005	1	A	2	A/G	54.1/45.9	158/134	292	A	54.109589	158	G	45.890411	134		
chr1	CLCBio	SNP	68090	68090	1	A	2	G/A	52.3/47.7	138/126	264	G	52.2727273	138	A	47.7272727	126		
chr1	CLCBio	SNP	69217	69217	1	G	1	C	100.0	4	4	C	100	4					
chr1	HapMap rs206123	SNP	69217	69217		g		C	hom										
chr1	CLCBio	SNP	70212	70212	1	T	2	T/C	66.7/33.3	14/7	21	T	66.6666667	14	C	33.3333333	7		
chr1	CLCBio	SNP	71934	71934	1	C	2	C/T	78.6/21.4	11/3	14	C	78.5714286	11	T	21.4285714	3		
chr1	CLCBio	SNP	72325	72325	1	C	2	C/T	80.0/20.0	4/1	5	C	80	4	T	20	1		
chr1	CLCBio	SNP	73043	73043	1	C	2	C/A	98.8/1.2	423/5	428	C	98.8317757	423	A	1.1682243	5		
chr1	CLCBio	SNP	73315	73315	1	T	2	T/A	75.9/24.1	22/7	29	T	75.862069	22	A	24.137931	7		
chr1	CLCBio	SNP	73736	73736	1	A	2	A/G	50.0/50.0	2/2	4	A	50	2	G	50	2		
chr1	CLCBio	SNP	73737	73737	1	A	2	A/C	80.0/20.0	4/1	5	A	80	4	C	20	1		
chr1	CLCBio	SNP	73841	73841	1	A	2	A/G	77.0/19.7	47/12	61	A	77.0491803	47	G	19.6721311	12		
chr1	CLCBio	SNP	73849	73849	1	A	2	A/G	82.6/15.9	57/11	69	A	82.6086957	57	G	15.942029	11		
chr1	CLCBio	SNP	73865	73865	1	A	2	A/C	76.5/23.5	13/4	17	A	76.4705882	13	C	23.5294118	4		
chr1	CLCBio	SNP	76478	76478	1	C	2	G/C	72.4/20.7	21/6	29	G	72.4137931	21	C	20.6896552	6		
chr1	CLCBio	SNP	76479	76479	1	A	2	G/A	72.0/28.0	18/7	25	G	72	18	A	28	7		
chr1	CLCBio	SNP	76666	76666	1	G	2	T/G	55.6/44.4	5/4	9	T	55.5555556	5	G	44.4444444	4		
chr1	CLCBio	SNP	76677	76677	1	G	2	G/A	77.8/22.2	7/2	9	G	77.7777778	7	A	22.2222222	2		
chr1	CLCBio	SNP	76717	76717	1	A	2	A/G	57.1/28.6	4/2	7	A	57.1428571	4	G	28.5714286	2		

chr1	CLCBio	SNP	77977	77977	1	A	2	A/C	92.7/7.3	228/18	246	A	92.6829268	228	C	7.31707317	18		
chr1	CLCBio	SNP	78340	78340	1	C	2	C/A	98.5/1.5	446/7	453	C	98.4547461	446	A	1.54525386	7		
chr1	CLCBio	SNP	79052	79052	1	A	2	A/G	57.1/42.9	8/6	14	A	57.1428571	8	G	42.8571429	6		
chr1	CLCBio	SNP	79059	79059	1	G	2	G/A	80.0/20.0	8/2	10	G	80	8	A	20	2		
chr1	CLCBio	SNP	79061	79061	1	A	2	A/G	75.0/25.0	3/1	4	A	75	3	G	25	1		
chr1	CLCBio	SNP	79163	79163	1	C	2	T/C	69.4/30.6	50/22	72	T	69.4444444	50	C	30.5555556	22		
chr1	CLCBio	SNP	79377	79377	1	T	2	T/C	70.0/30.0	7/3	10	T	70	7	C	30	3		
chr1	CLCBio	SNP	79395	79395	1	A	2	A/G	55.6/44.4	5/4	9	A	55.5555556	5	G	44.4444444	4		
chr1	CLCBio	SNP	79923	79923	1	A	2	A/G	90.2/9.8	46/5	51	A	90.1960784	46	G	9.80392157	5		
chr1	CLCBio	SNP	79932	79932	1	C	2	C/T	89.8/8.5	53/5	59	C	89.8305085	53	T	8.47457627	5		
chr1	CLCBio	SNP	79933	79933	1	G	2	G/A	86.8/9.4	46/5	53	G	86.7924528	46	A	9.43396226	5		
chr1	CLCBio	SNP	79937	79937	1	A	2	A/G	58.1/40.3	36/25	62	A	58.0645161	36	G	40.3225806	25		
chr1	CLCBio	SNP	79961	79961	1	A	2	A/G	85.4/12.2	35/5	41	A	85.3658537	35	G	12.195122	5		
chr1	CLCBio	SNP	80043	80043	1	A	2	A/G	74.1/25.9	20/7	27	A	74.0740741	20	G	25.9259259	7		
chr1	CLCBio	SNP	80073	80073	1	A	1	G	91.9	34	37	G	91.8918919	34					
chr1	CLCBio	SNP	80074	80074	1	C	2	C/T	80.6/16.7	29/6	36	C	80.5555556	29	T	16.6666667	6		
chr1	CLCBio	SNP	80221	80221	1	G	2	G/C	86.4/13.6	191/30	221	G	86.4253394	191	C	13.5746606	30		
chr1	CLCBio	SNP	81219	81219	1	T	2	T/C	97.0/3.0	292/9	301	T	97.0099668	292	C	2.99003322	9		
chr1	CLCBio	SNP	81735	81735	1	T	2	T/C	97.9/2.1	230/5	235	T	97.8723404	230	C	2.12765957	5		
chr1	CLCBio	SNP	81961	81961	1	A	2	A/G	67.2/32.8	86/42	128	A	67.1875	86	G	32.8125	42		
chr1	HapMap rs1799944	SNP	81961	81961		A		G	het										
chr1	CLCBio	SNP	81983	81983	1	G	2	G/A	98.5/1.5	326/5	331	G	98.489426	326	A	1.51057402	5		
chr1	CLCBio	SNP	82254	82254	1	T	2	T/C	92.3/7.7	84/7	91	T	92.3076923	84	C	7.69230769	7		
chr1	CLCBio	SNP	82401	82401	1	G	2	G/T	98.5/1.5	450/7	457	G	98.4682713	450	T	1.53172867	7		
chr1	CLCBio	SNP	83513	83513	1	G	2	G/A	95.8/4.2	203/9	212	G	95.754717	203	A	4.24528302	9		
chr1	CLCBio	SNP	83553	83553	1	A	1	G	100.0	92	92	G	100	92					
chr1	HapMap rs206075	SNP	83553	83553		A		G	hom										
chr1	CLCBio	SNP	83725	83725	1	G	2	G/T	99.1/0.9	689/6	695	G	99.1366906	689	T	0.86330935	6		
chr1	HapMap rs28897731	SNP	83918	83918		T		A	hom										
chr1	CLCBio	SNP	84408	84408	1	A	2	G/A	51.2/48.8	173/165	338	G	51.183432	173	A	48.816568	165		
chr1	CLCBio	SNP	84956	84956	1	C	2	C/A	98.6/1.4	359/5	364	C	98.6263736	359	A	1.37362637	5		
chr1	CLCBio	SNP	85503	85503	1	G	1	C	100.0	55	55	C	100	55					
chr1	CLCBio	Complex SNP	86267	86267	1	C	3	C/T/A	95.8/3.2/1.1	542/18/6	566	C	95.7597173	542	T	3.18021201	18		
chr1	CLCBio	SNP	86268	86268	1	G	2	G/A	96.1/3.9	584/24	608	G	96.0526316	584	A	3.94736842	24		
chr1	CLCBio	Complex SNP	86272	86272	1	C	3	C/T/A	92.4/6.4/1.0	475/33/5	514	C	92.4124514	475	T	6.42023346	33		
chr1	CLCBio	SNP	86273	86273	1	G	2	G/A	95.4/4.1	558/24	585	G	95.3846154	558	A	4.1025641	24		
chr1	CLCBio	SNP	86276	86276	1	C	2	C/T	92.6/6.9	549/41	593	C	92.5801012	549	T	6.91399663	41		
chr1	CLCBio	SNP	86277	86277	1	G	2	G/A	95.6/4.4	567/26	593	G	95.6155143	567	A	4.38448567	26		
chr1	CLCBio	SNP	86283	86283	1	C	2	C/T	97.0/2.7	755/21	778	C	97.0437018	755	T	2.69922879	21		
chr1	CLCBio	SNP	86284	86284	1	G	2	G/A	96.8/2.9	867/26	896	G	96.7633929	867	A	2.90178571	26		
chr1	CLCBio	Complex SNP	86297	86297	1	C	3	C/T/G	92.7/5.7/1.2	748/46/10	807	C	92.6889715	748	T	5.70012392	46		
chr1	CLCBio	SNP	86298	86298	1	G	2	G/A	98.5/1.4	865/12	878	G	98.5193622	865	A	1.3667426	12		
chr1	CLCBio	SNP	86308	86308	1	C	2	T/C	63.3/36.2	243/139	384	T	63.28125	243	C	36.1979167	139		
chr1	CLCBio	SNP	86309	86309	1	G	2	G/A	97.0/2.6	480/13	495	G	96.969697	480	A	2.62626263	13		
chr1	CLCBio	SNP	86315	86315	1	A	2	C/A	66.7/31.8	174/83	261	C	66.6666667	174	A	31.8007663	83		
chr1	CLCBio	SNP	89302	89302	1	T	1	C	100.0	13	13	C	100	13					
chr1	CLCBio	SNP	90380	90380	1	G	2	G/A	75.0/25.0	3/1	4	G	75	3	A	25	1		
chr1	CLCBio	SNP	90383	90383	1	G	2	G/A	75.0/25.0	3/1	4	G	75	3	A	25	1		
chr1	CLCBio	SNP	90482	90482	1	G	2	G/A	71.4/28.6	5/2	7	G	71.4285714	5	A	28.5714286	2		
chr1	CLCBio	SNP	90936	90936	1	T	2	G/T	75.6/24.4	31/10	41	G	75.6097561	31	T	24.3902439	10		
chr1	HapMap rs11571699	SNP	90936	90936		T		G	het										
chr1	CLCBio	SNP	90943	90943	1	C	2	C/T	86.9/13.1	53/8	61	C	86.8852459	53	T	13.1147541	8		
chr1	CLCBio	SNP	91136	91136	1	C	2	C/A	97.1/2.9	166/5	171	C	97.0760234	166	A	2.92397661	5		
chr1	CLCBio	SNP	91152	91152	1	C	2	C/A	97.4/2.6	189/5	194	C	97.4226804	189	A	2.57731959	5		
chr1	CLCBio	SNP	91226	91226	1	A	2	A/G	59.4/39.4	104/69	175	A	59.4285714	104	G	39.4285714	69		
chr1	CLCBio	SNP	91690	91690	1	C	2	T/C	60.0/40.0	33/22	55	T	60	33	C	40	22		
chr1	HapMap rs9943876	SNP	91690	91690		C		T	het										
chr1	CLCBio	SNP	91959	91959	1	C	2	C/A	92.7/7.3	203/16	219	C	92.6940639	203	A	7.30593607	16		
chr1	CLCBio	SNP	92197	92197	1	G	2	G/C	59.1/40.9	136/94	230	G	59.1304348	136	C	40.8695652	94		
chr1	CLCBio	SNP	92203	92203	1	T	2	T/C	96.9/3.1	247/8	255	T	96.8627451	247	C	3.1372549	8		
chr1	CLCBio	SNP	92499	92499	1	A	2	A/G	67.5/32.5	199/96	295	A	67.4576271	199	G	32.5423729	96		
chr1	CLCBio	SNP	92577	92577	1	T	1	C	98.9	86	87	C	98.8505747	86					
chr1	CLCBio	SNP	92914	92914	1	C	2	C/T	72.2/27.8	13/5	18	C	72.2222222	13	T	27.7777778	5		
chr1	CLCBio	SNP	92941	92941	1	A	2	A/C	68.8/25.0	11/4	16	A	68.75	11	C	25	4		
chr1	CLCBio	SNP	92959	92959	1	C	2	C/T	60.0/40.0	3/2	5	C	60	3	T	40	2		

chr1	CLCBio	SNP	93035	93035	1	C	2	C/A	75.0/25.0	3/1	4	C	75	3	A	25	1		
chr1	CLCBio	SNP	93074	93074	1	T	2	T/C	75.0/25.0	6/2	8	T	75	6	C	25	2		
chr1	CLCBio	SNP	93075	93075	1	G	2	G/A	57.1/42.9	4/3	7	G	57.1428571	4	A	42.8571429	3		
chr1	CLCBio	SNP	93260	93260	1	T	2	T/G	50.0/33.3	3/2	6	T	50	3	G	33.3333333	2		
chr1	CLCBio	SNP	93279	93279	1	C	2	C/T	68.4/31.6	13/6	19	C	68.4210526	13	T	31.5789474	6		
chr1	CLCBio	SNP	93386	93386	1	G	2	G/A	80.0/20.0	8/2	10	G	80	8	A	20	2		
chr1	CLCBio	SNP	93387	93387	1	A	2	A/G	63.2/36.8	12/7	19	A	63.1578947	12	G	36.8421053	7		
chr1	CLCBio	SNP	93397	93397	1	C	2	C/A	94.6/3.0	157/5	166	C	94.5783133	157	A	3.01204819	5		
chr1	CLCBio	Complex SNP	93403	93403	1	A	3	A/G/T	84.3/11.3/4.4	172/23/9	204	A	84.3137255	172	G	11.2745098	23		
chr1	CLCBio	SNP	93406	93406	1	C	2	C/T	98.1/1.9	260/5	265	C	98.1132075	260	T	1.88679245	5		
chr1	CLCBio	SNP	93421	93421	1	G	2	G/A	98.4/1.6	310/5	315	G	98.4126984	310	A	1.58730159	5		
chr1	CLCBio	Complex SNP	93430	93430	1	C	3	C/T/A	83.3/14.4/2.3	220/38/6	264	C	83.3333333	220	T	14.3939394	38		
chr1	CLCBio	Complex SNP	93431	93431	1	G	3	G/A/T	90.0/7.5/1.8	252/21/5	280	G	90	252	A	7.5	21		
chr1	CLCBio	SNP	93440	93440	1	T	2	T/C	87.8/11.2	165/21	188	T	87.7659574	165	C	11.1702128	21		
chr1	CLCBio	SNP	93446	93446	1	T	2	T/C	92.9/7.1	118/9	127	T	92.9133858	118	C	7.08661417	9		
chr1	CLCBio	SNP	93625	93625	1	C	2	C/G	99.5/0.5	1434/7	1441	C	99.5142262	1434	G	0.48577377	7		
chr1	CLCBio	SNP	93626	93626	1	T	2	T/C	99.5/0.5	1092/5	1097	T	99.5442115	1092	C	0.45578851	5		
chr1	CLCBio	SNP	93674	93674	1	C	2	C/T	82.7/17.3	1163/243	1406	C	82.7169275	1163	T	17.2830725	243		
chr1	CLCBio	SNP	93838	93838	1	G	2	A/G	62.9/37.1	134/79	213	A	62.9107981	134	G	37.0892019	79		
chr1	CLCBio	SNP	93880	93880	1	G	1	A	99.4	155	156	A	99.3589744	155					
chr1	CLCBio	SNP	94531	94531	1	A	2	A/G	98.3/1.7	297/5	302	A	98.3443709	297	G	1.65562914	5		
chr1	CLCBio	SNP	95228	95228	1	T	2	T/C	61.2/38.8	30/19	49	T	61.2244898	30	C	38.7755102	19		
chr1	CLCBio	SNP	95229	95229	1	A	2	A/G	63.0/35.2	34/19	54	A	62.962963	34	G	35.1851852	19		
chr1	CLCBio	SNP	95231	95231	1	A	2	A/G	92.8/7.2	77/6	83	A	92.7710843	77	G	7.22891566	6		
chr1	CLCBio	SNP	95268	95268	1	C	2	C/T	93.3/6.0	139/9	149	C	93.2885906	139	T	6.04026846	9		
chr1	CLCBio	Complex SNP	95273	95273	1	C	3	C/A/T	90.5/4.8/4.8	114/6/6	126	C	90.4761905	114	A	4.76190476	6		
chr1	CLCBio	SNP	95277	95277	1	T	2	T/C	76.7/23.3	46/14	60	T	76.6666667	46	C	23.3333333	14		
chr1	CLCBio	SNP	95278	95278	1	A	2	A/G	56.9/41.2	29/21	51	A	56.8627451	29	G	41.1764706	21		
chr1	CLCBio	SNP	95431	95431	1	A	2	A/G	89.8/10.2	44/5	49	A	89.7959184	44	G	10.2040816	5		
chr1	CLCBio	SNP	95438	95438	1	C	2	C/T	95.0/4.3	153/7	161	C	95.0310559	153	T	4.34782609	7		
chr1	CLCBio	SNP	95440	95440	1	C	2	C/T	95.1/4.5	212/10	223	C	95.0672646	212	T	4.48430493	10		
chr1	CLCBio	SNP	95456	95456	1	G	2	G/A	95.5/2.7	212/6	222	G	95.4954955	212	A	2.7027027	6		
chr1	CLCBio	SNP	95470	95470	1	T	2	T/C	87.3/10.3	186/22	213	T	87.3239437	186	C	10.3286385	22		
chr1	CLCBio	Complex SNP	95475	95475	1	T	3	T/C/A	91.7/3.9/2.8	166/7/5	181	T	91.7127072	166	C	3.86740331	7		
chr1	CLCBio	SNP	95485	95485	1	T	1	A	85.7	12	14	A	85.7142857	12					
chr1	CLCBio	SNP	96167	96167	1	A	1	G	100.0	112	112	G	100	112					
chr1	CLCBio	SNP	96920	96920	1	T	1	C	100.0	138	138	C	100	138					
chr1	HapMap rs9534262	SNP	96920	96920		T		C	hom										
chr1	CLCBio	SNP	97224	97224	1	C	2	C/T	99.4/0.6	1529/10	1539	C	99.3502274	1529	T	0.64977258	10		
chr1	CLCBio	SNP	97239	97239	1	C	2	C/A	99.6/0.4	1598/6	1604	C	99.6259352	1598	A	0.37406484	6		
chr1	CLCBio	SNP	97893	97893	1	C	2	C/A	99.0/1.0	590/6	596	C	98.9932886	590	A	1.00671141	6		
chr1	CLCBio	SNP	98226	98226	1	C	2	C/A	98.5/1.5	529/8	537	C	98.5102421	529	A	1.48975791	8		
chr1	CLCBio	SNP	98249	98249	1	C	2	C/A	98.6/1.4	544/8	552	C	98.5507246	544	A	1.44927536	8		
chr1	CLCBio	SNP	98277	98277	1	A	2	G/A	62.1/37.9	234/143	377	G	62.0689655	234	A	37.9310345	143		
chr1	CLCBio	SNP	99279	99279	1	T	2	T/C	84.2/13.2	32/5	38	T	84.2105263	32	C	13.1578947	5		
chr1	CLCBio	SNP	99299	99299	1	T	2	C/T	66.0/31.9	31/15	47	C	65.9574468	31	T	31.9148936	15		
chr1	CLCBio	SNP	99309	99309	1	G	2	A/G	53.8/46.2	7/6	13	A	53.8461538	7	G	46.1538462	6		
chr1	CLCBio	SNP	99882	99882	1	G	2	G/A	96.2/3.8	480/19	499	G	96.1923848	480	A	3.80761523	19		
chr1	CLCBio	SNP	100249	100249	1	A	2	A/T	57.4/42.6	27/20	47	A	57.4468085	27	T	42.5531915	20		
chr1	CLCBio	SNP	100260	100260	1	C	2	C/T	95.3/4.7	181/9	190	C	95.2631579	181	T	4.73684211	9		
chr1	CLCBio	SNP	100261	100261	1	G	2	G/A	91.3/7.1	168/13	184	G	91.3043478	168	A	7.06521739	13		
chr1	CLCBio	SNP	100267	100267	1	T	2	T/C	81.7/17.3	170/36	208	T	81.7307692	170	C	17.3076923	36		
chr1	CLCBio	SNP	100268	100268	1	G	2	G/A	94.2/4.7	180/9	191	G	94.2408377	180	A	4.71204188	9		
chr1	CLCBio	SNP	100271	100271	1	C	2	C/T	92.5/7.5	186/15	201	C	92.5373134	186	T	7.46268657	15		
chr1	CLCBio	SNP	100272	100272	1	G	2	G/A	88.6/10.0	187/21	211	G	88.6255924	187	A	9.95260664	21		
chr1	CLCBio	SNP	100276	100276	1	T	2	T/C	60.6/37.3	117/72	193	T	60.6217617	117	C	37.3056995	72		
chr1	CLCBio	SNP	100277	100277	1	G	2	G/A	96.7/2.3	206/5	213	G	96.713615	206	A	2.34741784	5		
chr1	CLCBio	SNP	100305	100305	1	T	2	A/T	50.0/40.0	5/4	10	A	50	5	T	40	4		
chr1	CLCBio	SNP	100523	100523	1	T	2	T/C	97.7/2.3	208/5	213	T	97.6525822	208	C	2.34741784	5		
chr1	CLCBio	SNP	100842	100842	1	C	2	C/T	95.6/4.4	108/5	113	C	95.5752212	108	T	4.42477876	5		
chr1	CLCBio	SNP	100857	100857	1	T	2	T/C	58.8/41.2	94/66	160	T	58.75	94	C	41.25	66		
chr1	CLCBio	SNP	100882	100882	1	C	2	C/A	97.7/2.3	388/9	397	C	97.7329975	388	A	2.26700252	9		
chr1	CLCBio	SNP	100954	100954	1	G	2	G/T	99.2/0.8	1052/8	1060	G	99.245283	1052	T	0.75471698	8		
chr1	CLCBio	SNP	100959	100959	1	C	2	C/A	98.8/1.2	1134/14	1148	C	98.7804878	1134	A	1.2195122	14		
chr1	CLCBio	SNP	102085	102085	1	C	2	C/A	98.9/1.1	887/10	897	C	98.8851728	887	A	1.1148272	10		

chr1	CLCBio	SNP	102100	102100	1	C	2	C/A	98.7/1.3	818/11	829	C	98.6731001	818	A	1.32689988	11		
chr1	CLCBio	SNP	102371	102371	1	T	2	T/G	61.1/27.8	11/5	18	T	61.1111111	11	G	27.7777778	5		
chr1	CLCBio	SNP	103005	103005	1	T	2	C/T	69.6/30.4	39/17	56	C	69.6428571	39	T	30.3571429	17		
chr1	CLCBio	SNP	103809	103809	1	C	2	G/C	61.8/38.2	55/34	89	G	61.7977528	55	C	38.2022472	34		
chr1	CLCBio	SNP	103874	103874	1	C	2	C/T	97.7/2.3	1108/26	1134	C	97.707231	1108	T	2.29276896	26		
chr1	CLCBio	SNP	103876	103876	1	T	2	T/C	98.6/1.4	1081/15	1096	T	98.6313869	1081	C	1.36861314	15		
chr1	CLCBio	SNP	104100	104100	1	G	2	G/A	92.4/7.6	439/36	475	G	92.4210526	439	A	7.57894737	36		
chr1	CLCBio	SNP	104135	104135	1	A	2	A/G	74.9/25.1	824/276	1100	A	74.9090909	824	G	25.0909091	276		
chr1	CLCBio	SNP	104149	104149	1	A	2	A/C	95.7/4.3	331/15	346	A	95.6647399	331	C	4.33526012	15		
chr1	CLCBio	SNP	104153	104153	1	C	2	C/T	98.6/1.4	827/12	839	C	98.5697259	827	T	1.43027414	12		
chr1	CLCBio	SNP	104509	104509	1	A	2	A/G	75.0/25.0	12/4	16	A	75	12	G	25	4		
chr1	CLCBio	SNP	104632	104632	1	A	2	G/A	80.0/20.0	12/3	15	G	80	12	A	20	3		
chr1	CLCBio	SNP	106199	106199	1	C	2	C/T	94.8/5.2	92/5	97	C	94.8453608	92	T	5.15463918	5		
chr1	CLCBio	SNP	106324	106324	1	T	2	C/T	52.0/48.0	26/24	50	C	52	26	T	48	24		
chr1	HapMap rs4942486	SNP	106324	106324		T		C	het										
chr1	CLCBio	SNP	106540	106540	1	G	2	G/A	72.7/27.3	242/91	333	G	72.6726727	242	A	27.3273273	91		
chr1	CLCBio	SNP	107357	107357	1	C	2	T/C	63.0/37.0	51/30	81	T	62.962963	51	C	37.037037	30		
chr1	CLCBio	SNP	108127	108127	1	C	2	C/A	99.2/0.8	1156/9	1165	C	99.2274678	1156	A	0.77253219	9		
chr1	CLCBio	SNP	108140	108140	1	G	2	G/A	99.6/0.4	1275/5	1280	G	99.609375	1275	A	0.390625	5		
chr1	CLCBio	SNP	108221	108221	1	A	2	A/G	99.1/0.9	1068/10	1078	A	99.0723562	1068	G	0.92764378	10		
chr1	CLCBio	SNP	108523	108523	1	A	2	A/G	98.9/1.1	977/11	988	A	98.8866397	977	G	1.11336032	11		
chr1	CLCBio	SNP	108543	108543	1	G	2	G/T	99.2/0.8	972/8	980	G	99.1836735	972	T	0.81632653	8		
chr1	CLCBio	SNP	108545	108545	1	G	2	G/A	95.3/4.7	863/43	906	G	95.2538631	863	A	4.74613687	43		
chr1	CLCBio	SNP	108566	108566	1	C	2	C/T	99.0/1.0	781/8	789	C	98.9860583	781	T	1.0139417	8		
chr1	CLCBio	SNP	108575	108575	1	A	2	A/G	94.2/5.8	596/37	633	A	94.1548183	596	G	5.84518167	37		
chr1	CLCBio	SNP	108598	108598	1	A	2	A/G	96.9/3.1	347/11	358	A	96.9273743	347	G	3.0726257	11		
chr1	CLCBio	SNP	108637	108637	1	C	2	C/T	54.7/45.3	238/197	435	C	54.7126437	238	T	45.2873563	197		
chr1	CLCBio	SNP	108786	108786	1	C	2	C/A	98.8/1.2	592/7	599	C	98.8313856	592	A	1.16861436	7		
chr1	CLCBio	SNP	108820	108820	1	G	2	G/A	98.2/1.8	443/8	451	G	98.2261641	443	A	1.77383592	8		
chr1	CLCBio	SNP	109189	109189	1	C	2	C/T	99.1/0.9	554/5	559	C	99.1055456	554	T	0.89445438	5		
chr1	CLCBio	SNP	109199	109199	1	G	2	G/C	98.9/1.1	560/6	566	G	98.9399293	560	C	1.06007067	6		
chr1	CLCBio	SNP	109232	109232	1	C	2	C/A	98.8/1.2	600/7	607	C	98.8467875	600	A	1.15321252	7		
chr1	CLCBio	SNP	109277	109277	1	A	2	A/C	81.7/18.3	264/59	323	A	81.7337461	264	C	18.2662539	59		
chr1	CLCBio	SNP	109801	109801	1	T	1	G	100.0	6	6	G	100	6					
chr1	CLCBio	SNP	110937	110937	1	C	2	C/A	98.0/2.0	291/6	297	C	97.979798	291	A	2.02020202	6		
chr1	CLCBio	SNP	111055	111055	1	G	2	G/T	98.3/1.7	585/10	595	G	98.3193277	585	T	1.68067227	10		
chr1	CLCBio	SNP	112755	112755	1	T	1	C	100.0	17	17	C	100	17					
chr1	CLCBio	SNP	113664	113664	1	T	2	T/C	92.9/7.1	65/5	70	T	92.8571429	65	C	7.14285714	5		
chr1	CLCBio	SNP	113695	113695	1	A	2	A/G	63.0/33.3	17/9	27	A	62.962963	17	G	33.3333333	9		
chr1	CLCBio	SNP	113702	113702	1	A	2	A/G	75.9/24.1	22/7	29	A	75.862069	22	G	24.137931	7		
chr1	CLCBio	SNP	113805	113805	1	G	1	A	100.0	27	27	A	100	27					
chr1	CLCBio	SNP	113821	113821	1	A	1	G	94.4	34	36	G	94.4444444	34					
chr1	CLCBio	SNP	114462	114462	1	A	2	A/G	94.5/5.5	173/10	183	A	94.5355191	173	G	5.46448087	10		
chr1	CLCBio	SNP	114593	114593	1	G	2	G/T	96.2/3.8	125/5	130	G	96.1538462	125	T	3.84615385	5		
chr1	CLCBio	SNP	115437	115437	1	A	2	A/G	78.3/21.7	47/13	60	A	78.3333333	47	G	21.6666667	13		
chr1	CLCBio	SNP	115510	115510	1	A	2	A/G	94.2/5.8	113/7	120	A	94.1666667	113	G	5.83333333	7		
chr1	CLCBio	SNP	115746	115746	1	G	2	G/C	57.5/42.5	150/111	261	G	57.4712644	150	C	42.5287356	111		
chr1	CLCBio	SNP	116435	116435	1	T	1	C	100.0	11	11	C	100	11					
chr1	CLCBio	SNP	117204	117204	1	G	2	G/A	56.6/43.4	304/233	537	G	56.6108007	304	A	43.3891993	233		
chr1	HapMap rs11571831	SNP	117204	117204		G		A	het										
chr1	CLCBio	SNP	117275	117275	1	G	2	G/T	97.7/2.3	466/11	477	G	97.6939203	466	T	2.30607966	11		
chr1	CLCBio	SNP	118108	118108	1	G	2	G/C	80.0/20.0	4/1	5	G	80	4	C	20	1		
chr1	CLCBio	SNP	118116	118116	1	G	2	G/T	80.0/20.0	4/1	5	G	80	4	T	20	1		
chr1	CLCBio	SNP	118119	118119	1	C	2	C/T	75.0/25.0	3/1	4	C	75	3	T	25	1		
chr1	CLCBio	Complex SNP	118136	118136	1	G	3	G/A/T	50.0/25.0/25.(	2/1/1	4	G	50	2	A	25	1		

This table shows unfiltered output from CLC bio Workbench 3.7.1. (exported in Excel format), into which we have merged our 15 Gold Standard Consensus genotypes (highlighted in yellow and orange) and the 4 erroneous HapMap3 genotypes (highlighted in blue, details see Table S19). The yellow-highlighted cells indicate concordance of the CLC bio SNP calls with our Gold Standard genotypes for our first rule, i.e. that homozygotes are determined strictly by Variant #1 being represented by 80% or more reads. The orange-highlighted cells indicate concordance for our second rule, i.e. that homozygotes are represented by 78% or more reads. SNP overlap is 15/15 (100%) and genotype concordance is 13/15 (86.7%) using the first rule, or 15/15 (100%) using the second rule.

Table S10: NextGENe V2 Annotated SNP-List after whole genome mapping (Yoruban HapMap ID NA18507, sample 4-plex1bc05)

NA18507

chrom	chromstart+1	snp_name	refBase	varbase	muttype
chr13	31787968	rs206119	G	A	hom
chr13	31788026	rs9562605	C	T	het
chr13	31791791	rs9534174	A	G	hom
chr13	31793377	rs206123	g	C	hom
chr13	31809463	rs1799944	A	G	het
chr13	31811055	rs206075	A	G	hom
chr13	*31811420*	*rs28897731*	*T*	*A*	*hom*
chr13	31824654	rs11571699	T	G	het
chr13	31825894	rs9943876	C	T	het
chr13	31834646	rs9534262	T	C	hom
chr13	31851388	rs4942486	T	C	het
chr13	31870380	rs11571831	G	A	het
chr17	38489325	rs8067269	G	A	hom
chr17	*38497326*	*rs28897686*	*C*	*G*	*hom*
chr17	*38498108*	*rs4986848*	*A*	*T*	*hom*
chr17	38498462	rs799917	G	A	hom
chr17	*38509792*	*rs28897673*	*T*	*A*	*hom*
chr17	38510660	rs799912	T	C	hom
chr17	38515089	rs8065872	A	T	hom

Software NextGENe V 2
Project Name s0329_20091008_751to758_2_752_F3_Output.pjt
Date/Time 5/25/2010

						Unique Reads							ALL Reads										
Reference Position	Gene	CDS	Chr	Reference Nucleotide	Score	Coverage	A (#F,#R)	C (#F,#R)	G (#F,#R)	T (#F,#R)	Ins (#F,#R)	Del (#F,#R)	Coverage	A (#F,#R)	C (#F,#R)	G (#F,#R)	T (#F,#R)	Ins (#F,#R)	Del (#F,#R)	SNP db_xref	Genotype	Mutation Call	Amino Acid Change
31788026	BRCA2	0	13	C	30	42	0;0	13;12	0;0	9;8	0;0	0;0	613	0;0	271;75	0;0	204;63	0;0	0;0	rs9562605	CT	c.[-572C>T]+[=]	
31788227	BRCA2	0	13	G	30	15	0;0	0;0	4;4	5;2	0;0	0;0	369	0;0	0;0	180;63	116;10	0;0	0;0	rs9567552	GT	c.[-371G>T]+[=]	
31791704	BRCA2	0	13	A	30	28	10;7	0;0	8;3	0;0	0;0	0;0	134	65;38	0;0	18;13	0;0	0;0	0;0		AG	c.[316+242A>G]+[=]	
31791791	BRCA2	0	13	A	30	21	0;0	0;0	12;9	0;0	0;1	0;0	38	0;0	0;0	21;17	0;0	1;3	0;0	rs9534174	GG	c.[316+329A>G]+[316+329A>G]	
31791823	BRCA2	0	13	A	30	19	3;5	0;0	6;5	0;0	0;0	0;0	249	112;5	0;0	126;6	0;0	0;0	0;0		GA	c.[316+361A>G]+[=]	
31811055	BRCA2	10	13	A	30	21	0;0	0;0	13;8	0;0	0;0	0;0	128	0;0	0;0	43;85	0;0	0;0	0;0	rs206075	GG	c.[4563A>G]+[4563A>G]	1521L>L
31811910	BRCA2	10	13	A	30	26	5;5	0;0	7;9	0;0	0;0	0;0	179	57;4	0;0	105;13	0;0	0;0	0;0	rs34351119	GA	c.[5418A>G]+[=]	1806E>EE
31812196	BRCA2	10	13	G	29	16	3;1	0;0	7;5	0;0	0;0	0;0	168	18;7	0;0	48;95	0;0	0;0	0;0	rs4987048	GA	c.[5704G>A]+[=]	1902D>ND
31813005	BRCA2	10	13	G	30	20	0;0	11;9	0;0	0;0	0;0	0;0	45	0;0	32;13	0;0	0;0	0;0	0;0	rs206076	CC	c.[6513G>C]+[6513G>C]	2171V>V
31818844	BRCA2	0	13	T	29	6	0;0	4;2	0;0	0;0	0;0	0;0	19	0;0	6;13	0;0	0;0	0;0	0;0	rs206080	CC	c.[6938-120T>C]+[6938-120T>C]	
31824654	BRCA2	0	13	T	30	9	0;0	0;0	1;5	0;3	0;0	0;0	42	0;0	0;0	18;18	2;4	0;0	0;0	rs11571699	GT	c.[7008-2344T>G]+[=]	
31824661	BRCA2	0	13	C	16	11	0;0	3;5	0;0	0;3	0;0	0;0	48	0;0	19;19	0;0	3;7	0;0	0;0		CT	c.[7008-2337C>T]+[=]	
31825894	BRCA2	0	13	C	30	19	0;0	6;3	0;0	6;4	0;0	0;0	64	0;0	19;12	0;0	13;20	0;0	0;0	rs9943876	TC	c.[7008-1104C>T]+[=]	
31827007	BRCA2	13	13	G	30	24	0;0	3;5	10;6	0;0	0;0	0;0	236	0;0	92;3	117;24	0;0	0;0	0;0	rs45574331	GC	c.[7017G>C]+[=]	2339K>NK
31827309	BRCA2	13	13	A	30	23	7;6	0;0	5;5	0;0	0;0	0;0	295	70;119	0;0	51;55	0;0	0;0	0;0	rs4986860	AG	c.[7319A>G]+[=]	2440H>HR
31827387	BRCA2	13	13	T	30	19	0;0	8;11	0;0	0;0	0;0	0;0	93	0;0	28;65	0;0	0;0	0;0	0;0	rs169547	CC	c.[7397T>C]+[7397T>C]	2466V>A
31828894	BRCA2	0	13	G	30	22	5;7	0;0	3;7	0;0	0;0	0;0	192	98;13	0;0	55;26	0;0	0;0	0;0	rs11571708	AG	c.[7617+148G>A]+[=]	
31828936	BRCA2	0	13	G	30	24	9;14	0;1	0;0	0;0	0;1	0;0	167	57;107	0;0	2;1	0;0	2;1	0;0	rs206096	AA	c.[7617+190G>A]+[7617+190G>A]	
31829855	BRCA2	0	13	T	20	6	0;0	0;0	5;0	0;1	0;0	0;0	32	0;0	0;0	4;7	3;18	0;0	0;0		GG	c.[7618-24T>G]+[7618-24T>G]	
31831937	BRCA2	0	13	A	30	14	0;0	0;0	7;7	0;0	0;0	0;0	127	0;0	0;0	15;112	0;0	0;0	0;0	rs206098	GG	c.[7805+1871A>G]+[7805+1871A>G]	
31834646	BRCA2	0	13	T	30	20	0;0	10;10	0;0	0;0	0;0	0;0	139	0;0	116;23	0;0	0;0	0;0	0;0	rs9534262	CC	c.[7806-14T>C]+[7806-14T>C]	
31836003	BRCA2	0	13	A	30	5	0;1	0;0	3;1	0;0	0;0	0;0	105	2;3	0;0	3;97	0;0	0;0	0;0	rs11571721	GA	c.[8331+333A>G]+[=]	
31843629	BRCA2	0	13	T	30	12	0;0	4;4	0;0	2;2	0;0	0;0	80	0;0	10;38	0;0	24;8	0;0	0;0	rs11571748	CT	c.[8632+392T>C]+[=]	
31851388	BRCA2	0	13	T	26	13	0;0	3;0	0;0	4;6	0;0	0;0	22	0;0	3;12	0;0	4;3	0;0	0;0	rs4942486	TC	c.[8755-66T>C]+[=]	
31851604	BRCA2	21	13	G	30	22	2;8	0;0	4;8	0;0	0;0	0;0	307	16;69	0;0	42;180	0;0	0;0	0;0		GA	c.[8905G>A]+[=]	2969V>MV
31852421	BRCA2	0	13	C	30	6	0;0	1;1	0;0	3;1	0;0	0;0	74	0;0	4;40	0;0	6;24	0;0	0;0		TC	c.[9256+139C>T]+[=]	
31857199	IFIT1P	0	13	C	30	24	0;0	5;4	0;0	8;7	0;0	0;0	345	0;0	122;38	0;0	143;42	0;0	0;0	rs2238162	TC	c.[9256+4917C>T]+[=]	
31857839	BRCA2	0	13	A	30	23	8;7	3;5	0;0	0;0	0;0	0;0	209	75;5	76;53	0;0	0;0	0;0	0;0	rs2238163	AC	c.[9256+5557A>C]+[=]	
31869425	BRCA2	0	13	T	22	5	0;0	1;3	0;0	0;1	0;0	0;0	12	0;0	5;5	0;0	1;1	0;0	0;0	rs206344	CT	c.[9648+244T>C]+[=]	
31870380	BRCA2	26	13	G	30	38	12;11	0;0	10;5	0;0	0;0	0;0	465	147;50	0;0	215;53	0;0	0;0	0;0	rs11571831	AG	c.[9730G>A]+[=]	3244V>IV
38456214	BRCA1	0	17	G	30	18	5;5	0;0	5;3	0;0	0;0	0;0	212	23;7	0;0	38;144	0;0	0;0	0;0	rs8070179	AG	c.[5332+392G>A]+[=]	
38458368	BRCA1	0	17	T	25	7	3;4	0;0	0;0	0;0	0;0	0;0	12	9;3	0;0	0;0	0;0	0;0	0;0		AA	c.[5278-1708T>A]+[5278-1708T>A]	
38480031	BRCA1	13	17	A	30	42	11;15	0;0	6;10	0;0	0;0	0;0	774	167;250	0;0	196;161	0;0	0;0	0;0		AG	c.[4518A>G]+[=]	1506D>DD
38489325	BRCA1	0	17	G	30	16	9;7	0;0	0;0	0;0	0;0	0;0	196	7;189	0;0	0;0	0;0	0;0	0;0	rs8067269	AA	c.[4186-1207G>A]+[4186-1207G>A]	
38498462	BRCA1	9	17	G	30	22	8;14	0;0	0;0	0;0	0;0	0;0	299	228;71	0;0	0;0	0;0	0;0	0;0	rs799917	AA	c.[2612G>A]+[2612G>A]	871P>L
38500652	BRCA1	0	17	A	30	18	10;8	0;0	0;0	0;0	8;7	0;0	103	97;6	0;0	0;0	0;0	97;6	0;0	rs34725251	insCCT	c.671-250_671-249insCCT;	
38510660	BRCA1	0	17	T	30	21	0;0	7;14	0;0	0;0	0;0	0;0	117	0;0	83;34	0;0	0;0	0;0	0;0	rs799912	CC	c.[213-161T>C]+[213-161T>C]	
38515089	BRCA1	0	17	A	30	25	2;1	0;0	0;0	13;9	0;0	0;0	299	12;8	0;0	0;0	148;131	0;0	0;0	rs8065872	TT	c.[135-3013A>T]+[135-3013A>T]	
38530713	BRCA1	0	17	G	30	33	0;0	4;5	9;15	0;0	0;0	0;0	977	0;0	377;8	537;55	0;0	0;0	0;0	rs799905	GC	c.[-1074G>C]+[=]	

The left table lists 19 non-reference HapMap3 genotypes (of which the 4 erroneous HapMap3 genotypes are italicized, see Table S19). The right table shows the output from NextGENe V2. Concordant genotypes are highlighted in green. NextGENe called 12 of our 15 Gold Standard non-reference genotypes (Table S19). Within this overlap of 12/15 (80%), the genotype concordance is 12/12 (100%).

Table S11: diBayes SNP-calling for Chinese samples versus our Bronze Standard*

HapMap Han Chinese from Beijing NA18561 (C)	Bioscope: Identical mapping and SNP-calling settings (see Table T1 in http://www.ikmb.uni-kiel.de/tngs-backmapping/bioscope_settings.xls)																																			
	With SNP Backmapping												Without SNP Backmapping																							
	TR mapping / no SAET						TR mapping / SAET						TR mapping / no SAET						TR mapping / SAET						WG mapping / no SAET						WG mapping / SAET					
	SNP concord. rate	SNP overlap (Bronze)	Non-Bronze SNPs total	Non-Bronze SNPs annotated	Non-Bronze SNPs pot. novel	ADoC at SNPs	SNP concord. rate	SNP overlap (Bronze)	Non-Bronze SNPs total	Non-Bronze SNPs annotated	Non-Bronze SNPs pot. novel	ADoC at SNPs	SNP concord. rate	SNP overlap (Bronze)	Non-Bronze SNPs total	Non-Bronze SNPs annotated	Non-Bronze SNPs pot. novel	ADoC at SNPs	SNP concord. rate	SNP overlap (Bronze)	Non-Bronze SNPs total	Non-Bronze SNPs annotated	Non-Bronze SNPs pot. novel	ADoC at SNPs	SNP concord. rate	SNP overlap (Bronze)	Non-Bronze SNPs total	Non-Bronze SNPs annotated	Non-Bronze SNPs pot. novel	ADoC at SNPs	SNP concord. rate	SNP overlap (Bronze)	Non-Bronze SNPs total	Non-Bronze SNPs annotated	Non-Bronze SNPs pot. novel	ADoC at SNPs
**No BC - C**	100%	14 / 28	57	24	33	667	100%	12 / 28	42	24	18	887	100%	14 / 28	73	26	47	970	100%	12 / 28	54	26	28	1316	100%	14 / 28	28	27	1	473	100%	14 / 28	28	27	1	473
**4-plex 1**	100%	41%	56	26	30	316	100%	38%	47	23	24	397	100%	41%	73	29	44	462	100%	38%	59	26	33	648	100%	41%	25	24	1	198	100%	38%	23	22	1	194
BC7 - C	100%	12 / 28	61	25	36	283	100%	12 / 28	44	21	23	404	100%	12 / 28	76	27	49	405	100%	12 / 28	56	24	32	641	100%	12 / 28	22	21	1	172	100%	12 / 28	20	19	1	176
BC8 - C	100%	11 / 28	50	27	23	349	100%	9 / 28	49	25	24	390	100%	11 / 28	70	31	39	519	100%	9 / 28	61	27	34	656	100%	11 / 28	28	27	1	224	100%	9 / 28	26	25	1	213
**4-plex 2**	95%	38%	45	24	21	554	94%	30%	36	19	17	787	95%	38%	57	27	30	875	94%	30%	45	21	24	1362	95%	38%	25	24	1	433	94%	30%	20	19	1	485
BC7 - C	100%	11 / 28	45	24	21	529	100%	8 / 28	32	17	15	735	100%	11 / 28	56	27	29	896	100%	8 / 28	42	19	23	1327	100%	11 / 28	23	22	1	415	100%	8 / 28	16	15	1	462
BC8 - C	90%	10 / 28	44	24	20	579	89%	9 / 28	40	21	19	839	90%	10 / 28	58	27	31	854	89%	9 / 28	48	23	25	1397	90%	10 / 28	26	25	1	450	89%	9 / 28	23	22	1	508
**8-plex ex bc4**	87%	14%	31	12	18	335	93%	14%	27	11	16	381	37%	14%	42	14	28	441	93%	14%	35	13	22	575	37%	14%	11	10	0.3	218	93%	14%	11	11	0.3	255
**8-plex**	65%	12%	26	11	15	270	70%	12%	22	9.3	13	305	33%	12%	36	13	23	352	70%	12%	29	11	18	455	33%	12%	9	8.8	0.3	181	70%	12%	9	8.5	0.3	211
BC3 - C	100%	3 / 28	25	12	13	343	100%	4 / 28	20	12	8	368	100%	3 / 28	40	14	26	447	100%	4 / 28	28	14	14	581	100%	3 / 28	11	11	0	267	100%	4 / 28	11	11	0	234
BC4 - C	0%	1 / 28	11	7	4	76	0%	1 / 28	7	3	4	76	0%	1 / 28	16	8	8	86	0%	1 / 28	10	3	7	96	0%	1 / 28	4	4	0	72	0%	1 / 28	1	1	0	80
BC7 - C	100%	4 / 28	32	12	20	356	100%	3 / 28	31	12	19	393	100%	4 / 28	40	14	26	457	100%	3 / 28	39	14	25	557	100%	4 / 28	10	10	0	173	100%	3 / 28	11	11	0	238
BC8 - C	60%	5 / 28	35	13	22	306	80%	5 / 28	30	10	20	383	60%	5 / 28	46	15	31	418	80%	5 / 28	38	12	26	587	60%	5 / 28	11	10	1	213	80%	5 / 28	12	11	1	291
**16-plex ex bc4**	79%	11%	21	9.4	12	181	65%	11%	22	9	13	216	79%	11%	31	11	21	239	65%	11%	29	10	19	326	79%	11%	7	6.7	0.4	160	65%	11%	7	6.4	0.7	189
**16-plex**	69%	9%	20	8.8	11	162	57%	9%	20	8.5	12	195	69%	9%	29	9.9	19	216	57%	9%	28	9.6	18	296	69%	9%	7	6.3	0.4	143	57%	9%	7	6.1	0.6	171
BC3 - C	100%	3 / 28	27	11	16	215	100%	2 / 28	26	12	14	255	100%	3 / 28	34	12	22	289	100%	2 / 28	34	13	21	408	100%	3 / 28	8	8	0	229	100%	2 / 28	9	9	0	267
BC4 - C	0%	0 / 28	7	4	3	29	0%	0 / 28	8	5	3	48	0%	0 / 28	13	5	8	54	0%	0 / 28	15	6	9	84	0%	0 / 28	3	3	0	20	0%	0 / 28	4	4	0	41
BC7 - C	100%	3 / 28	22	9	13	156	100%	3 / 28	20	6	14	164	100%	3 / 28	29	9	20	204	100%	3 / 28	27	8	19	268	100%	3 / 28	7	6	1	130	100%	3 / 28	4	4	0	114
BC8 - C	100%	1 / 28	23	8	15	158	0%	1 / 28	20	7	13	202	100%	1 / 28	34	9	25	239	0%	1 / 28	26	8	18	322	100%	1 / 28	4	4	0	132	0%	1 / 28	4	4	0	135
BC11 - C	100%	2 / 28	16	5	11	152	75%	4 / 28	21	6	15	171	100%	2 / 28	25	6	19	197	75%	4 / 28	30	7	23	239	100%	2 / 28	2	2	0	156	75%	4 / 28	3	3	0	152
BC12 - C	67%	3 / 28	15	9	6	200	67%	3 / 28	23	11	12	266	67%	3 / 28	21	10	11	300	67%	3 / 28	29	12	17	355	67%	3 / 28	8	7	1	134	67%	3 / 28	11	8	3	293
BC19 - C	25%	4 / 28	19	10	9	178	33%	3 / 28	22	10	12	247	25%	4 / 28	32	13	19	191	33%	3 / 28	26	11	15	314	25%	4 / 28	7	7	0	189	33%	3 / 28	7	7	0	226
BC20 - C	60%	5 / 28	28	14	14	207	80%	5 / 28	23	11	12	207	60%	5 / 28	43	15	28	256	80%	5 / 28	34	12	22	376	60%	5 / 28	14	13	1	154	80%	5 / 28	12	10	2	139
**20-plex ex bc4**	22%	2%	21	6.3	15	65	22%	2%	22	6.6	15	78	22%	2%	31	8.8	22	97	22%	2%	32	8.8	24	127	22%	2%	4	4.3	0.1	31	22%	2%	4	4.2	0.1	31
**20-plex**	20%	1%	20	5.9	14	61	20%	2%	20	6.1	14	72	20%	1%	29	8.3	21	90	20%	2%	30	8.3	22	117	20%	1%	4	3.9	0.1	28	20%	1%	4	3.8	0.1	28
BC3 - C	0%	0 / 28	21	9	12	59	0%	0 / 28	22	8	14	88	0%	0 / 28	34	13	21	93	0%	0 / 28	33	11	22	125	0%	0 / 28	7	7	0	29	0%	0 / 28	6	6	0	19
BC4 - C	0%	0 / 28	7	2	5	23	0%	0 / 28	6	2	4	19	0%	0 / 28	12	4	8	29	0%	0 / 28	10	4	6	31	0%	0 / 28	0	0	0	0	0%	0 / 28	0	0	0	0
BC7 - C	0%	0 / 28	17	5	12	76	0%	0 / 28	21	5	16	82	0%	0 / 28	25	7	18	86	0%	0 / 28	33	8	25	110	0%	0 / 28	3	3	0	31	0%	0 / 28	3	3	0	29
BC8 - C	0%	0 / 28	19	3	16	49	0%	0 / 28	19	4	15	70	0%	0 / 28	27	6	21	86	0%	0 / 28	27	5	22	110	0%	0 / 28	1	1	0	17	0%	0 / 28	2	2	0	28
BC11 - C	0%	0 / 28	22	7	15	67	0%	1 / 28	20	6	14	67	0%	0 / 28	34	9	25	85	0%	1 / 28	31	8	23	112	0%	1 / 28	3	3	0	57	0%	1 / 28	3	3	0	57
BC12 - C	100%	1 / 28	23	8	15	66	100%	1 / 28	25	8	17	93	100%	1 / 28	31	9	22	80	100%	1 / 28	38	11	27	132	100%	1 / 28	7	7	0	44	100%	1 / 28	6	6	0	49
BC15 - C	0%	1 / 28	22	8	14	66	0%	1 / 28	21	7	14	64	0%	1 / 28	35	11	24	114	0%	1 / 28	33	9	24	145	0%	1 / 28	5	5	0	23	0%	1 / 28	5	5	0	23
BC16 - C	100%	2 / 28	26	4	22	70	100%	1 / 28	26	6	20	93	100%	2 / 28	38	7	31	112	100%	1 / 28	37	8	29	152	100%	1 / 28	5	4	1	34	100%	1 / 28	5	4	1	34
BC19 - C	0%	0 / 28	16	6	10	61	0%	0 / 28	18	6	12	71	0%	0 / 28	21	8	13	110	0%	0 / 28	26	8	18	113	0%	0 / 28	4	4	0	23	0%	0 / 28	4	4	0	23
BC20 - C	0%	0 / 28	24	7	17	68	0%	1 / 28	23	9	14	79	0%	0 / 28	34	9	25	107	0%	1 / 28	34	11	23	139	0%	0 / 28	5	5	0	22	0%	0 / 28	5	5	0	22

* Bronze Standard: 28 non-reference-base HapMap SNPs [http://www.sanger.ac.uk/humgen/hapmap3/], not validated by further means, see Table S19.

Annotated SNPs are SNPs in dbSNP130.

We separate barcode 4 samples from the others, because the SOLiD on-machine software SETS assigned only ca. 1% of the expected reads to barcode 4.

The object of this table is to provide supporting data for Tables S2 and S3 to identify the best approach(es) from: enhanced (SAET 2.2) reads or raw off-machine reads, whole genome (WG) or target region (TR) mapping, SNP-backmapping or not. This table confirms the Gold and Silver Standard results (Tables S2 and S3): Concordance/overlap within samples of the same pool are evenly distributed, and the results deteriorate as expected for higher plexing. TR mapping of raw reads leads to the highest number of known SNPs, despite lower coverages, and also to the highest number of potential novel SNPs. WG mapping leads to near-zero potential novel SNPs. Backmapping reduces the total number of SNPs by 15-20% and the number of potential novel SNPs by about a third. SNP concordance is near 100% for the non-barcoded control and for the 4-plex samples. SNP overlap is significantly lower than for the Yoruban, which we suspect is due to the significant false positive rate in the HapMap 3 data (see estimate of 12%-14% on the HapMap website). The sequencing error is probably much smaller than the Bioscope diBayes SNP-calling error (see Table S2, footnote ** manual inspection using IGV).

Table S12: In silico novel SNP validation by computing inter-sample intra-plex SNP concordances

HapMap Yoruban from Ibadan NA18507 (Y) and Han Chinese from Beijing NA18561 (C)	TR mapping / no SAET											TR mapping / SAET										
		SNPs confirmed in min 75% of samples of same plex					SNPs confirmed in all samples of same plex						SNPs confirmed in min 75% of samples of same plex					SNPs confirmed in all samples of same plex				
	Total SNPs found	confirmed SNPS	% confirmed SNPs	unknown in dbSNP 130	pot. novel (unknown in dbSNP and WG-NGS)	% pot. novel (unknown in dbSNP and WG-NGS)	all confirmed	% all confirmed	unknown in dbSNP 130	pot. novel (unknown in dbSNP and WG-NGS)	% pot. novel (unknown in dbSNP and WG-NGS)	Total SNPs found	confirmed SNPS	% confirmed SNPs	unknown in dbSNP 130	pot. novel (unknown in dbSNP and WG-NGS)	% pot. novel (unknown in dbSNP and WG-NGS)	all confirmed	% all confirmed	unknown in dbSNP 130	pot. novel (unknown in dbSNP and WG-NGS)	% pot. novel (unknown in dbSNP and WG-NGS)
No BC - Y	75											58										
No BC - C	71											54										
**no BC**																						
**4-plex 1**																						
BC5 - Y	75	54	72%	15	15	20%	54	72%	15	15	20%	64	46	69%	12	11	17%	46	69%	12	11	17%
BC6 - Y	76											69										
BC7 - C	73	51	76%	17	17	25%	51	76%	17	17	25%	56	47	82%	18	18	32%	47	82%	18	18	32%
BC8 - C	61											58										
**4-plex 2**																						
BC7 - C	56	43	78%	14	14	25%	43	78%	14	14	25%	40	32	72%	10	10	22%	32	72%	10	10	22%
BC8 - C	54											49										
BC9 - Y	61	48	74%	11	10	15%	48	74%	11	10	15%	50	33	62%	8	7	13%	33	62%	8	7	13%
BC10 - Y	69											57										
**8-plex**																						
BC1 - Y	50	30	59%	10	10	20%	18	35%	8	8	16%	45	26	54%	9	9	19%	11	23%	3	3	6%
BC2 - Y	46											42										
BC5 - Y	56											55										
BC6 - Y	53											49										
BC3 - C	28	28	83%	15	9	27%	12	36%	6	6	18%	24	21	68%	8	5	16%	9	29%	3	3	10%
BC7 - C	36											34										
BC8 - C	37											35										
**16-plex**																						
BC1 - Y	40	8	25%	4	4	12%	5	15%	2	2	6%	30	8	28%	3	3	10%	3	10%	2	2	7%
BC2 - Y	38											34										
BC5 - Y	36											25										
BC6 - Y	37											36										
BC9 - Y	27											28										
BC10 - Y	30											26										
BC13 - Y	28											28										
BC14 - Y	23											25										
BC3 - C	30	11	45%	6	4	16%	5	20%	2	2	8%	28	8	32%	6	3	12%	3	12%	2	2	8%
BC7 - C	25											23										
BC8 - C	24											21										
BC11 - C	18											25										
BC12 - C	18											26										
BC19 - C	23											25										
BC20 - C	33											28										
**20-plex**																						
BC1 - Y	45	14	43%	8	8	25%	5	16%	4	4	12%	43	14	45%	8	8	25%	5	16%	3	3	10%
BC2 - Y	38											41										
BC5 - Y	31											32										
BC6 - Y	34											30										
BC9 - Y	28											26										
BC10 - Y	27											27										
BC13 - Y	37											31										
BC14 - Y	24											29										
BC17 - Y	29											27										
BC18 - Y	29											28										
BC3 - C	21	11	51%	7	3	14%	3	14%	2	2	9%	22	12	54%	7	4	18%	3	14%	2	2	9%
BC7 - C	17											21										
BC8 - C	19											19										
BC11 - C	22											21										
BC12 - C	24											26										
BC15 - C	23											22										
BC16 - C	28											27										
BC19 - C	16											18										
BC20 - C	24											24										

We did not include barcode 4 samples, because the SOLiD on-machine software SETS assigned only ca. 1% of the expected reads to barcode 4.
Whole genome NGS SNPs for Yoruban NA18507: [ftp://hgdownload.cse.ucsc.edu/goldenPath/hg18/database/pgYh1.txt.gz], [http://solidsoftwaretools.com/gf/project/yoruban/, Yoruban_snp_18x.gff]

To validate potential novel SNPs in silico, we narrowed down the SNP-calls (after SNP-backmapping) for each sample: We considered SNPs to be confirmed if they were shared by several samples within the same plex. From these confirmed SNPs we subtracted the SNPs known in dbSNP130, and from the remaining SNPs we subtracted the SNPs known from whole genome sequencing (Illumina, SOLiD). The remaining SNPs are potential novel SNPs, subject to detailed manual inspection in the target region mapping files and the whole genome mapping files. For Illumina reads and the BWA mapper, we have automated this last manual inspection step by extracting all reads under the SNPs which were detected in the target-region-mapping-run, and then mapping just these reads to the whole genome (read-backmapping) to eliminate mapping-artefacts (our pipeline is available from http://www.ikmb.uni-kiel.de/tngs-backmapping/).

Table S13: Potential novel SNPs manually inspected and selected for Sanger re-sequencing

(a)

Backmapped potential novel SNPs from SAET-enhanced reads mapped to the target region. (Known SNPs from dbSNP130 and from SOLiD+Illumina whole genome NGS are eliminated.)

	#bm_sa_array Y		Pos (0)	Pos (1)	Ref	Var	Mut
1	stabm_bm_sa_752bc05_06	chr17	38481261	38481262	C	T	het
2	stabm_bm_sa_752bc05_06	chr17	38484828	38484829	T	C	het
3	stabm_bm_sa_752bc05_06	chr17	38484837	38484838	T	C	het
4	stabm_bm_sa_752bc05_06	chr17	38484944	38484945	G	A	het
5	stabm_bm_sa_752bc05_06	chr17	38484957	38484958	A	T	het
6	stabm_bm_sa_752bc05_06	chr17	38485031	38485032	A	G	het
7	stabm_bm_sa_752bc05_06	chr17	38485148	38485149	C	T	het
8	stabm_bm_sa_752bc05_06	chr17	38523329	38523330	A	C	het
9	stabm_bm_sa_752bc05_06	chr17	38529391	38529392	A	T	het
10	stabm_bm_sa_752bc05_06	chr17	38530164	38530165	G	A	het
11	stabm_bm_sa_752bc05_06	chr17	38530167	38530168	G	A	het

(b)

Backmapped potential novel SNPs from non-SAET reads mapped to the target region. (Known SNPs from dbSNP130 from and SOLiD+Illumina whole genome NGS are eliminated.)

	#bm_ns_array Y	chrom	Pos (0)	Pos (1)	Ref	Var	Mut
1	stabm_bm_ns_752bc05_06	chr13	31821094	31821095	T	C	het
2	stabm_bm_ns_752bc05_06	chr13	31840436	31840437	T	A	het
3	stabm_bm_ns_752bc05_06	chr17	38484828	38484829	T	C	het
4	stabm_bm_ns_752bc05_06	chr17	38484837	38484838	T	C	het
5	stabm_bm_ns_752bc05_06	chr17	38484944	38484945	G	A	het
6	stabm_bm_ns_752bc05_06	chr17	38484957	38484958	A	T	het
7	stabm_bm_ns_752bc05_06	chr17	38485031	38485032	A	G	het
8	stabm_bm_ns_752bc05_06	chr17	38485130	38485131	A	G	het
9	stabm_bm_ns_752bc05_06	chr17	38485148	38485149	C	T	het
10	stabm_bm_ns_752bc05_06	chr17	38490811	38490812	A	G	het
11	stabm_bm_ns_752bc05_06	chr17	38495206	38495207	T	C	het
12	stabm_bm_ns_752bc05_06	chr17	38495283	38495284	T	G	het
13	stabm_bm_ns_752bc05_06	chr17	38523329	38523330	A	C	het
14	stabm_bm_ns_752bc05_06	chr17	38527578	38527579	A	G	het
15	stabm_bm_ns_752bc05_06	chr17	38529391	38529392	A	T	het

(c)

Possible novel SNPs	Manual Inspection of potential novel SNPs in the IGV Viewer					Unlikely novel SNPs
	**Genomic coords chr17**	**Whole-genome-mapping: Yoruban-non-BC**	**Target-Region coords:**	**Target region mapping: 4-plex1-BC05**	**Target region mapping: Yoruban-non-BC**	
	38481262	8C	22722	945C, 277T, 2A, 2G, 11N	5003C, 2G, 1A, 1T, 8N	X
	38484829	2270T, 1N	25437	553T, 274C	2497T, 587C, 4N	X
X	38484838	2386T, 1G	25446	799C, 667T	2832T, 1499C, 2G, 1N	
X	38484945	4607G, 1C, 1T	25553	827G, 709A, 5T, 2N	5111G, 2711A, 36T, 2C	
X	38484958	4808A,1C,2G,3T,6N	25566	900T, 820A, 21C, 1G, 3N	5105A, 3297T, 31C, 5G, 16N	
X	38485032	2214A,2C	25640	1097G, 558A, 1N	2679A, 2514G, 2C, 1T, 5N	
X	38485149	2546C,1A,1T,3N	25757	614C, 227T, 5G, 1N	4206C, 1058T, 6A, 6G, 6N	
	38523330	244A	57362	316A, 89C, 3N	283A, 32C, 1N	X
	38529392	2402A,1T,3N	61390	516A, 1G, 158T, 3N	2470A, 313T, 5N	X
	38530165	8971G,5A,1T	62163	1599G, 322A, 3N	10197G, 1837A, 1T, 24N	X
	38530168	7958G,1A,1N	62166	1525G, 318A, 4N	9379G, 1612A, 2T, 3N	X

Tables (a) and (b) show the list of potential novel SNPs validated in silico by inter-sample intra-plex concordances (see Table S12). The green highlighted cells in tables (a) and (b) show SNPs which are called both from the SAET-enhanced reads and from the raw off-machine reads of the same 4-plex1 libraries. Table (c) shows manual inspection results in IGV for the Yoruban control without bar codes mapped to the whole genome, for the 4-plex1-BC05 Yoruban mapped to the target region, and for the Yoruban control mapped to the target region: Yellow denotes the whole genome mapped genotypes and the single target region mapped genotype which is concordant; blue denotes the confidently different target region mapped genotypes; grey denotes two roughly concordant target region mapped genotypes. We consider the grey and yellow highlighted target region SNPs unlikely. We also consider SNPs unlikely, if less than 20% of reads call a non-reference base. We thus rule out 6 SNPs, leaving 5 remaining possible novel heterozygous SNPs.

Table S14: Exome Sequencing Run Info for the selected public exome sequencing data and public genome sequencing data

	Illumina HiSeq 2000 paired end sequencing; Whole Exome sequencing for the 1000 Genomes Project		Illumina Genome Analyzer II paired end sequencing; 1000 genomes - CEPH/UTAH Trio
	NA12878 (Daughter)	NA12891 (Father)	NA12892 (Mother)
SRA Run ID	SRR098401	SRR098359	SRR032860
Submitting Centre	Broad Institute, Cambridge, MA, USA	Broad Institute, Cambridge, MA, USA	Broad Institute, Cambridge, MA, USA
Run Date	20-DEC-2010	20-DEC-2010	23-MAY-2008
Platform	ILLUMINA	ILLUMINA	ILLUMINA
Model	Illumina HiSeq 2000	Illumina HiSeq 2000	Illumina Genome Analyzer II
Read Count	114,059,565	118,218,244	19,432,355
Base Count	17Gb	17Gb	1Gb
Library Layout	PAIRED	PAIRED	PAIRED
Library Strategy	WXS	WXS	WGS
Library Source	GENOMIC	GENOMIC	GENOMIC
Library Selection	Hybrid Selection	Hybrid Selection	RANDOM
Library Name	Solexa-51024	Solexa-50884	Solexa-3594
Information Source	http://www.ebi.ac.uk/ena/data/view/SRR098401	http://www.ebi.ac.uk/ena/data/view/SRR098359	http://www.ebi.ac.uk/ena/data/view/SRR032860
Download URL	ftp.1000genomes.ebi.ac.uk/vol1/ftp/data/NA12878/sequence_read/SRR098401_1.filt.f	ftp.1000genomes.ebi.ac.uk/vol1/ftp/data/NA12891/sequence_read/SRR098359_1.filt.f	ftp.1000genomes.ebi.ac.uk/vol1/ftp/data/NA12892/sequence_read/SRR032860_1.filt.fastq.gz
Download URL	ftp.1000genomes.ebi.ac.uk/vol1/ftp/data/NA12878/sequence_read/SRR098401_2.filt.f	ftp.1000genomes.ebi.ac.uk/vol1/ftp/data/NA12891/sequence_read/SRR098359_2.filt.f	ftp.1000genomes.ebi.ac.uk/vol1/ftp/data/NA12892/sequence_read/SRR032860_2.filt.fastq.gz

Insert Size (BWA)	165 bp +- 60bp	166 bp +- 60 bp	77 bp +- 9 bp
HapMap Population	CEU	CEU	CEU

Table S15: Exome comparison of mapping and SNP-calling times for
Proposed Combined Targetregion Mapping + Whole Genome Read-Backmapping (TR) vs. Conventional Whole-Genome-Mapping (WG)

	NA12878 (daughter)						NA12892 (mother)						NA12891 (father)					
	TR-Timing		WG-Timing		TR-Speedup		TR-Timing		WG-Timing		TR-Speedup		TR-Timing		WG-Timing		TR-Speedup	
Step/program	step	total	step	total	step	total	step	total	step	total	step	total	step	total	step	total	step	total
1_bwa_aln	0:50	0:50	2:22	2:22	2.85	2.85	0:05	0:05	0:21	0:21	3.77	3.77	0:52	0:52	2:32	2:32	2.91	2.91
2_bwa_sampe	1:51	2:41	5:08	7:31	2.77	2.79	0:03	0:09	1:01	1:23	18.16	9.16	1:57	2:50	5:40	8:13	2.89	2.90
3_sam2bam	1:48	4:30	2:05	9:36	1.15	2.13	0:07	0:16	0:10	1:33	1.50	5.78	1:51	4:42	2:08	10:22	1.15	2.21
4_rmdup_step1	1:23	5:53	2:25	12:02	1.75	2.04	0:04	0:20	0:11	1:45	2.63	5.12	1:25	6:07	2:27	12:49	1.72	2.09
5_rmdup_step2	0:19	6:12	0:52	12:54	2.70	2.08	0:00	0:21	0:03	1:49	7.52	5.18	0:20	6:28	0:53	13:43	2.56	2.12
6_mpileup_step1	2:36	8:49	8:58	21:53	3.44	2.48	0:01	0:22	0:40	2:29	29.25	6.66	2:46	9:14	8:44	22:27	3.16	2.43
7_mpileup_step2	0:02	8:51	0:05	**21:58**	2.09	2.48	0:02	0:24	0:02	**2:32**	1.25	6.18	0:02	9:17	0:04	**22:32**	1.99	2.43
8_convert_tr_SNPs_to_wg	0:00	8:51					0:00	0:24					0:00	9:17				
9_split_hom_and_het_snps	0:00	8:51					0:00	0:24					0:00	9:17				
10_hetsnp_read_extraction	0:05	8:57					0:00	0:24					0:05	9:22				
11_bwa_aln_hets	0:03	9:00					0:00	0:25					0:02	9:25				
12_bwa_sampe_hets	0:13	9:13					0:07	0:32					0:16	9:42				
13_sam2bam_hets	0:03	9:16					0:00	0:32					0:03	9:45				
14_rmdup_step1	0:02	9:19					0:00	0:32					0:02	9:48				
15_rmdup_step2	0:01	9:20					0:00	0:32					0:01	9:50				
16_pibase_hets	0:24	9:41				2.27	0:00	0:32				4.68	0:24	10:12				2.21

Table S16: Exome comparison of SNP-results for
Proposed Combined Targetregion Mapping + Whole Genome Read-Backmapping (TR) vs. Conventional Whole-Genome-Mapping (WG)

		NA12878 (daughter)		NA12892 (mother)		NA12891 (father)	
		TR	WG	TR	WG	TR	WG
1	Number of SNPs called by SAMtools	71488	805909	4131	280	77055	709807
2	Number of homozygous SNPs	16518	367460	1921	229	17052	329290
3	Number of heterozygous SNPs	54970	438449	2210	51	60003	380517
4	Number of SNPs after read-backmapping and pibase-recal	38602	-	1932	-	38763	-
5	Number of hets after read-backmapping	19368	-	0	-	19068	-
6	Number of homs after read-backmapping	19234	-	1932	-	19695	-
7	Number of SNPs eliminated by read-backmapping	32886	-	2199	-	38292	-
	Percentage of SNPs eliminated by read-backmapping	46%	-	53%	-	50%	-
8	Number of SNPs after masking with exome target BED-file	20745	20183	1445	0	20901	19912
9	SNP-overlap between TR and WG	18545		0		18316	
		89%	92%	0%	0%	88%	92%
10	Concordance between TR and WG	18333		0		18151	
		99%		0%		99%	
11	Number of HapMap* non-reference SNPs in target	10193		10037		10135	
12	Overlap with HapMap chipdata*	9277	9529	0	0	9221	9503
13	Concordant with HapMap chipdata*	9209	9492	0	0	9164	9480
14	HapMap SNPs which were not detected (false negatives)	916	664	10037	10037	914	632
15	False negative rate	9.0%	6.5%	100%	100%	9.0%	6.2%

* hapmap3_r1_b36_fwd.CEU.qc.poly.recode.map/ped, only chr1-chr22

Table S17: Coverage differences between plus and minus strand in the *BRCA1* and *BRCA2* regions

	Barcode	Plus strand	Minus strand	% coverage difference between strands
**4-plex 1**	**6**	173	162	6.5%
**4-plex 2**	**9**	320	295	7.8%
	**10**	403	371	7.9%
**8-plex**	**1**	236	218	7.6%
	**2**	166	160	3.3%
	**5**	176	167	4.7%
	**6**	144	132	8.0%
**16-plex**	**1**	166	159	4.2%
	**2**	90	91	-1.1%
	**5**	105	97	7.5%
	**6**	73	66	9.5%
	**9**	60	56	6.7%
	**10**	82	80	3.6%
	**13**	84	78	7.0%
	**14**	71	67	6.7%
**Average**		156	146	6.4%
**Median**		144	132	6.7%

This table shows strand bias in the coverage. We used Bedtools 2.9.0 with the option genomeCoverageBed. The BAM-files were generated from Bioscope 1.0.1 target-region mapped sam files using samtools 0.1.8. It should be mentioned that Bedtools was unable to process the whole-genome mapped BAM files. The table shows a fairly low median bias of strand coverage towards the plus strand of 6.7%.

Table S18: Potential Energy/CO2-Footprint Reduction resulting from
Proposed Combined Targetregion Mapping + Whole Genome Read-Backmapping (TR) for exome data

	kW
Electrical power requirement for 8-core compute node, AMD Shanghai, 32GB RAM, 1.7TB scratch disk (Volker Rehberg, High-performance computing center, Christian-Albrechts-University of Kiel)	0.35

	No. of exomes
HiSeq 2500: 120Gb in 27h = 7 x 17Gb exomes per run (see Table S15)	7
HiSeq 2500 exomes per day	6.3

	Conventional	TR
Number of required 8-core compute nodes per HiSeq 2500	5.8	2.6
Electrical energy required for compute nodes per HiSeq2500 per year, in kWh	17635	8016
Electrical energy required for air-conditioning (Volker Rehberg), in kWh	10581	4809
Total electrical energy required for compute nodes per annum, in kWh	28215	12825
Energy savings per annum per HiSeq2500, in kWh	0	15390
Energy savings per annum per HiSeq2500, in average 2-person-households (www.verivox.de)	0	5.5
CO2 emission footprint reduction per HiSeq2500, in av. 2-person-households	0	5.5
Compute-electricity cost savings per annum per HiSeq2500 (Discounted price from electricity supplier: contract "24Sieben StromBusiness" for large business customers)	0 €	2,770 €

Table S19: Our Gold*/Silver Standards (Yoruban consensus) and Bronze*** Standard (Chinese, raw HapMap3)**

(a)

Gold Standard Yoruban NA18507 Consensus					
Chromosome	Coordinate (1-based)	SNP Name	Ref base	Variant base	muttype
chr13	31787968	rs206119	G	A	hom
chr13	31788026	rs9562605	C	T	het
chr13	31791791	rs9534174	A	G	hom
chr13	31793377	rs206123	g	C	hom
chr13	31809463	rs1799944	A	G	het
chr13	31811055	rs206075	A	G	hom
chr13	31824654	rs11571699	T	G	het
chr13	31825894	rs9943876	C	T	het
chr13	31834646	rs9534262	T	C	hom
chr13	31851388	rs4942486	T	C	het
chr13	31870380	rs11571831	G	A	het
chr17	38489325	rs8067269	G	A	hom
chr17	38498462	rs799917	G	A	hom

Removed from Gold Standard, because false positive in HapMap3:

[chr13	31811420	rs28897731	T	A	hom]
[chr17	38497326	rs28897686	C	G	hom]
[chr17	38498108	rs4986848	A	T	hom]
[chr17	38509792	rs28897673	T	A	hom]

(b)

Silver Standard Yoruban NA18507 Consensus					
Chromosome	Coordinate (1-based)	SNP Name	Ref base	Variant base	muttype
chr13	31787968	rs206119	G	A	hom
chr13	31788026	rs9562605	C	T	het
chr13	31788227		G	T	het
chr13	31790885		G	A	het
chr13	31791704		A	G	het
chr13	31791791	rs9534174	A	G	hom
chr13	31791823		A	G	het
chr13	31791908		A	G	het
chr13	31793377	rs206123	g	C	hom
chr13	31796543		A	G	het
chr13	31797444		C	T	het
chr13	31798933		T	A	het
chr13	31800777		G	A	het
chr13	31803568		G	T	het
chr13	31807615		G	C	het
chr13	31809463	rs1799944	A	G	het
chr13	31809756		T	C	het
chr13	31810733		C	T	het
chr13	31811055	rs206075	A	G	hom
chr13	31811910		A	G	het
chr13	31812196		G	A	het
chr13	31813005		G	C	hom
chr13	31818844		T	C	hom
chr13	31824654	rs11571699	T	G	het
chr13	31824661		C	T	het
chr13	31824944		A	G	het
chr13	31825894	rs9943876	C	T	het
chr13	31827007		G	C	het
chr13	31827309		A	G	het
chr13	31827387		T	C	hom
chr13	31828730		C	T	het
chr13	31828894		G	A	het
chr13	31828936		G	A	hom
chr13	31831937		A	G	hom
chr13	31834646	rs9534262	T	C	hom
chr13	31836003		A	G	het
chr13	31838345		C	A	het
chr13	31843629		T	C	het
chr13	31848257		A	G	het
chr13	31848771		C	G	het
chr13	31849097		A	G	het
chr13	31851388	rs4942486	T	C	het
chr13	31851604		G	A	het
chr13	31852421		C	T	het
chr13	31857199		C	T	het
chr13	31857839		A	C	het
chr13	31864080		T	C	het
chr13	31866607		A	G	het
chr13	31868385		A	G	het
chr13	31868736		G	C	het
chr13	31869425		T	C	hom
chr13	31869590		A	G	het
chr13	31870380	rs11571831	G	A	het
chr13	31871736		A	T	het
chr17	38456214		G	A	hom
chr17	38458368		T	A	hom
chr17	38473846		C	A	hom
chr17	38480031		A	G	het
chr17	38485870		G	C	hom
chr17	38489325	rs8067269	G	A	hom
chr17	38498462	rs799917	G	A	hom
chr17	38510660	rs799912	T	C	hom
chr17	38511674		A	G	hom
chr17	38512162		T	G	het
chr17	38515089	rs8065872	A	T	hom
chr17	38530713		G	C	hom

(c)

Bronze Standard Chinese NA18561					
Chromosome	Coordinate	SNP Name	Ref	Variant	muttype
chr13	31787363	rs3092989	G	A	het
chr13	31787968	rs206119	G	A	het
chr13	31791791	rs9534174	A	G	het
chr13	31797388	rs11571610	A	C	het
chr13	31798149	rs3783265	T	C	het
chr13	31804480	rs766173	A	C	het
chr13	31804729	rs144848	A	C	het
chr13	31804980	rs1801439	A	G	het
chr13	31805153	rs28897709	A	T	hom
chr13	31808721	rs1801499	T	C	het
chr13	31809436	rs28897717	A	T	hom
chr13	31809463	rs1799944	A	G	het
chr13	31809932	rs1799951	A	T	hom
chr13	31812977	rs11571660	A	T	hom
chr13	31818618	rs206079	G	A	het
chr13	31824654	rs11571699	T	G	het
chr13	31825894	rs9943876	C	T	het
chr13	31828735	rs169548	T	A	hom
chr13	31834646	rs9534262	T	C	het
chr13	31835495	rs28897748	T	A	hom
chr13	31848257	rs9534323	a	G	het
chr13	31851388	rs4942486	T	C	het
chr13	31863764	rs11571809	C	T	het
chr17	38469446	rs28897696	G	C	hom
chr17	38476472	rs28897695	A	T	hom
chr17	38498108	rs4986848	A	T	hom
chr17	38505408	rs28897674	T	A	hom
chr17	38509792	rs28897673	T	A	hom

* Gold Standard consensus: consensus of 19 non-reference-base HapMap SNPs [http://www.sanger.ac.uk/humgen/hapmap3/] with ** Silver Standard Consensus (66 non-reference-base whole genome NGS SNPs [ftp://hgdownload.cse.ucsc.edu/goldenPath/hg18/database/pgYh1.txt.gz], [http://solidsoftwaretools.com/gf/project/yoruban/, Yoruban_snp_18x.gff]). The 4 false positive HapMap3 SNPs were confirmed as false positive by the above NGS resequencing and by our NGS resequencing. The last 3 false positive SNPs were confirmed by our Sanger resequencing. The first false positive SNP was discovered afterwards and not submitted for Sanger resequencing because it was obvious (Table S8) and followed the same pattern (complementary base instead of actual base). In table (b), 'gold' SNPs are highlighted in yellow.

*** Bronze Standard: 28 non-reference-base HapMap SNPs [http://www.sanger.ac.uk/humgen/hapmap3/], not validated by further means.

Table S20: Upper bound estimate for false positive SNPs via inter-sample SNP validation between barcoded samples versus non-barcoded controls

Bioscope: Identical mapping and SNP-calling settings (see Table T1 in http://www.ikmb.uni-kiel.de/tngs-backmapping/bioscope_settings.xls)

HapMap Yoruban from Ibadan NA18507 (Y) and Han Chinese from Beijing NA18561 (C)	With SNP Backmapping											
	TR mapping / no SAET						TR mapping / SAET					
	SNP concord. rate with Control Lib	SNP overlap with Control Lib	Non-Control SNPs total	annotated	rest	% rest	SNP concord. rate with Control Lib	SNP overlap with Control Lib	Non-Control SNPs total	annotated	rest	% rest
No BC - Y	100%	75 / 75					100%	58 / 58				
No BC - C	100%	71 / 71					100%	54 / 54				
**no BC - mean**	**100%**	**100%**					**100%**	**100%**				
**4-plex 1**	**97%**	**70%**	**20**	**7**	**14**	**19%**	**97%**	**74%**	**21**	**7**	**14**	**23%**
BC5 - Y	94%	51 / 75	24	7	17	23%	93%	40 / 58	24	7	17	27%
BC6 - Y	96%	53 / 75	23	7	16	21%	98%	47 / 58	22	9	13	19%
BC7 - C	100%	52 / 71	21	7	14	19%	100%	38 / 54	18	5	13	23%
BC8 - C	98%	48 / 71	13	6	7	11%	98%	40 / 54	18	5	13	22%
**4-plex 2**	**95%**	**65%**	**13**	**5**	**8**	**12%**	**96%**	**65%**	**13**	**5**	**8**	**17%**
BC7 - C	98%	45 / 71	11	4	7	13%	97%	32 / 54	8	2	6	15%
BC8 - C	95%	43 / 71	11	4	7	13%	95%	39 / 54	10	3	7	14%
BC9 - Y	92%	48 / 75	13	6	7	11%	91%	34 / 58	16	7	9	18%
BC10 - Y	96%	54 / 75	15	6	9	13%	100%	40 / 58	17	6	11	19%
**8-plex ex bc4**	**91%**	**42%**	**14**	**5**	**9**	**20%**	**90%**	**44%**	**16**	**5**	**11**	**28%**
**8-plex**	**89%**	**38%**	**13**	**5**	**8**	**18%**	**89%**	**40%**	**14**	**4**	**10**	**24%**
BC1 - Y	87%	38 / 75	12	5	7	14%	86%	28 / 58	17	5	12	27%
BC2 - Y	88%	34 / 75	12	6	6	13%	89%	27 / 58	15	6	9	21%
BC3 - C	100%	21 / 71	7	4	3	11%	100%	14 / 54	10	5	5	21%
BC4 - C	80%	5 / 71	7	6	1	8%	83%	6 / 54	2	2	0	0%
BC5 - Y	83%	36 / 75	20	5	15	27%	79%	33 / 58	22	2	20	36%
BC6 - Y	92%	36 / 75	17	5	12	23%	90%	31 / 58	18	8	10	20%
BC7 - C	100%	23 / 71	13	3	10	28%	95%	21 / 54	13	3	10	29%
BC8 - C	85%	26 / 71	14	5	9	23%	89%	18 / 54	17	3	14	40%
**16-plex ex bc4**	**86%**	**26%**	**11**	**4**	**7.6**	**22%**	**86%**	**30%**	**11**	**4**	**7.6**	**26%**
**16-plex**	**87%**	**24%**	**10**	**4**	**6**	**20%**	**85%**	**28%**	**10**	**4**	**6.6**	**25%**
BC1 - Y	88%	26 / 75	14	5	9	23%	83%	18 / 58	12	3	9	30%
BC2 - Y	91%	23 / 75	15	4	11	29%	95%	19 / 58	15	4	11	32%
BC3 - C	95%	21 / 71	9	4	5	17%	93%	14 / 54	14	5	9	32%
BC4 - C	100%	4 / 71	3	2	1	14%	75%	4 / 54	4	2	2	25%
BC5 - Y	72%	25 / 75	11	3	8	22%	94%	16 / 58	9	5	4	16%
BC6 - Y	87%	23 / 75	14	2	12	32%	85%	20 / 58	16	5	11	31%
BC7 - C	82%	17 / 71	8	4	4	16%	93%	15 / 54	8	2	6	26%
BC8 - C	80%	15 / 71	9	5	4	17%	87%	15 / 54	6	3	3	14%
BC9 - Y	82%	17 / 75	10	4	6	22%	76%	17 / 58	11	4	7	25%
BC10 - Y	82%	17 / 75	13	4	9	30%	79%	14 / 58	12	4	8	31%
BC11 - C	91%	11 / 71	7	3	4	22%	84%	19 / 54	6	1	5	20%
BC12 - C	92%	13 / 71	5	3	2	11%	79%	14 / 54	12	4	8	31%
BC13 - Y	90%	20 / 75	8	3	5	18%	76%	17 / 58	11	3	8	29%
BC14 - Y	85%	13 / 75	10	5	5	22%	89%	18 / 58	7	2	5	20%
BC19 - C	75%	16 / 71	7	4	3	13%	80%	15 / 54	10	6	4	16%
BC20 - C	92%	24 / 71	9	3	6	18%	95%	20 / 54	8	3	5	18%
**20-plex ex bc4**	**86%**	**22%**	**11**	**4**	**7**	**26%**	**87%**	**26%**	**12**	**4**	**8.5**	**31%**
**20-plex**	**87%**	**22%**	**10**	**3**	**7**	**26%**	**86%**	**25%**	**12**	**4**	**8**	**31%**
BC1 - Y	82%	28 / 75	17	4	13	29%	75%	24 / 58	19	6	13	30%
BC2 - Y	88%	25 / 75	13	4	9	24%	81%	21 / 58	20	5	15	37%
BC3 - C	83%	12 / 71	9	6	3	14%	100%	9 / 54	13	4	9	41%
BC4 - C	100%	4 / 71	3	1	2	29%	67%	3 / 54	3	1	2	33%
BC5 - Y	74%	19 / 75	12	5	7	23%	83%	18 / 58	14	5	9	28%
BC6 - Y	78%	18 / 75	16	5	11	32%	71%	14 / 58	16	5	11	37%
BC7 - C	91%	11 / 71	6	3	3	18%	100%	10 / 54	11	3	8	38%
BC8 - C	100%	12 / 71	7	2	5	26%	100%	12 / 54	7	2	5	26%
BC9 - Y	92%	13 / 75	15	6	9	32%	92%	13 / 58	13	7	6	23%
BC10 - Y	88%	16 / 75	11	3	8	30%	87%	15 / 58	12	3	9	33%
BC11 - C	86%	14 / 71	8	3	5	23%	77%	13 / 54	8	3	5	24%
BC12 - C	88%	17 / 71	7	2	5	21%	88%	17 / 54	9	1	8	31%
BC13 - Y	78%	23 / 75	14	4	10	27%	75%	16 / 58	15	6	9	29%
BC14 - Y	79%	19 / 75	5	2	3	13%	78%	18 / 58	11	4	7	24%
BC15 - C	83%	12 / 71	11	4	7	30%	85%	13 / 54	9	2	7	32%
BC16 - C	100%	15 / 71	13	2	11	39%	93%	14 / 54	13	2	11	41%
BC17 - Y	75%	16 / 75	13	4	9	31%	80%	15 / 58	12	2	10	37%
BC18 - Y	94%	16 / 75	13	3	10	34%	94%	16 / 58	12	3	9	32%
BC19 - C	80%	10 / 71	6	3	3	19%	100%	10 / 54	8	4	4	22%
BC20 - C	100%	16 / 71	8	2	6	25%	100%	13 / 54	11	4	7	29%

HapMap Yoruban from Ibadan NA18507 (Y) and Han Chinese from Beijing NA18561 (C)	Without SNP Backmapping											
	TR mapping / no SAET						TR mapping / SAET					
	SNP concord. rate with Control Lib	SNP overlap with Control Lib	Non-Control SNPs total	annotated	rest	% rest	SNP concord. rate with Control Lib	SNP overlap with Control Lib	Non-Control SNPs total	annotated	rest	% rest
No BC - Y	100%	90 / 90					100%	67 / 67				
No BC - C	100%	87 / 87					100%	66 / 66				
**no BC - mean**	**100%**	**100%**					**100%**	**100%**				
**4-plex 1**	**97%**	**69%**	**27**	**8**	**19**	**21%**	**97%**	**72%**	**26**	**8**	**18**	**24%**
BC5 - Y	93%	62 / 90	30	9	21	23%	93%	46 / 67	29	9	20	27%
BC6 - Y	98%	62 / 90	29	8	21	23%	98%	52 / 67	28	11	17	21%
BC7 - C	100%	62 / 87	26	7	19	22%	100%	45 / 66	23	6	17	25%
BC8 - C	98%	60 / 87	21	8	13	16%	98%	48 / 66	22	6	16	23%
**4-plex 2**	**96%**	**63%**	**16**	**6**	**11**	**14%**	**96%**	**62%**	**16**	**6**	**10**	**18%**
BC7 - C	97%	53 / 87	14	5	9	13%	97%	37 / 66	13	3	10	20%
BC8 - C	95%	52 / 87	16	5	11	16%	95%	45 / 66	12	4	8	14%
BC9 - Y	91%	57 / 90	16	7	9	12%	91%	38 / 67	20	9	11	19%
BC10 - Y	100%	62 / 90	19	6	13	16%	100%	45 / 67	20	8	12	18%
**8-plex ex bc4**	**90%**	**43%**	**18**	**6**	**13**	**23%**	**90%**	**43%**	**21**	**6**	**15**	**30%**
**8-plex**	**89%**	**38%**	**17**	**6**	**11**	**21%**	**89%**	**39%**	**19**	**6**	**13**	**26%**
BC1 - Y	86%	47 / 90	17	6	11	17%	86%	32 / 67	23	7	16	29%
BC2 - Y	89%	42 / 90	21	8	13	21%	89%	30 / 67	22	8	14	27%
BC3 - C	100%	29 / 87	14	5	9	21%	100%	19 / 66	13	6	7	22%
BC4 - C	83%	8 / 87	9	7	2	12%	83%	9 / 66	2	2	0	0%
BC5 - Y	79%	43 / 90	21	5	16	25%	79%	37 / 67	26	4	22	35%
BC6 - Y	90%	42 / 90	21	6	15	24%	90%	33 / 67	25	10	15	26%
BC7 - C	95%	29 / 87	15	4	11	25%	95%	26 / 66	16	4	12	29%
BC8 - C	89%	32 / 87	19	6	13	25%	89%	22 / 66	21	4	17	40%
**16-plex ex bc4**	**86%**	**28%**	**15**	**4**	**11**	**25%**	**86%**	**30%**	**15**	**5**	**10**	**28%**
**16-plex**	**85%**	**26%**	**14**	**5**	**9**	**24%**	**85%**	**29%**	**14**	**5**	**10**	**28%**
BC1 - Y	83%	34 / 90	18	5	13	25%	83%	21 / 67	17	4	13	34%
BC2 - Y	95%	27 / 90	17	4	13	30%	95%	22 / 67	18	5	13	33%
BC3 - C	93%	27 / 87	10	4	6	16%	93%	18 / 66	18	5	13	36%
BC4 - C	75%	7 / 87	6	3	3	23%	75%	7 / 66	8	3	5	33%
BC5 - Y	94%	30 / 90	15	5	10	22%	94%	19 / 67	14	6	8	24%
BC6 - Y	85%	30 / 90	20	3	17	34%	85%	23 / 67	18	6	12	29%
BC7 - C	93%	21 / 87	11	4	7	22%	93%	19 / 66	11	3	8	27%
BC8 - C	87%	21 / 87	14	6	8	23%	87%	19 / 66	8	4	4	15%
BC9 - Y	76%	23 / 90	17	5	12	30%	76%	21 / 67	18	5	13	33%
BC10 - Y	79%	21 / 90	18	5	13	33%	79%	17 / 67	14	5	9	29%
BC11 - C	84%	16 / 87	11	4	7	26%	84%	21 / 66	13	2	11	32%
BC12 - C	79%	18 / 87	6	4	2	8%	79%	17 / 66	15	5	10	31%
BC13 - Y	76%	27 / 90	14	4	10	24%	76%	21 / 67	17	5	12	32%
BC14 - Y	89%	19 / 90	14	6	8	24%	89%	21 / 67	10	3	7	23%
BC19 - C	80%	23 / 87	13	6	7	19%	80%	17 / 66	12	7	5	17%
BC20 - C	95%	31 / 87	17	4	13	27%	95%	25 / 66	14	4	10	26%
**20-plex ex bc4**	**87%**	**25%**	**16**	**4.8**	**12**	**29%**	**87%**	**29%**	**19**	**5**	**14**	**36%**
**20-plex**	**86%**	**24%**	**16**	**5**	**11**	**29%**	**86%**	**28%**	**18**	**5**	**13**	**35%**
BC1 - Y	75%	36 / 90	24	6	18	30%	75%	29 / 67	28	8	20	35%
BC2 - Y	81%	32 / 90	22	6	16	30%	81%	26 / 67	30	6	24	43%
BC3 - C	100%	19 / 87	15	8	7	21%	100%	15 / 66	18	5	13	39%
BC4 - C	67%	8 / 87	4	2	2	17%	67%	6 / 66	4	2	2	20%
BC5 - Y	83%	26 / 90	17	7	10	23%	83%	19 / 67	19	7	12	32%
BC6 - Y	71%	22 / 90	23	6	17	38%	71%	18 / 67	24	7	17	40%
BC7 - C	100%	16 / 87	9	4	5	20%	100%	17 / 66	16	4	12	36%
BC8 - C	100%	18 / 87	9	4	5	19%	100%	16 / 66	11	3	8	30%
BC9 - Y	92%	18 / 90	21	7	14	36%	92%	17 / 67	19	8	11	31%
BC10 - Y	87%	19 / 90	18	5	13	35%	87%	18 / 67	20	5	15	39%
BC11 - C	77%	20 / 87	14	4	10	29%	77%	18 / 66	14	4	10	31%
BC12 - C	88%	21 / 87	11	3	8	25%	88%	23 / 66	16	2	14	36%
BC13 - Y	75%	28 / 90	21	4	17	35%	75%	20 / 67	25	8	17	38%
BC14 - Y	78%	26 / 90	16	2	14	33%	78%	22 / 67	20	5	15	36%
BC15 - C	85%	20 / 87	16	5	11	31%	85%	19 / 66	15	3	12	35%
BC16 - C	93%	20 / 87	20	4	16	40%	93%	18 / 66	20	3	17	45%
BC17 - Y	80%	20 / 90	16	5	11	31%	80%	18 / 67	19	4	15	41%
BC18 - Y	94%	19 / 90	22	5	17	41%	94%	19 / 67	16	4	12	34%
BC19 - C	100%	14 / 87	7	4	3	14%	100%	14 / 66	12	5	7	27%
BC20 - C	100%	22 / 87	12	3	9	26%	100%	19 / 66	16	5	11	31%

HapMap Yoruban from Ibadan NA18507 (Y) and Han Chinese from Beijing NA18561 (C)	Without SNP Backmapping											
	WG mapping / no SAET						WG mapping / SAET					
	SNP concord. rate with Control Lib	SNP overlap with Control Lib	Non-Control SNPs total	annotated	rest	% rest	SNP concord. rate with Control Lib	SNP overlap with Control Lib	Non-Control SNPs total	annotated	rest	% rest
No BC - Y	100%	50 / 50					100%	50 / 50				
No BC - C	100%	42 / 42					100%	42 / 42				
**no BC - mean**	**100%**	**100%**					**100%**	**100%**				
**4-plex 1**	**97%**	**76%**	**6**	**5**	**1**	**1%**	**97%**	**74%**	**4**	**4**	**0**	**1%**
BC5 - Y	93%	36 / 50	12	10	2	4%	93%	37 / 50	6	5	1	2%
BC6 - Y	98%	35 / 50	7	7	0	0%	98%	34 / 50	7	7	0	0%
BC7 - C	100%	33 / 42	1	1	0	0%	100%	32 / 42	0	0	0	0%
BC8 - C	98%	36 / 42	3	3	0	0%	98%	33 / 42	2	2	0	0%
**4-plex 2**	**96%**	**77%**	**6**	**6**	**0**	**0%**	**96%**	**65%**	**4**	**3**	**0**	**1%**
BC7 - C	97%	33 / 42	1	1	0	0%	97%	23 / 42	1	1	0	0%
BC8 - C	95%	34 / 42	2	2	0	0%	95%	31 / 42	1	1	0	0%
BC9 - Y	91%	36 / 50	9	9	0	0%	91%	31 / 50	5	5	0	0%
BC10 - Y	100%	38 / 50	13	12	1	2%	100%	35 / 50	7	6	1	2%
**8-plex ex bc4**	**90%**	**40%**	**6**	**5**	**1**	**2%**	**90%**	**40%**	**4**	**3**	**1**	**2%**
**8-plex**	**89%**	**36%**	**5**	**5**	**1**	**2%**	**89%**	**36%**	**4**	**3**	**1**	**2%**
BC1 - Y	86%	24 / 50	8	8	0	0%	86%	22 / 50	6	5	1	4%
BC2 - Y	89%	21 / 50	7	6	1	4%	89%	21 / 50	4	4	0	0%
BC3 - C	100%	11 / 42	3	3	0	0%	100%	13 / 42	2	2	0	0%
BC4 - C	83%	1 / 42	4	4	0	0%	83%	1 / 42	1	1	0	0%
BC5 - Y	79%	26 / 50	9	7	2	6%	79%	27 / 50	3	1	2	7%
BC6 - Y	90%	21 / 50	9	9	0	0%	90%	23 / 50	7	7	0	0%
BC7 - C	95%	13 / 42	1	1	0	0%	95%	12 / 42	2	2	0	0%
BC8 - C	89%	14 / 42	2	1	1	6%	89%	14 / 42	3	2	1	6%
**16-plex ex bc4**	**86%**	**22%**	**3**	**3**	**0**	**1%**	**86%**	**21%**	**2**	**2**	**0**	**1%**
**16-plex**	**85%**	**21%**	**3**	**3**	**0**	**2%**	**85%**	**20%**	**2**	**2**	**0.5**	**3%**
BC1 - Y	83%	17 / 50	4	4	0	0%	83%	15 / 50	2	2	0	0%
BC2 - Y	95%	13 / 50	4	4	0	0%	95%	11 / 50	3	2	1	7%
BC3 - C	93%	9 / 42	2	2	0	0%	93%	9 / 42	2	2	0	0%
BC4 - C	75%	1 / 42	2	2	0	0%	75%	2 / 42	2	2	0	0%
BC5 - Y	94%	14 / 50	5	5	0	0%	94%	9 / 50	4	4	0	0%
BC6 - Y	85%	11 / 50	4	4	0	0%	85%	11 / 50	3	3	0	0%
BC7 - C	93%	7 / 42	3	2	1	10%	93%	7 / 42	0	0	0	0%
BC8 - C	87%	3 / 42	2	2	0	0%	87%	3 / 42	2	2	0	0%
BC9 - Y	76%	11 / 50	1	0	1	8%	76%	11 / 50	3	2	1	7%
BC10 - Y	79%	9 / 50	5	4	1	7%	79%	11 / 50	3	2	1	7%
BC11 - C	84%	3 / 42	1	1	0	0%	84%	7 / 42	0	0	0	0%
BC12 - C	79%	8 / 42	3	2	1	9%	79%	9 / 42	5	2	3	21%
BC13 - Y	76%	11 / 50	3	3	0	0%	76%	11 / 50	2	2	0	0%
BC14 - Y	89%	9 / 50	2	2	0	0%	89%	10 / 50	1	1	0	0%
BC19 - C	80%	9 / 42	2	2	0	0%	80%	8 / 42	2	2	0	0%
BC20 - C	95%	16 / 42	3	2	1	5%	95%	13 / 42	4	2	2	12%
**20-plex ex bc4**	**87%**	**14%**	**3**	**3**	**0**	**1%**	**87%**	**14%**	**3**	**3**	**0**	**1%**
**20-plex**	**86%**	**14%**	**3**	**3**	**0**	**1%**	**86%**	**13%**	**2**	**2**	**0**	**1%**
BC1 - Y	75%	16 / 50	5	5	0	0%	75%	15 / 50	5	5	0	0%
BC2 - Y	81%	15 / 50	5	5	0	0%	81%	13 / 50	4	4	0	0%
BC3 - C	100%	3 / 42	4	4	0	0%	100%	3 / 42	3	3	0	0%
BC4 - C	67%	0 / 42	0	0	0	0%	67%	0 / 42	0	0	0	0%
BC5 - Y	83%	9 / 50	6	6	0	0%	83%	10 / 50	4	4	0	0%
BC6 - Y	71%	10 / 50	4	4	0	0%	71%	9 / 50	5	5	0	0%
BC7 - C	100%	1 / 42	2	2	0	0%	100%	1 / 42	2	2	0	0%
BC8 - C	100%	0 / 42	1	1	0	0%	100%	1 / 42	1	1	0	0%
BC9 - Y	92%	6 / 50	5	5	0	0%	92%	6 / 50	4	4	0	0%
BC10 - Y	87%	6 / 50	2	2	0	0%	87%	6 / 50	2	2	0	0%
BC11 - C	77%	3 / 42	1	1	0	0%	77%	3 / 42	1	1	0	0%
BC12 - C	88%	6 / 42	2	2	0	0%	88%	6 / 42	1	1	0	0%
BC13 - Y	75%	13 / 50	6	5	1	5%	75%	11 / 50	4	4	0	0%
BC14 - Y	78%	10 / 50	3	3	0	0%	78%	10 / 50	2	2	0	0%
BC15 - C	85%	4 / 42	2	2	0	0%	85%	4 / 42	2	2	0	0%
BC16 - C	93%	4 / 42	2	1	1	17%	93%	4 / 42	2	1	1	17%
BC17 - Y	80%	7 / 50	3	3	0	0%	80%	9 / 50	2	2	0	0%
BC18 - Y	94%	7 / 50	2	2	0	0%	94%	7 / 50	2	2	0	0%
BC19 - C	100%	2 / 42	2	2	0	0%	100%	2 / 42	2	2	0	0%
BC20 - C	100%	4 / 42	1	1	0	0%	100%	4 / 42	1	1	0	0%

Annotated SNPs are SNPs in dbSNP130.

We separate barcode 4 samples from the others, because the SOLiD on-machine software SETS assigned only ca. 1% of the expected reads to barcode 4.

Annotated SNPs are SNPs in dbSNP130

In this table we analyse the concordance of barcoded samples with the non-barcoded sample (control). Concordance/overlap within samples of the same plex are evenly distributed, and the overlap deteriorates in a similar way for increased plexing as in Tables S2-S3 and S11. For the target region (TR) mapping approach, ca. 12%-36% of SNPs (column "%rest") in a barcoded sample do not occur in the non-barcoded control; this would be the upper bound for false positives. The upper bound is ca. 12%-20% for the 4-plexes and increases as expected for higher plexing. For whole genome (WG) mapping, the upper bound for false positives is mostly lower than 10%, in most cases in fact near 1%. The sequencing error is probably much smaller than the Bioscope diBayes SNP-calling error (see Table S2, footnote ** manual inspection using IGV)

Table S21: Summary of Mapping and SNP-calling with SAET 2.2 + Bioscope 1.0.1/diBayes for all BRCA1/2 samples

HapMap YRI NA18507 (Y) & CHB NA18561 (C)	On-target reads	1X Cov.	5X Cov.	10X Cov.	15X Cov.	20X Cov.	ADoC	EF	AUC	diBayes genotype concord. rate	diBayes SNP overlap (HapMap)	Non-HapMap diBayes SNPs		
												total	annotated	unknown
No BC - Y	62%	99.6%	99.0%	98.5%	98.0%	97.6%	3119.2	21399	0.989	100%	10 / 15	40	38	2
No BC - C	15%	99.6%	98.7%	97.7%	96.8%	96.0%	1084	5298	0.988	100%	14 / 28	28	27	1
**no BC - mean**	**39%**	**99.6%**	**98.8%**	**98.1%**	**97.4%**	**96.8%**	**2102**	**13348**	**0.989**					
**4-plex 1**	**34%**	**97.7%**	**94.6%**	**91.4%**	**88.8%**	**86.6%**	**407**	**11804**	**0.977**					
BC5 - Y	36%	98.2%	95.9%	93.5%	91.2%	89.3%	515	12470	0.981	90%	10 / 15	33	32	1
BC6 - Y	35%	97.8%	95.1%	92.2%	89.7%	87.5%	399	12121	0.978	100%	11 / 15	30	29	1
BC7 - C	33%	97.5%	93.5%	90.0%	87.1%	84.8%	345	11240	0.975	100%	12 / 28	20	19	1
BC8 - C	33%	97.4%	93.6%	89.9%	87.1%	84.9%	369	11383	0.975	100%	9 / 28	26	25	1
**4-plex 2**	**74%**	**97.2%**	**94.7%**	**92.4%**	**90.6%**	**89.2%**	**856**	**25409**	**0.975**					
BC7 - C	77%	97.5%	95.4%	92.9%	91.1%	89.5%	830	26399	0.977	100%	8 / 28	16	15	1
BC8 - C	72%	97.5%	95.1%	92.8%	91.1%	89.9%	865	24892	0.976	89%	9 / 28	23	22	1
BC9 - Y	71%	97.0%	93.9%	91.4%	89.3%	87.9%	765	24566	0.974	63%	8 / 15	28	27	1
BC10 - Y	75%	96.8%	94.5%	92.7%	91.0%	89.5%	965	25781	0.975	100%	10 / 15	32	30	2
**8-plex**	**56%**	**85.8%**	**79.0%**	**74.8%**	**71.6%**	**69.0%**	**331**	**19375**	**0.911**					
BC1 - Y	62%	92.3%	88.6%	85.8%	83.4%	81.4%	561	21424	0.945	56%	9 / 15	19	18	1
BC2 - Y	63%	90.6%	86.4%	82.7%	79.8%	77.1%	403	21536	0.935	86%	7 / 15	18	18	0
BC3 - C	53%	89.1%	83.2%	78.9%	75.4%	72.8%	333	18311	0.927	100%	4 / 28	11	11	0
BC4 - C	*46%*	*57.1%*	*37.9%*	*32.3%*	*28.0%*	*25.4%*	*25*	*15957*	*0.768*	*0%*	*1 / 28*	*1*	*1*	*0*
BC5 - Y	59%	90.8%	86.6%	82.9%	80.2%	77.7%	421	20315	0.937	57%	7 / 15	23	21	2
BC6 - Y	57%	90.2%	85.5%	80.7%	77.8%	75.1%	339	19595	0.933	78%	9 / 15	21	20	1
BC7 - C	55%	88.4%	82.5%	77.9%	74.4%	71.2%	273	19024	0.923	100%	3 / 28	11	11	0
BC8 - C	55%	87.8%	81.4%	77.4%	74.1%	70.9%	290	18836	0.920	80%	5 / 28	12	11	1
**16-plex**	**52%**	**83.3%**	**73.3%**	**67.2%**	**62.7%**	**59.2%**	**187**	**17871**	**0.897**					
BC1 - Y	55%	92.1%	85.5%	80.9%	77.1%	74.2%	401	19086	0.942	60%	5 / 15	12	12	0
BC2 - Y	53%	85.9%	78.2%	72.8%	68.6%	65.0%	225	18335	0.909	100%	4 / 15	10	9	1
BC3 - C	48%	85.9%	75.5%	68.9%	64.2%	60.4%	184	16425	0.909	100%	2 / 28	9	9	0
BC4 - C	*41%*	*49.7%*	*28.5%*	*21.8%*	*18.2%*	*15.8%*	*14*	*14199*	*0.733*	*0%*	*0 / 28*	*4*	*4*	*0*
BC5 - Y	54%	87.6%	78.5%	72.4%	67.7%	64.5%	249	18661	0.919	75%	4 / 15	9	9	0
BC6 - Y	50%	84.9%	75.7%	69.4%	64.8%	61.2%	171	17358	0.904	100%	6 / 15	8	8	0
BC7 - C	49%	83.1%	71.8%	65.3%	60.8%	56.8%	154	16750	0.894	100%	3 / 28	4	4	0
BC8 - C	50%	82.9%	71.2%	64.3%	59.6%	56.1%	155	17126	0.893	0%	1 / 28	4	4	0
BC9 - Y	47%	79.7%	68.5%	62.6%	57.5%	53.7%	142	16132	0.877	100%	3 / 15	11	10	1
BC10 - Y	50%	83.3%	73.5%	67.9%	63.5%	60.1%	198	17214	0.895	75%	4 / 15	10	9	1
BC11 - C	55%	84.6%	75.9%	69.4%	64.1%	60.5%	156	18938	0.903	75%	4 / 28	3	3	0
BC12 - C	58%	84.9%	76.8%	71.0%	66.2%	62.7%	179	20076	0.904	67%	3 / 28	11	8	3
BC13 - Y	54%	85.2%	76.9%	70.7%	66.4%	63.3%	200	18729	0.905	75%	4 / 15	9	9	0
BC14 - Y	54%	83.8%	74.7%	68.6%	64.4%	60.6%	171	18465	0.898	100%	5 / 15	6	6	0
BC19 - C	55%	86.4%	77.6%	71.5%	66.8%	63.1%	168	18961	0.913	33%	3 / 28	7	7	0
BC20 - C	57%	92.2%	84.2%	77.8%	73.7%	69.7%	234	19480	0.945	80%	5 / 28	12	10	2
**20-plex**	**8%**	**76.5%**	**57.5%**	**45.2%**	**37.6%**	**32.2%**	**30**	**2916**	**0.856**					
BC1 - Y	8%	87.8%	75.4%	65.1%	57.2%	51.5%	70	2899	0.913	43%	7 / 15	13	12	1
BC2 - Y	10%	81.1%	65.4%	53.5%	45.4%	39.6%	39	3318	0.878	100%	6 / 15	11	11	0
BC3 - C	8%	76.0%	56.3%	44.3%	36.5%	30.7%	26	2679	0.852	0%	0 / 28	6	6	0
BC4 - C	*6%*	*32.6%*	*11.4%*	*6.0%*	*3.9%*	*2.7%*	*2*	*2235*	*0.645*	*0%*	*0 / 28*	*0*	*0*	*0*
BC5 - Y	8%	80.4%	63.0%	49.8%	42.0%	36.2%	35	2877	0.876	80%	5 / 15	9	9	0
BC6 - Y	8%	78.2%	58.0%	44.1%	35.5%	30.1%	26	2772	0.864	100%	5 / 15	9	9	0
BC7 - C	9%	74.8%	52.8%	40.9%	33.6%	28.1%	23	2959	0.847	0%	0 / 28	3	3	0
BC8 - C	8%	72.2%	52.5%	40.5%	32.6%	26.8%	22	2891	0.834	0%	0 / 28	2	2	0
BC9 - Y	7%	73.8%	52.8%	39.4%	31.6%	26.5%	22	2384	0.840	100%	3 / 15	7	7	0
BC10 - Y	7%	75.8%	56.6%	44.2%	36.8%	31.5%	28	2497	0.851	100%	2 / 15	6	6	0
BC11 - C	10%	76.2%	56.0%	42.6%	35.3%	29.6%	23	3461	0.854	0%	1 / 28	3	3	0
BC12 - C	10%	80.3%	61.2%	48.1%	40.3%	34.3%	29	3524	0.876	100%	1 / 28	6	6	0
BC13 - Y	9%	78.7%	58.9%	46.0%	38.3%	32.9%	30	2925	0.867	100%	6 / 15	9	9	0
BC14 - Y	9%	76.6%	58.3%	45.4%	38.0%	32.2%	27	3195	0.856	60%	5 / 15	7	7	0
BC15 - C	8%	80.1%	61.5%	49.5%	41.6%	36.4%	34	2813	0.871	0%	1 / 28	5	5	0
BC16 - C	9%	82.5%	64.8%	52.2%	44.6%	39.0%	40	3023	0.885	100%	1 / 28	5	4	1
BC17 - Y	8%	79.2%	60.5%	47.6%	39.8%	34.3%	32	2902	0.870	67%	3 / 15	8	8	0
BC18 - Y	8%	79.2%	59.1%	45.6%	37.0%	31.5%	28	2829	0.870	100%	5 / 15	4	4	0
BC19 - C	9%	79.9%	59.4%	46.8%	38.8%	32.8%	26	3087	0.873	0%	0 / 28	4	4	0
BC20 - C	9%	84.9%	66.0%	52.8%	43.8%	37.6%	35	3045	0.900	0%	0 / 28	5	5	0

This table summarises mapping and SNP-calling for each sample. The mapping metrics shown in the table are: the number of on-target reads (high-quality 49.0 reads), bases covered with 1, 5, 10, 15 and 20 reads (completeness), average depth of coverage (ADoC, for 49.0 reads), enrichment factor (EF, for 49.0 reads), and area under ROC curve (AUC). We imported the SNP lists generated by the Bioscope diBayes software into an in-house database, within which we computed genotype concordance (for non-reference SNPs only, as the SNP-caller does not call genotypes which are identical to the reference sequence) and SNP overlap with HapMap 3, SNPs known in dbSNP130, and unknown SNPs. The table shows that very high coverage and high completeness helps the SNP-calling software to detect SNPs, but that many SNPs in the sequenced reads are overlooked. We confirmed the presence of overlooked SNPs using IGV (Tables S8 and S13) and conclude that Bioscope diBayes sometimes over-stringently ignores SNPs.

Table S22: Detailed mapping summary for off-machine reads using Bioscope 1.0.1

HapMap YRI NA18507 (Y) & CHB NA18561 (C)	Number of barcodes	Genome Size	HybSelected Region	Reads Obtained (raw reads)*	Filtered Reads for Calculation (uniquely mappable to genome)	49.0 reads uniquely mapped to genome	Mb of coverage	On-Target Reads (unique)	49.0 reads uniquely mapped to target	Mb of target coverage	Percent On-Target Reads	Percent on target 49.0 reads	Percent on target bases	Enrichment Factor	Enrichment Factor (49.0 reads)	Average Depth of Coverage (per barcode)	Average Depth of Coverage (per spot)	1X Consensus Coverage	5X Cons. Cov.	8X Cons. Cov.	10X Cons. Cov.	15X Cons. Cov.	20X Cons. Cov.	30X Cons. Cov.	40X Cons. Cov.	50X Cons. Cov.
4-plex 1	4	3,080,436,051	89,568	28,392,276	16,340,678	5,671,837	744	3,019,562	1,004,861	136	18%	18%	18%	6355.3	6093.1	356.1	1424.5	97.7%	94.4%	92.3%	91.1%	88.4%	86.2%	82.4%	79.1%	76.0%
4-plex 2	4	3,080,436,051	89,568	34,631,217	19,008,476	5,211,856	851	5,989,428	1,671,854	272	32%	32%	32%	10836.7	11032.3	740.3	2961.0	97.2%	94.5%	93.0%	92.1%	90.2%	88.6%	85.9%	83.5%	81.5%
8-plex	8	3,080,436,051	89,568	29,352,928	16,357,189	5,215,004	741	4,767,966	1,503,046	217	29%	29%	29%	10025.0	9912.4	291.0	2328.2	85.6%	78.8%	76.0%	74.4%	71.2%	68.4%	63.7%	59.8%	56.4%
16-plex	16	3,080,436,051	89,568	37,197,621	19,798,860	6,080,436	893	5,317,746	1,615,719	240	27%	27%	27%	9237.3	9138.8	162.1	2593.7	83.0%	72.9%	68.8%	66.6%	62.0%	58.3%	52.5%	48.1%	44.4%
20-plex	20	3,080,436,051	89,568	33,875,493	20,753,516	6,699,709	946	1,392,083	310,138	59	7%	5%	6%	2306.9	1592.1	26.3	525.4	76.2%	56.9%	48.6%	44.4%	36.4%	30.7%	23.0%	17.9%	14.4%
No BC - Y	-	3,080,436,051	89,568	33,216,839	19,465,742	6,050,455	880	5,366,668	1,735,974	247	28%	29%	28%	9481.9	9867.7		2726.5	99.6%	98.9%	98.6%	98.4%	97.9%	97.5%	96.6%	95.9%	95.1%
No BC - C	-	3,080,436,051	89,568	37,890,578	22,414,101	7,949,682	1,021	2,100,645	663,192	93	9%	8%	9%	3223.2	2869.1		947.9	99.6%	98.7%	98.0%	97.6%	96.7%	95.9%	94.4%	92.8%	91.3%
**no BC - averaged**	**-**	**3,080,436,051**	**89,568**	**35,553,708**	**20,939,921**	**7,000,068**	**950**	**3,733,656**	**1,199,583**	**169**	**18%**	**17%**	**18%**	**6132.2**	**5893.7**		**1786.4**	**99.7%**	**98.8%**	**98.3%**	**98.0%**	**97.3%**	**96.7%**	**95.5%**	**94.3%**	**93.2%**

	Barcode	Genome Size	HybSelected Region	Reads Obtained (raw reads)*	Reads uniquely mapped to genome (incl. 24.2 reads)	49.0 reads uniquely mapped to genome	Mb of coverage	On-Target Reads (unique)	49.0 reads uniquely mapped to target	Mb of target coverage	Percent On-Target Reads	Percent on target 49.0 reads	Percent on target bases	Enrichment Factor	Enrichment Factor (49.0 reads)	Average Depth of Coverage (per barcode)	Average Depth of Coverage (per spot)	1X Consensus Coverage	5X Cons. Cov.	8X Cons. Cov.	10X Cons. Cov.	15X Cons. Cov.	20X Cons. Cov.	30X Cons. Cov.	40X Cons. Cov.	50X Cons. Cov.
**4-plex 1**	BC5 - Y	3,080,436,051	89,568	9,096,031	5,045,339	1,676,393	228	947,117	307,615	43	19%	18%	19%	6456.1	6310.9	447.4		98.1%	95.7%	94.1%	93.2%	90.9%	88.9%	85.4%	82.3%	79.8%
	BC6 - Y	3,080,436,051	89,568	6,611,794	3,912,812	1,379,671	179	740,350	250,181	34	19%	18%	19%	6507.4	6236.5	351.1		97.8%	95.0%	93.1%	91.9%	89.3%	87.1%	83.4%	80.0%	76.9%
	BC7 - C	3,080,436,051	89,568	6,076,429	3,586,261	1,283,820	164	643,895	220,692	29	18%	17%	18%	6174.9	5912.1	303.5		97.5%	93.4%	91.1%	89.7%	86.8%	84.3%	80.4%	77.1%	73.6%
	BC8 - C	3,080,436,051	89,568	6,608,022	3,796,266	1,331,953	173	688,200	226,373	31	18%	17%	18%	6234.7	5845.1	322.3		97.4%	93.4%	91.1%	89.7%	86.7%	84.4%	80.6%	77.0%	73.8%
	**ALL**	**3,080,436,051**	**89,568**	**28,392,276**	**16,340,678**	**5,671,837**	**744**	**3,019,562**	**1,004,861**	**136**	**18%**	**18%**	**18%**	**6355.3**	**6093.1**	**356.1**	**1424.5**	**97.7%**	**94.4%**	**92.3%**	**91.1%**	**88.4%**	**86.2%**	**82.4%**	**79.1%**	**76.0%**
**4-plex 2**	BC7 - C	3,080,436,051	89,568	7,961,278	4,437,986	1,227,092	199	1,452,489	411,465	66	33%	34%	33%	11256.1	11532.3	720.6		97.5%	95.2%	93.6%	92.5%	90.7%	88.9%	86.0%	83.7%	81.8%
	BC8 - C	3,080,436,051	89,568	8,900,065	4,938,100	1,349,598	221	1,517,769	419,009	69	31%	31%	31%	10570.7	10677.7	749.0		97.4%	94.9%	93.3%	92.5%	90.7%	89.3%	86.4%	84.0%	82.0%
	BC9 - Y	3,080,436,051	89,568	8,028,156	4,350,560	1,191,386	195	1,334,403	370,683	61	31%	31%	31%	10548.8	10700.6	658.4		97.0%	93.7%	91.8%	91.0%	89.0%	87.3%	84.6%	82.2%	79.9%
	BC10 - Y	3,080,436,051	89,568	9,741,718	5,281,830	1,443,780	236	1,684,767	470,697	77	32%	33%	32%	10970.2	11212.4	833.0		96.7%	94.3%	93.2%	92.3%	90.5%	89.0%	86.5%	84.2%	82.3%
	**ALL**	**3,080,436,051**	**89,568**	**34,631,217**	**19,008,476**	**5,211,856**	**851**	**5,989,428**	**1,671,854**	**272**	**32%**	**32%**	**32%**	**10836.7**	**11032.3**	**740.3**	**2961.0**	**97.2%**	**94.5%**	**93.0%**	**92.1%**	**90.2%**	**88.6%**	**85.9%**	**83.5%**	**81.5%**
**8-plex**	BC1 - Y	3,080,436,051	89,568	6,004,455	3,327,875	1,035,687	150	1,006,934	311,129	46	30%	30%	30%	10406.2	10331.7	493.6		92.1%	88.5%	86.6%	85.5%	83.0%	81.0%	77.1%	73.8%	70.8%
	BC2 - Y	3,080,436,051	89,568	4,008,564	2,327,933	754,952	106	728,961	234,822	33	31%	31%	31%	10769.4	10697.4	358.9		90.4%	86.2%	83.9%	82.3%	79.5%	76.7%	72.3%	68.6%	65.3%
	BC3 - C	3,080,436,051	89,568	4,114,328	2,237,043	715,287	101	591,613	188,425	27	26%	26%	27%	9095.4	9059.8	289.8		88.8%	82.9%	80.2%	78.5%	74.9%	72.3%	67.6%	63.2%	59.7%
	BC4 - C	3,080,436,051	89,568	290,433	162,453	55,667	7	46,459	15,151	2	29%	27%	28%	9835.6	9360.6	22.2		57.1%	37.5%	33.6%	31.3%	27.0%	24.2%	19.7%	16.5%	13.2%
	BC5 - Y	3,080,436,051	89,568	4,880,335	2,616,438	795,955	118	754,595	228,204	34	29%	29%	29%	9918.9	9860.4	366.4		90.7%	86.4%	84.0%	82.6%	79.9%	77.2%	72.5%	68.7%	65.3%
	BC6 - Y	3,080,436,051	89,568	3,626,078	2,109,171	695,238	96	617,497	201,119	28	29%	29%	29%	10068.9	9949.0	301.6		90.1%	85.1%	82.2%	80.4%	77.4%	74.7%	69.8%	65.5%	62.1%
	BC7 - C	3,080,436,051	89,568	3,052,954	1,727,544	568,319	79	496,883	159,676	23	29%	28%	29%	9892.0	9662.9	241.3		88.2%	82.2%	79.1%	77.5%	73.9%	70.9%	65.6%	60.8%	57.2%
	BC8 - C	3,080,436,051	89,568	3,375,781	1,848,732	593,899	84	525,024	164,520	24	28%	28%	28%	9767.1	9527.2	254.5		87.6%	81.2%	78.5%	77.1%	73.7%	70.4%	65.2%	61.1%	57.5%
	**ALL**	**3,080,436,051**	**89,568**	**29,352,928**	**16,357,189**	**5,215,004**	**741**	**4,767,966**	**1,503,046**	**217**	**29%**	**29%**	**29%**	**10025.0**	**9912.4**	**291.0**	**2328.2**	**85.6%**	**78.8%**	**76.0%**	**74.4%**	**71.2%**	**68.4%**	**63.7%**	**59.8%**	**56.4%**
**16-plex**	BC1 - Y	3,080,436,051	89,568	4,899,027	2,591,032	776,023	117	704,735	210,067	32	27%	27%	27%	9354.3	9309.8	345.1		91.7%	85.3%	82.3%	80.5%	76.8%	73.6%	69.0%	65.2%	61.9%
	BC2 - Y	3,080,436,051	89,568	2,676,886	1,469,076	452,853	66	319,781	120,062	14	22%	27%	22%	7486.3	9118.2	194.9		85.7%	77.9%	74.6%	72.3%	68.0%	64.1%	58.6%	54.0%	50.5%
	BC3 - C	3,080,436,051	89,568	2,668,379	1,333,918	398,232	60	414,431	94,312	17	31%	24%	29%	10685.2	8145.0	155.4		85.6%	75.2%	70.9%	68.3%	63.3%	59.2%	53.6%	48.9%	45.2%
	BC4 - C	3,080,436,051	89,568	192,251	101,453	33,820	5	26,309	8,748	1	26%	26%	26%	8918.6	8896.0	12.6		49.5%	27.9%	22.7%	20.7%	17.1%	14.8%	11.4%	8.8%	6.8%
	BC5 - Y	3,080,436,051	89,568	3,195,035	1,639,682	483,018	74	437,081	129,349	20	27%	27%	27%	9167.7	9210.0	212.8		87.5%	78.2%	74.0%	71.9%	67.3%	63.9%	58.4%	54.5%	50.5%
	BC6 - Y	3,080,436,051	89,568	2,137,151	1,169,254	368,531	53	304,857	95,360	14	26%	26%	26%	8967.0	8899.2	148.8		84.6%	75.1%	71.2%	68.8%	64.2%	60.3%	54.0%	49.2%	45.0%
	BC7 - C	3,080,436,051	89,568	2,046,478	1,062,486	331,351	48	273,054	83,424	12	26%	25%	26%	8838.6	8658.9	132.3		82.8%	71.3%	66.8%	64.7%	60.0%	55.8%	49.7%	44.9%	40.9%
	BC8 - C	3,080,436,051	89,568	2,009,518	1,054,989	329,533	48	275,214	84,450	12	26%	26%	26%	8971.8	8813.7	133.7		82.6%	70.7%	66.0%	63.7%	58.8%	55.1%	48.6%	44.1%	40.1%
	BC9 - Y	3,080,436,051	89,568	1,880,236	998,390	315,735	45	252,875	77,805	11	25%	25%	25%	8710.9	8475.1	122.2		79.4%	67.9%	64.1%	62.0%	56.8%	52.7%	47.0%	42.5%	39.0%
	BC10 - Y	3,080,436,051	89,568	2,779,455	1,360,684	407,227	61	347,474	102,790	16	26%	25%	26%	8782.6	8681.1	167.9		82.9%	73.2%	69.6%	67.5%	62.7%	59.1%	53.7%	49.3%	45.3%
	BC11 - C	3,080,436,051	89,568	1,711,792	956,872	307,332	43	276,904	88,016	13	29%	29%	29%	9952.6	9849.5	136.4		84.4%	75.4%	70.9%	68.8%	63.3%	59.3%	52.9%	48.4%	44.4%
	BC12 - C	3,080,436,051	89,568	1,943,943	1,103,327	332,318	50	315,790	95,136	14	29%	29%	29%	9843.6	9845.8	156.7		84.7%	76.3%	72.3%	70.4%	65.5%	61.6%	55.4%	50.5%	46.6%
	BC13 - Y	3,080,436,051	89,568	2,389,711	1,265,241	393,244	57	353,981	109,303	16	28%	28%	28%	9622.0	9559.4	173.0		84.9%	76.4%	72.5%	70.3%	65.7%	62.1%	55.8%	50.9%	47.2%
	BC14 - Y	3,080,436,051	89,568	1,979,651	1,101,275	347,642	50	304,351	95,463	14	28%	27%	28%	9504.7	9444.1	149.6		83.6%	74.4%	70.4%	68.2%	63.7%	59.6%	53.9%	49.3%	45.6%
	BC19 - C	3,080,436,051	89,568	2,009,942	1,086,007	329,468	49	294,743	90,194	13	27%	27%	27%	9334.1	9415.1	146.2		86.4%	77.1%	72.9%	70.9%	66.0%	62.1%	55.7%	50.7%	46.9%
	BC20 - C	3,080,436,051	89,568	2,678,166	1,505,174	474,109	68	416,166	131,240	19	28%	28%	28%	9509.1	9520.2	206.2		92.2%	83.8%	79.5%	77.3%	73.3%	69.1%	62.9%	58.1%	54.2%
	**ALL**	**3,080,436,051**	**89,568**	**37,197,621**	**19,798,860**	**6,080,436**	**893**	**5,317,746**	**1,615,719**	**240**	**27%**	**27%**	**27%**	**9237.3**	**9138.8**	**162.1**	**2593.7**	**83.0%**	**72.9%**	**68.8%**	**66.6%**	**62.0%**	**58.3%**	**52.5%**	**48.1%**	**44.4%**
**20-plex**	BC1 - Y	3,080,436,051	89,568	4,126,955	2,453,376	763,979	111	161,242	33,433	7	7%	4%	6%	2260.3	1505.1	60.8		87.6%	75.1%	68.3%	64.5%	56.6%	50.6%	42.1%	36.0%	31.1%
	BC2 - Y	3,080,436,051	89,568	1,991,692	1,250,868	405,698	57	89,554	20,754	4	7%	5%	7%	2462.3	1759.4	34.9		80.8%	64.8%	57.0%	52.8%	44.5%	38.3%	29.8%	23.6%	19.4%
	BC3 - C	3,080,436,051	89,568	1,561,461	933,948	300,853	42	59,598	12,917	3	6%	4%	6%	2194.7	1476.6	22.5		75.5%	55.4%	47.7%	43.4%	35.2%	29.0%	20.5%	15.9%	12.4%
	BC4 - C	3,080,436,051	89,568	131,453	76,352	25,250	3	5,577	1,125	0	7%	4%	7%	2512.1	1532.3	1.9		32.1%	10.9%	6.7%	5.6%	3.3%	2.3%	0.9%	0.5%	0.2%
	BC5 - Y	3,080,436,051	89,568	2,139,831	1,260,586	389,943	57	83,509	17,518	3	7%	4%	6%	2278.3	1545.1	30.7		80.1%	62.5%	54.0%	48.9%	40.8%	34.8%	27.0%	21.4%	17.4%
	BC6 - Y	3,080,436,051	89,568	1,508,733	948,873	314,672	43	62,242	14,174	3	7%	5%	6%	2256.0	1549.2	23.3		78.0%	57.4%	48.2%	43.2%	34.4%	28.8%	21.1%	16.2%	13.1%
	BC7 - C	3,080,436,051	89,568	1,292,026	779,109	253,107	35	54,153	11,831	2	7%	5%	6%	2390.5	1607.6	20.0		74.4%	52.2%	43.7%	39.5%	31.5%	26.0%	18.4%	13.7%	10.4%
	BC8 - C	3,080,436,051	89,568	1,268,419	768,829	253,869	35	52,853	11,545	2	7%	5%	6%	2364.3	1564.0	19.6		71.9%	51.7%	43.3%	39.2%	30.7%	25.1%	18.4%	14.0%	10.7%
	BC9 - Y	3,080,436,051	89,568	1,447,438	874,628	288,115	40	55,329	11,478	2	6%	4%	6%	2175.6	1370.1	19.6		73.4%	52.1%	43.4%	38.7%	30.3%	25.0%	17.9%	13.5%	10.7%
	BC10 - Y	3,080,436,051	89,568	1,879,568	1,105,076	360,499	50	69,585	14,616	3	6%	4%	6%	2165.6	1394.4	24.6		75.5%	55.8%	47.4%	43.2%	35.4%	30.0%	22.2%	17.5%	14.0%
	BC11 - C	3,080,436,051	89,568	1,130,415	713,192	239,842	33	53,150	13,106	2	7%	5%	7%	2563.0	1879.3	20.9		75.9%	55.4%	46.3%	41.6%	33.9%	28.0%	20.2%	14.7%	11.4%
	BC12 - C	3,080,436,051	89,568	1,423,950	900,471	280,065	41	63,353	14,661	3	7%	5%	7%	2419.7	1800.4	25.4		80.2%	60.3%	51.4%	47.0%	38.6%	32.2%	23.6%	17.8%	13.9%
	BC13 - Y	3,080,436,051	89,568	1,619,611	1,014,058	335,415	46	70,265	15,970	3	7%	5%	6%	2383.1	1637.5	26.4		78.4%	58.2%	49.6%	45.4%	37.3%	31.5%	23.2%	18.6%	15.2%
	BC14 - Y	3,080,436,051	89,568	1,374,871	873,743	290,001	40	63,007	14,805	3	7%	5%	7%	2480.1	1755.8	24.1		76.2%	57.8%	48.8%	44.5%	36.4%	30.3%	21.7%	16.5%	12.9%
	BC15 - C	3,080,436,051	89,568	2,021,309	1,215,553	382,056	55	78,509	16,882	3	6%	4%	6%	2221.3	1519.7	29.5		79.8%	60.8%	53.0%	48.9%	40.7%	34.7%	25.8%	20.2%	16.6%
	BC16 - C	3,080,436,051	89,568	2,211,808	1,331,904	429,764	61	91,250	20,781	4	7%	5%	6%	2356.2	1663.0	34.5		82.1%	64.2%	55.4%	51.4%	43.6%	37.6%	28.8%	23.0%	18.8%
	BC17 - Y	3,080,436,051	89,568	1,911,811	1,152,099	362,882	52	75,071	16,198	3	7%	4%	6%	2241.0	1535.2	27.9		79.0%	60.0%	51.0%	46.8%	38.4%	32.7%	24.6%	19.3%	15.8%
	BC18 - Y	3,080,436,051	89,568	1,594,490	997,706	333,102	46	66,459	15,350	3	7%	5%	6%	2290.9	1584.9	24.8		79.0%	58.7%	49.8%	44.8%	35.9%	30.2%	22.7%	17.7%	13.9%
	BC19 - C	3,080,436,051	89,568	1,413,120	889,009	281,439	40	57,308	13,663	2	6%	5%	6%	2217.0	1669.6	22.9		79.5%	58.8%	50.0%	45.7%	37.1%	30.8%	22.1%	16.1%	12.2%
	BC20 - C	3,080,436,051	89,568	1,826,532	1,214,136	409,158	56	80,069	19,331	3	7%	5%	6%	2268.1	1624.9	31.2		84.6%	65.4%	56.9%	52.2%	42.7%	36.3%	28.1%	22.4%	18.0%
	**ALL**	**3,080,436,051**	**89,568**	**33,875,493**	**20,753,516**	**6,699,709**	**946**	**1,392,083**	**310,138**	**59**	**7%**	**5%**	**6%**	**2306.9**	**1592.1**	**26.3**	**525.4**	**76.2%**	**56.9%**	**48.6%**	**44.4%**	**36.4%**	**30.7%**	**23.0%**	**17.9%**	**14.4%**
**No BC - Y**	**-**	**3,080,436,051**	**89,568**	**33,216,839**	**19,465,742**	**6,050,455**	**880**	**5,366,668**	**1,735,974**	**247**	**28%**	**29%**	**28%**	**9481.9**	**9867.7**		**2726.5**	**99.6%**	**98.9%**	**98.6%**	**98.4%**	**97.9%**	**97.5%**	**96.6%**	**95.9%**	**95.1%**
**No BC - C**	**-**	**3,080,436,051**	**89,568**	**37,890,578**	**22,414,101**	**7,949,682**	**1,021**	**2,100,645**	**663,192**	**93**	**9%**	**8%**	**9%**	**3223.2**	**2869.1**		**947.9**	**99.6%**	**98.7%**	**98.0%**	**97.6%**	**96.7%**	**95.9%**	**94.4%**	**92.8%**	**91.3%**
**No BC - Y & C**	**Average**	**3,080,436,051**	**89,568**	**35,553,708**	**20,939,921**	**7,000,068**	**950**	**3,733,656**	**1,199,583**	**169**	**18%**	**17%**	**18%**	**6132.2**	**5893.7**		**1786.4**	**99.7%**	**98.8%**	**98.3%**	**98.0%**	**97.3%**	**96.7%**	**95.5%**	**94.3%**	**93.2%**

* herein identical to reads obtained off the SOLiD 3 machine, i.e. without QualityValue-filtering (this filtering is carried out during tertiary analyses, e.g. mapping/pairing/SNP-calling)

24.2 reads are read segments of length 24 which could be mapped with 2 mismatches; this is the shortest allowed read segment in the default mapping settings (see Table T1 in http://www.ikmb.uni-kiel.de/tngs-backmapping/bioscope_settings.xls)

49.0 reads are read segments of length 49 which could be mapped with 0 mismatches; this is the longest possible read segment and the best possible match

This table summarises mapped reads and coverages in detail for all samples. Uniform distributions between barcodes within each pool are achieved for mapped reads, enrichment factors, coverages, and covered bases. For higher plexing, coverages and covered bases are thinner, much as expected. Notably, the 20-plex is significantly below expectations and the 4-plex2 is significantly above expectations.

Table S23a: Detailed summary for corona lite v4.0r2.0 whole genome and target region mapping (fragment length 50, 5 mismatches), all HapMap samples

HapMap YRI NA18507 (Y) & CHB NA18561 (C)	Number of barcodes	Genome Size	HybSelected Region	Reads Obtained (raw reads)*	Reads uniquely mapped to genome	On_Target Reads (unique)	Percent On_Target Reads	Enrichment Factor	Average Depth of Coverage (per barcode)	Average Depth of Coverage (per spot)	1X Consensus Coverage	5X Cons. Cov.	8X Cons. Cov.	10X Cons. Cov.	15X Cons. Cov.	20X Cons. Cov.	30X Cons. Cov.	40X Cons. Cov.	50X Cons. Cov.
4-plex 1	4	3,080,436,051	89,568	28,392,276	11,542,925	2,437,641	21%	7263.0	326.6	1306.6	96.4%	92.0%	89.8%	88.4%	85.5%	83.2%	79.3%	75.5%	72.2%
4-plex 2	4	3,080,436,051	89,568	34,631,217	12,915,844	4,795,435	37%	12769.2	653.9	2615.7	95.6%	92.1%	90.3%	89.3%	87.4%	85.6%	82.6%	80.0%	77.7%
8-plex	8	3,080,436,051	89,568	29,352,928	11,376,966	3,884,341	34%	11742.2	263.6	2108.7	82.7%	75.9%	73.0%	71.4%	68.1%	65.3%	60.5%	56.6%	53.3%
16-plex	16	3,080,436,051	89,568	37,197,621	13,535,119	4,282,527	32%	10881.7	145.4	2327.2	79.5%	69.2%	65.1%	63.0%	58.6%	55.0%	49.4%	45.0%	41.4%
20-plex	20	3,080,436,051	89,568	33,875,493	5,450,939	913,163	17%	5761.5	23.0	459.2	72.0%	52.3%	44.4%	40.4%	32.8%	27.5%	20.4%	15.7%	12.5%
No BC - Y	-	3,080,436,051	89,568	33,216,839	13,976,509	4,465,562	32%	10988.5	-	2454.8	99.0%	97.7%	97.2%	96.8%	96.0%	95.5%	94.6%	93.6%	92.7%
No BC - C	-	3,080,436,051	89,568	37,890,578	15,955,304	1,603,486	10%	3456.4	-	854.7	98.9%	97.2%	96.1%	95.7%	94.6%	93.5%	91.4%	89.3%	87.6%
**no BC - averaged**	**-**	**3,080,436,051**	**89,568**	**35,553,708**	**14,965,906**	**3,034,524**	**20%**	**6973.4**	**-**	**1609.1**	**99.7%**	**97.4%**	**96.6%**	**96.3%**	**95.3%**	**94.5%**	**93.0%**	**91.5%**	**90.2%**

	Barcode	Genome Size	HybSelected Region	Reads Obtained (raw reads)*	Reads uniquely mapped to genome	On_Target Reads (unique)	Percent On_Target Reads	Enrichment Factor	Average Depth of Coverage (per barcode)	Average Depth of Coverage (per spot)	1X Consensus Coverage	5X Cons. Cov.	8X Cons. Cov.	10X Cons. Cov.	15X Cons. Cov.	20X Cons. Cov.	30X Cons. Cov.	40X Cons. Cov.	50X Cons. Cov.
**4-plex 1**	BC5 - Y	3,080,436,051	89,568	9,096,031	3,508,755	761,443	22%	7463.5	408.6		97.0%	93.5%	91.7%	90.5%	88.0%	85.9%	82.4%	79.1%	76.2%
	BC6 - Y	3,080,436,051	89,568	6,611,794	2,796,177	602,317	22%	7408.3	323.3		96.7%	92.8%	90.5%	89.2%	86.4%	83.9%	80.1%	76.3%	73.2%
	BC7 - C	3,080,436,051	89,568	6,076,429	2,558,796	521,616	20%	7010.9	279.3		95.9%	90.8%	88.4%	86.9%	83.8%	81.4%	77.3%	73.2%	69.8%
	BC8 - C	3,080,436,051	89,568	6,608,022	2,679,197	552,265	21%	7089.3	295.3		95.9%	91.1%	88.4%	87.0%	83.9%	81.4%	77.4%	73.3%	69.8%
	**ALL**	**3,080,436,051**	**89,568**	**28,392,276**	**11,542,925**	**2,437,641**	**21%**	**7263.0**	**326.6**	**1306.6**	**96.4%**	**92.0%**	**89.8%**	**88.4%**	**85.5%**	**83.2%**	**79.3%**	**75.5%**	**72.2%**
**4-plex 2**	BC7 - C	3,080,436,051	89,568	7,961,278	3,034,236	1,169,339	39%	13254.1	638.0		95.9%	92.6%	90.8%	89.8%	87.8%	85.7%	82.8%	80.3%	78.1%
	BC8 - C	3,080,436,051	89,568	8,900,065	3,360,268	1,212,822	36%	12413.2	661.2		95.8%	92.5%	90.7%	89.6%	87.8%	86.1%	83.0%	80.5%	78.2%
	BC9 - Y	3,080,436,051	89,568	8,028,156	2,949,246	1,064,335	36%	12411.6	580.6		95.5%	91.5%	89.4%	88.4%	86.2%	84.4%	81.3%	78.3%	75.7%
	BC10 - Y	3,080,436,051	89,568	9,741,718	3,572,094	1,348,939	38%	12987.6	735.9		95.3%	92.0%	90.5%	89.5%	87.7%	85.9%	83.3%	80.8%	78.6%
	**ALL**	**3,080,436,051**	**89,568**	**34,631,217**	**12,915,844**	**4,795,435**	**37%**	**12769.2**	**653.9**	**2615.7**	**95.6%**	**92.1%**	**90.3%**	**89.3%**	**87.4%**	**85.6%**	**82.6%**	**80.0%**	**77.7%**
**8-plex**	BC1 - Y	3,080,436,051	89,568	6,004,455	2,309,711	821,291	36%	12229.2	446.4		89.8%	85.9%	83.7%	82.3%	79.9%	77.5%	73.6%	70.4%	67.1%
	BC2 - Y	3,080,436,051	89,568	4,008,564	1,643,002	600,633	37%	12572.8	326.5		88.1%	83.5%	80.5%	79.0%	76.1%	73.3%	68.9%	65.1%	61.9%
	BC3 - C	3,080,436,051	89,568	4,114,328	1,548,483	481,631	31%	10697.1	261.9		85.4%	79.8%	76.9%	75.1%	71.7%	68.7%	63.8%	59.6%	56.5%
	BC4 - C	3,080,436,051	89,568	290,433	112,589	37,401	33%	11424.7	20.1		53.3%	35.4%	31.6%	29.7%	25.5%	23.0%	18.6%	15.1%	12.0%
	BC5 - Y	3,080,436,051	89,568	4,880,335	1,788,461	608,042	34%	11692.7	329.9		88.0%	83.3%	81.0%	79.6%	76.5%	73.9%	68.7%	64.8%	61.5%
	BC6 - Y	3,080,436,051	89,568	3,626,078	1,494,162	507,030	34%	11670.7	275.0		87.6%	82.1%	78.8%	77.1%	74.4%	71.3%	66.3%	62.2%	59.1%
	BC7 - C	3,080,436,051	89,568	3,052,954	1,207,766	404,250	33%	11511.4	219.0		84.9%	79.0%	75.9%	74.2%	70.5%	67.4%	62.1%	57.7%	54.1%
	BC8 - C	3,080,436,051	89,568	3,375,781	1,272,792	424,063	33%	11458.6	229.9		84.2%	78.2%	75.7%	74.2%	70.6%	67.1%	61.7%	57.6%	54.1%
	**ALL**	**3,080,436,051**	**89,568**	**29,352,928**	**11,376,966**	**3,884,341**	**34%**	**11742.2**	**263.6**	**2108.7**	**82.7%**	**75.9%**	**73.0%**	**71.4%**	**68.1%**	**65.3%**	**60.5%**	**56.6%**	**53.3%**
**16-plex**	BC1 - Y	3,080,436,051	89,568	4,899,027	1,763,942	568,303	32%	11080.4	309.0		88.7%	81.3%	78.2%	76.6%	72.6%	69.8%	65.0%	61.4%	58.1%
	BC2 - Y	3,080,436,051	89,568	2,676,886	1,012,073	321,712	32%	10932.4	174.7		82.6%	74.2%	70.5%	68.4%	64.2%	60.7%	55.4%	50.9%	47.5%
	BC3 - C	3,080,436,051	89,568	2,668,379	892,992	253,695	28%	9770.7	137.9		81.7%	70.7%	66.1%	64.0%	59.2%	55.5%	50.2%	45.7%	42.2%
	BC4 - C	3,080,436,051	89,568	192,251	69,348	21,245	31%	10536.2	11.4		46.0%	25.8%	21.4%	19.5%	16.1%	14.0%	10.2%	7.9%	6.3%
	BC5 - Y	3,080,436,051	89,568	3,195,035	1,105,138	348,896	32%	10857.7	189.6		83.6%	74.1%	70.1%	67.9%	63.6%	60.0%	55.2%	51.1%	47.1%
	BC6 - Y	3,080,436,051	89,568	2,137,151	808,725	247,362	31%	10519.4	134.3		81.5%	71.4%	67.4%	65.0%	60.7%	57.2%	50.9%	45.9%	41.9%
	BC7 - C	3,080,436,051	89,568	2,046,478	717,859	218,117	30%	10449.8	118.2		78.0%	67.4%	62.9%	60.8%	56.1%	52.2%	46.5%	41.8%	38.0%
	BC8 - C	3,080,436,051	89,568	2,009,518	717,838	221,620	31%	10618.0	120.2		78.4%	66.8%	62.3%	60.3%	55.3%	51.2%	45.4%	40.8%	36.9%
	BC9 - Y	3,080,436,051	89,568	1,880,236	682,333	201,609	30%	10161.9	109.2		75.8%	64.6%	60.7%	58.3%	53.2%	49.1%	44.1%	39.8%	36.7%
	BC10 - Y	3,080,436,051	89,568	2,779,455	898,160	276,277	31%	10579.1	149.6		79.0%	69.5%	65.8%	63.7%	59.2%	56.1%	50.6%	46.2%	42.3%
	BC11 - C	3,080,436,051	89,568	1,711,792	664,388	226,251	34%	11711.9	123.0		81.4%	71.4%	67.2%	65.0%	59.9%	56.2%	49.9%	45.2%	41.4%
	BC12 - C	3,080,436,051	89,568	1,943,943	769,503	257,387	33%	11503.6	140.5		80.8%	72.7%	68.7%	66.6%	62.3%	58.3%	51.9%	47.1%	43.2%
	BC13 - Y	3,080,436,051	89,568	2,389,711	863,430	287,078	33%	11434.9	155.9		81.7%	72.5%	68.6%	66.8%	62.5%	58.8%	52.6%	47.7%	44.2%
	BC14 - Y	3,080,436,051	89,568	1,979,651	767,513	248,869	32%	11151.8	135.4		80.1%	70.7%	66.8%	64.7%	60.2%	56.4%	50.6%	46.4%	42.8%
	BC19 - C	3,080,436,051	89,568	2,009,942	747,860	240,866	32%	11076.8	131.3		82.7%	73.1%	69.1%	67.2%	62.3%	58.5%	52.3%	47.4%	44.0%
	BC20 - C	3,080,436,051	89,568	2,678,166	1,054,017	343,240	33%	11199.8	187.0		89.5%	80.3%	76.1%	74.0%	69.7%	65.5%	59.7%	54.6%	50.6%
	**ALL**	**3,080,436,051**	**89,568**	**37,197,621**	**13,535,119**	**4,282,527**	**32%**	**10881.7**	**145.4**	**2327.2**	**79.5%**	**69.2%**	**65.1%**	**63.0%**	**58.6%**	**55.0%**	**49.4%**	**45.0%**	**41.4%**
**20-plex**	BC1 - Y	3,080,436,051	89,568	4,126,955	622,555	104,443	17%	5769.8	52.6		83.9%	70.4%	63.4%	59.5%	51.4%	46.0%	37.9%	32.3%	27.7%
	BC2 - Y	3,080,436,051	89,568	1,991,692	330,256	60,206	18%	6269.7	30.6		76.6%	59.7%	52.2%	48.3%	40.5%	34.8%	26.5%	20.9%	17.3%
	BC3 - C	3,080,436,051	89,568	1,561,461	244,059	38,838	16%	5472.9	19.6		70.9%	51.4%	43.7%	39.6%	31.7%	25.8%	18.1%	13.6%	10.4%
	BC4 - C	3,080,436,051	89,568	131,453	20,205	3,535	17%	6017.1	1.7		28.9%	9.4%	6.2%	5.2%	3.0%	2.0%	0.8%	0.4%	0.2%
	BC5 - Y	3,080,436,051	89,568	2,139,831	316,510	53,289	17%	5790.4	26.5		75.9%	57.2%	48.6%	44.3%	36.9%	31.5%	24.2%	18.7%	15.0%
	BC6 - Y	3,080,436,051	89,568	1,508,733	255,951	40,744	16%	5474.8	20.4		73.8%	52.4%	43.5%	39.0%	30.8%	25.7%	18.8%	14.2%	11.3%
	BC7 - C	3,080,436,051	89,568	1,292,026	204,759	34,862	17%	5855.6	17.3		69.8%	47.6%	39.8%	36.0%	28.3%	22.9%	15.9%	11.7%	9.1%
	BC8 - C	3,080,436,051	89,568	1,268,419	205,238	34,397	17%	5764.0	17.1		67.3%	47.3%	39.4%	35.2%	27.4%	22.5%	16.0%	11.9%	9.2%
	BC9 - Y	3,080,436,051	89,568	1,447,438	233,302	34,848	15%	5137.1	17.1		69.6%	47.8%	39.1%	35.1%	27.0%	22.3%	15.7%	11.8%	9.0%
	BC10 - Y	3,080,436,051	89,568	1,879,568	291,577	43,554	15%	5137.3	21.4		71.8%	51.6%	43.4%	39.1%	32.0%	26.8%	19.6%	15.3%	11.9%
	BC11 - C	3,080,436,051	89,568	1,130,415	195,219	36,319	19%	6398.4	18.5		71.6%	50.3%	41.9%	37.9%	30.5%	25.0%	17.7%	12.9%	9.8%
	BC12 - C	3,080,436,051	89,568	1,423,950	229,965	43,307	19%	6476.7	22.2		75.5%	55.7%	46.7%	42.4%	34.7%	28.6%	20.5%	15.1%	11.9%
	BC13 - Y	3,080,436,051	89,568	1,619,611	271,932	46,065	17%	5826.0	23.1		73.7%	53.3%	45.6%	41.4%	33.7%	28.2%	20.8%	16.7%	13.3%
	BC14 - Y	3,080,436,051	89,568	1,374,871	235,356	42,278	18%	6178.0	21.4		71.9%	53.5%	45.0%	40.8%	33.1%	27.1%	19.2%	14.4%	11.1%
	BC15 - C	3,080,436,051	89,568	2,021,309	311,022	51,092	16%	5649.6	25.7		75.6%	56.4%	48.2%	44.4%	37.0%	31.3%	23.0%	17.9%	14.2%
	BC16 - C	3,080,436,051	89,568	2,211,808	349,119	60,078	17%	5918.4	30.2		78.0%	59.3%	51.0%	47.2%	39.6%	33.9%	25.8%	20.0%	16.2%
	BC17 - Y	3,080,436,051	89,568	1,911,811	295,946	48,375	16%	5621.7	24.2		74.8%	55.0%	46.6%	42.4%	34.6%	29.1%	21.9%	17.0%	13.7%
	BC18 - Y	3,080,436,051	89,568	1,594,490	270,969	43,711	16%	5547.9	21.9		74.3%	53.7%	45.7%	40.6%	32.6%	27.2%	20.3%	15.5%	12.1%
	BC19 - C	3,080,436,051	89,568	1,413,120	231,732	38,917	17%	5775.8	20.0		75.1%	53.8%	45.9%	41.5%	33.1%	27.1%	19.5%	13.7%	10.5%
	BC20 - C	3,080,436,051	89,568	1,826,532	335,267	54,305	16%	5570.7	27.7		80.6%	60.3%	52.0%	47.5%	38.6%	32.7%	24.9%	19.8%	15.8%
	**ALL**	**3,080,436,051**	**89,568**	**33,875,493**	**5,450,939**	**913,163**	**17%**	**5761.5**	**23.0**	**459.2**	**72.0%**	**52.3%**	**44.4%**	**40.4%**	**32.8%**	**27.5%**	**20.4%**	**15.7%**	**12.5%**
**No BC - Y**	**-**	**3,080,436,051**	**89,568**	**33,216,839**	**13,976,509**	**4,465,562**	**32%**	**10988.5**		**2454.8**	**99.0%**	**97.7%**	**97.2%**	**96.8%**	**96.0%**	**95.5%**	**94.6%**	**93.6%**	**92.7%**
**No BC - C**	**-**	**3,080,436,051**	**89,568**	**37,890,578**	**15,955,304**	**1,603,486**	**10%**	**3456.4**		**854.7**	**98.9%**	**97.2%**	**96.1%**	**95.7%**	**94.6%**	**93.5%**	**91.4%**	**89.3%**	**87.6%**
**No BC - Y & C**	**Average**	**3,080,436,051**	**89,568**	**35,553,708**	**14,965,906**	**3,034,524**	**20%**	**6973.4**		**1609.1**	**99.7%**	**97.4%**	**96.6%**	**96.3%**	**95.3%**	**94.5%**	**93.0%**	**91.5%**	**90.2%**

* herein identical to reads obtained off the SOLiD3 machine, i.e. without QualityValue-filtering (this filtering is carried out during tertiary analyses, e.g. mapping/pairing/SNP-calling)

Table S23b: Detailed summary for corona lite v4.0r2.0 target region mapping (fragment length 50, 4 mismatches), all HapMap samples

HapMap YRI NA18507 (Y) & CHB NA18561 (C)	Number of barcodes	Genome Size	HybSelected Region	Reads Obtained (raw reads)*	On_Target Reads (unique)	Average Depth of Coverage (per barcode)	Average Depth of Coverage (per spot)	1X Consensus Coverage	5X Cons. Cov.	8X Cons. Cov.	10X Cons. Cov.	15X Cons. Cov.	20X Cons. Cov.	30X Cons. Cov.	40X Cons. Cov.	50X Cons. Cov.
4-plex 1	4	3,080,436,051	89,568	28,392,276	2,245,534	304.8	1219.3	95.8%	91.1%	88.7%	87.4%	84.4%	81.9%	77.7%	73.8%	70.5%
4-plex 2	4	3,080,436,051	89,568	34,631,217	4,366,642	599.1	2396.4	95.0%	91.3%	89.4%	88.3%	86.1%	84.2%	81.1%	78.3%	75.9%
8-plex	8	3,080,436,051	89,568	29,352,928	3,582,298	245.1	1960.9	81.7%	74.7%	71.8%	70.2%	66.7%	63.8%	59.0%	54.9%	51.5%
16-plex	16	3,080,436,051	89,568	37,197,621	3,927,326	134.5	2151.3	78.1%	67.6%	63.5%	61.4%	56.8%	53.2%	47.6%	43.3%	39.7%
20-plex	20	3,080,436,051	89,568	33,875,493	804,301	21.0	420.5	70.3%	50.3%	42.4%	38.5%	31.1%	25.9%	18.9%	14.5%	11.5%
No BC - Y	-	3,080,436,051	89,568	33,216,839	4,132,386	-	2278.3	98.8%	97.3%	96.6%	96.2%	95.5%	95.0%	93.9%	92.9%	91.9%
No BC - C	-	3,080,436,051	89,568	37,890,578	1,468,434	-	795.2	98.7%	96.7%	95.6%	95.0%	93.7%	92.5%	90.1%	88.0%	86.3%
**no BC - averaged**	**-**	**3,080,436,051**	**89,568**	**35,553,708**	**2,800,410**	**-**	**1494.4**	**99.7%**	**97.0%**	**96.1%**	**95.6%**	**94.6%**	**93.7%**	**92.0%**	**90.4%**	**89.1%**

	Barcode	Genome Size	HybSelected Region	Reads Obtained (raw reads)*	On_Target Reads (unique)	Average Depth of Coverage (per barcode)	Average Depth of Coverage (per spot)	1X Consensus Coverage	5X Cons. Cov.	8X Cons. Cov.	10X Cons. Cov.	15X Cons. Cov.	20X Cons. Cov.	30X Cons. Cov.	40X Cons. Cov.	50X Cons. Cov.
**4-plex 1**	BC5 - Y	3,080,436,051	89,568	9,096,031	699,918	380.4		96.7%	92.7%	90.6%	89.5%	86.9%	84.6%	80.9%	77.3%	74.2%
	BC6 - Y	3,080,436,051	89,568	6,611,794	556,109	302.3		96.1%	92.0%	89.5%	88.2%	85.1%	82.8%	78.4%	74.7%	71.5%
	BC7 - C	3,080,436,051	89,568	6,076,429	481,407	261.2		95.3%	89.8%	87.4%	85.9%	82.8%	80.2%	75.8%	71.6%	68.1%
	BC8 - C	3,080,436,051	89,568	6,608,022	508,100	275.4		95.3%	90.1%	87.5%	85.9%	82.7%	80.1%	75.8%	71.4%	68.0%
	**ALL**	**3,080,436,051**	**89,568**	**28,392,276**	**2,245,534**	**304.8**	**1219.3**	**95.8%**	**91.1%**	**88.7%**	**87.4%**	**84.4%**	**81.9%**	**77.7%**	**73.8%**	**70.5%**
**4-plex 2**	BC7 - C	3,080,436,051	89,568	7,961,278	1,065,538	584.7		95.4%	91.8%	90.0%	88.8%	86.4%	84.4%	81.3%	78.7%	76.3%
	BC8 - C	3,080,436,051	89,568	8,900,065	1,104,445	605.9		95.3%	91.6%	89.7%	88.7%	86.8%	84.8%	81.5%	78.8%	76.5%
	BC9 - Y	3,080,436,051	89,568	8,028,156	968,303	531.5		94.6%	90.5%	88.3%	87.3%	84.8%	83.0%	79.7%	76.6%	73.8%
	BC10 - Y	3,080,436,051	89,568	9,741,718	1,228,356	674.2		94.6%	91.2%	89.6%	88.7%	86.5%	84.8%	81.9%	79.2%	76.9%
	**ALL**	**3,080,436,051**	**89,568**	**34,631,217**	**4,366,642**	**599.1**	**2396.4**	**95.0%**	**91.3%**	**89.4%**	**88.3%**	**86.1%**	**84.2%**	**81.1%**	**78.3%**	**75.9%**
**8-plex**	BC1 - Y	3,080,436,051	89,568	6,004,455	757,054	414.6		89.1%	84.5%	82.4%	81.0%	78.3%	75.9%	72.2%	68.8%	65.2%
	BC2 - Y	3,080,436,051	89,568	4,008,564	555,855	304.4		86.9%	82.1%	79.3%	77.8%	74.5%	71.5%	67.1%	63.4%	60.0%
	BC3 - C	3,080,436,051	89,568	4,114,328	443,259	243.0		84.6%	78.6%	75.8%	73.9%	70.3%	67.2%	62.1%	58.1%	54.7%
	BC4 - C	3,080,436,051	89,568	290,433	34,529	18.8		52.1%	34.5%	30.8%	28.8%	24.6%	22.1%	17.8%	14.1%	11.2%
	BC5 - Y	3,080,436,051	89,568	4,880,335	558,842	305.8		87.0%	82.3%	79.7%	78.2%	75.1%	72.4%	67.3%	63.0%	59.4%
	BC6 - Y	3,080,436,051	89,568	3,626,078	468,767	256.5		86.7%	80.8%	77.6%	75.9%	72.9%	69.7%	64.9%	60.4%	57.1%
	BC7 - C	3,080,436,051	89,568	3,052,954	373,103	203.9		84.0%	77.7%	74.5%	72.8%	69.3%	65.9%	60.3%	55.8%	52.2%
	BC8 - C	3,080,436,051	89,568	3,375,781	390,889	213.9		83.3%	77.3%	74.5%	72.8%	69.0%	65.5%	60.0%	55.8%	52.3%
	**ALL**	**3,080,436,051**	**89,568**	**29,352,928**	**3,582,298**	**245.1**	**1960.9**	**81.7%**	**74.7%**	**71.8%**	**70.2%**	**66.7%**	**63.8%**	**59.0%**	**54.9%**	**51.5%**
**16-plex**	BC1 - Y	3,080,436,051	89,568	4,899,027	520,213	285.1		87.6%	79.9%	76.8%	74.9%	71.0%	68.0%	63.2%	59.6%	56.0%
	BC2 - Y	3,080,436,051	89,568	2,676,886	294,618	161.2		80.8%	72.5%	68.8%	66.8%	62.4%	58.8%	53.8%	49.3%	45.5%
	BC3 - C	3,080,436,051	89,568	2,668,379	231,767	127.0		80.1%	68.7%	64.3%	62.0%	57.4%	54.0%	48.4%	43.9%	40.7%
	BC4 - C	3,080,436,051	89,568	192,251	19,534	10.6		44.5%	24.8%	20.5%	18.9%	15.6%	13.0%	9.5%	7.5%	5.9%
	BC5 - Y	3,080,436,051	89,568	3,195,035	318,951	174.7		82.5%	72.6%	68.4%	66.3%	61.6%	58.4%	53.3%	49.1%	45.4%
	BC6 - Y	3,080,436,051	89,568	2,137,151	227,005	124.4		80.0%	69.8%	65.6%	63.4%	59.1%	55.2%	49.1%	44.1%	40.0%
	BC7 - C	3,080,436,051	89,568	2,046,478	199,642	109.2		76.6%	65.8%	61.1%	59.1%	54.2%	50.6%	44.8%	39.9%	36.3%
	BC8 - C	3,080,436,051	89,568	2,009,518	203,200	111.1		76.9%	65.2%	60.9%	58.5%	53.4%	49.5%	43.4%	39.1%	35.5%
	BC9 - Y	3,080,436,051	89,568	1,880,236	184,861	101.1		74.2%	63.0%	58.8%	56.5%	51.2%	47.6%	42.7%	38.5%	34.9%
	BC10 - Y	3,080,436,051	89,568	2,779,455	252,092	137.8		77.2%	68.0%	64.5%	62.2%	57.7%	54.6%	48.8%	44.4%	40.6%
	BC11 - C	3,080,436,051	89,568	1,711,792	208,097	114.0		79.9%	69.8%	65.5%	63.5%	58.2%	54.4%	47.9%	43.5%	39.4%
	BC12 - C	3,080,436,051	89,568	1,943,943	236,475	129.9		79.8%	70.8%	67.0%	64.9%	60.5%	56.5%	49.9%	45.1%	41.1%
	BC13 - Y	3,080,436,051	89,568	2,389,711	263,362	144.2		80.6%	70.9%	67.0%	65.0%	60.9%	56.8%	50.6%	45.9%	42.3%
	BC14 - Y	3,080,436,051	89,568	1,979,651	229,240	125.7		79.0%	69.0%	65.1%	62.9%	58.2%	54.2%	48.8%	44.7%	40.9%
	BC19 - C	3,080,436,051	89,568	2,009,942	221,572	121.5		81.5%	71.5%	67.3%	65.4%	60.4%	56.5%	50.4%	45.8%	42.0%
	BC20 - C	3,080,436,051	89,568	2,678,166	316,697	173.7		88.6%	78.7%	74.7%	72.6%	67.7%	63.6%	57.6%	52.5%	48.3%
	**ALL**	**3,080,436,051**	**89,568**	**37,197,621**	**3,927,326**	**134.5**	**2151.3**	**78.1%**	**67.6%**	**63.5%**	**61.4%**	**56.8%**	**53.2%**	**47.6%**	**43.3%**	**39.7%**
**20-plex**	BC1 - Y	3,080,436,051	89,568	4,126,955	91,534	47.9		82.5%	68.2%	61.0%	57.0%	49.2%	43.9%	36.0%	30.3%	25.7%
	BC2 - Y	3,080,436,051	89,568	1,991,692	53,569	28.2		75.0%	57.7%	50.5%	46.3%	38.7%	33.1%	24.9%	19.5%	15.9%
	BC3 - C	3,080,436,051	89,568	1,561,461	34,224	17.9		69.1%	49.3%	41.5%	37.7%	30.0%	24.1%	16.5%	12.4%	9.6%
	BC4 - C	3,080,436,051	89,568	131,453	3,001	1.5		27.1%	8.8%	5.7%	4.7%	2.8%	1.7%	0.7%	0.3%	0.2%
	BC5 - Y	3,080,436,051	89,568	2,139,831	46,581	24.2		74.6%	54.9%	46.2%	42.2%	34.9%	30.0%	22.5%	17.3%	13.8%
	BC6 - Y	3,080,436,051	89,568	1,508,733	36,045	18.8		71.9%	50.2%	41.4%	37.3%	29.2%	24.1%	17.5%	12.9%	10.4%
	BC7 - C	3,080,436,051	89,568	1,292,026	30,496	15.8		67.9%	45.5%	38.0%	34.0%	26.6%	21.6%	14.6%	10.7%	8.3%
	BC8 - C	3,080,436,051	89,568	1,268,419	30,119	15.6		65.4%	45.5%	37.4%	33.5%	25.8%	21.0%	14.5%	10.9%	8.4%
	BC9 - Y	3,080,436,051	89,568	1,447,438	30,208	15.6		67.6%	45.5%	37.4%	33.3%	25.5%	20.7%	14.2%	10.6%	8.1%
	BC10 - Y	3,080,436,051	89,568	1,879,568	38,029	19.5		70.3%	49.8%	41.4%	37.2%	30.5%	25.2%	18.3%	14.1%	11.0%
	BC11 - C	3,080,436,051	89,568	1,130,415	32,458	17.1		70.0%	48.4%	40.2%	36.1%	28.9%	23.6%	16.4%	12.0%	9.2%
	BC12 - C	3,080,436,051	89,568	1,423,950	38,397	20.3		73.8%	53.5%	44.4%	40.5%	32.7%	26.6%	18.9%	13.8%	10.8%
	BC13 - Y	3,080,436,051	89,568	1,619,611	40,496	21.2		72.2%	51.6%	43.6%	39.6%	32.1%	26.7%	19.4%	15.5%	11.9%
	BC14 - Y	3,080,436,051	89,568	1,374,871	37,243	19.6		70.5%	51.0%	43.0%	39.0%	31.1%	25.1%	17.6%	13.2%	10.4%
	BC15 - C	3,080,436,051	89,568	2,021,309	44,873	23.5		73.9%	54.3%	46.4%	42.3%	35.1%	29.4%	21.3%	16.4%	12.8%
	BC16 - C	3,080,436,051	89,568	2,211,808	52,810	27.6		76.1%	57.3%	48.9%	44.9%	37.6%	32.3%	23.8%	18.5%	14.8%
	BC17 - Y	3,080,436,051	89,568	1,911,811	42,534	22.2		73.3%	52.9%	44.6%	40.4%	32.6%	27.4%	20.4%	15.9%	12.6%
	BC18 - Y	3,080,436,051	89,568	1,594,490	38,602	20.1		72.7%	51.6%	43.4%	38.8%	31.0%	25.8%	19.1%	14.3%	11.0%
	BC19 - C	3,080,436,051	89,568	1,413,120	34,598	18.3		73.3%	51.6%	44.0%	39.9%	30.8%	25.3%	17.9%	12.5%	9.6%
	BC20 - C	3,080,436,051	89,568	1,826,532	48,484	25.6		78.8%	58.1%	49.8%	45.3%	36.8%	31.2%	23.3%	18.3%	14.6%
	**ALL**	**3,080,436,051**	**89,568**	**33,875,493**	**804,301**	**21.0**	**420.5**	**70.3%**	**50.3%**	**42.4%**	**38.5%**	**31.1%**	**25.9%**	**18.9%**	**14.5%**	**11.5%**
**No BC - Y**	**-**	**3,080,436,051**	**89,568**	**33,216,839**	**4,132,386**		**2278.3**	**98.8%**	**97.3%**	**96.6%**	**96.2%**	**95.5%**	**95.0%**	**93.9%**	**92.9%**	**91.9%**
**No BC - C**	**-**	**3,080,436,051**	**89,568**	**37,890,578**	**1,468,434**		**795.2**	**98.7%**	**96.7%**	**95.6%**	**95.0%**	**93.7%**	**92.5%**	**90.1%**	**88.0%**	**86.3%**
**No BC - Y & C**	**Average**	**3,080,436,051**	**89,568**	**35,553,708**	**2,800,410**		**1494.4**	**99.7%**	**97.0%**	**96.1%**	**95.6%**	**94.6%**	**93.7%**	**92.0%**	**90.4%**	**89.1%**

* herein identical to reads obtained off the SOLiD3 machine, i.e. without QualityValue-filtering (this filtering is carried out during tertiary analyses, e.g. mapping/pairing/SNP-calling)

Table S24: Detailed summary for SAET2.2 / corona lite v4.0r2.0 whole genome and target region mapping (fragment length 50, 5 mismatches), all HapMap samples

HapMap YRI NA18507 (Y) & CHB NA18561 (C)	Number of barcodes	Genome Size	HybSelected Region	Reads Obtained (raw reads)*	Reads uniquely mapped to genome	On-Target Reads (unique)	Percent On-Target Reads	Enrichment Factor	Average Depth of Coverage (per barcode)	Average Depth of Coverage (per spot)	1X Consensus Coverage	5X Cons. Cov.	8X Cons. Cov.	10X Cons. Cov.	15X Cons. Cov.	20X Cons. Cov.	30X Cons. Cov.	40X Cons. Cov.	50X Cons. Cov.
4-plex 1	4	3,080,436,051	89,568	28,392,276	11,542,925	2,859,198	25%	8519.0	378.3	1513.1	96.5%	92.1%	89.8%	88.5%	85.7%	83.4%	79.6%	76.0%	72.9%
4-plex 2	4	3,080,436,051	89,568	34,631,217	12,915,844	5,857,843	45%	15598.2	793.6	3174.4	95.7%	92.3%	90.5%	89.5%	87.6%	85.8%	82.9%	80.5%	78.3%
8-plex	8	3,080,436,051	89,568	29,352,928	11,376,966	4,578,087	40%	13839.4	308.0	2463.8	82.9%	76.2%	73.4%	71.8%	68.6%	65.8%	61.3%	57.6%	54.5%
16-plex	16	3,080,436,051	89,568	37,197,621	13,535,119	5,156,758	38%	13103.1	173.7	2779.3	79.7%	69.5%	65.5%	63.4%	59.2%	55.8%	50.9%	47.0%	43.7%
20-plex	20	3,080,436,051	89,568	33,875,493	5,450,939	1,093,101	20%	6896.8	26.4	528.4	72.2%	52.5%	44.7%	40.9%	33.9%	29.0%	22.2%	17.6%	14.4%
No BC - Y	-	3,080,436,051	89,568	33,216,839	13,976,509	5,583,805	40%	13740.1	0.0	2909.0	99.1%	97.9%	97.3%	97.0%	96.3%	95.7%	94.7%	93.9%	93.0%
No BC - C	-	3,080,436,051	89,568	37,890,578	15,955,304	2,121,891	13%	4573.8	0.0	977.9	98.9%	97.2%	96.2%	95.8%	94.8%	93.7%	91.5%	89.4%	87.7%
**no BC - averaged**	**-**	**3,080,436,051**	**89,568**	**35,553,708**	**14,965,906**	**3,852,848**	**26%**	**8854.0**	**0.0**	**1888.1**	**99.7%**	**97.5%**	**96.8%**	**96.4%**	**95.5%**	**94.7%**	**93.1%**	**91.7%**	**90.4%**

	Barcode	Genome Size	HybSelected Region	Reads Obtained (raw reads)*	Reads uniquely mapped to genome	On-Target Reads (unique)	Percent On-Target Reads	Enrichment Factor	Average Depth of Coverage (per barcode)	Average Depth of Coverage (per spot)	1X Consensus Coverage	5X Cons. Cov.	8X Cons. Cov.	10X Cons. Cov.	15X Cons. Cov.	20X Cons. Cov.	30X Cons. Cov.	40X Cons. Cov.	50X Cons. Cov.
**4-plex 1**	BC5 - Y	3,080,436,051	89,568	9,096,031	3,508,755	898,412	26%	8806.1	476.0		97.1%	93.5%	91.7%	90.5%	88.1%	86.2%	82.7%	79.4%	76.7%
	BC6 - Y	3,080,436,051	89,568	6,611,794	2,796,177	702,989	25%	8646.6	372.8		96.8%	92.9%	90.6%	89.3%	86.6%	84.1%	80.4%	76.9%	74.0%
	BC7 - C	3,080,436,051	89,568	6,076,429	2,558,796	608,673	24%	8181.0	321.7		96.1%	90.9%	88.5%	87.0%	84.1%	81.7%	77.7%	73.7%	70.4%
	BC8 - C	3,080,436,051	89,568	6,608,022	2,679,197	649,124	24%	8332.6	342.5		95.9%	91.2%	88.5%	87.1%	84.0%	81.5%	77.7%	73.9%	70.5%
	**ALL**	**3,080,436,051**	**89,568**	**28,392,276**	**11,542,925**	**2,859,198**	**25%**	**8519.0**	**378.3**	**1513.1**	**96.5%**	**92.1%**	**89.8%**	**88.5%**	**85.7%**	**83.4%**	**79.6%**	**76.0%**	**72.9%**
**4-plex 2**	BC7 - C	3,080,436,051	89,568	7,961,278	3,034,236	1,421,235	47%	16109.3	770.7		96.1%	92.7%	91.0%	89.9%	87.9%	85.9%	83.0%	80.8%	78.7%
	BC8 - C	3,080,436,051	89,568	8,900,065	3,360,268	1,480,302	44%	15150.8	801.8		95.9%	92.7%	90.8%	89.8%	88.1%	86.5%	83.4%	80.9%	78.9%
	BC9 - Y	3,080,436,051	89,568	8,028,156	2,949,246	1,305,502	44%	15223.9	707.4		95.5%	91.5%	89.5%	88.5%	86.3%	84.6%	81.7%	79.0%	76.4%
	BC10 - Y	3,080,436,051	89,568	9,741,718	3,572,094	1,650,804	46%	15894.0	894.6		95.4%	92.2%	90.6%	89.7%	87.9%	86.2%	83.4%	81.2%	79.2%
	**ALL**	**3,080,436,051**	**89,568**	**34,631,217**	**12,915,844**	**5,857,843**	**45%**	**15598.2**	**793.6**	**3174.4**	**95.7%**	**92.3%**	**90.5%**	**89.5%**	**87.6%**	**85.8%**	**82.9%**	**80.5%**	**78.3%**
**8-plex**	BC1 - Y	3,080,436,051	89,568	6,004,455	2,309,711	969,541	42%	14436.7	522.7		89.8%	86.2%	84.0%	82.7%	80.1%	77.7%	74.2%	70.8%	67.8%
	BC2 - Y	3,080,436,051	89,568	4,008,564	1,643,002	699,874	43%	14650.1	377.6		88.3%	83.6%	80.7%	79.2%	76.4%	73.6%	69.3%	65.9%	62.9%
	BC3 - C	3,080,436,051	89,568	4,114,328	1,548,483	572,638	37%	12718.4	308.8		85.5%	80.1%	77.3%	75.6%	72.2%	69.2%	64.6%	60.7%	57.6%
	BC4 - C	3,080,436,051	89,568	290,433	112,589	43,345	38%	13240.4	23.0		53.5%	35.9%	32.4%	30.6%	26.9%	24.3%	20.2%	16.9%	13.8%
	BC5 - Y	3,080,436,051	89,568	4,880,335	1,788,461	723,788	40%	13918.5	389.1		88.2%	83.6%	81.2%	79.6%	76.7%	74.2%	69.5%	65.8%	62.9%
	BC6 - Y	3,080,436,051	89,568	3,626,078	1,494,162	592,253	40%	13632.3	318.3		87.9%	82.4%	79.1%	77.5%	74.7%	71.8%	67.0%	63.2%	60.0%
	BC7 - C	3,080,436,051	89,568	3,052,954	1,207,766	475,387	39%	13537.0	255.0		85.3%	79.4%	76.2%	74.7%	71.1%	67.9%	63.1%	59.0%	55.7%
	BC8 - C	3,080,436,051	89,568	3,375,781	1,272,792	501,261	39%	13544.6	269.1		84.6%	78.5%	75.9%	74.4%	70.9%	67.7%	62.7%	58.9%	55.7%
	**ALL**	**3,080,436,051**	**89,568**	**29,352,928**	**11,376,966**	**4,578,087**	**40%**	**13839.4**	**308.0**	**2463.8**	**82.9%**	**76.2%**	**73.4%**	**71.8%**	**68.6%**	**65.8%**	**61.3%**	**57.6%**	**54.5%**
**16-plex**	BC1 - Y	3,080,436,051	89,568	4,899,027	1,763,942	687,831	39%	13410.9	370.9		89.0%	81.5%	78.4%	76.7%	72.9%	70.1%	65.7%	62.3%	59.3%
	BC2 - Y	3,080,436,051	89,568	2,676,886	1,012,073	386,136	38%	13121.6	208.2		83.0%	74.5%	70.9%	68.8%	64.6%	61.5%	56.6%	52.7%	49.7%
	BC3 - C	3,080,436,051	89,568	2,668,379	892,992	310,989	35%	11977.2	167.5		81.8%	71.0%	66.7%	64.6%	59.8%	56.3%	51.8%	47.9%	44.6%
	BC4 - C	3,080,436,051	89,568	192,251	69,348	25,240	36%	12517.4	13.4		46.2%	26.4%	22.2%	20.7%	17.2%	15.2%	11.8%	9.3%	7.5%
	BC5 - Y	3,080,436,051	89,568	3,195,035	1,105,138	426,109	39%	13260.6	229.5		83.8%	74.5%	70.1%	68.0%	64.0%	60.9%	56.1%	52.7%	49.3%
	BC6 - Y	3,080,436,051	89,568	2,137,151	808,725	295,560	37%	12569.1	159.1		81.8%	71.7%	67.8%	65.5%	61.3%	57.8%	52.3%	48.2%	44.4%
	BC7 - C	3,080,436,051	89,568	2,046,478	717,859	264,299	37%	12662.4	141.9		78.4%	67.6%	63.3%	61.1%	56.8%	53.4%	48.3%	43.8%	40.7%
	BC8 - C	3,080,436,051	89,568	2,009,518	717,838	267,807	37%	12830.8	143.9		78.8%	67.3%	62.7%	60.7%	56.1%	52.2%	47.1%	43.1%	39.4%
	BC9 - Y	3,080,436,051	89,568	1,880,236	682,333	244,284	36%	12312.8	131.1		76.1%	64.9%	60.9%	58.7%	53.9%	50.1%	45.9%	42.2%	39.0%
	BC10 - Y	3,080,436,051	89,568	2,779,455	898,160	338,070	38%	12945.3	181.4		79.4%	69.6%	66.0%	64.0%	59.8%	56.9%	51.9%	48.0%	44.7%
	BC11 - C	3,080,436,051	89,568	1,711,792	664,388	268,928	40%	13921.1	145.1		81.6%	71.8%	67.5%	65.3%	60.5%	57.3%	51.8%	47.3%	43.9%
	BC12 - C	3,080,436,051	89,568	1,943,943	769,503	307,849	40%	13759.0	167.0		81.2%	73.0%	69.0%	66.9%	62.9%	59.1%	53.8%	49.5%	45.9%
	BC13 - Y	3,080,436,051	89,568	2,389,711	863,430	344,892	40%	13737.7	185.7		81.9%	73.0%	69.1%	67.2%	63.1%	59.8%	54.2%	49.9%	46.7%
	BC14 - Y	3,080,436,051	89,568	1,979,651	767,513	295,798	39%	13254.7	159.6		80.5%	70.9%	67.2%	65.2%	60.8%	57.3%	52.3%	48.3%	44.9%
	BC19 - C	3,080,436,051	89,568	2,009,942	747,860	287,357	38%	13214.8	155.6		83.0%	73.3%	69.4%	67.3%	62.8%	59.6%	54.0%	49.6%	46.6%
	BC20 - C	3,080,436,051	89,568	2,678,166	1,054,017	405,609	38%	13234.9	219.4		89.5%	80.6%	76.6%	74.3%	70.0%	65.8%	60.8%	56.4%	53.0%
	**ALL**	**3,080,436,051**	**89,568**	**37,197,621**	**13,535,119**	**5,156,758**	**38%**	**13103.1**	**173.7**	**2779.3**	**79.7%**	**69.5%**	**65.5%**	**63.4%**	**59.2%**	**55.8%**	**50.9%**	**47.0%**	**43.7%**
**20-plex**	BC1 - Y	3,080,436,051	89,568	4,126,955	622,555	126,164	20%	6969.7	60.9		84.1%	70.3%	63.4%	59.6%	51.7%	46.4%	38.7%	33.3%	29.1%
	BC2 - Y	3,080,436,051	89,568	1,991,692	330,256	71,846	22%	7481.9	35.3		76.7%	59.7%	52.5%	48.4%	41.3%	36.0%	28.5%	23.2%	19.5%
	BC3 - C	3,080,436,051	89,568	1,561,461	244,059	46,667	19%	6576.2	22.6		71.6%	51.7%	44.0%	40.0%	32.8%	27.3%	20.2%	15.7%	12.5%
	BC4 - C	3,080,436,051	89,568	131,453	20,205	4,091	20%	6963.5	1.9		29.1%	10.1%	6.5%	5.5%	3.5%	2.4%	1.0%	0.5%	0.3%
	BC5 - Y	3,080,436,051	89,568	2,139,831	316,510	64,795	20%	7040.7	30.9		76.3%	57.1%	48.8%	44.7%	37.5%	32.6%	26.0%	20.7%	17.2%
	BC6 - Y	3,080,436,051	89,568	1,508,733	255,951	48,416	19%	6505.7	23.3		74.1%	52.5%	43.7%	39.7%	31.8%	27.0%	20.6%	16.2%	13.2%
	BC7 - C	3,080,436,051	89,568	1,292,026	204,759	42,334	21%	7110.6	20.2		70.0%	48.2%	40.4%	37.0%	30.1%	25.2%	18.3%	13.9%	10.7%
	BC8 - C	3,080,436,051	89,568	1,268,419	205,238	41,203	20%	6904.5	19.7		67.3%	47.5%	39.9%	36.1%	28.9%	24.3%	18.2%	14.0%	11.0%
	BC9 - Y	3,080,436,051	89,568	1,447,438	233,302	42,123	18%	6209.6	19.7		70.0%	48.0%	39.3%	35.6%	27.9%	23.5%	17.4%	13.4%	10.7%
	BC10 - Y	3,080,436,051	89,568	1,879,568	291,577	52,879	18%	6237.2	24.8		72.0%	52.0%	43.6%	39.6%	33.0%	28.2%	21.5%	17.2%	14.2%
	BC11 - C	3,080,436,051	89,568	1,130,415	195,219	42,703	22%	7523.1	21.0		71.8%	50.7%	42.3%	38.9%	32.2%	26.9%	19.9%	14.9%	11.6%
	BC12 - C	3,080,436,051	89,568	1,423,950	229,965	51,665	22%	7726.7	25.7		75.4%	56.0%	47.2%	43.2%	36.2%	30.8%	23.0%	17.8%	14.1%
	BC13 - Y	3,080,436,051	89,568	1,619,611	271,932	54,721	20%	6920.7	26.5		73.7%	53.3%	45.8%	42.0%	34.8%	29.6%	22.6%	18.3%	15.5%
	BC14 - Y	3,080,436,051	89,568	1,374,871	235,356	49,981	21%	7303.6	24.3		71.8%	53.5%	45.4%	41.5%	34.7%	29.1%	21.3%	16.5%	13.1%
	BC15 - C	3,080,436,051	89,568	2,021,309	311,022	61,561	20%	6807.3	29.8		75.6%	56.6%	48.6%	44.9%	37.9%	32.5%	24.7%	19.9%	16.4%
	BC16 - C	3,080,436,051	89,568	2,211,808	349,119	72,408	21%	7133.0	35.0		78.1%	59.3%	51.3%	47.5%	40.4%	35.1%	27.5%	22.4%	18.5%
	BC17 - Y	3,080,436,051	89,568	1,911,811	295,946	58,331	20%	6778.7	28.0		75.1%	55.1%	47.1%	42.9%	35.9%	30.6%	23.7%	19.0%	15.7%
	BC18 - Y	3,080,436,051	89,568	1,594,490	270,969	52,054	19%	6606.8	25.0		74.5%	53.8%	45.8%	41.1%	33.4%	28.6%	22.2%	17.7%	13.9%
	BC19 - C	3,080,436,051	89,568	1,413,120	231,732	45,876	20%	6808.6	22.8		75.1%	53.9%	46.5%	42.4%	34.8%	29.3%	21.8%	16.3%	12.4%
	BC20 - C	3,080,436,051	89,568	1,826,532	335,267	63,283	19%	6491.7	31.1		80.9%	60.3%	52.6%	48.0%	39.5%	34.0%	26.7%	21.7%	18.0%
	**ALL**	**3,080,436,051**	**89,568**	**33,875,493**	**5,450,939**	**1,093,101**	**20%**	**6896.8**	**26.4**	**528.4**	**72.2%**	**52.5%**	**44.7%**	**40.9%**	**33.9%**	**29.0%**	**22.2%**	**17.6%**	**14.4%**
**No BC - Y**	**-**	**3,080,436,051**	**89,568**	**33,216,839**	**13,976,509**	**5,583,805**	**40%**	**13740.1**		**2909.0**	**99.1%**	**97.9%**	**97.3%**	**97.0%**	**96.3%**	**95.7%**	**94.7%**	**93.9%**	**93.0%**
**No BC - C**	**-**	**3,080,436,051**	**89,568**	**37,890,578**	**15,955,304**	**2,121,891**	**13%**	**4573.8**		**977.9**	**98.9%**	**97.2%**	**96.2%**	**95.8%**	**94.8%**	**93.7%**	**91.5%**	**89.4%**	**87.7%**
**No BC - Y & C**	**Average**	**3,080,436,051**	**89,568**	**35,553,708**	**14,965,906**	**3,852,848**	**26%**	**8854.0**		**1888.1**	**99.7%**	**97.5%**	**96.8%**	**96.4%**	**95.5%**	**94.7%**	**93.1%**	**91.7%**	**90.4%**

* herein identical to reads obtained off the SOLiD3 machine, i.e. without QualityValue-filtering (this filtering is carried out during tertiary analyses, e.g. mapping/pairing/SNP-calling)

This table shows in detail the effect of SAET2.2 (SOLiD Software Spectral Analysis Enhancement Tool) on read mapping with corona lite. Comparison with Table S23 (no SAET 2.2) shows that SAET2.2 improved coverages by 16%-21%.

Table S25: On-target reads (uniquely mapped) for different mapping strategies

HapMap YRI NA18507 (Y) & CHB NA18561 (C)	Number of barcodes	SAET + Bioscope	Bioscope	SAET + corona lite (5MM)	corona lite (5MM)	corona lite (4MM)	corona lite (4MM)	corona lite (5MM)	SAET 2.2 + corona lite (5MM)	BioScope 1.01	SAET 2.2 + BioScope 1.01
4-plex 1	4	3,591,810	3,019,562	2,859,198	2,437,641	2,245,534	1.00	1.09	1.27	1.34	1.60
4-plex 2	4	7,077,969	5,989,428	5,857,843	4,795,435	4,366,642	1.00	1.10	1.34	1.37	1.62
8-plex	8	5,543,898	4,767,966	4,578,087	3,884,341	3,582,298	1.00	1.08	1.28	1.33	1.55
16-plex	16	6,296,338	5,317,746	5,156,758	4,282,527	3,927,326	1.00	1.09	1.31	1.35	1.60
20-plex	20	1,717,165	1,392,083	1,093,101	913,163	804,301	1.00	1.14	1.36	1.73	2.13
No BC - Y	-	6,232,487	5,366,668	5,583,805	4,465,562	4,132,386	1.00	1.08	1.35	1.30	1.51
No BC - C	-	2,554,210	2,100,645	2,121,891	1,603,486	1,468,434	1.00	1.09	1.45	1.43	1.74
**no BC - averaged**	**-**	**4,393,348**	**3,733,656**	**3,852,848**	**3,034,524**	**2,800,410**	1.00	1.08	1.38	1.33	1.57

	Barcode	SAET + Bioscope	Bioscope	SAET + corona lite (5MM)	corona lite (5MM)	corona lite (4MM)	corona lite (4MM)	corona lite (5MM)	SAET + corona lite (5MM)	Bioscope	SAET + Bioscope
**4-plex 1**	BC5 - Y	1,138,964	947,117	898,412	761,443	699,918	1.00	1.09	1.28	1.35	1.63
	BC6 - Y	872,393	740,350	702,989	602,317	556,109	1.00	1.08	1.26	1.33	1.57
	BC7 - C	760,764	643,895	608,673	521,616	481,407	1.00	1.08	1.26	1.34	1.58
	BC8 - C	819,689	688,200	649,124	552,265	508,100	1.00	1.09	1.28	1.35	1.61
	**ALL**	**3,591,810**	**3,019,562**	**2,859,198**	**2,437,641**	**2,245,534**	1.00	1.09	1.27	1.34	1.60
**4-plex 2**	7	1,705,759	1,452,489	1,421,235	1,169,339	1,065,538	1.00	1.10	1.33	1.36	1.60
	8	1,791,048	1,517,769	1,480,302	1,212,822	1,104,445	1.00	1.10	1.34	1.37	1.62
	9	1,586,804	1,334,403	1,305,502	1,064,335	968,303	1.00	1.10	1.35	1.38	1.64
	10	1,994,358	1,684,767	1,650,804	1,348,939	1,228,356	1.00	1.10	1.34	1.37	1.62
	**ALL**	**7,077,969**	**5,989,428**	**5,857,843**	**4,795,435**	**4,366,642**	1.00	1.10	1.34	1.37	1.62
**8-plex**	1	1,170,691	1,006,934	969,541	821,291	757,054	1.00	1.08	1.28	1.33	1.55
	2	834,081	728,961	699,874	600,633	555,855	1.00	1.08	1.26	1.31	1.50
	3	696,228	591,613	572,638	481,631	443,259	1.00	1.09	1.29	1.33	1.57
	4	52,846	46,459	43,345	37,401	34,529	1.00	1.08	1.26	1.35	1.53
	5	888,915	754,595	723,788	608,042	558,842	1.00	1.09	1.30	1.35	1.59
	6	710,705	617,497	592,253	507,030	468,767	1.00	1.08	1.26	1.32	1.52
	7	576,042	496,883	475,387	404,250	373,103	1.00	1.08	1.27	1.33	1.54
	8	614,390	525,024	501,261	424,063	390,889	1.00	1.08	1.28	1.34	1.57
	**ALL**	**5,543,898**	**4,767,966**	**4,578,087**	**3,884,341**	**3,582,298**	1.00	1.08	1.28	1.33	1.55
**16-plex**	1	840,229	704,735	687,831	568,303	520,213	1.00	1.09	1.32	1.35	1.62
	2	469,776	319,781	386,136	321,712	294,618	1.00	1.09	1.31	1.09	1.59
	3	389,808	414,431	310,989	253,695	231,767	1.00	1.09	1.34	1.79	1.68
	4	30,890	26,309	25,240	21,245	19,534	1.00	1.09	1.29	1.35	1.58
	5	526,516	437,081	426,109	348,896	318,951	1.00	1.09	1.34	1.37	1.65
	6	359,581	304,857	295,560	247,362	227,005	1.00	1.09	1.30	1.34	1.58
	7	327,672	273,054	264,299	218,117	199,642	1.00	1.09	1.32	1.37	1.64
	8	329,037	275,214	267,807	221,620	203,200	1.00	1.09	1.32	1.35	1.62
	9	302,505	252,875	244,284	201,609	184,861	1.00	1.09	1.32	1.37	1.64
	10	422,948	347,474	338,070	276,277	252,092	1.00	1.10	1.34	1.38	1.68
	11	322,884	276,904	268,928	226,251	208,097	1.00	1.09	1.29	1.33	1.55
	12	368,324	315,790	307,849	257,387	236,475	1.00	1.09	1.30	1.34	1.56
	13	420,863	353,981	344,892	287,078	263,362	1.00	1.09	1.31	1.34	1.60
	14	355,431	304,351	295,798	248,869	229,240	1.00	1.09	1.29	1.33	1.55
	19	346,545	294,743	287,357	240,866	221,572	1.00	1.09	1.30	1.33	1.56
	20	483,329	416,166	405,609	343,240	316,697	1.00	1.08	1.28	1.31	1.53
	**ALL**	**6,296,338**	**5,317,746**	**5,156,758**	**4,282,527**	**3,927,326**	1.00	1.09	1.31	1.35	1.60
**20-plex**	1	202,306	161,242	126,164	104,443	91,534	1.00	1.14	1.38	1.76	2.21
	2	108,983	89,554	71,846	60,206	53,569	1.00	1.12	1.34	1.67	2.03
	3	74,202	59,598	46,667	38,838	34,224	1.00	1.13	1.36	1.74	2.17
	4	6,728	5,577	4,091	3,535	3,001	1.00	1.18	1.36	1.86	2.24
	5	105,030	83,509	64,795	53,289	46,581	1.00	1.14	1.39	1.79	2.25
	6	75,966	62,242	48,416	40,744	36,045	1.00	1.13	1.34	1.73	2.11
	7	67,284	54,153	42,334	34,862	30,496	1.00	1.14	1.39	1.78	2.21
	8	65,679	52,853	41,203	34,397	30,119	1.00	1.14	1.37	1.75	2.18
	9	69,109	55,329	42,123	34,848	30,208	1.00	1.15	1.39	1.83	2.29
	10	88,136	69,585	52,879	43,554	38,029	1.00	1.15	1.39	1.83	2.32
	11	63,760	53,150	42,703	36,319	32,458	1.00	1.12	1.32	1.64	1.96
	12	77,141	63,353	51,665	43,307	38,397	1.00	1.13	1.35	1.65	2.01
	13	85,387	70,265	54,721	46,065	40,496	1.00	1.14	1.35	1.74	2.11
	14	76,077	63,007	49,981	42,278	37,243	1.00	1.14	1.34	1.69	2.04
	15	97,836	78,509	61,561	51,092	44,873	1.00	1.14	1.37	1.75	2.18
	16	113,270	91,250	72,408	60,078	52,810	1.00	1.14	1.37	1.73	2.14
	17	94,006	75,071	58,331	48,375	42,534	1.00	1.14	1.37	1.76	2.21
	18	81,370	66,459	52,054	43,711	38,602	1.00	1.13	1.35	1.72	2.11
	19	69,821	57,308	45,876	38,917	34,598	1.00	1.12	1.33	1.66	2.02
	20	95,074	80,069	63,283	54,305	48,484	1.00	1.12	1.31	1.65	1.96
	**ALL**	**1,717,165**	**1,392,083**	**1,093,101**	**913,163**	**804,301**	1.00	1.14	1.36	1.73	2.13
**No BC - Y**	**-**	**6,232,487**	**5,366,668**	**5,583,805**	**4,465,562**	**4,132,386**	1.00	1.08	1.35	1.30	1.51
**No BC - C**	**-**	**2,554,210**	**2,100,645**	**2,121,891**	**1,603,486**	**1,468,434**	1.00	1.09	1.45	1.43	1.74
**No BC - Y & C**	**Average**	**4,393,348**	**3,733,656**	**3,852,848**	**3,034,524**	**2,800,410**	1.00	1.08	1.38	1.33	1.57

This table compares SOLiD software mapping tools (corona lite 4.0, BioScope 1.0.1) with and without prior SAET 2.2 read enhancement. For corona lite the default settings (four color space mismatches with two valid adjacents counting as one mismatch) and the least stringent setting (five color space mismatches with two valid adjacents counting as one mismatch) are included. From corona lite with default settings to SAET + Bioscope we obtained an improvement of about 60% in the number of mapped reads. See manuscript Figure 2 for a graphical plot of the comparison.

Table S26: BRCA1/2 target regions (1-based hg18 / NCBI36 coordinates)

chr17:38449800-38450359	chr17:38502678-38502937	chr13:31799804-31799925
chr17:38450622-38451487	chr17:38502948-38503039	chr13:31800134-31800393
chr17:38452296-38452483	chr17:38503122-38503177	chr13:31800614-31800693
chr17:38452920-38453083	chr17:38503368-38503597	chr13:31800734-31800807
chr17:38453118-38453311	chr17:38503836-38503903	chr13:31800920-31801863
chr17:38454336-38455891	chr17:38504328-38504383	chr13:31802978-31803243
chr17:38456154-38457205	chr17:38504640-38504827	chr13:31803392-31803591
chr17:38457456-38457703	chr17:38505090-38505613	chr13:31803698-31803957
chr17:38458032-38458441	chr17:38505696-38505901	chr13:31803998-31805775
chr17:38458662-38458945	chr17:38506224-38506303	chr13:31806356-31806561
chr17:38459202-38459257	chr17:38506554-38506759	chr13:31806812-31807221
chr17:38459508-38460007	chr17:38507016-38507071	chr13:31807478-31808037
chr17:38460570-38460931	chr17:38507322-38507473	chr13:31808294-31813785
chr17:38462214-38462281	chr17:38508588-38508751	chr13:31814474-31814649
chr17:38462490-38462791	chr17:38509008-38509075	chr13:31815266-31815723
chr17:38463564-38463643	chr17:38509140-38509387	chr13:31815968-31816095
chr17:38463900-38464663	chr17:38509608-38510095	chr13:31816154-31816869
chr17:38465286-38465461	chr17:38510220-38510803	chr13:31817588-31817745
chr17:38466006-38466067	chr17:38511060-38511259	chr13:31818038-31818099
chr17:38466330-38466409	chr17:38511348-38511427	chr13:31818560-31819191
chr17:38467032-38467153	chr17:38511666-38512465	chr13:31821032-31821099
chr17:38467404-38467609	chr17:38513334-38513629	chr13:31822184-31822251
chr17:38467914-38468167	chr17:38513868-38513995	chr13:31823834-31823955
chr17:38468706-38470393	chr17:38514540-38514595	chr13:31824218-31825203
chr17:38470650-38470951	chr17:38514816-38515465	chr13:31825838-31825965
chr17:38471214-38471299	chr17:38515716-38515867	chr13:31826720-31827741
chr17:38471736-38471977	chr17:38516124-38516257	chr13:31828010-31828209
chr17:38472228-38472379	chr17:38516640-38516803	chr13:31828484-31829199
chr17:38472636-38472805	chr17:38517390-38517661	chr13:31829402-31830351
chr17:38473074-38473327	chr17:38517906-38518003	chr13:31830614-31830669
chr17:38473416-38473561	chr17:38518212-38518363	chr13:31831364-31831989
chr17:38473812-38473993	chr17:38518626-38518927	chr13:31833980-31834179
chr17:38474088-38475499	chr17:38519190-38519365	chr13:31834442-31836399
chr17:38475756-38476879	chr17:38519754-38519953	chr13:31837190-31837269
chr17:38477136-38477287	chr17:38519982-38520787	chr13:31837418-31837527
chr17:38477568-38477671	chr17:38521038-38521471	chr13:31837790-31837851
chr17:38477916-38477977	chr17:38521512-38521789	chr13:31838144-31838547
chr17:38478522-38478613	chr17:38521890-38521945	chr13:31840088-31840161
chr17:38478690-38479303	chr17:38522034-38522089	chr13:31840424-31840491
chr17:38479578-38480335	chr17:38522616-38522725	chr13:31840712-31840923
chr17:38480634-38481127	chr17:38523288-38523427	chr13:31841222-31841415
chr17:38481246-38481409	chr17:38523888-38523943	chr13:31841540-31841631
chr17:38481648-38482897	chr17:38524260-38524327	chr13:31842068-31842129
chr17:38483154-38483287	chr17:38524572-38524753	chr13:31842194-31842771
chr17:38483994-38484481	chr17:38524806-38524939	chr13:31843010-31843875
chr17:38484726-38485759	chr17:38525022-38526049	chr13:31848254-31848399
chr17:38485770-38485879	chr17:38526588-38526661	chr13:31848656-31849467
chr17:38486016-38486089	chr17:38527236-38527903	chr13:31849724-31850343
chr17:38486436-38488723	chr17:38528754-38528917	chr13:31850468-31850997
chr17:38488974-38489083	chr17:38529168-38530315	chr13:31851164-31852545
chr17:38489106-38489425	chr17:38530512-38532199	chr13:31853096-31853325
chr17:38489988-38490823	chr13:31787000-31788297	chr13:31856570-31858329
chr17:38490906-38491663	chr13:31788404-31788957	chr13:31858538-31858839
chr17:38491668-38492587	chr13:31789616-31789695	chr13:31858874-31859001
chr17:38493210-38493307	chr13:31790390-31790601	chr13:31860782-31860903
chr17:38493570-38493715	chr13:31790852-31791495	chr13:31861838-31862307
chr17:38494062-38494597	chr13:31791644-31791981	chr13:31862348-31862469
chr17:38494854-38495113	chr13:31792238-31792419	chr13:31862546-31863021
chr17:38495196-38495293	chr13:31792748-31793001	chr13:31863164-31863381
chr17:38495550-38495761	chr13:31793204-31793379	chr13:31863686-31864563
chr17:38495796-38495935	chr13:31793444-31794129	chr13:31866602-31867593
chr17:38496168-38500867	chr13:31795568-31796199	chr13:31867766-31868097
chr17:38501130-38501659	chr13:31796540-31796787	chr13:31868384-31868529
chr17:38501928-38501983	chr13:31797020-31797135	chr13:31868720-31869597
chr17:38502114-38502457	chr13:31797152-31797699	chr13:31869932-31871175
chr17:38502480-38502541	chr13:31797956-31799325	chr13:31871270-31871895
