# Supplementary material for: Improving mapping and SNP-calling performance in multiplexed targeted next-generation sequencing
Source: BMC Genomics. 2012 Aug 22;13:417. doi: 10.1186/1471-2164-13-417 (PMC3563481; doi:10.1186/1471-2164-13-417)
Supplement: Additional file 3 — Supporting Figures. Figures S1-S9. [file 1471-2164-13-417-S3.pdf]

Supplementary Figures S1-S9

Improving mapping and SNP-calling performance in multiplexed targeted next-generation sequencing

Abdou ElSharawy[1,5], Michael Forster[1, 5], Nadine Schracke[2], Andreas Keller [3], Ingo Thomsen[1], Britt-Sabina Petersen[1], Björn Stade[1], Peer Stähler[2], Stefan Schreiber[1,4], Philip Rosenstiel[1] and Andre Franke[1]

1 Institute of Clinical Molecular Biology, Christian-Albrechts-University, Kiel, Germany
2 Febit biomed GmbH, Heidelberg, Germany.
3 Biomarker Discovery Center, Heidelberg, Germany
4 Department of General Internal Medicine, Campus Kiel, University Hospital S.-H., Germany
5 These authors contributed equally to this work

Email addresses
AE: a.sharawy@mucosa.de
MF: m.forster@ikmb.uni-kiel.de
NS: nadine@schracke.de
AK: keller.andreas@siemens.com
BS: b.stade@ikmb.uni-kiel.de
B-SP: b.petersen@ikmb.uni-kiel.de
IT: i.thomsen@ikmb.uni-kiel.de
PFS: Peer.staehler@t-online.de
SS: s.schreiber@mucosa.de
PR: p.rosenstiel@mucosa.de
AF: a.franke@mucosa.de

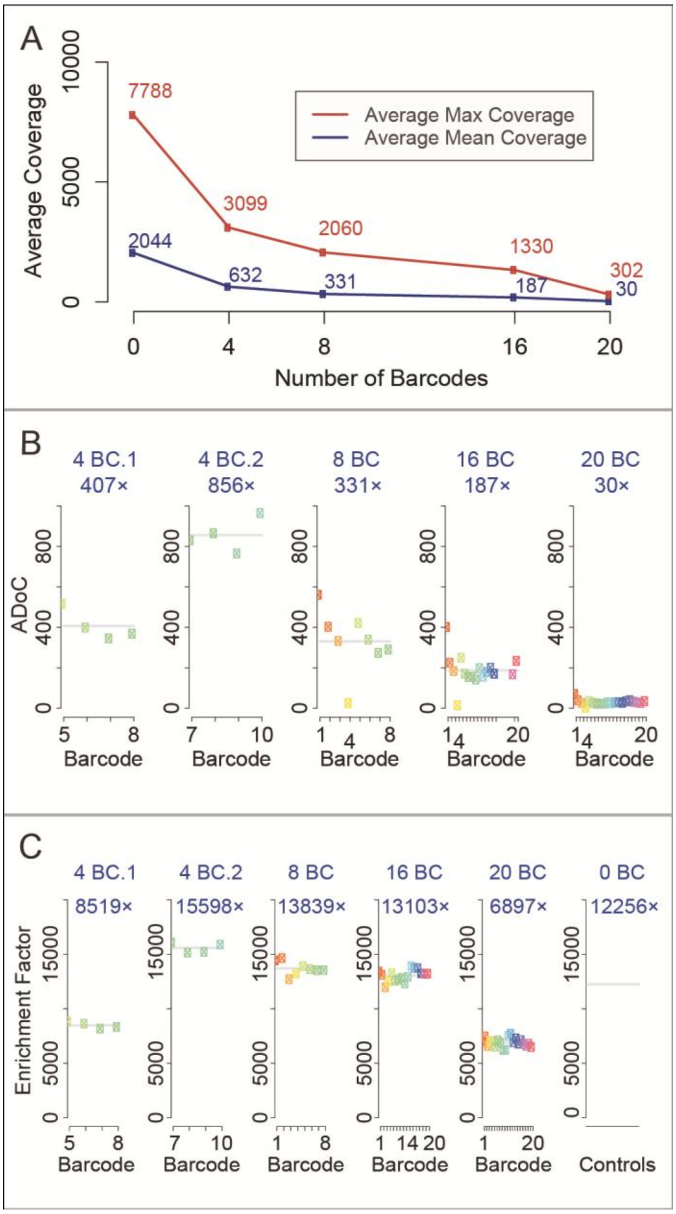

Figure S1. Coverage distribution across all the barcoded human samples for different multiplexing levels at the *BRCA1/2* genes (third stage of our *t*NGS test model).

This figure summarizes the coverages generated in the second stage of our experiments, i.e. barcoded human gDNA libraries enriched for the *BRCA1* and *BRCA2* cancer genes. Panel A: Average maximal and minimal coverage depth showing a nearly linear relationship between coverage and number of barcodes (4-plex1 and 4-plex2 are averaged into the same 4-plex). Panel B: ADoC for each barcode in each plex shows reasonable uniformity of coverage between barcodes within a plex, except for the low number of reads assigned to barcode 4 (yellow point) which we already observed in the *E.coli* experiments. Panel C: Enrichment folds for each barcode in each plex and for the non-barcoded controls. The enrichment factor (EF) was independent of the absolute number of reads and should ideally be identical for all barcodes and all multiplexes. The absolute enrichment factor was very high (the mean EF is 11781). Compared to the non-barcoded control, the 20-plex pool and one 4-plex pool showed under-enrichment. However, within each pool, the reproducibility of the enrichment step was very good.

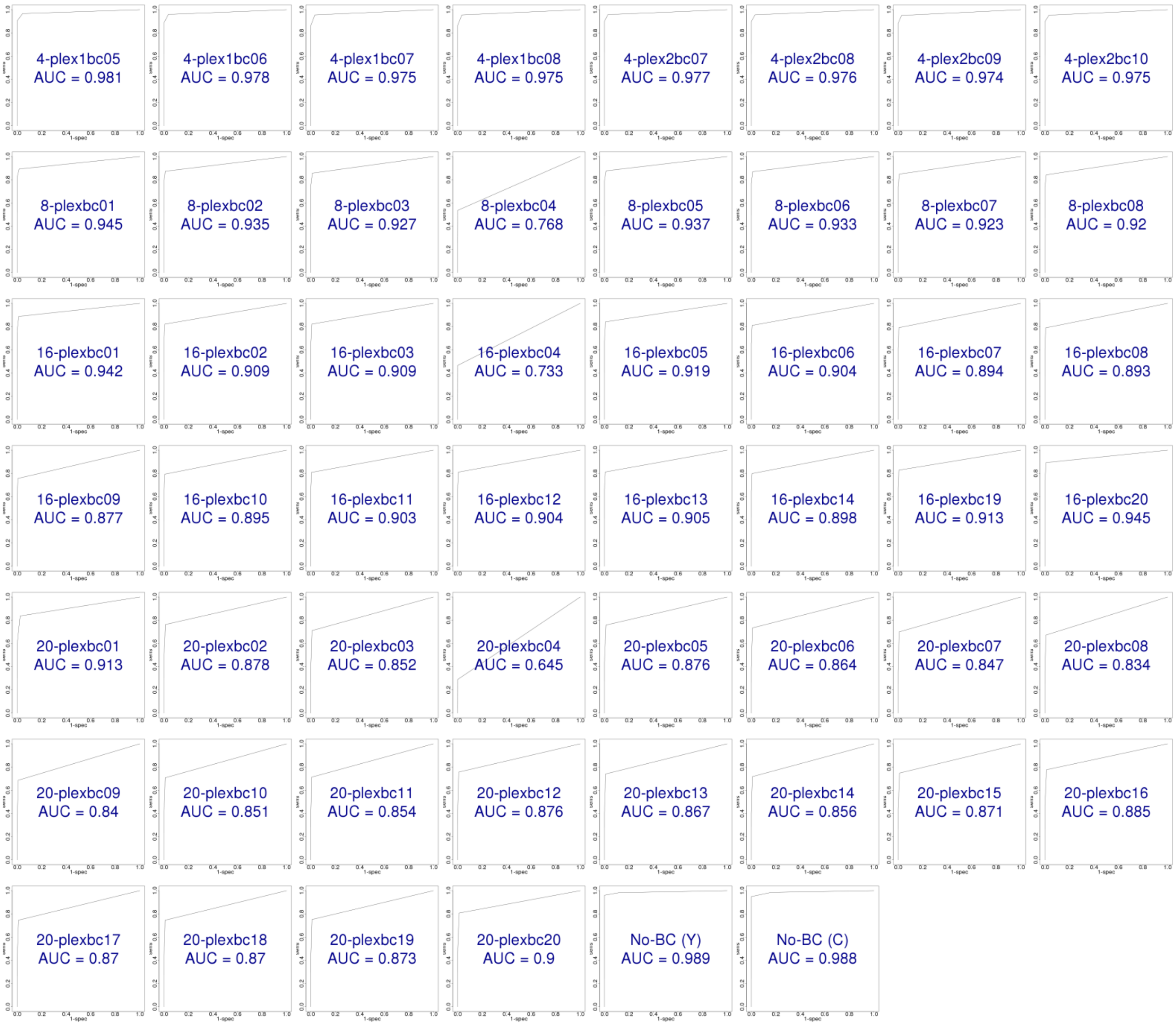

Figure S2: *BRCA1/2* target region ROC curves and AUC values for whole genome coverages obtained using SAET 2.2 read enhancement and Bioscope 1.0.1 read mapping. The AUC-values for all samples except those with barcode 4 are near 1, indicating very good target region enrichment. As we expected, the barcode 4 samples show poor enrichment with AUC-values near 0.5.

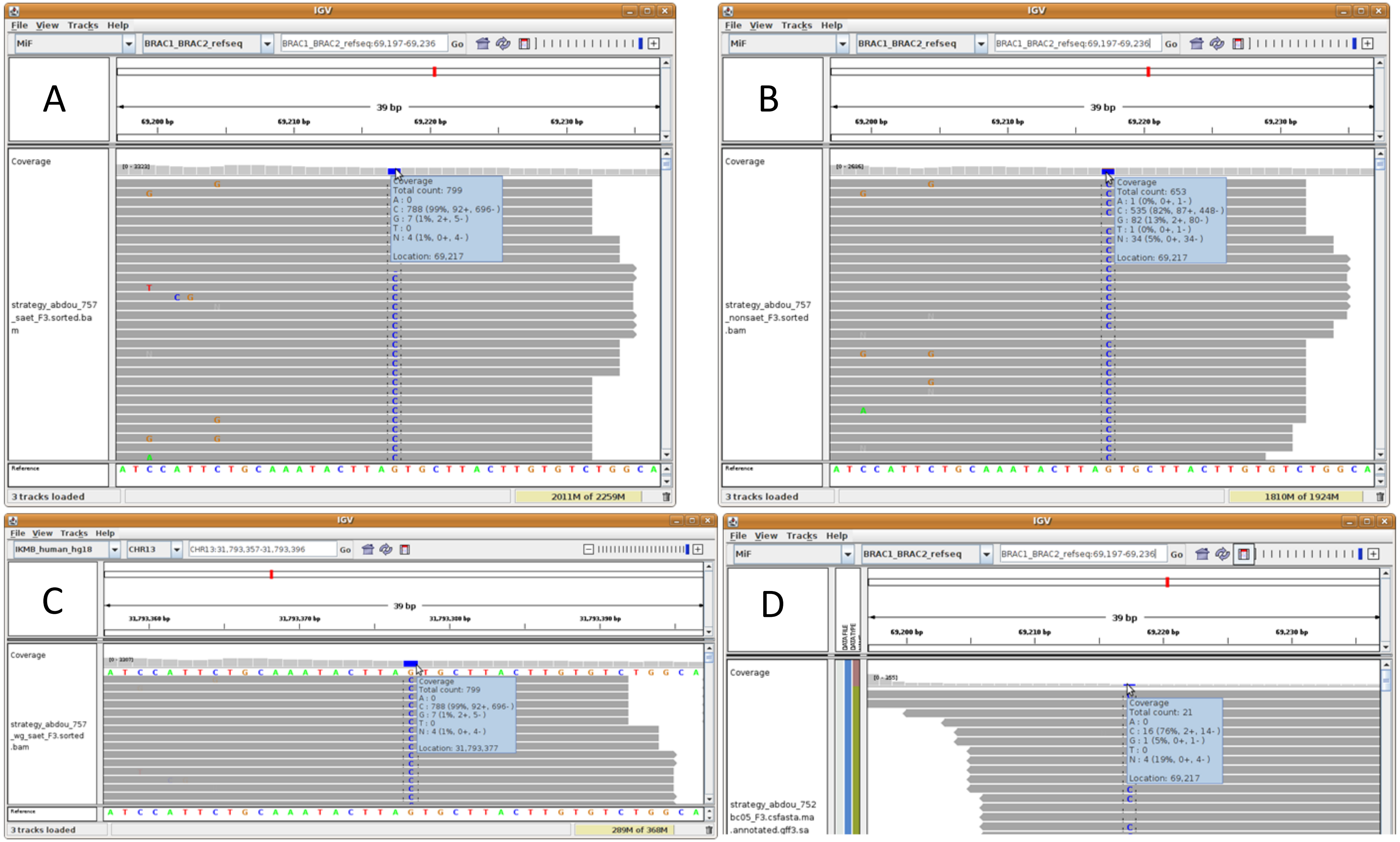

Figure S3: Manual inspection in IGV uncovered SNPs which the Bioscope/diBayes software did not call. Before manually inspecting the mapping results, the Bioscope 1.0.1 gff3-files needed to be converted to sam-format using Bioscope, and into indexed, sorted bam-format using samtools (Bioscope 1.2 now optionally generates sorted bam-files). We generated a list of expected SNPs and manually inspected these positions for target region mapping with SAET (**Panel A**) and without SAET (**Panel B**), and the corresponding position for whole genome mapping with SAET (**Panel C**). The mouse pop-up window - with coverages on each strand for each allele - appears when the mouse tip hovers over a coverage track position. Astonishingly it displayed absurdly high non-reference allele coverage at most known SNP positions which the Bioscope-SNP-caller filtered away, in fact no noticeable difference to the detected SNPs (e.g. top left and bottom left 799 reads indicate “A”, top right 653 reads indicate “A”). The lowest coverage at a known non-reference SNP, which was filtered away by Bioscope, was 21 reads (**Panel D:** 4 Ns, 1G, 16Cs). More IGV results are tabled in **Table S8**. We conclude that SNP-calling for enriched samples must take into account the underlying processes of a specific enrichment method so that high coverages are not misinterpreted as artifacts. From our manual inspections we concluded that the sequencing errors were in the region of 1% of bases within a read, and that coverages would be a valid method for scoring genotypes for the enrichment method presented here.

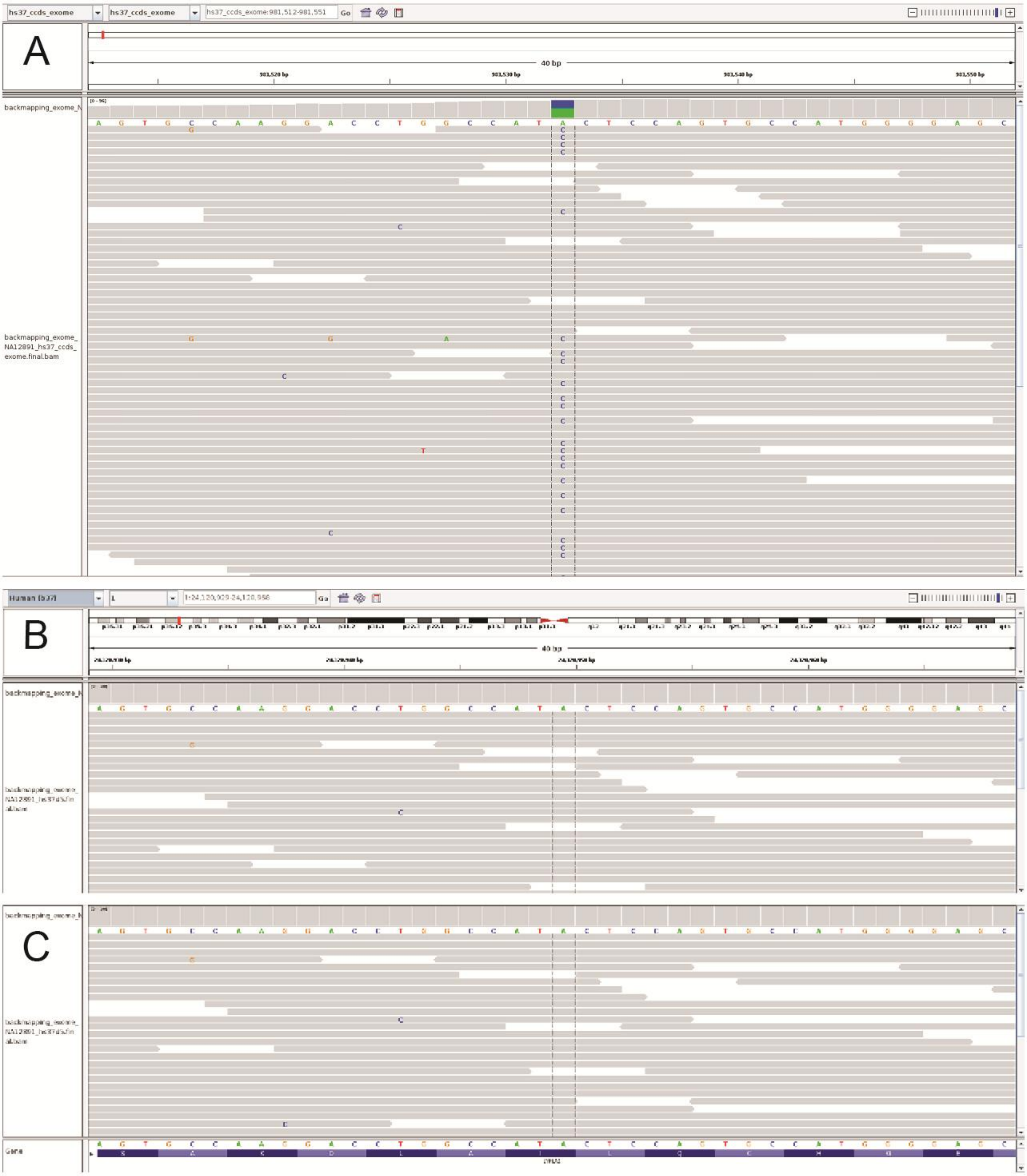

Figure S4. Cleaning up SNP-artifacts from target-region mapping by employing read-backmapping to the whole genome (fourth stage, exome data).

This figure shows the mapping results for publicly available Illumina HiSeq2000 human exome sequencing reads mapped to a target-region reference (**Panel A**), and to a genomic reference (**Panels B and C**). **Panel A** shows a treacherously convincing heterozygous SNP detected from the read-alignments to the exome target region. **Panel B** shows the same coordinate range without any trace of the SNP, when the reads are mapped to the whole genome. **Panel C** shows the same result as panel B. However, for Panel C, the reads covering the SNP in panel A were extracted from the target-region-mapping BAM-file, and in a second step only these extracted reads (and their corresponding paired-end mates) were aligned to the whole genome. Computation time for this exome was shortened by ~12 hours, i.e. more than halved compared to the conventional approach of mapping only to the whole genome.

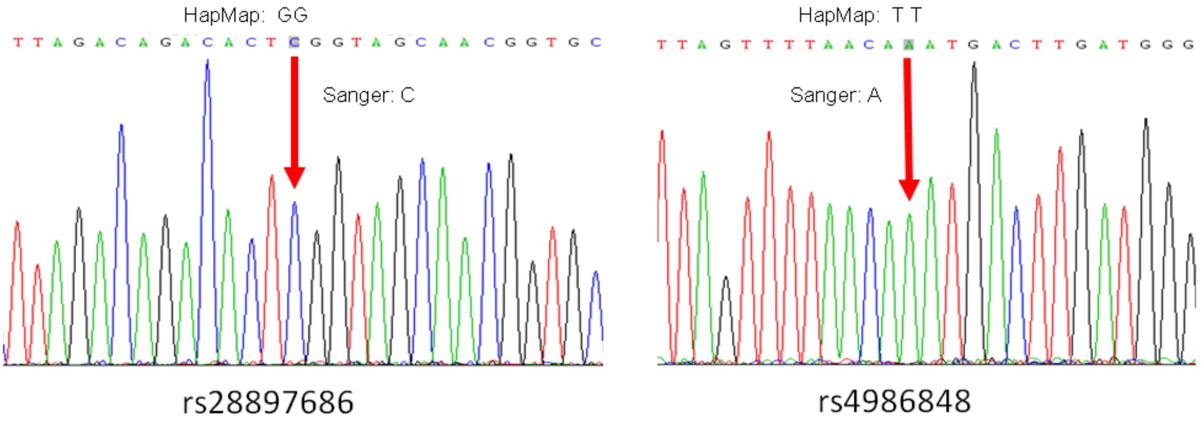

Sample	SNP	Pos.	Ref	Sanger	HapMap	IGV results						
						∑ reads	Consensus	∑ A	∑ C	∑ G	∑ T	∑ N
control (whole genome mapping of SAET reads)	rs28897686	chr17:38497326	C	C	GG	5369	C	0	5364	0	1	3
	rs4986848	chr17:38498108	A	A	TT	3825	A	3823	0	0	0	2
	rs28897673	chr17:38509792	T	T	AA	2138	T	1	1	1	2132	3
	rs28897731	chr13:31811420	T	-	AA	2165	T	0	0	1	2162	2
	rs206123	chr13:31793377	C	C	CC	799	C	0	788	7	0	4

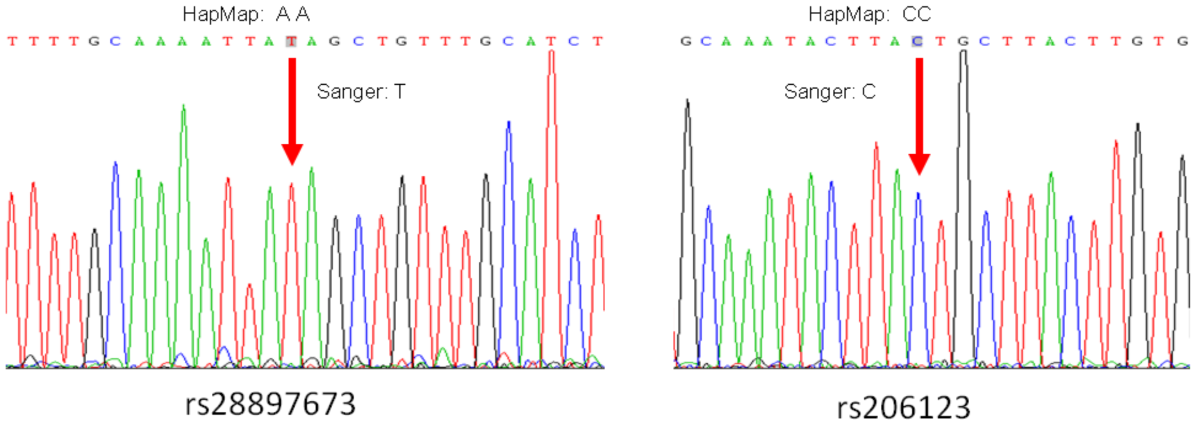

Figure S5: Sanger traces for 4 resequenced SNPs.

The HapMap3 data erroneously list the first three genotypes as the <u>reverse complement</u>. We first noticed this worrying type of HapMap3 error in the current NGS project, and also since then in new NGS projects. In contrast to the HapMap data, the Sanger results gave us high confidence in the NGS SNPs determined from the BAM files using IGV. It seems plausible that the HapMap researchers might have mixed up a strand flag and reported the complementary base instead of the correct base, and/or it was a bug in the SNP-chip platforms used by the HapMap project. This showed us that SOLiD NGS is a consistent method that we would prefer over genotyping platforms.

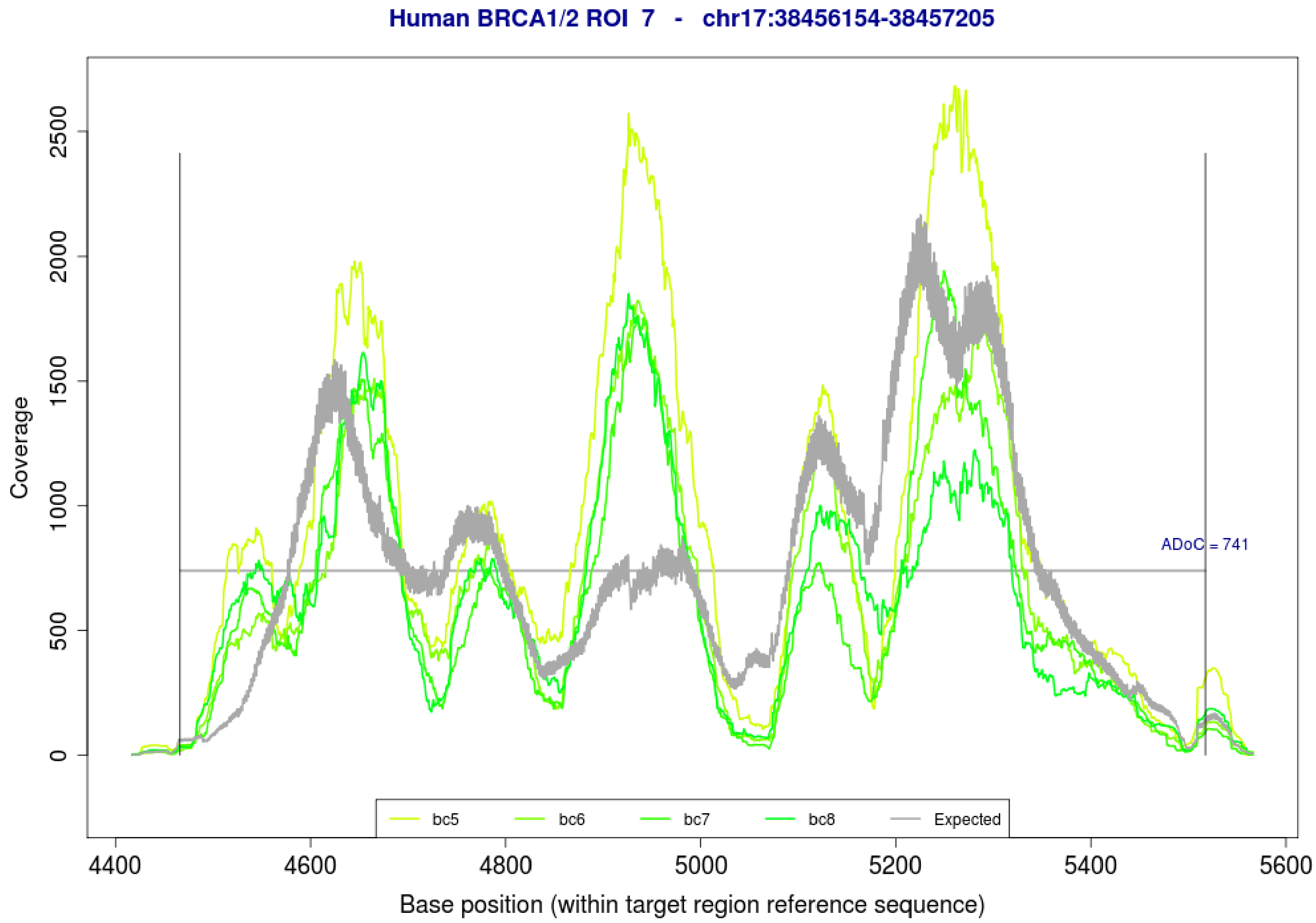

Figure S6: Multi-sample coverage plots give valuable insights into mapping uniformity and regions of high and low mapping.

This figure shows one example target region of about 1000 bp length. The vertical lines denote the limit of the target region. The coverage curves denote the extended target region (49 bases before and after the target region) which served to capture the 50 bp fragments at the end of a target region. In general, we observed high reproducibility between samples, as exemplified here in the similarity of the 4 coverage curves for barcodes 5-8 and the mean of the control samples' coverage curves. The figure clearly shows that, even with enrichment, some regions are not as highly covered as other regions. This is incidently also seen in non-enriched samples. Low sequence complexity (i.e. large scale repeats) was excluded and therefore cannot account for this phenomenon

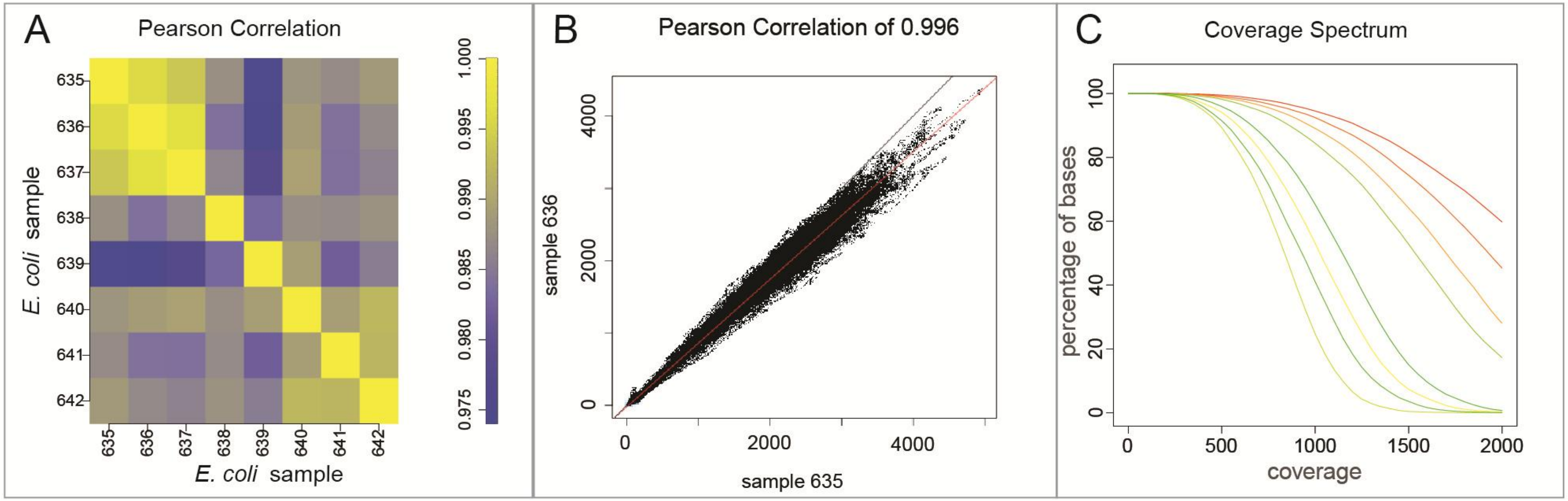

Figure S7. Sample correlation, reproducibility, and consensus coverage for the tested eight technical replicates (first stage of our *t*NGS test model).

This figure shows the results of the coverage-based analysis of the enrichment reproducibility for the first stage of our experiments, i.e. *E. coli* libraries enriched for 68 genes. **Panel A**: Heatmap of Pearson correlation values. The overall reproducibility within the eight experiments was very high, as demonstrated by Pearson correlation values of between 0.974 and 0.997. The highest coverage reproducibility is displayed for three of the four libraries prepared with the 'standard' fragment protocol. **Panel B**: Scatter plot comparing the coverages in two samples at each position in the target region. The point cloud is banded and nearly symmetrical to the bisector (gray line), indicating good reproducibility. **Panel C**: Coverage spectrum plot showing, for each sample, the percentage of target region covered by at least x reads. The highest coverages resulted for the four libraries prepared with the 'standard' fragment protocol.

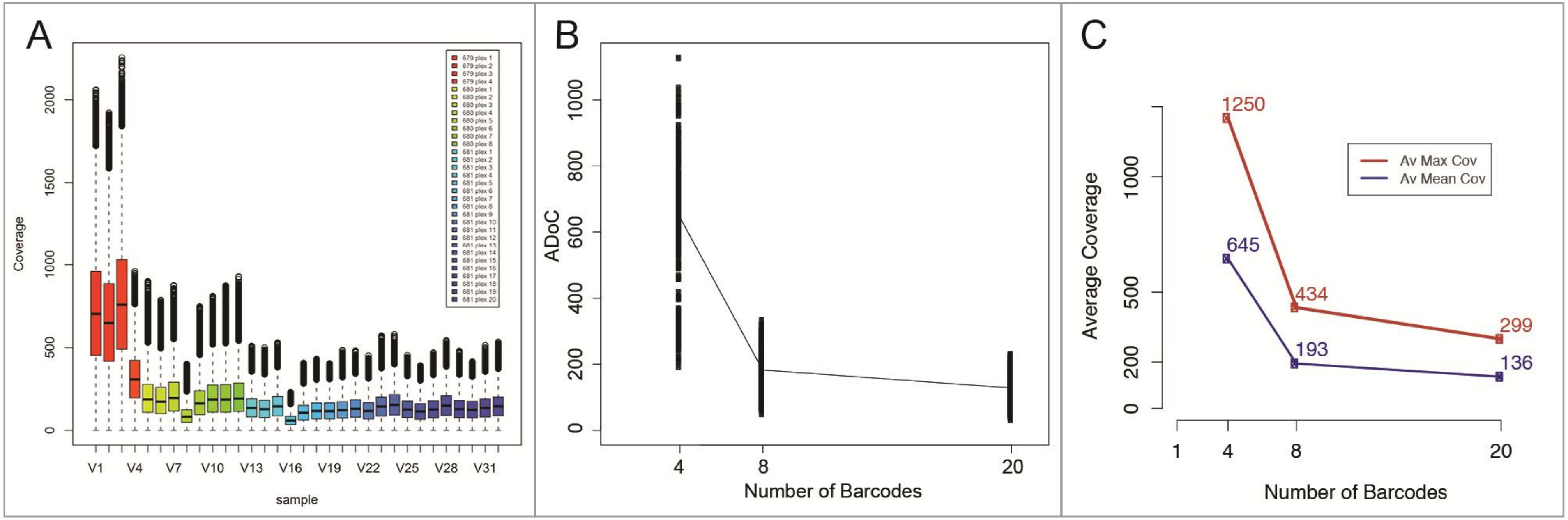

Figure S8. Coverage analysis of barcoded samples (second stage of our *t*NGS test model).

This figure summarizes the coverages generated in the second stage of our experiments, i.e. barcoded *E. coli* libraries enriched for 68 genes. **Panel A**: Box plot showing the coverage ranges for all barcoded samples (red color for the 4-plex, green color for the 8-plex, and blue color for the 20-plex). Within each pool of technical replicates, the coverages were reproducible except for barcode 4 (V4, V8, V16) at each multiplexing level. **Panel B**: Coverage ranges for 4-, 8- and 20-plexing. Each dot shows the average depth of coverage (ADoC) in one of the 68 targeted genes and for one of the barcoded samples. The leftmost column for example contains 68× 4 data points. The lines connect the medians of each pool of barcoded samples. **Panel C**: Average maximum and mean coverage at 4-, 8- and 20-plexing.

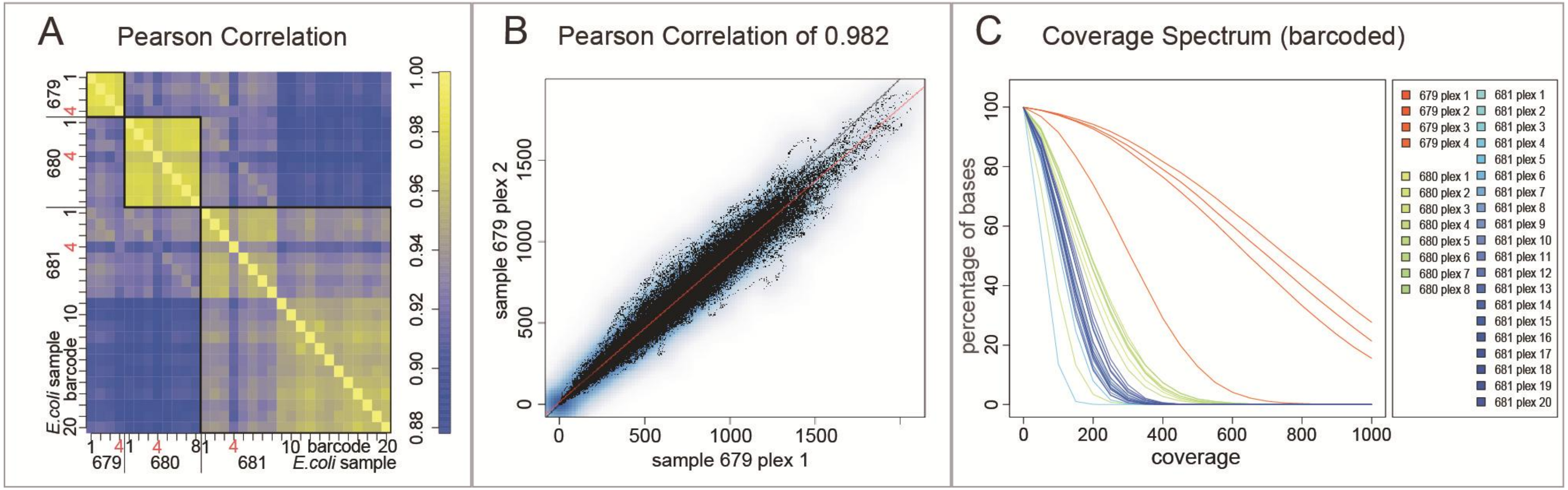

Figure S9. Correlation and reproducibility of multiplexed targeted NGS approach (second stage of our *t*NGS test model).

This figure demonstrates the coverage-based analysis of the enrichment reproducibility across different samples, for the second stage of our experiments: barcoded *E. coli* libraries enriched for 68 genes. **Panel A**: The heat map plot of Pearson correlation coefficients shows that the technical reproducibility of the barcoded samples within the 4- and 8-plex pools of barcoded libraries was good. The samples in the 20-plex pool were less reproducible. The yellow color indicates barcoded samples with high correlation values. The samples with barcode four are distinctive outliers in the 8- and 20-plex pools. **Panel B**: The scatter plot for a pair of 4-plex samples shows a narrow cloud of points, and a high Pearson correlation value of 0.982. This indicates that the investigated approach is reproducible. **Panel C**: Coverage spectrum plot. The curves for each sample show the percentages of bases (y-axis) covered by more than a specific number of reads (x-axis). The outlier curves of each color group (red, green, blue) represent the inferior coverages of the samples with barcode four.
